# Synthesis and Study of Substituted Chalcones Combined with Fluoroazobenzenes—New Photoswitches for Application in Biological Systems

**DOI:** 10.3390/molecules31020362

**Published:** 2026-01-20

**Authors:** Piotr Tobiasz, Damian Mielecki, Anna Stachurska-Skrodzka, Jakub Miętus, Filip Borys, Hanna Krawczyk

**Affiliations:** 1 Department of Organic Chemistry, Faculty of Chemistry, Warsaw University of Technology, Noakowskiego 3, 00-664 Warsaw, Poland; jakub.mietus.stud@pw.edu.pl (J.M.); filip.borys.dokt@pw.edu.pl (F.B.); hanna.krawczyk@pw.edu.pl (H.K.); 2 Department of Neurochemistry, Mossakowski Medical Research Institute, Pawińskiego 5, 02-106 Warsaw, Poland; dmielecki@imdik.pan.pl; 3 Department of Cell Biology and Immunology, Centre of Postgraduate Medical Education, Marymoncka 99/103, 01-813 Warsaw, Poland; anna.stachurska@cmkp.edu.pl

**Keywords:** photopharmacology, colchicine, chalcones, tubulin, photoswitches

## Abstract

Chalcones have garnered significant research interest due to their various medical bioactivities. Several chalcone compounds have been approved for marketing and clinical use in the treatment of various diseases. A critical aspect of the action of chalcones is their effect on microtubules. They are considered an excellent target for chemotherapeutic agents for the treatment of cancer. Consequently, scientists are constantly developing novel chalcone drug agents and also innovative drug delivery strategies. In this manuscript, we report the first synthesis of 12 new visible-light-activated, photoswitchable chalcone-based microtubule inhibitors (17a–17l). Among the obtained compounds, one photoswitch demonstrated light-dependent cytotoxicity in the PC-3 cancer cell line. The IC_50_ value of the *Z* conformer was determined to be 4.75 ± 1.00 μM after 48 h of treatment. The *E* conformer exhibited slightly lower activity compared to the *Z* conformer, with an IC_50_ value of 5.80 ± 0.80 µM following 48 h of incubation. In this study, NMR and UV spectroscopy, along with computational methods, were employed.

## 1. Introduction

**Figure 1:**
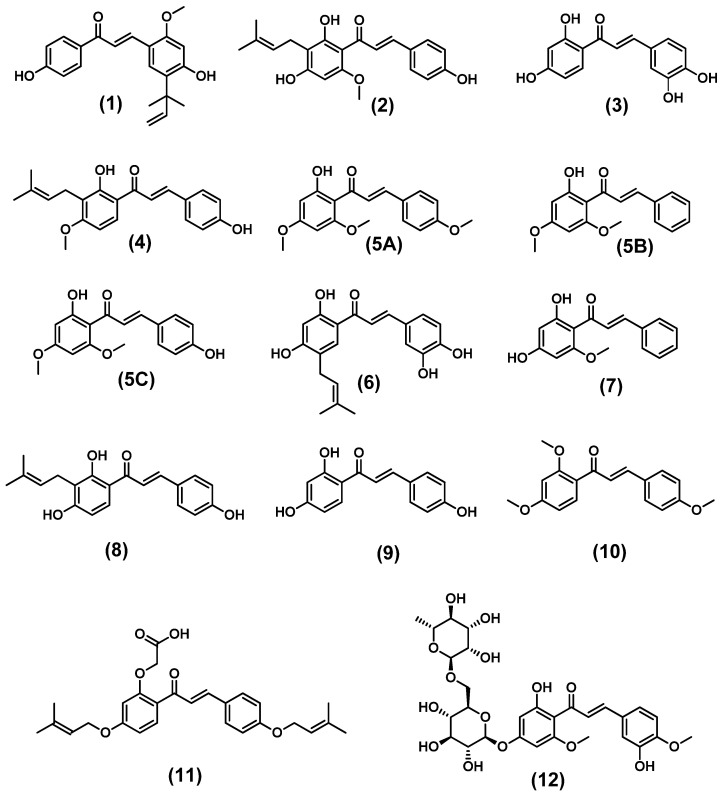
Structures of naturally occurring (**1**–**9**) and of clinically tested chalcones (**10**–**12**) (software: ChemDraw, version 12.0.2.1076).

Chalcones, which serve as precursors to flavonoids, have been extensively studied for their diverse medicinal properties [1], including anti-inflammatory [2,3], anticancer [4,5,6,7], antimalarial [8], and antioxidant activity [9,10]; inhibition of microtubule polymerization [4,11,12,13]; anti-HIV [14], antidiabetic [15], and antileishmanial activity [16]; anti-*Trichomonas vaginalis* activity [17]; antituberculosis activity [18]; and their potential in diagnosing Alzheimer’s disease [19]. The biological activity of these compounds is largely attributed to the presence of an α,β-unsaturated ketone group. Noteworthy anti-mitotic effects have been observed in analogs containing a trimethoxy group [20]. Some of these compounds also exhibit potent inhibition of liver cancer cell proliferation [21]. The most well-known naturally occurring chalcones with positive impacts on human health include licochalcone A (1); xanthohumol (2); butein (3); 4-hydroxyderricin (4); flavokawain A (**5A**), B (**5B**), and C (**5C**); broussochalcone A (6); cardamonin (7); isobavachalcone (8); and isoliquiritigenin (9) (Figure 1) [22,23,24,25]. Several chalcone compounds have been approved for clinical use in treating various diseases, including metochalcone (used as a choleretic/diuretic agent, 10), sofalcone (used as an antiulcer/mucoprotective agent, 11), and hesperidin and methylchalcone (used as vascular protective agents, 12) (Figure 1) [26,27,28,29]. A crucial aspect of chalcone activity is their effect on microtubules [13,30,31,32,33], which consist of α- and β-tubulin heterodimers. These heterodimers possess six known binding sites for compounds to attach [34], one of which is the colchicine binding site, where chalcones have been shown to bind [13,34].

Based on research, it has been established that chalcones bind reversibly to the colchicine binding site with high affinity. Additionally, it has been demonstrated that the number and position of methoxy groups on the aromatic ring are crucial in determining cytotoxicity [35]. Although anti-mitotic agents have been widely used in clinical cancer therapy for several decades [36,37], they still present significant limitations. Issues such as high toxicity, poor solubility, and low oral bioavailability make them less ideal for cancer treatment. As a result, scientists are continuously working on developing new anti-mitotic agents and innovative strategies to overcome these challenges.

Photopharmacology has the potential to offer a promising and less invasive alternative to traditional therapeutic methods, with the potential to either replace or complement existing treatments [38,39]. It holds significant promise for medical applications by enabling photo-controlled, reversible, and selective targeting of specific sites in the human body using small drug molecules that can change structure upon light exposure. Photopharmacological agents are bioactive molecules that have been modified with photoswitches—structures that undergo light-induced changes. Photochromic molecular switches, which are frequently used in photopharmacology, are molecules that undergo reversible transitions between stable thermodynamic states when exposed to electromagnetic radiation in the visible spectrum. These photoinduced changes can be harnessed, for instance, to control the absorption or release of biologically active molecules.

There are many strategies for the design and modulation of photochromic molecular switches [38]. One such approach is the **Bona Fide Extension** [38], which involves adding an aromatic ring containing a photoisomerizable system—such as azobenzene—to the original drug structure without removing existing substituents. This extension can influence interactions with the receptor’s hydrophobic pocket, depending on the isomeric state (*E* or *Z*). Another strategy is **Structure-Based Design** [38], which utilizes crystallographic data and molecular docking to plan specific modifications of the parent compound. Through binding site analysis, researchers can identify optimal positions for incorporating photoswitchable substituents. A third approach, known as **Azologization** [38], relies on the bioisosteric replacement of a structural fragment within a pharmacologically active compound by a photoswitchable unit—most commonly azobenzene—so that the configuration change alters the compound’s biological activity. The **Insertion Approach** [38] involves placing a photochromic molecular switch, such as azobenzene, between two atoms of the parent molecule. This switch then acts as an internal, light-responsive linker that modulates the compound’s conformation and function upon photoactivation. Finally, in **Linker Replacement in Bifunctional Ligands** [38], the photochromic molecular switch replaces the linker connecting two pharmacophoric units in complex ligands. Light-induced changes in the spatial length of the switch then modulate the molecule’s ability to form effective interactions. These strategies illustrate the diverse approaches to designing photochromic molecular switches that enable precise, temporally and spatially controlled pharmacological actions.

In our research, we explored hybrid compounds (Bona Fide Extension) which combine chalcone derivatives with fragments containing azo groups and fluorine substituents—both of which can function as molecular switches. The small size of fluorine allows bioisosteric substitution for hydrogen in many biologically active molecules, resulting in potent compounds without significant stereochemical changes [40,41]. Additionally, this substitution is known to influence the reactivity and stability of the compounds, due to the carbon–fluorine bond’s resistance to metabolic transformations and the changes in acidity caused by the electronegativity difference between the atoms [42]. Several studies have reported enhanced biological activity in fluorinated methoxychalcones as well [43,44]. A highly intriguing concept was introduced by Heck’s team [45,46] and later expanded upon by Feringa and Szymański [47,48,49]. The incorporation of halogen atoms (such as fluorine or chlorine) into azo molecules allows for the separation of *n*→π* absorption bands of the *E* and *Z* stereoisomers in the visible (VIS) region of the UV–VIS spectrum, distinguishing them from the π→π* bands found in the ultraviolet (UV) region. This separation facilitates the selective analysis and activation of each geometric isomer. Additionally, red-shifted azobenzene derivatives have proven to be effective molecular photoswitches, broadening their potential applications in photopharmacology. Fluorinated azobenzenes are, in particular, well-suited for designing visible-light-responsive systems due to their stable and reversible photoconversions, along with compatibility with biological tissues [40].

Continuing our research on hybrid molecules, the current study focuses on combining chalcone and azobenzene moieties into a single system to enhance their interaction with microtubules, aiming to improve their potential as cytostatic agents.

## 2. Results and Discussion

### 2.1. Synthesis

**Scheme 1:**
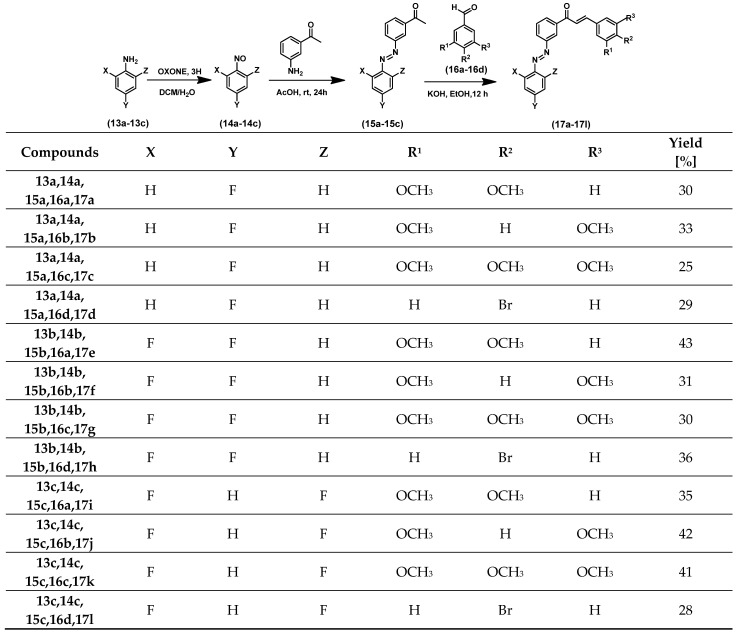
Three-step synthesis of compounds (**17a**–**17l**) (software: ChemDraw, version 12.0.2.1076).

As part of our search for biologically active compounds, we synthesized a series of novel fluoro-substituted chalcone derivatives linked to azo switches which have not been reported before (Scheme 1). Through a three-step synthetic route, a series of compounds (**17a**–**17l**) was obtained. In the first step, various nitroso derivatives (**14a**–**14c**) were synthesized via the reaction of appropriately substituted anilines (**13a**–**13c**) with oxone in a DCM/water solution. Oxone, as a mild oxidizing agent, facilitates the transformation of the amine group into a nitroso group. In the second step, a condensation reaction was carried out between the three nitroso fluorobenzenes (**14a**–**14c**) and 1-(3-aminophenyl)ethanone. In this reaction, azo bonds were formed between the nitroso group of the fluoroazobenzenes and the amine group of 1-(3-aminophenyl)ethanone, yielding compounds (**15a**–**15c**). Finally, the hybrid molecules (**17a**–**17l**), containing both chalcone and azo moieties, were obtained through an aldol condensation reaction between substituted aldehydes (**16a**–**16d**) and the azo compounds (**15a**–**15c**).

### 2.2. Molecular Docking Simulation

As previously stated, chalcones bind to the colchicine binding site and so do derivatives of *azo*-stilbenes [50]. To check how the obtained compounds interact with tubulin, we performed docking of selected obtained compounds (**17b**), (**17d**), (**17f**), (**17h**), (**17j**), and (**17l**) to tubulin in configuration *E* or *Z*. The molecular docking of the selected compounds of isomers *E* and *Z* into the 3D X-ray structure of tubulin (PDB code: 1SA0) [51] was carried out using the AutoDock Vina software, version 1.2.5 (http://vina.scripps.edu/, accessed on 27 May 2024) (the Broyden–Fletcher–Goldfarb–Shanno (BFGS) method) [52]. Firstly, density functional theory (DFT) calculations were performed [53,54]. In the calculations, the B3LYP functional and the 6-31G* basis set was employed and the continuum model (PCM; Gaussian 03W, see Section S1 in Supplementary Materials) was used to simulate the effects of the solvent, DMSO. We modeled the interaction between (**17b**), (**17d**), (**17f**), (**17h**), (**17j**), and (**17l**) *E* and *Z* isomers as well as the colchicine binding sites of α- and β-tubulin (see Section S1.2 in Supplementary Materials). In the binding mode, the *E* and *Z* isomers of compounds (**17b**), (**17d**), (**17f**), (**17h**), (**17j**), and (**17l**) bind to the colchicine binding site of tubulin via hydrophobic interactions, and hydrogen bonds stabilize binding (see Section S1.2 in Supplementary Materials). The calculated binding energies were used as criteria for selecting the docking cluster to be evaluated (see Section S1.2 in Supplementary Materials), where the binding mode of the lowest-energy structure was identified (selection of the docking cluster corresponding to the lowest-energy structure of the molecule under investigation). For the selected structures (**17b*E***), (**17b*Z***), (**17d*E***), (**17d*Z***), (**17f*E***), (**17f*Z***), (**17h*E***), (**17h*Z***), (**17j*E***), (**17j*Z***), (**17l*E***), and (**17l*Z***), the estimated binding free energies were as follows:

−8.9 kcal/mol for **17b*E***;
−8.9 kcal/mol for **17b*Z***;
−9.4 kcal/mol for **17d*E***;
−9.8 kcal/mol for **17d*Z***;
−9.4 kcal/mol for **17f*E***;
−9.0 kcal/mol for **17f*Z***;
−9.4 kcal/mol for **17h*E***;
−10.0 kcal/mol for **17h*Z***;
−9.2 kcal/mol for **17j*E***;
−9.1 kcal/mol for **17j*Z***;
−9.5 kcal/mol for **17l*E***;
−10.0 kcal/mol for **17l*Z***.

For comparison, the binding free energy of the control compound colchicine was −8.8 kcal/mol [55]. All the models of the investigated compounds showed similar interactions with the colchicine binding site (in terms of docking pose and binding energy), comparable to those observed for colchicine itself [56]. The estimated binding free energies of the E and Z isomers differ only slightly and should be interpreted with caution. The compounds (**17b*E***), (**17b*Z***), (**17d*E***), (**17d*Z***), (**17f*E***), (**17f*Z***), (**17h*E***), (**17h*Z***), (**17j*E***), (**17j*Z***), (**17l*E***), and (**17l*Z***) were embedded in the hydrophobic pocket of the tubulin binding site—specifically, the region normally occupied by the A ring of colchicine. Each compound formed van der Waals contacts with specific amino acid residues as follows:

- (**17b*E***): Leuβ255, Leuβ248, Ileβ378, Alaβ250;
- (**17b*Z***): Alaβ316, Leuβ255, Leuβ248, Lysβ352, Lysβ254, Ser178;
- (**17d*E***): Lysβ254, Alaβ250, Leuβ255, Leuβ248;
- (**17d*Z***): Ileβ378, Lysβ352, Lysβ254, Leuβ255, Leuβ248, Leuβ242, Alaβ317, Alaβ250;
- (**17f*E***): Leuβ248, Lysβ254, Alaβ250;
- (**17f*Z***): Lysβ352, Alaβ316, Leuβ255, Leuβ248, Lysβ254, Alaβ250, Ser178;
- (**17h*E***): Leuβ255, Leuβ248, Lysβ254, Alaβ250;
- (**17h*Z***): Ileβ378, Lysβ352, Lysβ254, Alaβ317, Leuβ255, Leuβ248, Leuβ242, Alaβ250;
- (**17j*E***): Lysβ352, Lysβ254;
- (**17j*Z***): Ileβ378, Alaβ316, Leuβ255, Leuβ248, Lysβ254, Alaβ250, Asn258;
- (**17l*E***): Lysβ254, Leuβ255, Leuβ248, Alaβ250;
- (**17l*Z***): Ileβ378, Alaβ317, Alaβ316, Lysβ352, Leuβ255, Leuβ248, Leuβ242, Lysβ254, Alaβ250.

All interactions for the compounds explored are provided in Section S1 in Supplementary Materials. In all cases, the Z isomers were docked “deeper” into the colchicine binding pocket of tubulin. These docking results indicate the compounds studied can adopt binding conformations within the tubulin colchicine site similar to colchicine. Moreover, these in silico findings strongly support that the compounds bind to the colchicine-binding site and justify further experimental investigation but do not constitute definitive evidence of functional binding or biological activity in cells or organisms; orthogonal biochemical and cellular validation (e.g., competition assays, binding kinetics, and microtubule polymerization/inhibition assays) is required. These results support our design hypothesis and provide a clear rationale for follow-up experimental evaluation of these hybrid compounds. In summary, molecular docking generates hypotheses on ligand–protein interactions but cannot reliably predict cellular efficacy or selectivity without experimental validation.

It should be emphasized that the observed differences are not to be interpreted quantitatively.

### 2.3. NMR and UV–VIS Spectra

**Figure 2:**
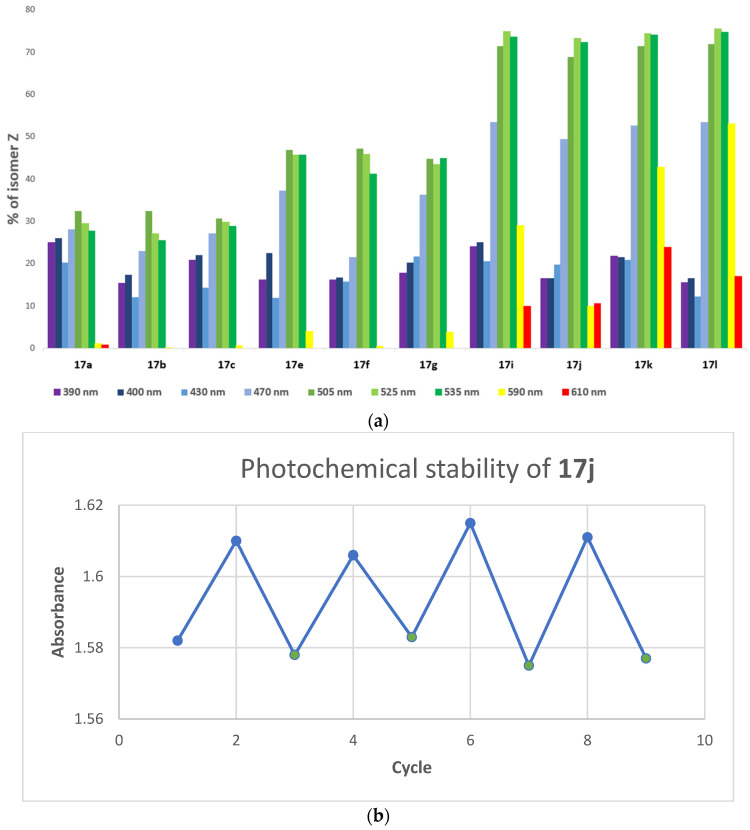
(**a**) Photostationary state (PSS) compositions for chalcones (**17a**–**17c**, **17e**–**17g**, and **17i**–**17l**) determined by ^19^F NMR analysis (c ≈ 100 mM in DMSO-*d*_6_), presented in a bar chart; (**b**) representative multiple photoswitching rounds of 60 µM of (**17j**) in DMSO by pulsed green (525 nm) and blue (430 nm) light illumination. The photochemical stability of the drug is analyzed via repeated cycles of irradiation (software: Microsoft Excel).

Azo compounds have been applied in various fields due to their ability to undergo structural changes, with one of the most interesting applications being the direct conversion of light into mechanical energy [57]. However, azobenzenes generally face two main limitations that hinder their practical use in biological and materials sciences. The first is the need for UV light to induce the *E*→*Z* isomerization through π→π* excitation, which can potentially interfere with and damage the surrounding environment. The second limitation is the typically incomplete reverse *Z*→*E* photoisomerization, which is induced by irradiation in the visible region. In this region, the *n*→π* bands of the *E* and *Z* isomers largely overlap, making it difficult to achieve *E*-rich photostationary states (PSSs) after back-switching. These limitations can be overcome by modifying azobenzene in such a way that the *n*→π* bands of the *E* and *Z* isomers are sufficiently separated [58]. If the separation is large enough, the two *n*→π* bands can be distinct, allowing visible light to selectively isomerize both isomers with high to complete *E*/*Z* photoconversions. We are looking for switches that operate within the visible light range of 380–750 nm, which is harmless. Fluorine-substituted azobenzenes are well-suited for designing visible-light-responsive systems, offering stable and bidirectional photoconversions along with tissue-compatible properties [40]. We confirmed that the compounds we synthesized successfully dock with tubulin. In the next step, we tested whether the added *azo* compound would meet our expectations as a visible light switch. For this purpose, we used NMR spectroscopy. We measured ^19^F spectra of (**17a**–**17c**, **17e**–**17g**, and **17i**–**17l**) at nine wavelengths from 390 nm to 610 nm to check which wavelengths produce the most *Z* isomer after exposure (see Section S4.2 in Supplementary Materials). We selected DMSO as the solvent due to its ability to dissolve both polar and nonpolar molecules, which is essential for the analytical methods employed in this study. Its intermediate polarity also makes it a good surrogate for both organic and aqueous solvents. Additionally, DMSO is commonly used for preparing stock solutions, which are then illuminated and diluted into aqueous systems for assessing biological activity [59,60]. Unfortunately, the bromine compounds (**17d** and **17h**) are poorly soluble in DMSO and we did not measure their ^19^F NMR spectra. The results obtained for the tested compounds (**17a**–**17c, 17e**–**17g,** and **17i**–**17l**) are summarized in Figure 2 (and see Section S4.2 in Supplementary Materials).

**Figure 3:**
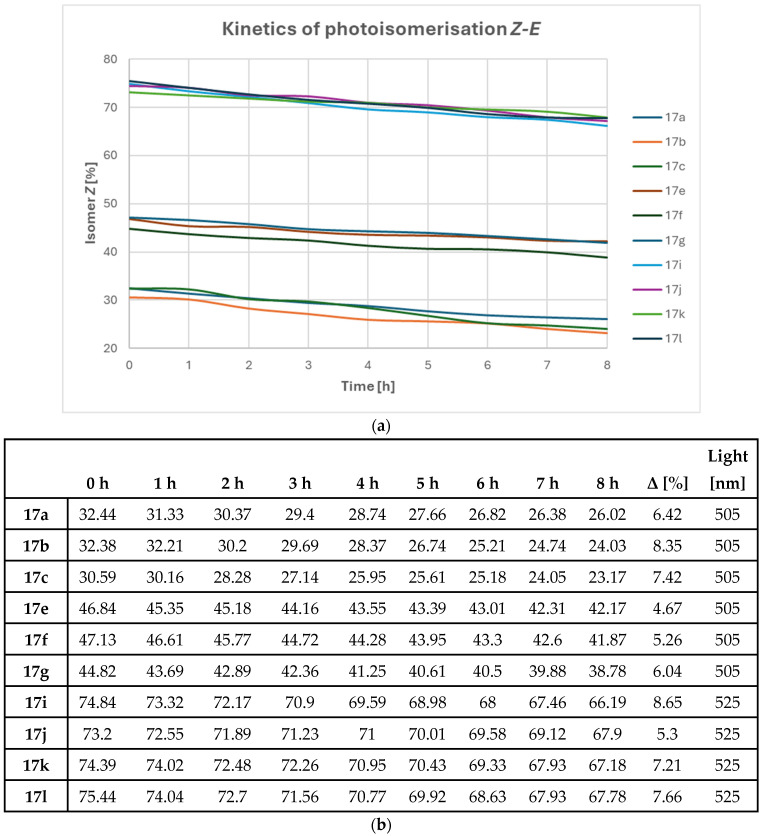
(**a**) Changes over time (photoisomerization kinetics) of transitions from *Z* to *E* upon irradiation for (**17a**–**17c**, **17e**–**17g**) irradiated with 505 nm and for (**17i**–**17l**) irradiated with 525 nm (for sample concentration of 100 mM in DMSO); (**b**) data presented in table (software Microsoft Excel).

We can observe that for electromagnetic radiation in the range of wavelengths 390 nm to 610 nm, transitions from *E* to *Z* of about 0–76% are obtained. The best results for all compounds are in visible wavelengths 505 nm or 525 nm (30.59–75.44%). From analyzing Figure 2 we can draw the following conclusions: all synthesized compounds undergo switching under visible light (this is important because UV radiation can damage the DNA of cells, inhibiting replication and, as a result, preventing cell division). For the 2,6-difluoro substituents (**17i**), (**17j**), (**17k**), and (**17l**) with values of 74.84%, 73.2%, 74.39%, and 75.44%, respectively, the largest share of the *Z* configuration is at (525 nm) and, for the rest of (**17a**), (**17b**), (**17c**), (**17e**), (**17f**), and (**17g**), it is in range of 30.57–47.13% at 505 nm. Methoxy or bromo substituents do not affect the amount of the *Z* isomer. Multiple cycles of photoreversible switching under alternating green and blue light irradiation, with no significant photobleaching or degradation, confirmed the repeatable and robust photochromic conversion of the synthesized azobenzenes (Figure 2b). Then we measured changes over time (transitions from *Z* to *E*, upon irradiation with 505 nm for (**17a**–**17c**) and (**17e**–**17g**), and 525 nm for (**17i**–**17l**) compounds (Figure 3, and see Section S4.3 in Supplementary Materials).

After eight hours the (**17a**–**17c**, **17e**–**17g**, and **17i**–**17l**) compounds change to levels above 4.5% (from 8.65% to 4.67%) for configuration from *Z* to *E*. This slight decrease in the *Z* to *E* isomer after 8 h is observed for all tested compounds regardless of the amount of substituted fluorine.

However, the amount of fluorine affects the amount of *Z* isomer formed after irradiation for 12 h (by night). Additivity can be observed at the following values: for compounds (**17a**–**17c**) about 30.5–32.44%, for molecules (**17e**–**17g**) about 44.8–47.1% (Δ about 14%), and for hybrids (**17i**–**17l**) 73.2–74.8% (Δ about 28%) (see Section S4.2 in Supplementary Materials). This phenomenon is probably related to the substitution of fluorine in the *ortho* position. When there is one fluorine atom in the *ortho* position, the amount of *Z* isomer increases by 14%; when there are two fluorines in the *ortho* position, the amount of *Z* isomer increases by approximately 28%. It is worth noting that *ortho*-fluorine atoms reduce the electron density in the nearby N=N bond, lowering the *n*-orbital energy [46]. This effect results in more *Z* isomers in the case of (**17i**–**17l**) with *ortho*-disubstituted fluorines. The next step in our experiments was to check if the obtained compounds show separation of the *n*→π* absorption bands in the visible range of the spectrum. This is related to the possibility of selective excitation of *E*/*Z* isomers, which is why the separation of these bands is important. The action of photopharmaceuticals containing skeletal fragments of azobenzene as a functionalizing unit allows control of biological functions with precision in space and time [61,62].

For this purpose, the *UV–VIS* spectra were measured for all synthesized hybrids (**17a**–**17l**) at concentrations from 20 to 500 µM in dimethyl sulfoxide (DMSO) in light at λ 505 nm and for (**17a**–**17c**), (**17e**–**17g**), and (**17i**–**17l**) at λ 525 nm, respectively, as detailed in Table 1 (see Section S4.4 in Supplementary Materials).

We observed strong π→π* transition bands at short wavelengths in the *UV* range below 400 nm for the *E*/*Z* stereoisomers of all compounds (**17a**–**17l**). In the case of compounds (**17b**, **17d**), (**17f**, **17h**), and (**17j**, **17l**), the bands π→π* and *n*→π* are separated for both stereoisomers *E*/*Z* (Table 1 and see Section S4.4 in Supplementary Materials). Moreover, *n*→π* bands occur for *E*/*Z* isomers in the visible range: 417.4 nm–448.6 nm (Table 1). The differences range from 8.1 to 30.9 nm. The largest separation of bands *n*→π* is for (**17j**) and (**17l**) at 30.9 nm and 30.7 nm, respectively. The molar absorption coefficient (***ε_max_*** (*n*→π*), transition probability) in the case of *Z* isomers is higher than for the *E* isomers. In compounds (**17b**, **17d**), (**17f**, **17h**), and (**17j**, **17l**), the selective excitation of *E*/*Z* isomers is possible. From the physicochemical point of view, (**17j**) compounds show good characteristics for stable and bidirectional photoswitching.

### 2.4. Cell Proliferation Analysis

**Figure 4:**
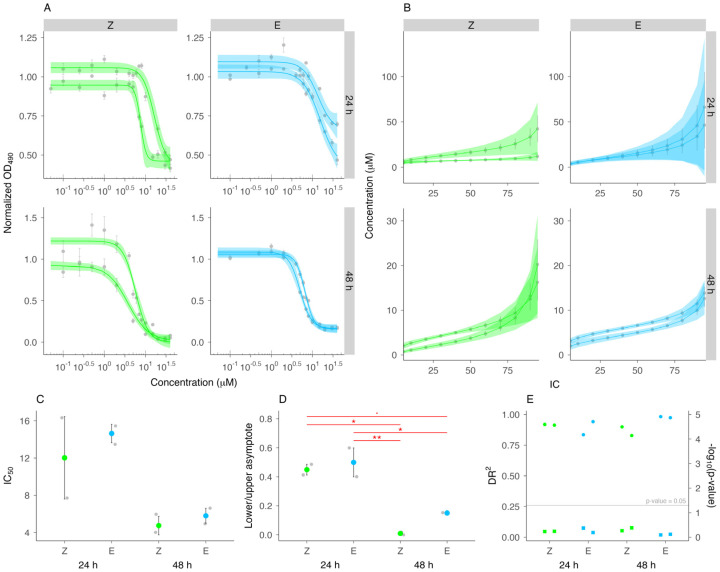
The proliferation of PC-3 in the presence of the (**17j**) compound was measured with MTS assay and the normalized OD_490_ values fit with the 4PL model. The normalized OD_490_ values and fit models are shown separately for each conformer of (**17j**) compound, *Z* and *E*, and the treatment time, 24 and 48 h (**A**), with the values for the inhibitory concentrations in the range of 5 to 90% in the potency spectrum plot (**B**). The mean IC_50_ and the normalized lower asymptote values are shown (**C**,**D**). The selected metric of the goodness of fit, deviance R^2^, is indicated (**E**). The error bars in each panel show the standard errors and the ribbons show 95% confidence intervals. The selected groups were compared with the two-way ANOVA with Tukey’s post hoc test. The significant differences between groups are shown by red horizontal lines with adjusted *p*-values marked as follows: 0–0.001 (***), 0.001–0.01 (**), 0.01–0.05 (*), and 0.05–0.1 (.).

After the spectral analysis, we decided to check the impact of the selected compound **17j** on a specific cancerous cell line. PC-3 cells exhibit characteristics that closely mimic the advanced stages of a cancer, making them particularly relevant for evaluating therapeutic strategies aimed at this aggressive disease. The selected compound, in the chosen concentration range, up to 40 µM, exerted a clearly visible negative impact on the proliferation of PC-3 cells (Figure 4). This effect increased when the cells were treated for an additional 24 h. The overall 4PL model was shown to be appropriate for the data obtained (Figure 4A), with the general tendency of the need to increase the concentration of the compound for a desired increased value of inhibitory concentration, IC (Figure 4B). The IC_50_ values for the *Z* conformer were assessed to equal 12.03 ± 4.42 (95% CI: 9.60, 14.46) and 4.75 ± 1.00 µM (95% CI: 4.24, 5.26) for 24 and 48 h treatment, respectively. The *E* conformer was slightly less active as compared to the *Z* conformer, with IC_50_ values of 14.64 ± 1.00 (95% CI: 9.88, 19.40) and 5.80 ± 0.80 µM (95% CI: 5.45, 6.16) after 24 and 48 h of incubation, although with no statistically significant difference (Figure 4C). The inhibition of proliferation, expressed as the ratio of lower to upper asymptote, assessed from the fit model, meaning the maximal cell proliferation in the experimental conditions, showed a very similar tendency as the IC_50_ values (Figure 4D). The least effective was the *Z* conformer, with 44.9 ± 3.7 (95% CI: 37.6, 52.2) and 0.9 ± 0.9% (95% CI: −4.3, 6.2) values of normalized asymptote, after 24 and 48 h of treatment, respectively. On the other hand, the *E* conformer showed the least pronounced inhibitory activity, as expressed in terms of the normalized limit, assessed as 50.0 ± 9.9 (95% CI: 37.5, 62.4) and 15.0 ± 0.2% (95% CI: 11.7, 18.4), after 24 and 48 h of incubation (Figure 4D). Regarding both conformers after 48 h, the difference was supported by statistical significance (*p*-value < 0.05, two-way ANOVA with Tukey’s post hoc test).

The deviance R^2^ (DR^2^) values, a measure indicating how close the model is to the perfect fit, are all above 0.83 for the assessed models, all with statistical significance (*p*-values < 0.05), showing a good overall fitness for each biological repeat (Figure 4E).

**Figure 5:**
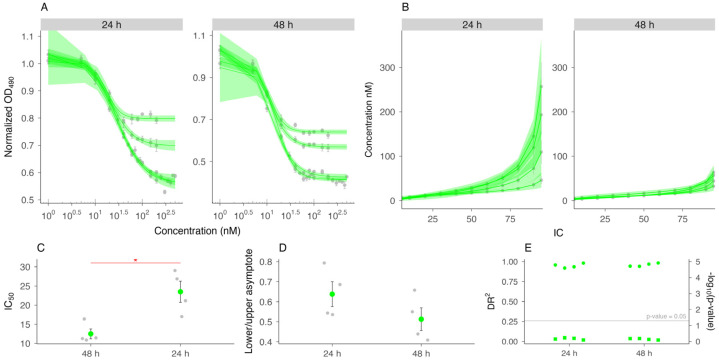
The proliferation of PC-3 in the presence of colchicine (green) was measured with MTS assay and the normalized OD_490_ values fit with the 4PL model. The normalized OD_490_ values and fit models are shown separately for each treatment time, 24 and 48 h (**A**), with the values for the inhibitory concentrations in the range of 5 to 90% in the potency spectrum plot (**B**). The mean IC_50_ and the normalized lower asymptote values are shown (**C**,**D**). The selected metric of the goodness of fit, deviance R^2^, is indicated (**E**). The error bars in each panel show the standard errors and the ribbons show 95% confidence intervals. The selected groups were compared with the two-way *t*-test. The significant difference between groups is shown by red horizontal line with *p*-value marked as follows: 0–0.001 (***), 0.001–0.01 (**), 0.01–0.05 (*), and 0.05–0.1 (.).

The activity of colchicine against PC-3 cells was evaluated in similar conditions, as a control study. The colchicine was characterized by IC_50_ values of 23.50 ± 2.75 (95% CI: 16.85, 30.15) and 12.50 ± 1.26 nM (95% CI: 9.55, 15.45) after 24 and 48 h of treatment, respectively (Figure 5A–C). The data presented are in good agreement with the IC_50_ value of 57.56 nM after 24 h of treatment, obtained by Ergul and Bakar-Ates [63], corroborating the reliability of the experimental setup. Moreover, the normalized asymptote values were calculated as equal to 63.8 ± 6.2 (95% CI: 59.7, 67.9) and 51.3 ± 5.7% (95% CI: 47.1, 55.5) after 24 and 48 h of treatment of PC-3 cells with colchicine (Figure 5D). Similarly, the model fitted very well as assessed by the DR^2^ values > 0.92 (Figure 5E).

**Figure 6:**
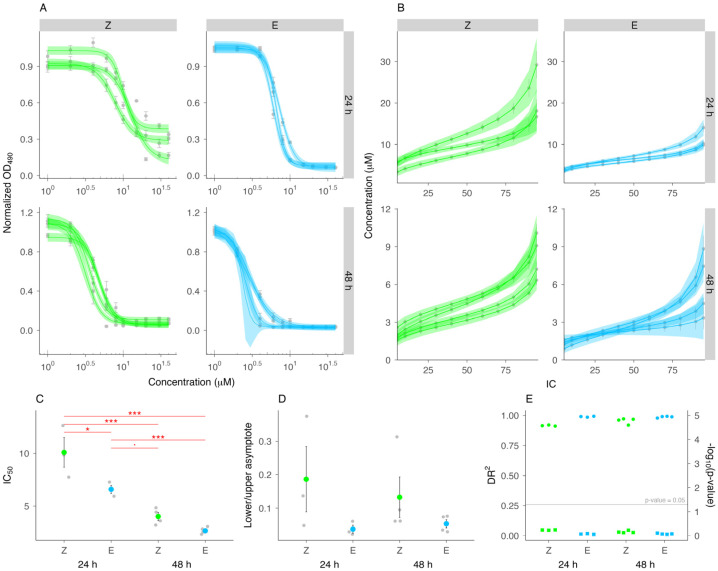
The proliferation of BJ fibroblasts in the presence of the (**17j**) compound was measured with MTS assay and the normalized OD_490_ values fit with the 4PL model. The normalized OD_490_ values and fit models are shown separately for each conformer of (**17j**) compound, *Z* (green) and *E* (blue), and the treatment time, 24 and 48 h (**A**), with the values for the inhibitory concentrations in the range of 5 to 90% in the potency spectrum plot (**B**). The mean IC_50_ and the normalized lower asymptote values are shown (**C**,**D**). The selected metric of the goodness of fit, deviance R^2^, is indicated (**E**). The error bars in each panel show the standard errors and the ribbons show 95% confidence intervals. The selected groups were compared with the two-way ANOVA with Tukey’s post hoc test. The significant differences between groups are shown by red horizontal lines with adjusted *p*-values marked as follows: 0–0.001 (***), 0.001–0.01 (**), 0.01–0.05 (*), and 0.05–0.1 (.).

The activity of the **17j** compound was also assessed against non-cancerous BJ fibroblasts. The results obtained generally indicate an overall similar extent of toxicity of the above-mentioned chemical, with IC_50_ values for the *Z* conformer being 10.09 ± 1.40 (95% CI: 9.57, 10.61) and 4.02 ± 0.39 µM (95% CI: 3.87, 4.17) after 24 and 48 h treatment, respectively. The *E* conformer revealed to be even slightly more potent, with IC_50_ values of 6.59 ± 0.38 (95% CI: 6.42, 6.75) and 2.66 ± 0.15 µM (95% CI: 2.49, 2.83) after 24 and 48 h treatment, respectively, with statistical significance between both conformers after 24 h of incubation. Both conformers were statistically more potent when the treatment was performed for 48 h (two-way ANOVA with Tukey’s post hoc test) (Figure 6A). The overall activity, as measured by the normalized asymptote, indicated even greater potency against the BJ fibroblasts, as compared to PC-3 cells, with the growth inhibition of the *Z* conformer reaching 18.7 ± 9.7 (95% CI: 16.2, 21.1) and 13.3 ± 6.1% (95% CI: 11.6, 14.9) after 24 and 48 h treatment, respectively. On the contrary, the *E* conformer was shown to be less potent in the experimental setup, with growth inhibition values of 37.1 ± 1.2 (95% CI: 2.0, 5.4) and 5.3 ± 1.2% (95% CI: 4.0, 6.6) after 24 and 48 h of incubation, respectively (Figure 6D). There were no statistically significant differences regarding the growth inhibition levels. The model showed relatively good fit, as shown by the high values of DR^2^ > 0.91 (Figure 6E).

The investigation into the cytotoxic potential of compound **17j** revealed a clear time-dependent response, with its activity increasing from 24 to 48 h of treatment. This pattern aligns with findings from large-scale cytotoxicity profiling, which demonstrate that the kinetics of cell death can be characteristic of the mechanism of action of a given compound. Specifically, delayed cytotoxic responses have been associated with pathways involving slower cellular processes, such as cell cycle disruption [64]. Interestingly, the cancerous PC-3 cell line exhibited slightly greater resistance to **17j** compared to the non-cancerous BJ fibroblasts. This differential sensitivity was observed both in terms of absolute IC_50_ values, which were higher for PC-3 cells by several µM, and in the fold-change in growth inhibition, which ranged from 1.3 to 2.8. Interestingly, the *E* conformer of compound **17j** showed a larger IC_50_ difference between PC-3 and BJ cells (2.2-fold) than the *Z* conformer, which had nearly identical IC_50_ values in both cell lines, regardless of treatment duration.

A significant finding was that both the *Z* and *E* conformers of **17j** displayed similar cytotoxic activity against both PC-3 and BJ cell lines. This observation agrees with our molecular docking results, which showed only a minimal difference (0.1 kcal/mol) in the calculated affinity energies of the two conformers for the putative target. The similar biological activity suggests that the isomerization state may not critically alter interaction of the compound with its biological target. This could be explained by a flexible binding pocket or by the fact that the isomerization in **17j** is thermally irreversible under physiological conditions, locking the molecule in a specific bioactive conformation. Such irreversible isomerization has been documented in other functional molecular systems, including stilbene derivatives [65]. Finally, a comparative analysis of the potency spectrum suggests that compound **17j** may share certain functional similarities with the microtubule-disrupting agent colchicine. However, given the reliance on a single cytotoxicity assay and indirect computational predictions, this observation should be regarded as preliminary. Considering the limited number of cell lines studied, no conclusions can be drawn at this stage regarding cancer selectivity.

Colchicine exerts its potent cytotoxic effect by binding to tubulin and inhibiting its polymerization into microtubules, thereby disrupting cytoskeletal functions and cellular division [66,67]. The weaker activity of **17j** could be attributed to a lower binding affinity to tubulin or reduced cellular uptake, which may conversely suggest a wider therapeutic window than colchicine. This correlation provides a preliminary foundation for future structural optimization and more detailed biological validation.

### 2.5. Cell Cycle Analysis

**Figure 7:**
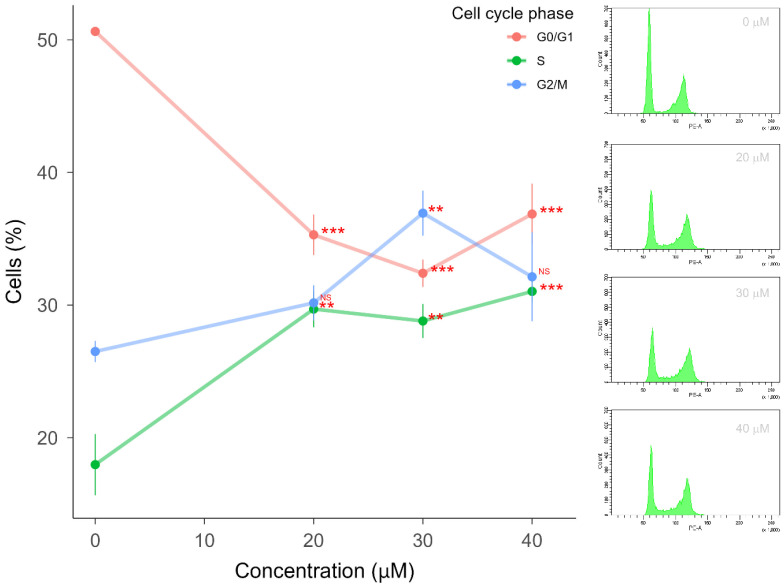
The impact of the *Z* isomer of the (**17j**) compound on the cell cycle progression of PC-3 cells was assessed by flow cytometry. The cell numbers in each cell cycle phase, G0/G1, S, and G2/M, were measured, as indicated by exemplary histograms. The values of cell number ratios in each of the cell cycle phases for each treatment were compared to the 0 µM concentration (the cells treated with 0.5% DMSO) with the two-way ANOVA with Tukey’s post hoc test. The significance of *p*-value was marked as follows: 0–0.001 (***), 0.001–0.01 (**), 0.01–0.05 (*), 0.05–0.1 (.), and >0.1 (not significant, NS).

The impact of the *Z* conformer of the **17j** compound on the progression of the cell cycle of PC-3 cells was assayed at 20, 30, and 40 µM concentrations (Figure 7). Results showed that **17j** significantly increased the accumulation of cells in the S and G2/M phases and considerably decreased the cell population in the G0/G1 phase. The percentage of PC-3 cells in the G2/M phase was 30.2% (20 µM), 37.0% (30 µM), and 32.1% (40 µM), as compared to the control cells (26.5%). The percentage of PC-3 in the S phase was higher and increased by 29.7% (20 µM), 28.8% (30 µM), and 31% (40 µM). Almost all the changes were statistically significant with *p* < 0.01 (two-way ANOVA with Tukey’s post hoc test). These results indicated that the *Z* conformer of the **17j** compound can arrest the PC-3 cells at G2/M with substantial accumulation of cells in the S phase.

**Figure 8:**
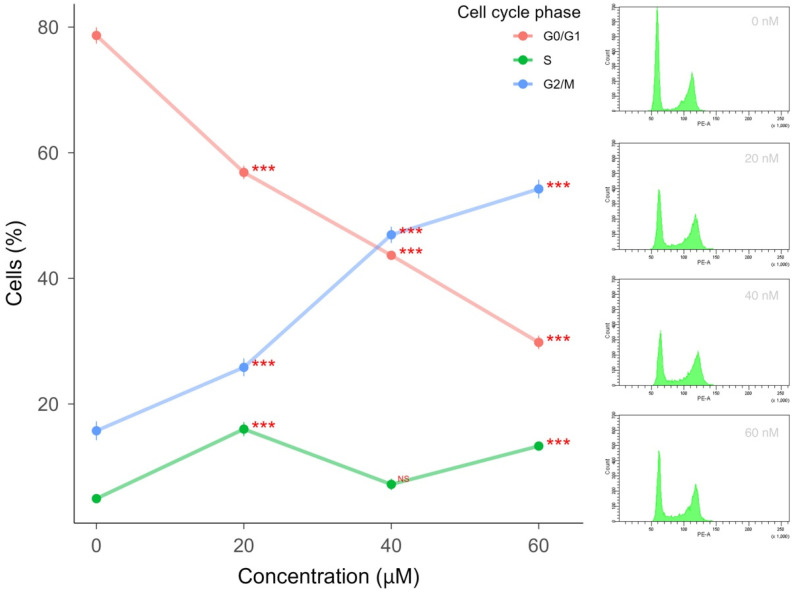
The impact of colchicine on the cell cycle progression of PC-3 cells was assessed by flow cytometry. The cell numbers in each cell cycle phase, G0/G1, S, and G2/M, were measured, as indicated by exemplary histograms. The values of cell number ratios in each of the cell cycle phases for each treatment were compared to the 0 nM concentration (the cells treated with 0.5% DMSO) with the two-way ANOVA with Tukey’s post hoc test. The significance of *p*-value was marked as follows: 0–0.001 (***), 0.001–0.01 (**), 0.01–0.05 (*), 0.05–0.1 (.), and >0.1 (not significant, NS).

Similarly, colchicine also stalled the PC-3 cells in the G2/M phase, with increasing concentration from 20 to 60 nM. The percentage of cells in the G2/M phase rose from 15.7 (20 nM) to 54.2% (60 nM). Consequently, the ratio of cells in the G0/G1 phase decreased accordingly (Figure 8). Almost all the changes, as compared to cells treated with a vehicle (DMSO), were statistically significant with an adjusted *p* value < 0.001 (two-way ANOVA with Tukey’s post hoc test). The results obtained are in accordance with the widely accepted action of colchicine in arresting the cell cycle in the G2/M phase [68]. The one profound difference can be seen regarding the impact of colchicine on the cell cycle progression of PC-3 cells, as compared to the **17j** compound. The presence of colchicine did not change the ratio of the cells in the S phase as much as the **17j** chemical.

Although the observed alterations in cell cycle progression are consistent with the effects of microtubule-interfering agents, the present data do not allow for definitive conclusions regarding the exact molecular mechanism of action. Further mechanistic studies will be required to confirm direct tubulin engagement and downstream signaling pathways.

**Figure 9:**
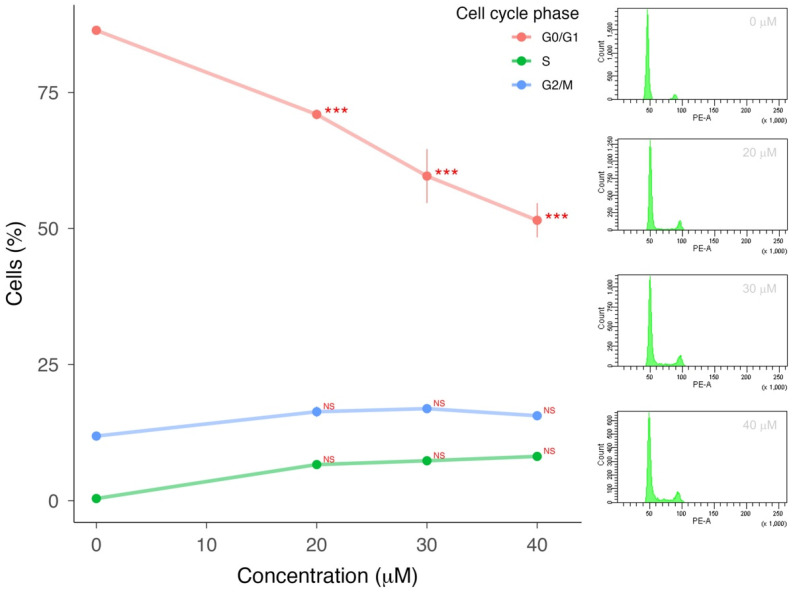
The impact of the *Z* isomer of the (**17j**) compound on the cell cycle progression of BJ fibroblasts was assessed by flow cytometry. The cell numbers in each cell cycle phase, G0/G1, S, and G2/M, were measured, as indicated by exemplary histograms. The values of cell number ratios in each of the cell cycle phases for each treatment were compared to the 0 µM concentration (the cells treated with 0.5% DMSO) with the two-way ANOVA with Tukey’s post hoc test. The significance of *p*-value was marked as follows: 0–0.001 (***), 0.001–0.01 (**), 0.01–0.05 (*), 0.05–0.1 (.), and >0.1 (not significant, NS).

The *Z* conformer of **17j** alters cell cycle progression in BJ fibroblasts in a manner similar to its effect on PC-3 cells, with some differences. A significant decrease was observed, from 86.4 to 51.5%, of cells undergoing the G0/G1 phase (Figure 9). On the other hand, the number of cells in the S or G2/M phase increased insignificantly, from 0.4 to 8% and 11.9 to 15.6%, respectively.

The effect of the *Z* conformer of the **17j** compound on cell cycle progression revealed a distinct differential response between cancerous PC-3 cells and non-cancerous BJ fibroblasts. While **17j** induced a profound S and G2/M phase arrest in PC-3 cells, its effect on BJ fibroblasts was limited to a significant decrease in the G0/G1 population. This pattern of strong cell cycle disruption in PC-3 cells aligns with the effects of other established anti-proliferative agents. For instance, the Cdc25 phosphatase inhibitor DA 3003-2 was shown to induce a clear G2/M accumulation in PC-3 cells [69], and the bioflavonoid luteolin was found to inhibit PC-3 cell growth by downregulating key genes governing the cell cycle. The more subdued response in BJ fibroblasts is likely rooted in fundamental biological differences. As non-transformed cells, fibroblasts are typically contact-inhibited and reside in a quiescent state (G0) with distinct chromatin organization compared to cycling cells, which may allow them to better withstand the assumed checkpoint-activating signal of **17j**. The decrease in G0/G1 could indicate that **17j** stimulates an initial, abortive entry into the cell cycle. However, the lack of subsequent accumulation in the S/G2/M phases suggests that these fibroblasts, with their intact cell cycle checkpoints, may undergo a stalled progression or utilize alternative pathways not leading to full arrest. In contrast, the PC-3 cancer cells, which lack functional p53 and other regulatory mechanisms, are more vulnerable to the checkpoint-activating signal of **17j**, leading to the observed buildup at the S and G2/M phases, a common response to agents that disrupt DNA replication or microtubule integrity [69]. Also, taking into account that the **17j** compound changes the ratio of PC-3 cells in the S phase, as compared to colchicine, this can imply an additional mechanism of action besides binding to microtubules.

Limitations of the study should be acknowledged. The biological evaluation was limited to a single cancer cell line and one non-cancerous cell line and relied primarily on cytotoxicity and cell cycle analyses. While molecular docking provided supportive insight into potential target interactions, it does not constitute direct evidence of a molecular mechanism. Therefore, the results presented should be interpreted as a proof-of-concept demonstration of cytotoxicity potential rather than definitive evidence of anticancer efficacy.

## 3. Materials and Methods

### 3.1. Synthesis

All commercially available compounds were purchased from Merck, Sigma-Aldrich, and used without further purification. Solvents were dried according to standard procedures.

(*R*)-N-deacetylcolchicine was obtained according to the known literature [70].

General procedure of synthesis of compounds (**15a**–**c**):

Fluoro derivative of aniline (9 mmol) was dissolved in dichloromethane (180 mL) and then an aqueous solution (180 mL) of oxone (31.5 mmol) was added. The reaction mixture was vigorously stirred at room temperature. After 4 h the mixture was separated, and the aqueous phase was extracted with DCM twice. The combined organic layers were dried over MgSO4 and concentrated in vacuum. The residue containing nitrosoarene was dissolved in glacial acetic acid (400 mL), and 3-aminoacetophenone (9 mmol) was then added to the mixture. The mixture was stirred for 24 h at room temperature. After 24 h the mixture was concentrated in vacuum and the crude product was purified by flash chromatography (DCM) to obtain (**15a**–**c**).

General procedure of synthesis of compounds (**17a**–**l**):

Compound **16a**–**d** (0.825 mmol) was dissolved in MeOH (0.826 mL). The resulting mixture was stirred for 15 min and then compound **15a**–**c** (0.825 mmol) was added to the mixture. A total of 6 M NaOH was added (0.413 mL) to the resulting mixture. The mixture was stirred for 2 h at room temperature. The mixture was then left for 16 h at −15 °C and the resulting precipitate was filtered. The resulting precipitate was crystallized from methanol to obtain (**17a**–**l**).

### 3.2. Molecular Docking Simulation

The optimal ground-state geometry for (**17b**), (**17d**), (**17f**), (**17h**), (**17j**), and (**17l**) *E* or *Z* compounds was calculated using the density functional theory (DFT). During calculation, the B3LYP functional and the 6-31G* basis set was employed and the polarizable continuum model (PCM; Gaussian 03W) [53,54]. All the calculations were performed on a server equipped with a 16-core Intel^®^ Xeon^®^ E7310 processor that operates at 1.60 GHz. The operating system was Open SUSE 10.3. DMSO was used as the solvent.

Molecular docking of isomers *E* and *Z* of compounds (**17b**), (**17d**), (**17f**), (**17h**), (**17j**), and (**17l**) into the 3D X-ray structure of tubulin (PDB code: 1SA0) [48] was carried out using the AutoDock Vina, version 1.2.5 software (the Broyden–Fletcher–Goldfarb–Shanno (BFGS) method) [52]. Configurations of protein/dimethoxydibenzo[*b,f*]oxepine complex were created using UCSF Chimera, version 1.18 software [71]. The graphical user interface AutoDockTools (ADT) was employed to set up the enzyme: all hydrogens were added. For macromolecules, generated pdbqt files were saved. The 3D structures of ligand molecules were built, optimized (B3LYP functional and 6-31* basis set level) for *E*/*Z* isomers, and saved in Mol2 format. The graphical user interface ADT was also employed to set up the ligand and the pdbqt file was saved. AutoDock Vina, version 1.2.5 software was employed for all docking calculations. The AutoDockTools program was used to generate the docking input files. During docking, a grid box of size 25 × 25 × 25 pointed in the x, y, and z directions was built, and the maps were center-located (39.82, 53.24, −8.21) in the catalytic site of the protein. A grid spacing of 0.375 Å (approximately one-fourth of the length of a carbon–carbon covalent bond) was used for the calculation of the energetic map.

All computations were performed on an Intel^®^CoreTM i7-4702MQ 3.2 GHz processor running Ubuntu 18.04 Work-station Linux distribution. PyMOL, version 2.1 software was used to analyze the docking results [72]. The Protein-Ligand Interaction Profiler (PLIP) was used to predict protein-docked ligand interactions [73].

### 3.3. Cell Proliferation Analysis

Human BJ fibroblasts (American Type Cell Culture Collection, Manassas, VA, USA, CRL-2522) and human prostate cancer cell line PC-3 (American Type Cell Culture Collection, Manassas, VA, USA, CRL-1435), derived from bone metastases, were routinely grown in Ham’s F-12K (Kaighn’s) Medium (ThermoFisher, Waltham, MA, USA, 21127-022) and supplemented with heat-inactivated 10% Fetal Bovine Serum (Biowest, France, S181B) and 1% antibiotic solution (Biowest, Nuaillé, France, L0022), in a humidified 5% CO_2_ atmosphere at 37 °C. Cells were used between 3 and 20 passages. The 96-well plates were prepared by seeding 5 × 10^3^ or 2.5 × 10^3^ cells per well, for 24 and 48 h treatments, respectively, with at least five technical and two biological repeats. After 24 h, the medium was changed to the FBS-free medium, with FBS substituted by Mg^2+^- and Ca^2+^-free Phosphate-Buffered Saline (PBS) (Biowest, Nuaillé, France, L0615), supplemented with selected concentrations of **17j** compound, ranging from 0.1 to 40 µM, or colchicine (Sigma-Aldrich, St. Louis, MO, USA, C9754) in the range of 1–200 nM (the control treatment medium contained 0.5% DMSO). The cells were treated with either the *Z* conformer of **17j**, with continuous illumination with 525 nm light, or the *E* conformer. The *Z* conformer was prepared by illuminating its DMSO solution with 525 nm light overnight**.** The cell proliferation was analyzed with CellTiter Aqueous One Solution Cell Proliferation MTS (3-(4,5-dimethylthiazol-2-yl)-5-(3-carboxymethoxyphenyl)-2-(4-sulfophenyl)-2H-tetrazolium) Assay (Promega, Madison, WI, USA, G5430) after 24 or 48 h of treatment. The absorbance was measured at 490 and 670 nm with FLUOStar Omega plate reader (BMG LABTECH GmbH, Ortenberg, Germany).

### 3.4. Cell Cycle Analysis

The BJ or PC-3 cells were treated as described previously, with the exception that 0.15 × 10^6^ cells per well were seeded in 6-well plates. After 24 h of treatment, cells were harvested by trypsinization (Biowest, Nuaillé, France, L0931), washed twice, resuspended in PBS, and added to cold 70% ethanol for overnight fixation at −20 °C. Subsequently, cells were centrifuged and resuspended in PBS containing 5 µg/mL of propidium iodide in PBS (Sigma-Aldrich, St. Louis, MO, USA, 4170), 0.1% Triton X-100, and 10 µg/mL of DNAse-free RNAse (A&A Biotechnology, Gdansk, Poland, 1006-10) in the dark for 4 h at 37 °C. FACS analysis was performed using a FACSCanto II flow cytometer and FACSDiva, version 9.0 software (BD Biosciences, San Jose, CA, USA). Flow rate was set to about 1000 events/cells per second. Signals from at least 10,000 events per sample were captured. The percentages of PC-3 cells in cell cycle phases (G0/G1, S, and G2/M) were determined using ModFit LT 4.1.7 (Verity Software House, Topsham, ME, USA).

### 3.5. Statistical Analysis

The statistical analysis was performed within R 4.5.1 project [74]. In the case of MTS cell proliferation assay, before being subjected to a dose response model, the OD_490_ values were normalized by (1) subtracting the OD_670_, (2) subtracting the OD_490_ mean value of negative control (MTS incubated with the growth medium), and (3) dividing by the positive control mean values of OD_490_ (cells grown in the presence of 0.5% DMSO). The log-logistic four parametric model (4PL), with the lower limit boundary set to 0 and group fitting, was used, as available in the drc package [75], to fit cell proliferation data. The residuals were checked for normality with QQ and density plots and considered as sufficiently normally distributed. The assessment metrics for model goodness of fit were performed with functions available in the drc package and with custom ones. When appropriate, the delta method was applied to obtain error of central tendency. To assess the goodness of fit for each of the biological replicates, deviance R^2^ (DR^2^) was calculated and assessed. Additionally, the Cook’s distance, Mean Absolute Scaled Error (MASE), and the Neill’s goodness of fit test metrics were applied [76]. The DR^2^ was calculated according to the following Equation: 1 − (D_res_/D_null_), where D_res_—residual deviance and D_null_—null deviance (deviance of the model with mean as a predicted value). The null hypothesis was assessed with the two-way ANOVA with Tukey’s post hoc test for multiple comparisons or with the two-way *t*-test [77] when only two groups were compared. The differences in the cell cycle percentages were assessed with the two-way ANOVA with Tukey’s post hoc test [78]. All the graphing was prepared with ggplot2 3.5.1 [79].

## 4. Conclusions

In this study, we designed and synthesized a series of previously unreported photoswitchable chalcone–fluoroazobenzene hybrids (**17a**–**17l**) and evaluated their photochemical and preliminary biological properties. Selected compounds exhibited visible-light-induced photoisomerization, enabling reversible *E*/*Z* switching under biologically compatible irradiation conditions, in contrast to classical photopharmaceutical systems that rely on ultraviolet light. Molecular docking provided supportive insight into potential interactions with the colchicine binding site of tubulin, and initial biological screening identified compound **17j** as exhibiting time-dependent anti-proliferative activity in PC-3 cells. These results should be interpreted as proof-of-concept evidence for the photopharmacological potential of this scaffold rather than definitive confirmation of anticancer efficacy or a fully established molecular mechanism. Overall, these findings open new avenues for the development of active-molecule switches, expand the repertoire of visible-light-responsive photoswitches, and lay the groundwork for further optimization and mechanistic validation of chalcone-based photopharmacological agents.

## Figures and Tables

**Table 1:** Selected *UV−Vis* spectroscopic data of compounds (**17b**), (**17d**), (**17f**), (**17h**), (**17j**), and (**17l**).

Compound *	λ_max_n→π* (*E*) ** [nm]	*ε_max_* (*n*→π*) (dm^3^·mol^−1^·cm^−1^)	λ_max_*n*→π* (*Z*) ** [nm]	*ε_max_* (*n*→π*) (dm^3^·mol^−1^·cm^−1^)	Δ λ _max_*n*→π* [nm]
**17b**	442.1	688	434.0	898	8.1
**17d**	440.8	660	434.1	850	6.7
**17f**	441.9	872	427.9	1150	14.0
**17h**	441.2	796	426.9	1016	14.3
**17j**	448.3	922	417.4	1130	30.9
**17l**	448.6	902	417.9	1074	30.7

* For compounds (**17a**, **17c**), (**17e**, **17g**), and (**17i**, **17k**), no separation of *n*→π* absorption bands is observed in the visible range of the spectrum between ***E*** and ***Z*** stereoisomers. ** λmax *n*→π* represents the wavelength at the maximal absorption of the *n*→π* transition bands. *ε*—Molar absorption coefficient; nm—nanometers.

## Data Availability

Data are contained within the article and Supplementary Materials.

## Supplementary Materials

The following supporting information can be downloaded at https://www.mdpi.com/article/10.3390/molecules31020362/s1, [80,81,82].
