# Supplementary material for: Synthesis and Study of Substituted Chalcones Combined with Fluoroazobenzenes—New Photoswitches for Application in Biological Systems
Source: Molecules. 2026 Jan 20;31(2):362. doi: 10.3390/molecules31020362 (PMC12844124; doi:10.3390/molecules31020362)
Supplement: Supplementary file 1 [file molecules-31-00362-s001.zip › molecules-4079134-supplementary.pdf]

Synthesis and study of substituted chalcones combined with fluoroazobenzenes—new photoswitches for application in biological systems

Piotr Tobiasz[a*], Damian Mielecki[b], Anna Stachurska-Skrodzka[c], Jakub Miętus[a], Filip Borys[a] and Hanna Krawczyk[a]

[a]Department of Organic Chemistry, Faculty of Chemistry, Warsaw University of Technology, Noakowskiego 3, 00-664 Warsaw, Poland.

[b]Department of Neurochemistry, Mossakowski Medical Research Institute, Pawińskiego 5, 02-106 Warsaw, Poland.

[c]Department of Cell Biology and Immunology, Center of Postgraduate Education, Marymoncka 99/103, 01-813 Warsaw, Poland.

* Correspondence: piotr.tobiasz.dokt@pw.edu.pl

Supplementary Materials

Table of Contents:

1. **Computational aspects** 3
   - **1.1 Calculated geometry** 3
   - **1.2 Molecular docking** 24
2. **Experimental section** 45
   - **2.1 General information** 45
   - **2.2 General procedure of synthesis** 45
3. **NMR spectra of obtained compounds** 48
   - **3.1 General information** 48
   - **3.2 Copies of ^{1}H NMR and ^{13}C NMR spectra of (17a-l)** 49

4. Photochemical studies 96
4.1 Illumination setup for light-dependent assays 96
4.2 Photostationary state (PSS) analysis 98
4.3 Photoisomerization kinetics 109
4.4 UV-ViS spectra 119
5. References 143

1. Computational aspects

1.1 Calculated geometry

The optimal ground-state geometry for [(**17b**), (**17d**), (**17f**), (**17h**), (**17j**), and (**17l**)] ***E*** and ***Z*** isomers was calculated using the density functional theory (DFT). In calculation, the B3LYP functional and the 6-31G* basis set was employed and the continuum model (PCM; Gaussian 03W) [1,2] was used to simulate the effects of the solvent. All the calculations were performed on a server equipped with a 16 quad-core XEON (R) CPU E7310 processor operating at 1.60 GHz. The operating system was Open SUSE 10.3. DMSO was used as the solvent. We performed vibrational analysis for the optimized geometry of all investigated ***E*** and ***Z*** isomers and obtained no negative frequencies in the potential energy surface for the calculated molecules.

- The calculated coordinates of (**17b*E***) (part of the calculated log file) -

```
     Item               Value     Threshold  Converged?
 Maximum Force            0.000021     0.000450     YES
 RMS     Force            0.000005     0.000300     YES
 Maximum Displacement     0.001697     0.001800     YES
 RMS     Displacement     0.000425     0.001200     YES
 Optimization completed.
                          Standard orientation:
 ---------------------------------------------------------------------
 Center     Atomic      Atomic             Coordinates (Angstroms)
 Number     Number       Type             X           Y           Z
 ---------------------------------------------------------------------
    1          6           0       -4.100358   -0.132688   -0.121511
    2          6           0       -5.125150   -1.082671   -0.172117
    3          6           0       -6.452090   -0.672882   -0.360032
    4          6           0       -6.760886    0.681778   -0.498466
    5          6           0       -5.731073    1.635344   -0.446956
    6          6           0       -4.407012    1.238379   -0.258500
    7          6           0       -2.693870   -0.497337    0.077889
    8          6           0       -2.177492   -1.742921    0.152360
    9          6           0       -0.740465   -2.070387    0.244959
   10          6           0        0.249900   -1.088083    0.803528
   11          8           0       -0.346867   -3.167591   -0.155250
```

12	6	0	-0.054497	-0.208837	1.852926
13	6	0	0.931743	0.637020	2.374949
14	6	0	2.219049	0.627237	1.853015
15	6	0	2.536632	-0.256745	0.804335
16	6	0	1.557611	-1.116142	0.300469
17	7	0	3.814341	-0.365063	0.194855
18	7	0	4.681145	0.433868	0.644297
19	6	0	8.219894	1.216536	0.015639
20	6	0	6.926177	1.222344	0.530800
21	6	0	5.958484	0.330972	0.043873
22	6	0	6.296613	-0.580269	-0.974543
23	6	0	7.583434	-0.594634	-1.494828
24	6	0	8.523239	0.305530	-0.989923
25	9	0	9.771736	0.288065	-1.498746
26	8	0	-7.376133	-1.673942	-0.392612
27	6	0	-8.745174	-1.326987	-0.574879
28	8	0	-6.137889	2.927923	-0.592121
29	6	0	-5.153136	3.955751	-0.549128
30	1	0	-4.927337	-2.143426	-0.064540
31	1	0	-7.774772	1.033623	-0.644365
32	1	0	-3.604762	1.965726	-0.218672
33	1	0	-2.012053	0.348016	0.145325
34	1	0	-2.806663	-2.619926	0.018651
35	1	0	-1.049945	-0.202846	2.284881
36	1	0	0.686051	1.304246	3.196578
37	1	0	2.990556	1.283261	2.240800
38	1	0	1.818238	-1.807660	-0.493939
39	1	0	8.983358	1.897664	0.377319
40	1	0	6.643277	1.916037	1.316763
41	1	0	5.540110	-1.264665	-1.341895
42	1	0	7.873075	-1.285927	-2.280240
43	1	0	-9.294309	-2.269940	-0.567169
44	1	0	-9.108114	-0.687317	0.239345
45	1	0	-8.905841	-0.819772	-1.534437
46	1	0	-5.695664	4.892870	-0.684097
47	1	0	-4.633148	3.971976	0.416890

```
  48          1              0       -4.418411    3.840862   -1.355851
---------------------------------------------------------------------
After PCM corrections, the SCF energy is -1322.83496186     a.u.
```

Visualization of the calculated geometry of (**17b*E***):

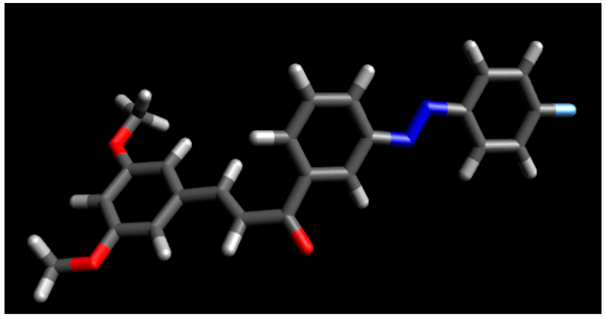

- The calculated coordinates of (**17b*Z***) (part of the calculated log file) -

```
      Item               Value     Threshold  Converged?
Maximum Force            0.000232     0.000450     YES
RMS     Force            0.000054     0.000300     YES
Maximum Displacement     0.000150     0.001800     YES
RMS     Displacement     0.000040     0.001200     YES
Optimization completed.
                      Standard orientation:
---------------------------------------------------------------------
Center     Atomic     Atomic              Coordinates (Angstroms)
Number     Number      Type             X           Y           Z
---------------------------------------------------------------------
   1          6              0       -3.763928   -0.042820   -0.054189
   2          6              0       -4.195989    1.285938   -0.065199
   3          6              0       -5.549990    1.594773    0.144859
   4          6              0       -6.469951    0.574030    0.369861
   5          6              0       -6.031805   -0.764715    0.385218
   6          6              0       -4.692637   -1.082322    0.175915
   7          6              0       -2.339163   -0.296437   -0.283997
   8          6              0       -1.730797   -1.496770   -0.400855
   9          6              0       -0.309176   -1.704177   -0.739765
  10          6              0        0.716167   -0.632890   -0.490950
  11          8              0        0.041168   -2.769365   -1.250511
  12          6              0        0.679425    0.213052    0.628084
  13          6              0        1.689808    1.156811    0.826978
  14          6              0        2.749959    1.259389   -0.069658
  15          6              0        2.802044    0.400548   -1.180024
  16          6              0        1.779642   -0.525118   -1.396252
```

17	7	0	3.772391	0.561973	-2.228445
18	7	0	5.005851	0.644044	-2.032675
19	6	0	6.125153	-1.008251	1.149264
20	6	0	5.356223	-0.741386	0.019949
21	6	0	5.640153	0.382189	-0.773204
22	6	0	6.722366	1.212315	-0.445536
23	6	0	7.470296	0.975220	0.704219
24	6	0	7.158155	-0.136239	1.480790
25	9	0	7.891305	-0.386792	2.585102
26	8	0	-5.862417	2.919843	0.109636
27	6	0	-7.220351	3.298705	0.311574
28	8	0	-7.016154	-1.677895	0.618019
29	6	0	-6.666204	-3.057683	0.650426
30	1	0	-3.497300	2.098768	-0.239097
31	1	0	-7.522537	0.766210	0.538469
32	1	0	-4.358975	-2.111446	0.193988
33	1	0	-1.733780	0.600577	-0.395784
34	1	0	-2.299781	-2.423099	-0.369818
35	1	0	-0.125668	0.125695	1.350170
36	1	0	1.651890	1.814652	1.690722
37	1	0	3.525703	2.003624	0.079718
38	1	0	1.810771	-1.171163	-2.267994
39	1	0	5.935441	-1.878983	1.768951
40	1	0	4.553862	-1.417634	-0.253774
41	1	0	6.960096	2.049633	-1.095110
42	1	0	8.296601	1.619054	0.988084
43	1	0	-7.239754	4.387198	0.237343
44	1	0	-7.874116	2.868796	-0.457526
45	1	0	-7.577444	2.994261	1.303346
46	1	0	-7.595721	-3.595686	0.843640
47	1	0	-6.247033	-3.386919	-0.308587
48	1	0	-5.948863	-3.270169	1.452991

After PCM corrections, the SCF energy is -1322.81300099 a.u.

Visualization of the calculated geometry of (**17b*Z***):

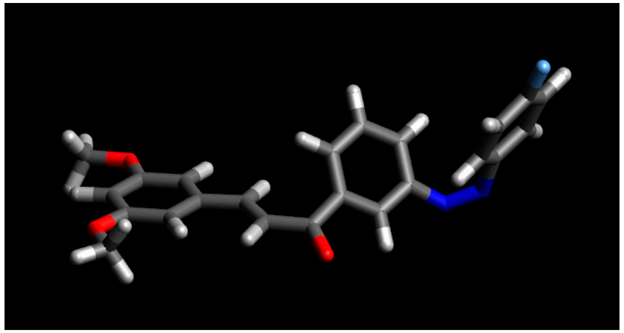

- The calculated coordinates of (**17d*E***) (part of the calculated log file) -

```
      Item               Value     Threshold  Converged?
Maximum Force            0.000003     0.000450     YES
RMS     Force            0.000001     0.000300     YES
Maximum Displacement     0.010295     0.001800     NO
RMS     Displacement     0.002762     0.001200     NO
Optimization completed on the basis of negligible forces.

                      Standard orientation:
---------------------------------------------------------------------
Center     Atomic     Atomic              Coordinates (Angstroms)
Number     Number      Type              X           Y           Z
---------------------------------------------------------------------
   1          6           0        2.857558    0.740707    0.017696
   2          6           0        2.857173   -0.300516    0.964010
   3          6           0        3.896607   -1.225312    1.028808
   4          6           0        4.955025   -1.110956    0.130284
   5          6           0        4.987175   -0.092411   -0.824995
   6          6           0        3.943210    0.823895   -0.876095
   7          6           0        1.732159    1.677329    0.005313
   8         35           0        6.389426   -2.371622    0.201940
   9          6           0        1.600358    2.779632   -0.763357
  10          6           0        0.501339    3.763459   -0.670494
  11          6           0       -0.843204    3.384845   -0.119146
  12          8           0        0.701568    4.920536   -1.043354
  13          6           0       -1.588296    4.373756    0.541689
  14          6           0       -2.863015    4.089548    1.034849
  15          6           0       -3.425035    2.829248    0.851866
  16          6           0       -2.700408    1.839356    0.167013
  17          6           0       -1.413082    2.119739   -0.308597
  18          7           0       -3.172520    0.528868   -0.105453
  19          7           0       -4.321532    0.277266    0.350622
  20          6           0       -4.799270   -1.027335    0.082365
  21          6           0       -6.076387   -1.316128    0.586009
  22          6           0       -6.651911   -2.568277    0.386106
```

23	6	0	-5.927406	-3.519274	-0.323642
24	6	0	-4.655124	-3.262916	-0.837534
25	6	0	-4.090533	-2.011462	-0.632624
26	9	0	-6.471567	-4.736246	-0.525438
27	1	0	2.029442	-0.386450	1.663828
28	1	0	3.882467	-2.021492	1.765508
29	1	0	5.817529	-0.019529	-1.519345
30	1	0	3.976140	1.608627	-1.625582
31	1	0	0.944891	1.448734	0.720907
32	1	0	2.391368	3.091191	-1.441786
33	1	0	-1.153631	5.360744	0.663593
34	1	0	-3.422858	4.859316	1.559079
35	1	0	-4.417557	2.594628	1.220692
36	1	0	-0.889344	1.340307	-0.851681
37	1	0	-6.604636	-0.542085	1.134435
38	1	0	-7.638281	-2.812522	0.766909
39	1	0	-4.132645	-4.041372	-1.384982
40	1	0	-3.104466	-1.775896	-1.017016

```
---------------------------------------------------------------------
After PCM corrections, the SCF energy is  -3664.89219229     a.u.
```

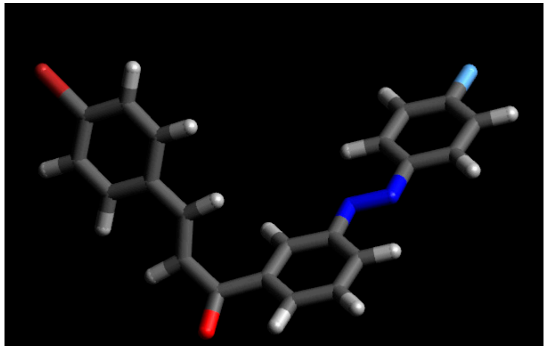

Visualization of the calculated geometry of (**17d*E***):

- The calculated coordinates of (**17d*Z***) (part of the calculated log file) -

```
        Item               Value     Threshold  Converged?
 Maximum Force            0.000403     0.000450     YES
 RMS     Force            0.000090     0.000300     YES
 Maximum Displacement     0.000403     0.001800     YES
 RMS     Displacement     0.000100     0.001200     YES
 Optimization completed.

                         Standard orientation:
 ---------------------------------------------------------------------
 Center     Atomic      Atomic              Coordinates (Angstroms)
```

Number	Number	Type	X	Y	Z
1	6	0	3.436676	0.299617	0.044013
2	6	0	3.953662	-0.628082	-0.878920
3	6	0	5.307693	-0.953309	-0.902989
4	6	0	6.161903	-0.343067	0.012761
5	6	0	5.683521	0.580810	0.944906
6	6	0	4.329839	0.896153	0.955613
7	6	0	2.002257	0.592268	0.013792
8	35	0	8.022269	-0.778977	-0.002385
9	6	0	1.342296	1.502543	0.761702
10	6	0	-0.086623	1.853624	0.627320
11	6	0	-1.081942	0.885416	0.053655
12	8	0	-0.463883	2.970409	0.985291
13	6	0	-0.992842	-0.503445	0.234730
14	6	0	-1.981589	-1.342543	-0.284586
15	6	0	-3.072075	-0.813770	-0.969449
16	6	0	-3.178716	0.577605	-1.132834
17	6	0	-2.176194	1.416062	-0.642161
18	7	0	-4.184841	1.180283	-1.962983
19	7	0	-5.408675	0.937675	-1.867024
20	6	0	-5.998893	0.187807	-0.797023
21	6	0	-5.705671	0.407127	0.559159
22	6	0	-6.430844	-0.257076	1.544384
23	6	0	-7.428324	-1.148469	1.159110
24	6	0	-7.750782	-1.370397	-0.175931
25	6	0	-7.049299	-0.670634	-1.153447
26	9	0	-8.119736	-1.803005	2.114279
27	1	0	3.284091	-1.102841	-1.592005
28	1	0	5.691304	-1.669792	-1.621488
29	1	0	6.361964	1.045702	1.652390
30	1	0	3.968621	1.612495	1.687115
31	1	0	1.438358	0.017349	-0.718056
32	1	0	1.872723	2.152171	1.454334
33	1	0	-0.166618	-0.927899	0.795133
34	1	0	-1.902860	-2.417550	-0.149332

35	1	0	-3.830498	-1.470522	-1.383322
36	1	0	-2.246715	2.488548	-0.792897
37	1	0	-4.931588	1.110173	0.844967
38	1	0	-6.231959	-0.093779	2.598871
39	1	0	-8.548973	-2.059605	-0.432242
40	1	0	-7.298281	-0.791618	-2.203621

After PCM corrections, the SCF energy is -3664.86988032 a.u.

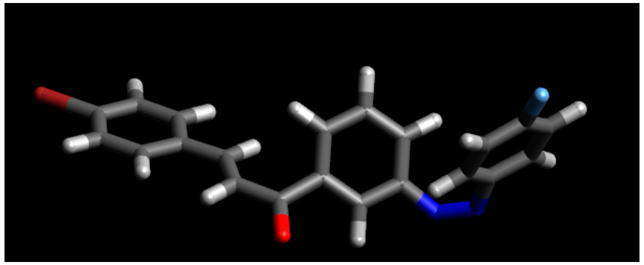

Visualization of the calculated geometry of (**17d*Z***):

- The calculated coordinates of (**17f*E***) (part of the calculated log file) -

Item	Value	Threshold	Converged?
Maximum Force	0.000066	0.000450	YES
RMS Force	0.000013	0.000300	YES
Maximum Displacement	0.000557	0.001800	YES
RMS Displacement	0.000140	0.001200	YES

Optimization completed.

Standard orientation:

Center Number	Atomic Number	Atomic Type	Coordinates (Angstroms) X	Y	Z
1	6	0	4.324909	-0.026238	-0.125002
2	6	0	4.592481	1.329810	-0.329975
3	6	0	5.910000	1.771211	-0.535001
4	6	0	6.959176	0.855792	-0.532114
5	6	0	6.687941	-0.510413	-0.321892
6	6	0	5.385775	-0.959281	-0.119401
7	6	0	2.928489	-0.419297	0.082498
8	6	0	2.443082	-1.671770	0.224018
9	6	0	1.013433	-2.028295	0.320717
10	6	0	-0.006415	-1.038793	0.810188

11	8	0	0.649402	-3.156036	-0.017657
12	6	0	0.263579	-0.093298	1.810137
13	6	0	-0.748413	0.757963	2.271080
14	6	0	-2.028201	0.687640	1.736118
15	6	0	-2.310988	-0.263092	0.736705
16	6	0	-1.306307	-1.126733	0.294127
17	7	0	-3.577021	-0.435665	0.120058
18	7	0	-4.468258	0.362612	0.522634
19	6	0	-7.310600	-0.853439	-1.614495
20	6	0	-6.037181	-0.766785	-1.070952
21	6	0	-5.727683	0.193176	-0.088312
22	6	0	-6.752034	1.062095	0.326033
23	6	0	-8.036375	1.000699	-0.198540
24	6	0	-8.288566	0.035843	-1.165637
25	9	0	-9.525614	-0.040991	-1.687585
26	9	0	-6.491470	1.988545	1.260978
27	8	0	6.057356	3.111613	-0.726499
28	6	0	7.369622	3.622699	-0.939010
29	8	0	7.789336	-1.312993	-0.335674
30	6	0	7.610898	-2.710591	-0.130505
31	1	0	3.790843	2.062150	-0.336739
32	1	0	7.989792	1.151786	-0.684715
33	1	0	5.182269	-2.008906	0.046278
34	1	0	2.224876	0.410421	0.093363
35	1	0	3.093224	-2.540284	0.147632
36	1	0	1.252854	-0.038742	2.252680
37	1	0	-0.528184	1.477248	3.055073
38	1	0	-2.819857	1.346258	2.075749
39	1	0	-1.540923	-1.868399	-0.462205
40	1	0	-7.559875	-1.588984	-2.372200
41	1	0	-5.249189	-1.438693	-1.392176
42	1	0	-8.809640	1.682449	0.136870
43	1	0	7.252443	4.700073	-1.066335
44	1	0	8.019904	3.425730	-0.077536
45	1	0	7.823701	3.195941	-1.842076
46	1	0	8.608788	-3.148861	-0.184218

47	1	0	7.174543	-2.917993	0.854609
48	1	0	6.976667	-3.151907	-0.909502

After PCM corrections, the SCF energy is -1422.06374310 a.u.

Visualization of the calculated geometry of (**17f*E***):

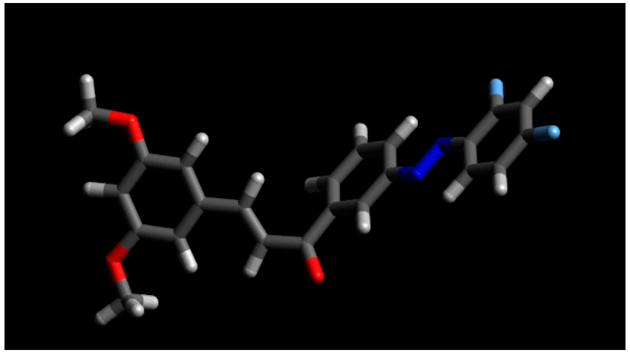

- The calculated coordinates of (**17f*Z***) (part of the calculated log file) -

Item	Value	Threshold	Converged?
Maximum Force	0.000323	0.000450	YES
RMS Force	0.000049	0.000300	YES
Maximum Displacement	0.001504	0.001800	YES
RMS Displacement	0.000374	0.001200	YES

Optimization completed.

Standard orientation:

Center Number	Atomic Number	Atomic Type	Coordinates (Angstroms) X	Y	Z
1	6	0	-4.042745	-0.024137	-0.063476
2	6	0	-4.457739	1.308102	0.011230
3	6	0	-5.808639	1.620067	0.236059
4	6	0	-6.743007	0.598965	0.388528
5	6	0	-6.322295	-0.743453	0.315622
6	6	0	-4.986274	-1.064163	0.092011
7	6	0	-2.619652	-0.281249	-0.300059
8	6	0	-2.025872	-1.481165	-0.479521
9	6	0	-0.607090	-1.692488	-0.826415
10	6	0	0.439540	-0.660959	-0.506876
11	8	0	-0.275032	-2.729898	-1.402527
12	6	0	0.403477	0.135709	0.647714
13	6	0	1.434246	1.042213	0.907516
14	6	0	2.515542	1.153653	0.037772
15	6	0	2.567492	0.341434	-1.107062

16	6	0	1.523834	-0.542637	-1.385810
17	7	0	3.567556	0.509429	-2.124305
18	7	0	4.796836	0.547332	-1.894484
19	6	0	5.828993	-1.324730	1.202734
20	6	0	5.074641	-0.953080	0.093869
21	6	0	5.380147	0.211194	-0.630083
22	6	0	6.488093	0.967303	-0.220226
23	6	0	7.241679	0.639958	0.896946
24	6	0	6.892834	-0.514562	1.589755
25	9	0	7.618619	-0.859729	2.668804
26	8	0	-6.103435	2.948607	0.289255
27	6	0	-7.456566	3.331200	0.515142
28	8	0	-7.319522	-1.657250	0.481828
29	6	0	-6.987821	-3.040503	0.419286
30	1	0	-3.747886	2.121534	-0.104685
31	1	0	-7.793769	0.793526	0.565116
32	1	0	-4.666735	-2.096615	0.042592
33	1	0	-2.003489	0.613533	-0.357163
34	1	0	-2.607873	-2.399529	-0.502214
35	1	0	-0.417692	0.039365	1.350112
36	1	0	1.394928	1.664030	1.797356
37	1	0	3.304945	1.872392	0.233289
38	1	0	1.554230	-1.148872	-2.285565
39	1	0	5.607907	-2.229058	1.759611
40	1	0	4.255154	-1.584188	-0.231605
41	9	0	6.808838	2.070765	-0.917375
42	1	0	8.075848	1.258534	1.208528
43	1	0	-7.460880	4.422360	0.517258
44	1	0	-8.114445	2.965337	-0.283063
45	1	0	-7.820138	2.963121	1.482711
46	1	0	-7.924816	-3.578162	0.573225
47	1	0	-6.570739	-3.308165	-0.559609
48	1	0	-6.275397	-3.317493	1.206399

After PCM corrections, the SCF energy is -1422.04163974 a.u.

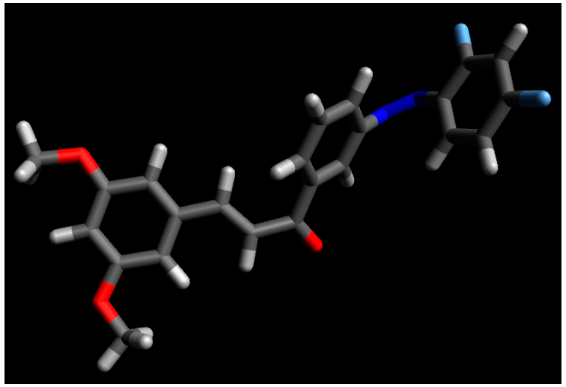

Visualization of the calculated geometry of (**17f*Z***):

- The calculated coordinates of (**17h*E***) (part of the calculated log file) -

```
    Item                     Value     Threshold  Converged?
Maximum Force            0.000030     0.000450     YES
RMS     Force            0.000009     0.000300     YES
Maximum Displacement     0.001259     0.001800     YES
RMS     Displacement     0.000311     0.001200     YES
Optimization completed.
```

Standard orientation:

Center Number	Atomic Number	Atomic Type	Coordinates (Angstroms) X	Y	Z
1	6	0	3.113107	0.736380	0.018910
2	6	0	3.093402	-0.302005	0.968123
3	6	0	4.124125	-1.235394	1.046936
4	6	0	5.193175	-1.132782	0.159600
5	6	0	5.244445	-0.117448	-0.798259
6	6	0	4.209030	0.807653	-0.863311
7	6	0	1.996058	1.682503	-0.008329
8	35	0	6.615506	-2.405785	0.250422
9	6	0	1.883842	2.786401	-0.777826
10	6	0	0.791135	3.778404	-0.698906
11	6	0	-0.564315	3.408440	-0.168128
12	8	0	1.004775	4.934748	-1.066134
13	6	0	-1.312747	4.401606	0.482436
14	6	0	-2.596820	4.125935	0.956391
15	6	0	-3.164781	2.869932	0.763949
16	6	0	-2.436093	1.876043	0.088742
17	6	0	-1.139836	2.147558	-0.367565
18	7	0	-2.912476	0.569992	-0.191477
19	7	0	-4.071090	0.328481	0.247700

20	6	0	-4.545433	-0.969369	-0.032229
21	6	0	-5.830105	-1.287890	0.441383
22	6	0	-6.412334	-2.531251	0.232736
23	6	0	-5.675360	-3.474130	-0.472687
24	6	0	-4.396083	-3.213222	-0.966693
25	6	0	-3.841741	-1.961591	-0.741737
26	9	0	-6.528614	-0.365800	1.121184
27	9	0	-6.220193	-4.684734	-0.687187
28	1	0	2.257458	-0.378848	1.659152
29	1	0	4.095239	-2.029220	1.785735
30	1	0	6.082764	-0.053820	-1.483890
31	1	0	4.256541	1.589750	-1.614772
32	1	0	1.197396	1.460563	0.696661
33	1	0	2.686453	3.092345	-1.445109
34	1	0	-0.873312	5.385516	0.611908
35	1	0	-3.158881	4.899141	1.473038
36	1	0	-4.164179	2.641362	1.117560
37	1	0	-0.613409	1.365043	-0.903486
38	1	0	-7.404835	-2.752989	0.608273
39	1	0	-3.862842	-3.984807	-1.512233
40	1	0	-2.851098	-1.718684	-1.109283

After PCM corrections, the SCF energy is -3764.12056548 a.u.

Visualization of the calculated geometry of (**17h*E***):

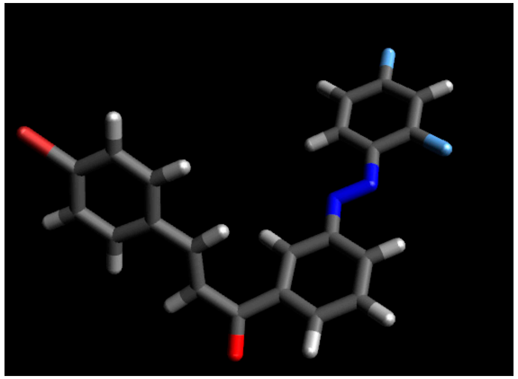

- The calculated coordinates of (**17h*Z***) (part of the calculated log file) -

```
     Item               Value     Threshold  Converged?
 Maximum Force            0.000157     0.000450     YES
 RMS     Force            0.000046     0.000300     YES
 Maximum Displacement     0.001024     0.001800     YES
 RMS     Displacement     0.000243     0.001200     YES
 Optimization completed.
```

Standard orientation:

Center Number	Atomic Number	Atomic Type	Coordinates (Angstroms) X	Y	Z
1	6	0	3.716784	0.282738	-0.104218
2	6	0	4.221418	-0.972715	-0.490291
3	6	0	5.574019	-1.281833	-0.368257
4	6	0	6.438855	-0.321302	0.151694
5	6	0	5.972455	0.934017	0.548749
6	6	0	4.620269	1.228177	0.419504
7	6	0	2.283967	0.541665	-0.257123
8	35	0	8.298117	-0.725042	0.328450
9	6	0	1.642084	1.707219	-0.026203
10	6	0	0.212800	1.970007	-0.292737
11	6	0	-0.797127	0.857130	-0.321200
12	8	0	-0.153974	3.126430	-0.505822
13	6	0	-0.710414	-0.277203	0.500787
14	6	0	-1.710324	-1.251636	0.459967
15	6	0	-2.812132	-1.101110	-0.377596
16	6	0	-2.915773	0.044555	-1.183605
17	6	0	-1.901993	1.004120	-1.169804
18	7	0	-3.941065	0.192610	-2.178505
19	7	0	-5.161643	0.041268	-1.948738
20	6	0	-5.712126	-0.074585	-0.631044
21	6	0	-5.414523	0.801395	0.425363
22	6	0	-6.140166	0.763779	1.612293
23	6	0	-7.165922	-0.168789	1.739038
24	6	0	-7.504281	-1.049365	0.716918
25	6	0	-6.780684	-0.966943	-0.463131
26	9	0	-7.090593	-1.798236	-1.473355
27	9	0	-7.863100	-0.221562	2.888553
28	1	0	3.543495	-1.719701	-0.896037
29	1	0	5.948157	-2.253896	-0.671515
30	1	0	6.658599	1.669407	0.955100
31	1	0	4.268438	2.205346	0.735952

```
  32          1             0          1.705393   -0.305059   -0.620807
  33          1             0          2.188498    2.600930    0.266466
  34          1             0          0.125403   -0.393302    1.182440
  35          1             0         -1.631845   -2.132675    1.090499
  36          1             0         -3.578217   -1.868659   -0.419763
  37          1             0         -1.972146    1.875180   -1.813332
  38          1             0         -4.625499    1.534745    0.302803
  39          1             0         -5.925373    1.445921    2.427936
  40          1             0         -8.308122   -1.768085    0.830391
 ---------------------------------------------------------------------
After PCM corrections, the SCF energy is  -3764.09853436     a.u.
```

Visualization of the calculated geometry of (**17h*Z***):

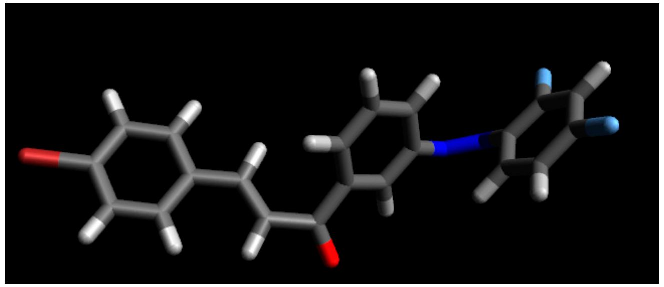

- The calculated coordinates of (**17j*E***) (part of the calculated log file) -

```
    Item                     Value     Threshold  Converged?
 Maximum Force              0.000103     0.000450     YES
 RMS     Force              0.000023     0.000300     YES
 Maximum Displacement       0.000308     0.001800     YES
 RMS     Displacement       0.000097     0.001200     YES
 Optimization completed.

                          Standard orientation:
 ---------------------------------------------------------------------
 Center     Atomic      Atomic             Coordinates (Angstroms)
 Number     Number       Type             X           Y           Z
 ---------------------------------------------------------------------
    1          6             0          4.168835   -0.019217   -0.090935
    2          6             0          4.441196    1.331001   -0.326114
    3          6             0          5.762113    1.765705   -0.523160
    4          6             0          6.809973    0.849722   -0.481709
    5          6             0          6.533849   -0.510335   -0.240398
    6          6             0          5.228369   -0.952738   -0.045552
    7          6             0          2.768961   -0.405682    0.105620
```

8	6	0	2.278441	-1.654940	0.257951
9	6	0	0.846979	-2.006648	0.344411
10	6	0	-0.175972	-1.004719	0.800929
11	8	0	0.484121	-3.140070	0.024195
12	6	0	0.081715	-0.047066	1.792609
13	6	0	-0.932787	0.817067	2.223041
14	6	0	-2.202888	0.747432	1.665244
15	6	0	-2.473286	-0.214901	0.673724
16	6	0	-1.466818	-1.091762	0.262406
17	7	0	-3.729130	-0.390514	0.032763
18	7	0	-4.609254	0.437013	0.403598
19	6	0	-8.093392	1.370069	-0.368635
20	6	0	-6.791815	1.344849	0.108034
21	6	0	-5.880250	0.306530	-0.180582
22	6	0	-6.379272	-0.741715	-0.987477
23	6	0	-7.679229	-0.749237	-1.473070
24	6	0	-8.532809	0.311544	-1.164165
25	9	0	-5.593677	-1.784316	-1.291098
26	9	0	-6.369455	2.362549	0.874988
27	8	0	5.913931	3.100836	-0.746384
28	6	0	7.229967	3.605584	-0.950633
29	8	0	7.634409	-1.313992	-0.217236
30	6	0	7.451262	-2.705970	0.019729
31	1	0	3.640830	2.063744	-0.363445
32	1	0	7.843057	1.140725	-0.626596
33	1	0	5.021341	-1.997266	0.145796
34	1	0	2.067764	0.426034	0.097604
35	1	0	2.927217	-2.525886	0.200601
36	1	0	1.063345	0.007592	2.251891
37	1	0	-0.722187	1.545547	3.001170
38	1	0	-2.996349	1.415931	1.980413
39	1	0	-1.693677	-1.842283	-0.487408
40	1	0	-8.737377	2.204725	-0.113526
41	1	0	-8.004324	-1.585427	-2.083303
42	1	0	-9.549134	0.310937	-1.545925
43	1	0	7.116224	4.679659	-1.105925

44	1	0	7.866428	3.429081	-0.074514
45	1	0	7.697706	3.156705	-1.835830
46	1	0	8.449233	-3.146446	-0.008606
47	1	0	7.000407	-2.888812	1.003194
48	1	0	6.827846	-3.165776	-0.757316

After PCM corrections, the SCF energy is -1422.05666789 a.u.

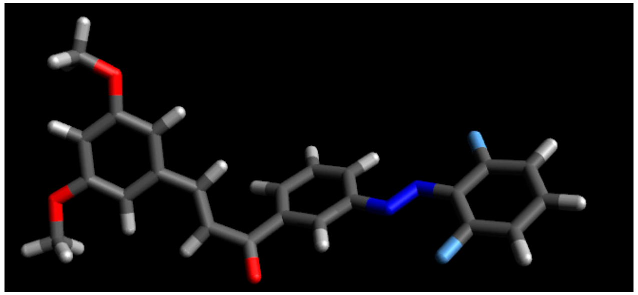

Visualization of the calculated geometry of (**17j*E***):

- The calculated coordinates of (**17j*Z***) (part of the calculated log file) -

Item	Value	Threshold	Converged?
Maximum Force	0.000113	0.000450	YES
RMS Force	0.000009	0.000300	YES
Maximum Displacement	0.000063	0.001800	YES
RMS Displacement	0.000014	0.001200	YES

Optimization completed.

Standard orientation:

Center Number	Atomic Number	Atomic Type	Coordinates (Angstroms) X	Y	Z
1	6	0	3.492298	-0.146379	0.231375
2	6	0	3.770450	1.162755	0.633114
3	6	0	5.009685	1.750853	0.331299
4	6	0	5.968583	1.029712	-0.375282
5	6	0	5.685097	-0.288702	-0.782541
6	6	0	4.461127	-0.882347	-0.486891
7	6	0	2.180616	-0.699486	0.578483
8	6	0	1.739349	-1.956735	0.357037
9	6	0	0.449497	-2.504873	0.819464
10	6	0	-0.720730	-1.609054	1.119375

11	8	0	0.330680	-3.721374	0.974585
12	6	0	-1.601611	-1.994480	2.143632
13	6	0	-2.717176	-1.217286	2.444000
14	6	0	-3.009052	-0.079782	1.689682
15	6	0	-2.158907	0.283594	0.636959
16	6	0	-0.995812	-0.453410	0.381558
17	7	0	-2.325207	1.494086	-0.106973
18	7	0	-3.413663	1.857285	-0.604772
19	6	0	-5.753038	-0.949576	-1.537717
20	6	0	-4.579600	-0.250870	-1.291375
21	6	0	-4.568716	1.008256	-0.674405
22	6	0	-5.818070	1.558217	-0.355732
23	6	0	-7.011114	0.884217	-0.565751
24	6	0	-6.970262	-0.377088	-1.165084
25	9	0	-5.835211	2.777245	0.216397
26	9	0	-3.404901	-0.783124	-1.681994
27	8	0	5.176253	3.028589	0.772140
28	6	0	6.411406	3.681891	0.496392
29	8	0	6.693534	-0.895698	-1.469453
30	6	0	6.495250	-2.229587	-1.926281
31	1	0	3.038481	1.744827	1.184900
32	1	0	6.936132	1.443769	-0.631417
33	1	0	4.245918	-1.893512	-0.805918
34	1	0	1.520838	-0.003959	1.092843
35	1	0	2.384760	-2.710231	-0.088237
36	1	0	-1.385487	-2.896069	2.707521
37	1	0	-3.370113	-1.497132	3.265842
38	1	0	-3.872101	0.531588	1.931914
39	1	0	-0.344027	-0.134992	-0.425038
40	1	0	-5.699092	-1.919849	-2.020318
41	1	0	-7.945212	1.349289	-0.268889
42	1	0	-7.894808	-0.916214	-1.346067
43	1	0	6.325694	4.677609	0.934407
44	1	0	7.255310	3.152549	0.956187
45	1	0	6.583967	3.771914	-0.583394
46	1	0	7.416628	-2.507728	-2.440543

47	1	0	6.323093	-2.918153	-1.089595
48	1	0	5.653769	-2.291561	-2.627653

After PCM corrections, the SCF energy is -1422.04005349 a.u.

Visualization of the calculated geometry of (**17j*Z***):

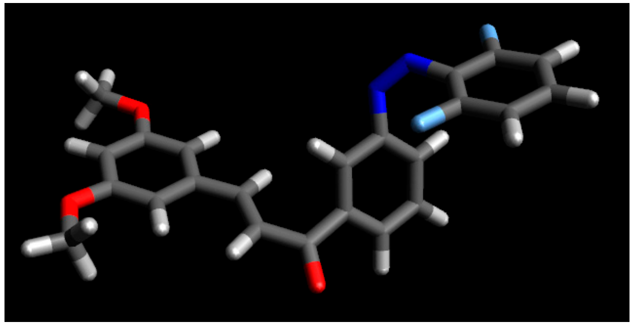

- The calculated coordinates of (**17l*E***) (part of the calculated log file) -

Item	Value	Threshold	Converged?
Maximum Force	0.000011	0.000450	YES
RMS Force	0.000002	0.000300	YES
Maximum Displacement	0.001035	0.001800	YES
RMS Displacement	0.000302	0.001200	YES

Optimization completed.

Standard orientation:

Center Number	Atomic Number	Atomic Type	Coordinates (Angstroms) X	Y	Z
1	6	0	2.963353	0.817013	0.046139
2	6	0	3.008033	-0.244395	0.968683
3	6	0	4.102347	-1.103349	1.037430
4	6	0	5.171118	-0.901260	0.167051
5	6	0	5.159739	0.139877	-0.764102
6	6	0	4.061221	0.989591	-0.819514
7	6	0	1.781764	1.681617	0.028168
8	35	0	6.681143	-2.069655	0.243405
9	6	0	1.598350	2.790493	-0.720297
10	6	0	0.439735	3.703547	-0.632752
11	6	0	-0.890695	3.233137	-0.119134
12	8	0	0.576559	4.878141	-0.978529
13	6	0	-1.709522	4.161798	0.541976
14	6	0	-2.975762	3.792338	0.999949

15	6	0	-3.455893	2.504315	0.782003
16	6	0	-2.656449	1.573861	0.096706
17	6	0	-1.378322	1.939933	-0.344320
18	7	0	-3.037589	0.240632	-0.210359
19	7	0	-4.185696	-0.072251	0.215353
20	6	0	-4.618622	-1.382151	-0.047352
21	6	0	-5.944958	-1.672085	0.340119
22	6	0	-6.534505	-2.915217	0.169720
23	6	0	-5.780270	-3.937376	-0.406957
24	6	0	-4.460806	-3.711637	-0.803461
25	6	0	-3.900041	-2.454983	-0.624296
26	9	0	-6.671461	-0.689381	0.895857
27	9	0	-2.627861	-2.279809	-1.007251
28	1	0	2.172026	-0.398771	1.646552
29	1	0	4.122189	-1.916082	1.755718
30	1	0	5.999117	0.281530	-1.436627
31	1	0	4.061595	1.792414	-1.550408
32	1	0	0.995513	1.388511	0.721033
33	1	0	2.383236	3.164320	-1.373851
34	1	0	-1.338467	5.170687	0.691788
35	1	0	-3.592499	4.517270	1.524203
36	1	0	-4.439937	2.202203	1.123227
37	1	0	-0.797838	1.202947	-0.888679
38	1	0	-7.560920	-3.065185	0.486647
39	1	0	-6.222397	-4.918838	-0.548297
40	1	0	-3.856098	-4.494569	-1.249226

After PCM corrections, the SCF energy is -3764.11357298 a.u.

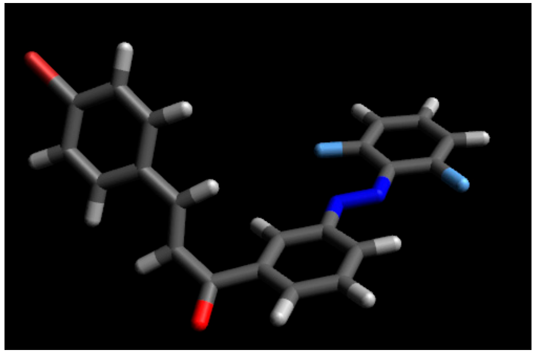

Visualization of the calculated geometry of (**17l*E***):

- The calculated coordinates of (**17l*Z***) (part of the calculated log file) -

Item	Value	Threshold	Converged?
Maximum Force	0.000089	0.000450	YES

RMS	Force	0.000010	0.000300	YES
Maximum	Displacement	0.000237	0.001800	YES
RMS	Displacement	0.000070	0.001200	YES

Optimization completed.

Standard orientation:

Center Number	Atomic Number	Atomic Type	Coordinates (Angstroms) X	Y	Z
1	6	0	-3.172855	0.606392	-0.140647
2	6	0	-3.563908	-0.462199	-0.968175
3	6	0	-4.834390	-1.025597	-0.877564
4	6	0	-5.731063	-0.514814	0.058261
5	6	0	-5.376596	0.543711	0.897951
6	6	0	-4.105457	1.096349	0.794652
7	6	0	-1.820844	1.148152	-0.287703
8	35	0	-7.477391	-1.276789	0.201158
9	6	0	-1.295994	2.211032	0.359232
10	6	0	0.036162	2.795927	0.103968
11	6	0	1.148506	1.980126	-0.492999
12	8	0	0.234810	3.980656	0.375494
13	6	0	2.074998	2.633516	-1.322818
14	6	0	3.141389	1.931545	-1.878321
15	6	0	3.339099	0.585466	-1.566935
16	6	0	2.444480	-0.059072	-0.702395
17	6	0	1.328612	0.623748	-0.203073
18	7	0	2.514987	-1.460955	-0.426211
19	7	0	3.567006	-2.047140	-0.088555
20	6	0	4.772121	-1.354379	0.269168
21	6	0	5.989863	-1.838929	-0.227668
22	6	0	7.218929	-1.361369	0.200079
23	6	0	7.246485	-0.389568	1.203418
24	6	0	6.061054	0.095490	1.758132
25	6	0	4.850654	-0.393935	1.287716

26	9	0	5.940134	-2.778349	-1.191353
27	9	0	3.704650	0.044614	1.844658
28	1	0	-2.861169	-0.858779	-1.696923
29	1	0	-5.120841	-1.848900	-1.523217
30	1	0	-6.085891	0.927940	1.623327
31	1	0	-3.840165	1.915986	1.455438
32	1	0	-1.204331	0.634087	-1.022473
33	1	0	-1.893442	2.801832	1.049840
34	1	0	1.931997	3.687484	-1.537743
35	1	0	3.829010	2.431754	-2.554306
36	1	0	4.163403	0.036012	-2.009654
37	1	0	0.640815	0.092341	0.446152
38	1	0	8.127220	-1.753560	-0.245037
39	1	0	8.199674	-0.010085	1.557831
40	1	0	6.059488	0.836988	2.550225

After PCM corrections, the SCF energy is -3764.09698526 a.u.

Visualization of the calculated geometry of (**17l*Z***) :

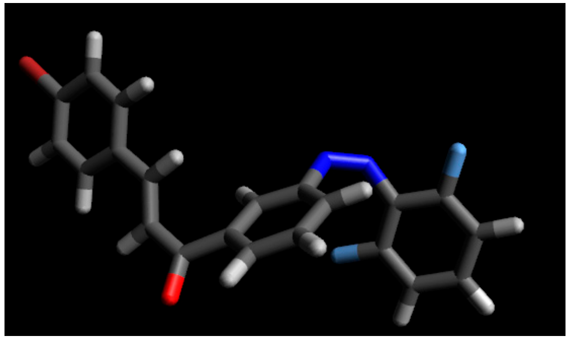

1.2 Molecular docking

The molecular docking of the compounds of the [**(17b)**, **(17d)**, **(17f)**, **(17h)**, **(17j)**, and **(17l)**] ***E*** and ***Z*** isomers into the 3D X-ray structure of tubulin (PDB code: 1SA0) [3] was conducted using the Auto-Dock Vina software (the Broyden–Fletcher–Goldfarb–Shanno (BFGS) method) [4]. The microtubule structure (PDB-ID:1SA0) was prepared using the PyMOL graphical user interface and Python scripts provided by AutodockTools. In the tubulin structure, chains C, D, and E were removed as well as small molecules (magnesium ion, GDT, GTP, and DAMA–colchicine). All hydrogens were added. Next, Python script *prepare_receptor4.py* was used for pdbqt file generation. The configurations of the protein/hybrid chalcones combined with fluoroazobenzene complex were created using UCSF Chimera software [5]. The graphical user interface, ADT, was employed to set up the enzyme, and all the hydrogens were added. For macromolecules, the generated pdbqt files were saved. The 3D structures of ligand molecules were built, optimized (a B3LYP functional and 6–31* basis set level) for the [**(17b)**, **(17d)**, **(17f)**, **(17h)**, **(17j)**, and **(17l)**] ***E*** and ***Z*** isomers, and saved in Mol2 format. The graphical user interface, ADT, was also employed to set up the ligand, and the pdbqt file was saved. The Auto-Dock Vina software was employed for all docking calculations. The AutoDockTools program was used to generate the docking input files. During docking, a grid box of size 25 x 25 x 25 pointed in the x, y, and z directions was built and the maps were center-located (39.82, 53.24, -8.21) in the catalytic site of the protein. A grid spacing of 0.375 Å (approximately one-fourth of the length of a carbon–carbon covalent bond) was used for the calculation of the energetic map. The.pdb files for investigated molecule [**(17b)**, **(17d)**, **(17f)**, **(17h)**, **(17j)**, and **(17l)**] ***E*** and ***Z*** isomers obtained from DFT calculations were converted to the pdbqt format with a script from AutoDockTool.

Compound	Affinity (kcal /mol)	Connections (date from RCSB Protein Data Bank—RCSB PDB)
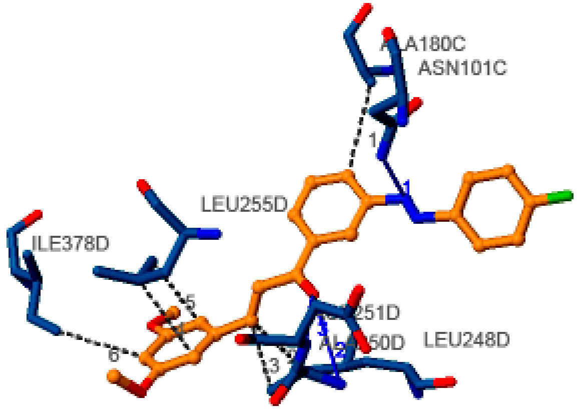 **17b*E***	-8.9	Hydrophobic Interactions; Hydrogen Bonds (see tables below)

⌄ Hydrophobic Interactions ····

Index	Residue	AA	Distance	Ligand Atom	Protein Atom
1	180C	ALA	3.58	6465	1284
2	248D	LEU	3.65	6470	5081
3	250D	ALA	3.41	6470	5095
4	255D	LEU	3.59	6476	5137
5	255D	LEU	3.47	6472	5139
6	378D	ILE	3.86	6474	5962

⌄ Hydrogen Bonds —

Index	Residue	AA	Distance H-A	Distance D-A	Donor Angle	Protein donor?	Side chain	Donor Atom	Acceptor Atom
1	101C	Amino acid type	[illegible]	3.27	160.57	✔	✔	674 [Nam]	6460 [Npl]
2	250D	ALA	3.01	3.46	109.44	✔	✖	5091 [Nam]	6485 [O2]
3	251D	ASP	3.22	3.91	128.44	✔	✖	5096 [Nam]	6485 [O2]

<table>
<tr>
<td>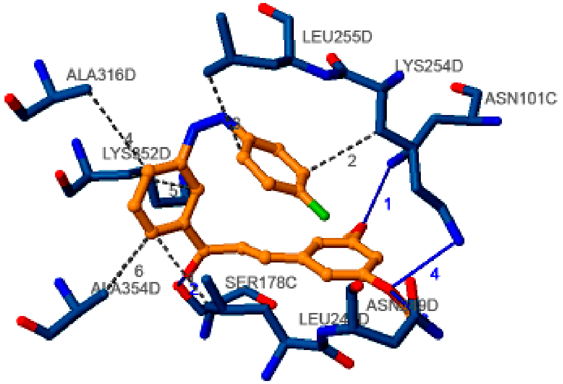

17b*Z*</td>
<td>-8.9</td>
<td>

Hydrophobic Interactions

Index	Residue	AA	Distance	Ligand Atom	Protein Atom
1	248D	LEU	3.87	6463	5082
2	254D	LYS	3.47	6483	5127
3	255D	LEU	3.66	6480	5139
4	316D	ALA	3.87	6465	5555
5	352D	LYS	3.64	6467	5836
6	354D	ALA	3.72	6463	5852

Hydrogen Bonds

Index	Residue	AA	Distance H-A	Distance D-A	Donor Angle	Protein donor?	Side chain	Donor Atom	Acceptor Atom
1	101C	ASN	Distance between hydrogen and acceptor atoms	[illegible]	[illegible]	[illegible]	✔	674 [Nam]	6486 [O3]
2	178C	SER	2.13	2.93	138.01	✔	✔	1272 [O3]	6485 [O2]
3	249D	ASN	3.26	3.60	102.74	✔	✔	5089 [Nam]	6487 [O3]
4	254D	LYS	2.39	3.24	140.00	✔	✔	5131 [N3+]	6487 [O3]

</td>
</tr>
</table>

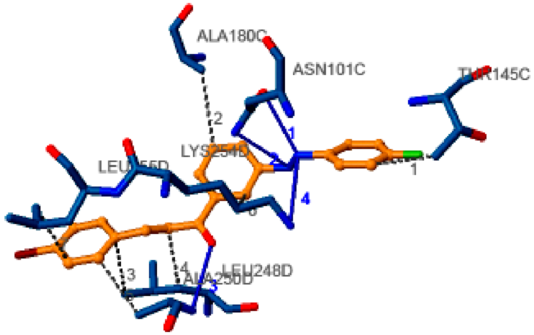

17d*E*

-9.4

Hydrophobic Interactions

Index	Residue	AA	Distance	Ligand Atom	Protein Atom
1	145C	THR	3.72	6481	1016
2	180C	ALA	3.79	6464	1284
3	248D	LEU	3.67	6471	5081
4	248D	LEU	3.34	6469	5079
5	250D	ALA	3.50	6472	5095
6	254D	LYS	3.93	6467	5129
7	255D	LEU	3.54	6473	5139

Hydrogen Bonds

Index	Residue	AA	Distance H-A	Distance D-A	Donor Angle	Protein donor?	Side chain	Donor Atom	Acceptor Atom
1	101C	ASN	2.71	3.51	138.55	✖	✔	6461 [Npl]	675 [O2]
2	101C	ASN	3.33	4.00	126.79	✔	✔	674 [Nam]	6460 [Npl]
3	250D	ALA	2.50	3.39	149.34	✔	✖	5091 [Nam]	6483 [O2]
4	254D	LYS	3.69	4.07	104.40	✔	✔	5131 [N3+]	6461 [Npl]

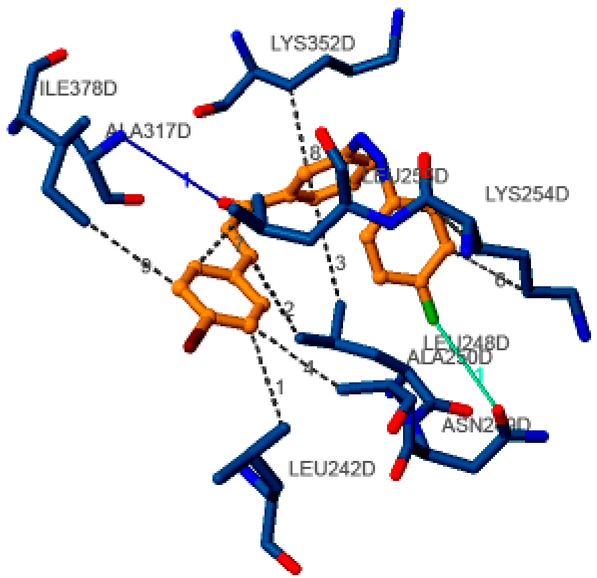

17d*Z*

-9.8

⌄ Hydrophobic Interactions ····

Index	Residue	AA	Distance	Ligand Atom	Protein Atom
1	242D	LEU	3.85	6473	5032
2	248D	LEU	3.80	6470	5081
3	248D	LEU	3.76	6463	5082
4	250D	ALA	3.61	6473	5095
5	254D	LYS	3.85	6478	5127
6	254D	LYS	3.74	6479	5129
7	255D	LEU	3.50	6476	5139
8	352D	LYS	3.86	6463	5836
9	378D	ILE	3.65	6475	5962

⌄ Hydrogen Bonds —

Index	Residue	AA	Distance H-A	Distance D-A	Donor Angle	Protein donor?	Side chain	Donor Atom	Acceptor Atom
1	317D	ALA	3.57	4.06	112.90	✔	✖	5556 [Nam]	6483 [O2]

⌄ Halogen Bonds —

Index	Residue	AA	Distance	Donor Angle	Acceptor Angle	Donor Atom	Acceptor Atom
1	249D	ASN	3.65	146.31	118.85	6485 [F]	5090 [O2]

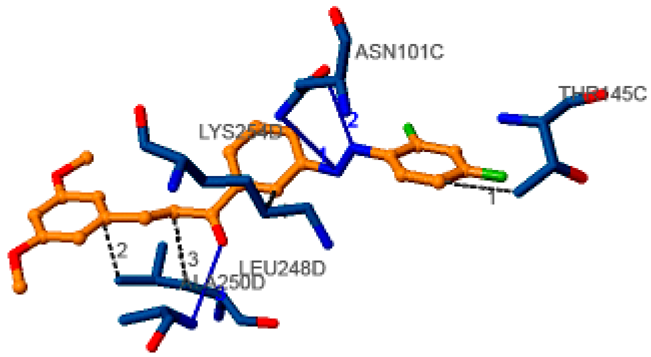

17f*E*

-9.4

Hydrophobic Interactions

Index	Residue	AA	Distance	Ligand Atom	Protein Atom
1	145C	THR	3.80	6481	1016
2	248D	LEU	3.60	6472	5081
3	248D	LEU	3.50	6469	5079
4	254D	LYS	3.64	6467	5129

Hydrogen Bonds

Index	Residue	AA	Distance H-A	Distance D-A	Donor Angle	Protein donor?	Side chain	Donor Atom	Acceptor Atom
1	101C	ASN	3.18	3.81	122.71	✔	✔	674 [Nam]	6460 [Npl]
2	101C	ASN	2.45	3.38	159.37	✖	✔	6461 [Npl]	675 [O2]
3	250D	ALA	2.42	3.26	143.33	✔	✖	5091 [Nam]	6485 [O2]

-9.0

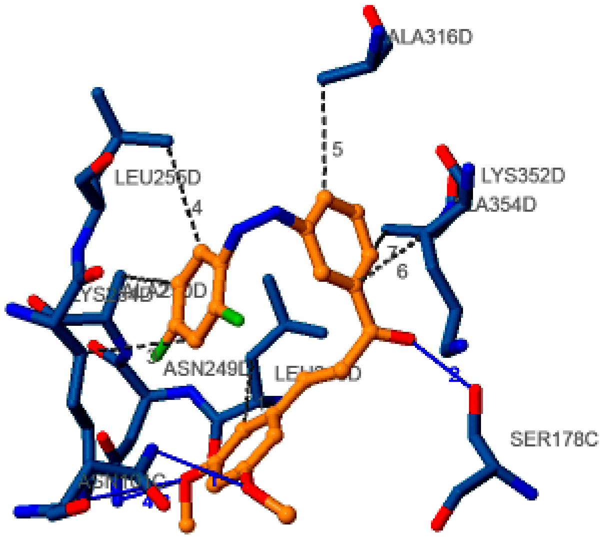

17fZ

Hydrophobic Interactions ····

Index	Residue	AA	Distance	Ligand Atom	Protein Atom
1	248D	LEU	3.92	6476	5079
2	250D	ALA	3.87	6483	5095
3	254D	LYS	3.52	6481	5127
4	255D	LEU	3.64	6484	5139
5	316D	ALA	3.82	6465	5555
6	352D	LYS	3.57	6462	5836
7	354D	ALA	3.69	6463	5852

Hydrogen Bonds —

Index	Residue	AA	Distance H-A	Distance D-A	Donor Angle	Protein donor?	Side chain	Donor Atom	Acceptor Atom
1	[illegible]	[illegible]	[illegible]	[illegible]	155.07	✔	✔	674 [Nam]	6486 [O3]
2	178C	SER	1.99	2.81	140.78	✔	✔	1272 [O3]	6485 [O2]
3	249D	ASN	3.46	3.77	100.58	✔	✔	5089 [Nam]	6487 [O3]
4	254D	LYS	2.52	3.37	139.88	✔	✔	5131 [N3+]	6487 [O3]

Number of receptor residue in PDB file

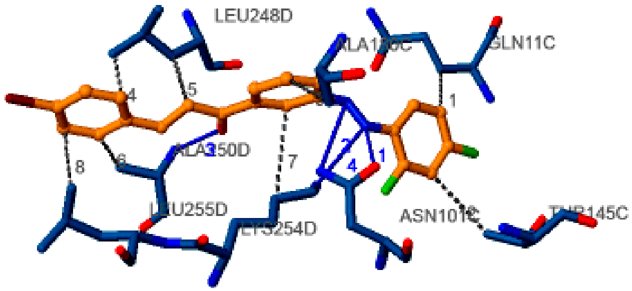 **17h*E***	-9.4	Hydrophobic Interactions; Hydrogen Bonds (tables below)

Hydrophobic Interactions ····

Index	Residue	AA	Distance	Ligand Atom	Protein Atom
1	11C	GLN	3.41	6479	74
2	145C	THR	3.77	6481	1016
3	180C	ALA	3.86	6464	1284
4	248D	LEU	3.72	6471	5081
5	248D	LEU	3.60	6469	5079
6	250D	ALA	3.50	6476	5095
7	254D	LYS	3.77	6467	5129
8	255D	LEU	3.63	6475	5139

Hydrogen Bonds —

Index	Residue	AA	Distance H-A	Distance D-A	Donor Angle	Protein donor?	Side chain	Donor Atom	Acceptor Atom
1	101C	ASN	2.71	3.62	153.89	✖	✔	6461 [Npl]	675 [O2]
2	101C	ASN	3.31	3.98	126.89	✔	✔	674 [Nam]	6460 [Npl]
3	250D	ALA	2.39	3.21	141.15	✔	✖	5091 [Nam]	6483 [O2]
4	254D	LYS	3.62	3.97	102.26	✔	✔	5131 [N3+]	6461 [Npl]

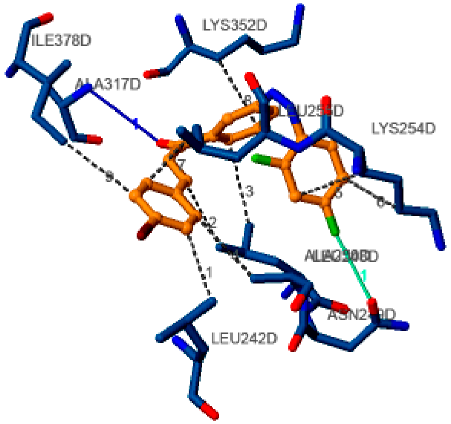

17h*Z*

-10.0

⌄ Hydrophobic Interactions ····

Index	Residue	AA	Distance	Ligand Atom	Protein Atom
1	242D	LEU	3.76	6475	5032
2	248D	LEU	3.76	6470	5081
3	248D	LEU	3.90	6463	5082
4	250D	ALA	3.54	6476	5095
5	254D	LYS	3.87	6479	5127
6	254D	LYS	3.63	6481	5129
7	255D	LEU	3.46	6472	5139
8	352D	LYS	3.65	6464	5836
9	378D	ILE	3.67	6473	5962

⌄ Hydrogen Bonds —

Index	Residue	AA	Distance H-A	Distance D-A	Donor Angle	Protein donor?	Side chain	Donor Atom	Acceptor Atom
1	317D	ALA	3.50	3.95	110.26	✔	✖	5556 [Nam]	6483 [O2]

⌄ Halogen Bonds —

Index	Residue	AA	Distance	Donor Angle	Acceptor Angle	Donor Atom	Acceptor Atom
1	249D	ASN	3.35	162.59	124.77	6486 [F]	5090 [O2]

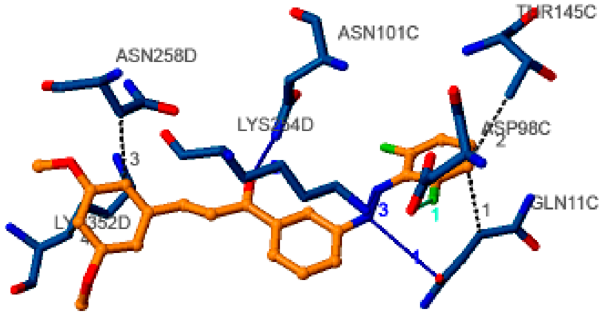

17j*E*

-9.2

⌄ Hydrophobic Interactions ····

Index	Residue	AA	Distance	Ligand Atom	Protein Atom
1	11C	GLN	3.82	6481	74
2	145C	THR	3.72	6481	1016
3	258D	ASN	3.86	6472	5156
4	352D	LYS	3.60	6474	5836

⌄ Hydrogen Bonds —

Index	Residue	AA	Distance H-A	Distance D-A	Donor Angle	Protein donor?	Side chain	Donor Atom	Acceptor Atom
1	11C	GLN	3.15	3.86	130.97	✖	✔	6460 [Npl]	78 [O2]
2	101C	ASN	2.24	3.17	157.84	✔	✔	674 [Nam]	6485 [O2]
3	254D	LYS	2.73	3.34	118.96	✔	✔	5131 [N3+]	6461 [Npl]

⌄ Halogen Bonds —

Index	Residue	AA	Distance	Donor Angle	Acceptor Angle	Donor Atom	Acceptor Atom
1	98C	ASP	3.88	149.55	100.76	6488 [F]	657 [O-]

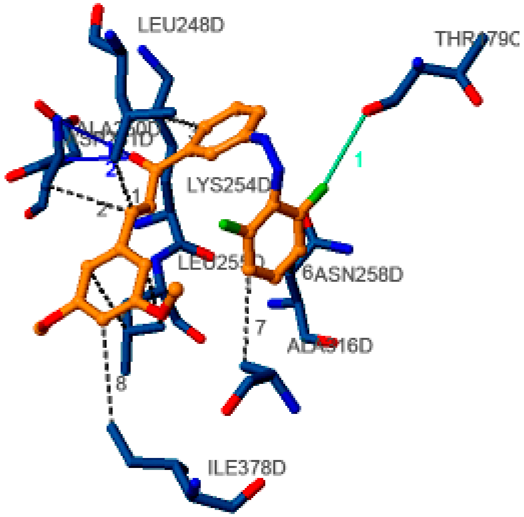

17j*Z*

-9.1

⌄ Hydrophobic Interactions ····

Index	Residue	AA	Distance	Ligand Atom	Protein Atom
1	248D	LEU	3.45	6470	5081
2	250D	ALA	3.47	6470	5095
3	254D	LYS	3.62	6463	5129
4	255D	LEU	3.65	6472	5137
5	255D	LEU	3.63	6476	5139
6	258D	ASN	3.68	6483	5156
7	316D	ALA	3.93	6481	5555
8	378D	ILE	3.90	6474	5962

⌄ Hydrogen Bonds —

Index	Residue	AA	Distance H-A	Distance D-A	Donor Angle	Protein donor?	Side chain	Donor Atom	Acceptor Atom
1	250D	ALA	2.80	3.19	104.75	✔	✖	5091 [Nam]	6485 [O2]
2	251D	ASP	2.66	3.38	129.95	✔	✖	5096 [Nam]	6485 [O2]

⌄ Halogen Bonds —

Index	Residue	AA	Distance	Donor Angle	Acceptor Angle	Donor Atom	Acceptor Atom
1	179C	THR	3.25	158.40	132.65	6489 [F]	1276 [O2]

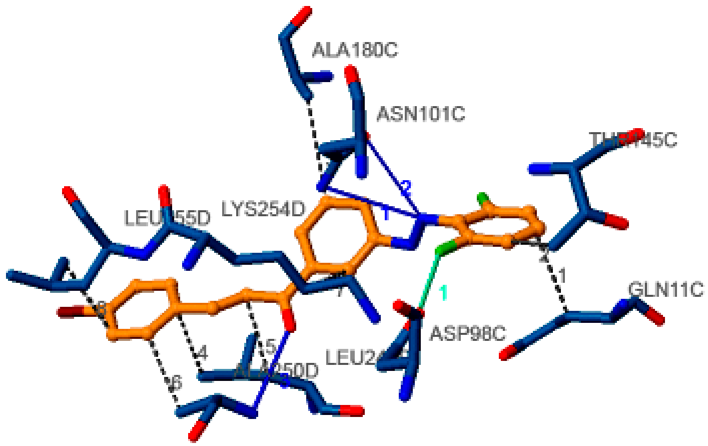

17l*E*

-9.5

Hydrophobic Interactions

Index	Residue	AA	Distance	Ligand Atom	Protein Atom
1	11C	GLN	3.96	6481	74
2	145C	THR	3.74	6479	1016
3	180C	ALA	3.89	6464	1284
4	248D	LEU	3.55	6471	5081
5	248D	LEU	3.52	6469	5079
6	250D	ALA	3.45	6476	5095
7	254D	LYS	3.81	6467	5129
8	255D	LEU	3.55	6475	5139

Hydrogen Bonds

Index	Residue	AA	Distance H-A	Distance D-A	Donor Angle	Protein donor?	Side chain	Donor Atom	Acceptor Atom
1	101C	ASN	3.33	3.76	108.69	✔	✔	674 [Nam]	6461 [Npl]
2	101C	ASN	2.69	3.66	168.24	✖	✔	6461 [Npl]	675 [O2]
3	250D	ALA	2.43	3.30	148.07	✔	✖	5091 [Nam]	6483 [O2]

Halogen Bonds

Index	Residue	AA	Distance	Donor Angle	Acceptor Angle	Donor Atom	Acceptor Atom
1	98C	ASP	3.55	144.45	126.51	6485 [F]	656 [O3]

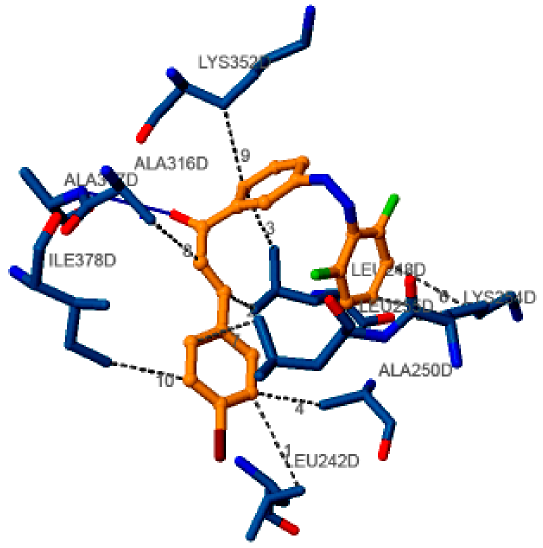

17l*Z*

-10.0

Hydrophobic Interactions

Index	Residue	AA	Distance	Ligand Atom	Protein Atom
1	242D	LEU	3.94	6473	5032
2	248D	LEU	3.86	6470	5081
3	248D	LEU	3.78	6463	5082
4	250D	ALA	3.60	6473	5095
5	254D	LYS	3.89	6480	5127
6	254D	LYS	3.88	6481	5129
7	255D	LEU	3.47	6476	5139
8	316D	ALA	3.94	6469	5555
9	352D	LYS	3.77	6463	5836
10	378D	ILE	3.64	6475	5962

Hydrogen Bonds

Index	Residue	AA	Distance H-A	Distance D-A	Donor Angle	Protein donor?	Side chain	Donor Atom	Acceptor Atom
1	317D	ALA	3.50	3.98	112.59	✔	✖	5556 [Nam]	6483 [O2]

17b*E*

```
Detected 4 CPUs
Reading input ... done.
Setting up the scoring function ... done.
Analyzing the binding site ... done.
Using random seed: 514935147
Performing search ... done.
Refining results ... done.

mode |   affinity | dist from best mode
     | (kcal/mol) | rmsd l.b.| rmsd u.b.
-----+------------+----------+----------
```

```
   1         -8.9      0.000      0.000
   2         -8.8      2.358      3.598
   3         -8.8      2.334      4.136
   4         -8.8      3.726      6.165
   5         -8.8      3.672      6.495
   6         -8.7      4.957     10.581
   7         -8.7      5.793     11.447
   8         -8.6      5.659     11.155
   9         -8.5      4.909     10.847
Writing output ... done.
```

17b*Z*

```
Detected 4 CPUs
Reading input ... done.
Setting up the scoring function ... done.
Analyzing the binding site ... done.
Using random seed: -2028784352
Performing search ... done.
Refining results ... done.

mode |   affinity | dist from best mode
     | (kcal/mol) | rmsd l.b.| rmsd u.b.
-----+------------+----------+----------
   1         -8.9      0.000      0.000
   2         -8.6      4.015      7.946
   3         -8.5      0.765      2.196
   4         -8.4      3.958      7.664
   5         -8.4      4.404      8.710
   6         -8.4      2.153      3.549
   7         -8.4      3.143      6.741
   8         -8.3      4.381      8.421
   9         -8.2      2.306      3.354
Writing output ... done.
```

17d*E*

```
Detected 4 CPUs
Reading input ... done.
Setting up the scoring function ... done.
Analyzing the binding site ... done.
Using random seed: 591957346
Performing search ... done.
Refining results ... done.

mode |   affinity | dist from best mode
     | (kcal/mol) | rmsd l.b.| rmsd u.b.
-----+------------+----------+----------
   1         -9.4      0.000      0.000
   2         -9.1      1.564      2.256
   3         -8.8      5.922     10.488
   4         -8.6      2.525      2.986
   5         -8.6      6.202     10.954
   6         -8.2      6.152     10.819
   7         -8.2      5.928     10.277
   8         -8.2      6.067     11.179
   9         -8.0      5.823     10.637
Writing output ... done.
```

17d*Z*

```
Detected 4 CPUs
Reading input ... done.
Setting up the scoring function ... done.
Analyzing the binding site ... done.
Using random seed: 620320170
Performing search ... done.
Refining results ... done.
```

```
mode |   affinity | dist from best mode
     | (kcal/mol) | rmsd l.b.| rmsd u.b.
-----+------------+----------+----------
   1         -9.8      0.000      0.000
   2         -9.3      2.709      3.437
   3         -9.0      1.425      2.012
   4         -8.9      2.516      3.749
   5         -8.8      5.127      6.664
   6         -8.8      3.935      6.469
   7         -8.7      4.690      6.458
   8         -8.7      2.843      4.816
   9         -8.6      2.679      3.268
Writing output ... done.
```

17f*E*

```
Detected 4 CPUs
Reading input ... done.
Setting up the scoring function ... done.
Analyzing the binding site ... done.
Using random seed: -1947638508
Performing search ... done.
Refining results ... done.

mode |   affinity | dist from best mode
     | (kcal/mol) | rmsd l.b.| rmsd u.b.
-----+------------+----------+----------
   1         -9.4      0.000      0.000
   2         -9.1      1.605      2.955
   3         -9.0      5.959     11.247
   4         -9.0      4.883      7.417
   5         -8.8      4.736      7.556
   6         -8.8      4.993     10.671
   7         -8.8      2.356      3.409
   8         -8.6      1.795      2.127
```

```
   9         -8.6      4.830     10.787
Writing output ... done.
```

17f*Z*

```
Detected 4 CPUs
Reading input ... done.
Setting up the scoring function ... done.
Analyzing the binding site ... done.
Using random seed: 1522435308
Performing search ... done.
Refining results ... done.

mode |   affinity | dist from best mode
     | (kcal/mol) | rmsd l.b.| rmsd u.b.
-----+------------+----------+----------
   1         -9.0      0.000      0.000
   2         -9.0      3.807      7.575
   3         -8.9      3.821      7.751
   4         -8.9      2.583      4.183
   5         -8.8      0.190      2.158
   6         -8.8      2.423      4.593
   7         -8.7      2.983      6.617
   8         -8.5      2.499      4.644
   9         -8.4      2.975      6.319
Writing output ... done.
```

17h*E*

```
Detected 4 CPUs
Reading input ... done.
Setting up the scoring function ... done.
Analyzing the binding site ... done.
Using random seed: 1550927649
Performing search ... done.
```

```
Refining results ... done.

mode |   affinity | dist from best mode
     | (kcal/mol) | rmsd l.b.| rmsd u.b.
-----+------------+----------+----------
   1         -9.4      0.000      0.000
   2         -9.2      1.769      2.117
   3         -9.1      3.259      4.575
   4         -9.0      2.875      3.583
   5         -8.9      5.886     10.331
   6         -8.5      6.305      9.507
   7         -8.4      5.848     10.811
   8         -8.3      6.146     10.790
   9         -8.3      3.777      4.470
Writing output ... done.
```

17h*Z*

```
Detected 4 CPUs
Reading input ... done.
Setting up the scoring function ... done.
Analyzing the binding site ... done.
Using random seed: 1579300096
Performing search ... done.
Refining results ... done.

mode |   affinity | dist from best mode
     | (kcal/mol) | rmsd l.b.| rmsd u.b.
-----+------------+----------+----------
   1        -10.0      0.000      0.000
   2         -9.5      2.200      3.261
   3         -9.0      2.511      3.864
   4         -8.9      1.565      2.304
   5         -8.9      4.676      6.479
   6         -8.8      3.670      6.455
```

```
   7         -8.8      3.867      6.387
   8         -8.8      3.056      4.897
   9         -8.4      5.057      6.782
Writing output ... done.
```

17j*E*

```
Detected 4 CPUs
Reading input ... done.
Setting up the scoring function ... done.
Analyzing the binding site ... done.
Using random seed: 1607726623
Performing search ... done.
Refining results ... done.

mode |   affinity | dist from best mode
     | (kcal/mol) | rmsd l.b.| rmsd u.b.
-----+------------+----------+----------
   1         -9.2      0.000      0.000
   2         -9.2      0.564      2.632
   3         -9.2      0.459      1.559
   4         -8.9      2.628      3.630
   5         -8.9      2.623      3.439
   6         -8.9      4.363     10.449
   7         -8.9      5.416     10.821
   8         -8.9      3.611      5.253
   9         -8.9      5.531     10.682
Writing output ... done.
```

17j*Z*

```
Detected 4 CPUs
Reading input ... done.
Setting up the scoring function ... done.
Analyzing the binding site ... done.
```

```
Using random seed: -960131244
Performing search ... done.
Refining results ... done.

mode |   affinity | dist from best mode
     | (kcal/mol) | rmsd l.b.| rmsd u.b.
-----+------------+----------+----------
   1         -9.1      0.000      0.000
   2         -9.1      0.345      2.780
   3         -9.1      3.534      8.413
   4         -9.1      2.705      7.083
   5         -9.0      2.677      6.986
   6         -9.0      3.745      7.537
   7         -8.8      4.686      9.425
   8         -8.7      3.920      8.174
   9         -8.7      4.614      9.588
Writing output ... done.
```

17l*E*

```
Detected 4 CPUs
Reading input ... done.
Setting up the scoring function ... done.
Analyzing the binding site ... done.
Using random seed: -1789038755
Performing search ... done.
Refining results ... done.

mode |   affinity | dist from best mode
     | (kcal/mol) | rmsd l.b.| rmsd u.b.
-----+------------+----------+----------
   1         -9.5      0.000      0.000
   2         -9.4      1.106      1.985
   3         -9.1      2.678      3.554
   4         -9.1      1.754      2.376
```

```
   5         -9.0      3.677      5.107
   6         -9.0      3.459      5.009
   7         -9.0      5.487     10.184
   8         -8.9      5.573      9.988
   9         -8.9      1.912      2.448
Writing output ... done.
```

17lZ

```
Detected 4 CPUs
Reading input ... done.
Setting up the scoring function ... done.
Analyzing the binding site ... done.
Using random seed: 815527948
Performing search ... done.
Refining results ... done.

mode |   affinity | dist from best mode
     | (kcal/mol) | rmsd l.b.| rmsd u.b.
-----+------------+----------+----------
   1        -10.0      0.000      0.000
   2         -9.6      1.829      2.550
   3         -8.8      2.236      3.460
   4         -8.8      4.865      8.282
   5         -8.8      3.870      6.569
   6         -8.7      4.074      7.238
   7         -8.4      1.514      2.483
   8         -8.3      4.546      7.171
   9         -8.3      3.552      6.234
Writing output ... done.
```

----

2. Experimental Section

2.1 General information

Nuclear magnetic resonance (NMR) spectroscopy: All the spectra were recorded using a Varian VNMRS spectrometer operating at 11.7 T and a Varian Mercury VX 9.4 T magnetic field. Measurements were performed for ca. 1.0 M solutions of all the compounds in DMSO-d6 or $CDCl_3$. The residual signals of DMSO-d6 (2.54 ppm) and $CDCl_3$ (7.26 ppm) in ^{1}H NMR and of the DMSO-d6 signal (40.4 ppm) and of $CDCl_3$ (77.0 ppm) in ^{13}C NMR spectra were used as the chemical shift references. Spin multiplicities are described as s (singlet), d (doublet), t (triplet), q (quartet), m (multiplet), and dd (double doublet). Coupling constants are reported in Hertz. All the proton spectra were recorded using the standard spectrometer software and parameters set: acquisition time 3s and pulse angle 30º. The standard measurement parameter set for 13C NMR spectra was as follows: pulse width 7 μs (the 90º pulse width was 12.5 μs), acquisition time 1 s, and spectral width 200 ppm. A total of 1000 scans of 32 K data points were accumulated and once again after zero-filling to 64 K, and the FID signals were subjected to Fourier transformation after applying a 1 Hz line broadening. The ^{1}H-^{13}Cgs-HSQC and ^{1}H-^{13}Cgs-HMBC spectra were also recorded using the standard Varian software. The measurement parameters guaranteeing the increase in magnetization and the possibility of quantitatively integrating the signals were as follows for ^{19}F NMR: pulse width 1 μs (the 90º pulse width was 9 μs), acquisition time 2.3 s, and spectral width 241 ppm. A total of 32 scans of 524 K data points were accumulated.
Mass spectrometry (MS): Mass spectra were recorded on the spectrometer QTOF Premier firmy Waters and spectrometer LTQ Orbitrap Velos.
UV–VIS spectrometer: EnSpire® multimode plate reader (PerkinElmer, Turku, Finland) with the software EnSpire Workstation version 4.10.3005.1440 (PerkinElmer) in absorbance mode was used for UV–VIS spectroscopic measurements. All experiments were performed in at least triplicate.

All commercially available compounds were purchased from Merck, Sigma-Aldrich, and used without further purification. Solvents were dried according to standard procedures.

2.2 General procedure of synthesis

- Compounds (15a-c) (Scheme 1 in the text):
Fluoro derivative of aniline (9 mmoL) was dissolved in dichloromethane (180 mL) and then an aqueous solution (180 mL) of oxone® (31.5 mmol) was added. The reaction mixture was vigorously stirred at room temperature. After 4 h the mixture was separated and the aqueous phase was extracted with DCM twice. The combined organic layers were dried over MgSO4 and concentrated in vacuum. The residue containing nitrosoarene was dissolved in glacial acetic acid (400 mL), and 3-aminoacetophenone (9 mmoL) was then added to the mixture. The mixture was stirred for 24 hours at room temperature. After 24 hours the mixture was concentrated in vacuum and the crude product was purified by flask chromatography (DCM) to obtain (**15a-c**).

Characterization Date of (15a-c)

Product	Conditions	Yield [%]
**(15a)**	1) DCM, H_2O, oxone, rt, 4h<br>2) AcOH, rt, 24h	56
**(15b)**	1) DCM, H_2O, oxone, rt, 4h<br>2) AcOH, rt, 24h	54
**(15c)**	1) DCM, H_2O, oxone, rt, 4h<br>2) AcOH, rt, 24h	49

- Compounds (17a-l) (Scheme 1 in the text):
Compound **16a-d** (0.825 mmoL) was dissolved in MeOH (0.826 mL). The resulting mixture was stirred for 15 minutes and then compound **15a-c** (0.825 mmol) was added to the mixture. A total of 6M NaOH was added (0.413 mL) to the resulting mixture. The mixture was stirred for 2 hours at room temperature. The mixture was then left for 16 hours at -15ºC and the resulting precipitate was filtrated. The resulting precipitate was crystalized from methanol to obtain (**17a-l**).

Characterization Date of (17a-l)

Product	Conditions	Yield [%]	Color
**(17a)**	MeOH, NaOH, 18 h, rt to -15ºC	30	Yellow
**(17b)**	MeOH, NaOH, 18 h, rt to -15ºC	33	Yellow
**(17c)**	MeOH, NaOH, 18 h, rt to -15ºC	25	Yellow
**(17d)**	MeOH, NaOH, 18 h, rt to -15ºC	29	Yellow
**(17e)**	MeOH, NaOH, 18 h, rt to -15ºC	43	Yellow
**(17f)**	MeOH, NaOH, 18 h, rt to -15ºC	31	Orange
**(17g)**	MeOH, NaOH, 18 h, rt to -15ºC	30	Orange
**(17h)**	MeOH, NaOH, 18 h, rt to -15ºC	36	Orange

**(17i)**	MeOH, NaOH, 18 h, rt to -15ºC	35	Orange
**(17j)**	MeOH, NaOH, 18 h, rt to -15ºC	42	Orange
**(17k)**	MeOH, NaOH, 18 h, rt to -15ºC	41	Red
**(17l)**	MeOH, NaOH, 18 h, rt to -15ºC	28	Red

3. NMR Spectra of Obtained Compounds

3.1 General information

All the spectra were recorded using a Varian VNMRS spectrometer operating at 11.7 T and a Varian Mercury VX 9.4 T magnetic field. Measurements were performed for ca. 1.0 M solutions of all the compounds in DMSO-d_6 or $CDCl_3$. The residual signals of DMSO-d6 (2.54 ppm) and $CDCl_3$ (7.26 ppm) in ^1H NMR and of the DMSO-d_6 signal (40.4 ppm) and of $CDCl_3$ (77.0 ppm) in ^{13}C NMR spectra were used as the chemical shift references. Spin multiplicities are described as s (singlet), d (doublet), t (triplet), q (quartet), m (multiplet), and dd (double doublet). Coupling constants are reported in Hertz. All the proton spectra were recorded using the standard spectrometer software and parameters set: acquisition time 3s and pulse angle 30°. The standard measurement parameter set for ^{13}C NMR spectra was as follows: pulse width 7 μs (the 90° pulse width was 12.5 μs), acquisition time 1 s, and spectral width 200 ppm. A total of 1000 scans of 32 K data points were accumulated and once again after zero-filling to 64 K, and the FID signals were subjected to Fourier transformation after applying a 1 Hz line broadening. The ^1H-^{13}Cgs-HSQC and ^1H-^{13}Cgs-HMBC spectra were also recorded using the standard Varian software.

3.2 Copies of ^{1}H NMR and ^{13}C NMR spectra of (17a-l)

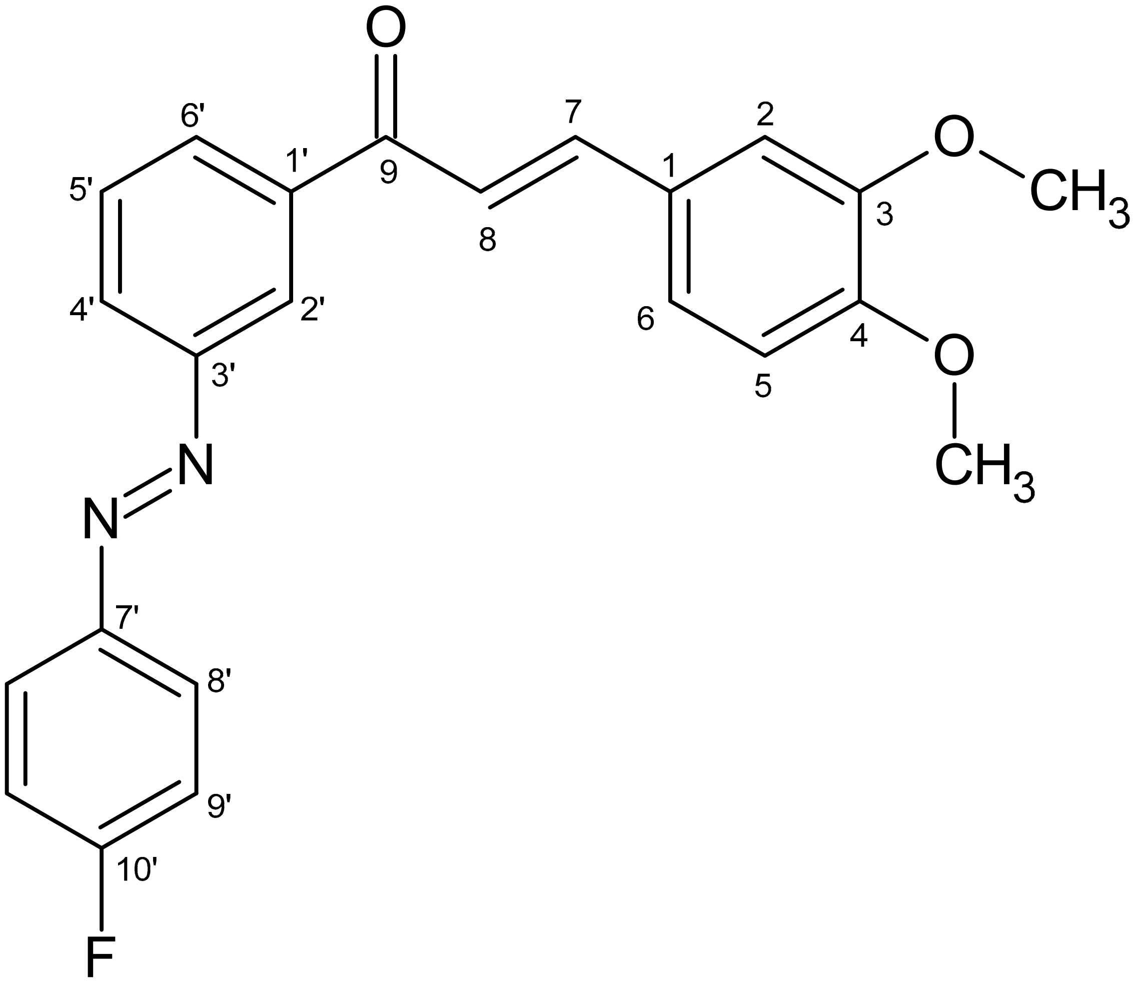

17a

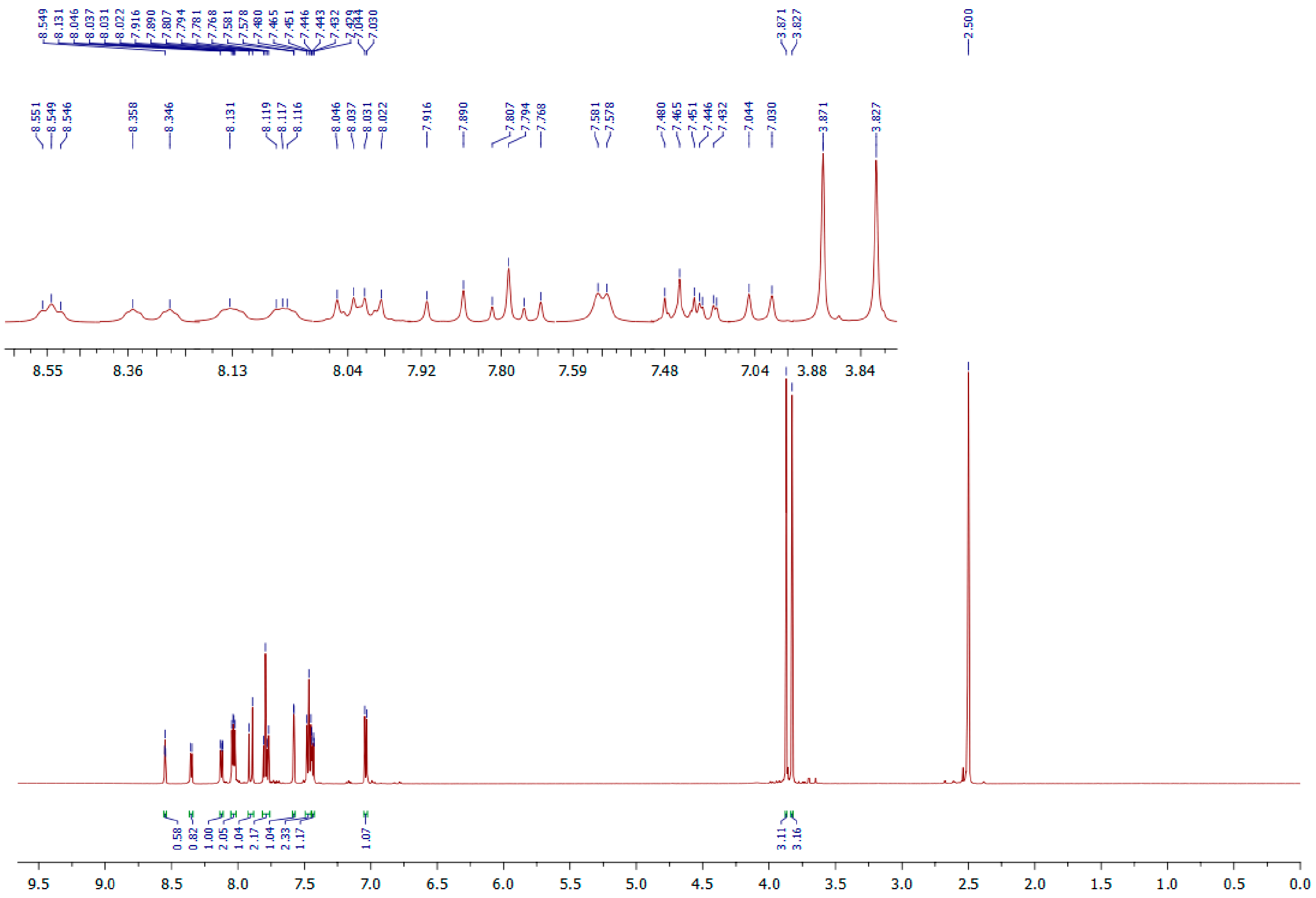

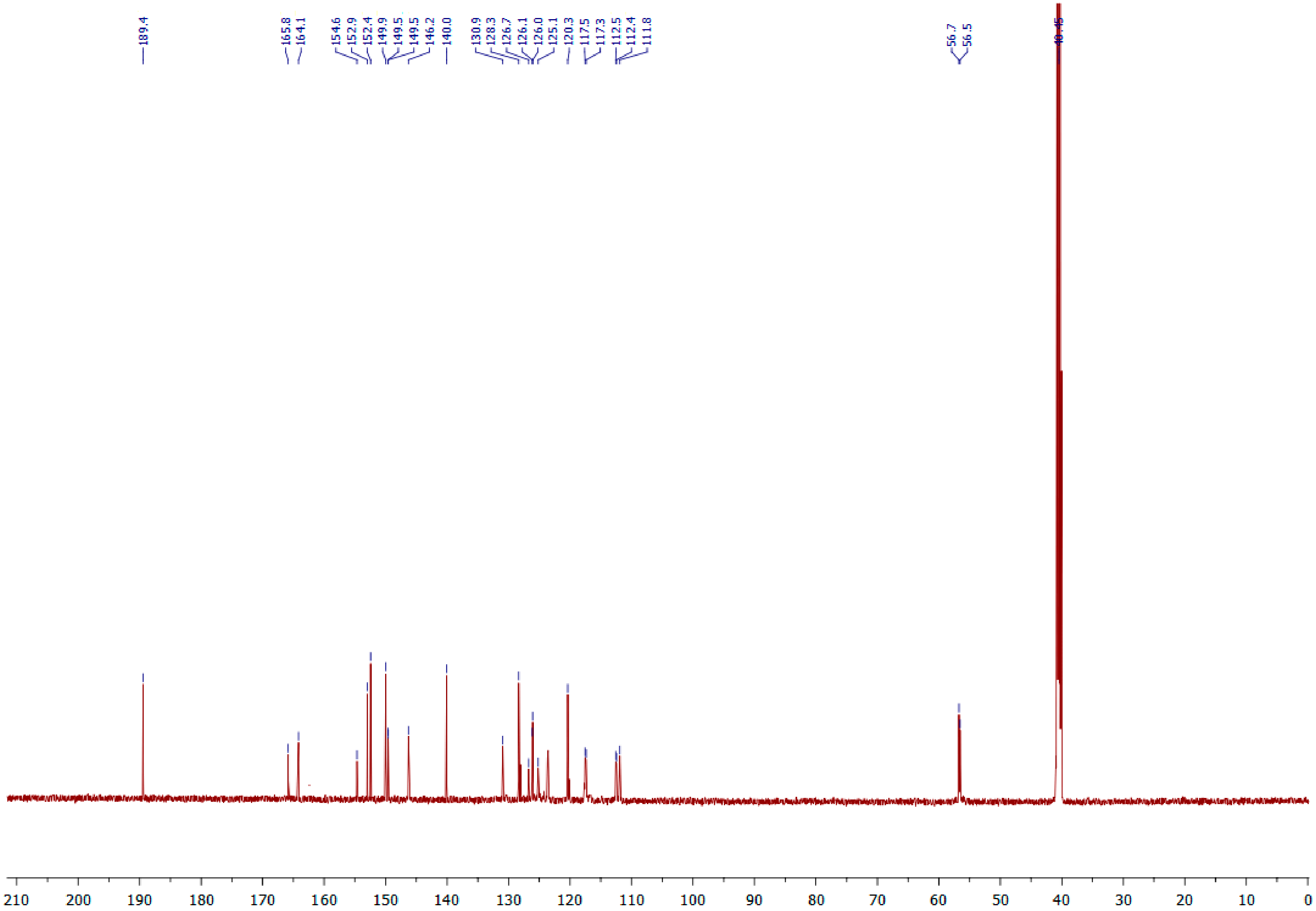
189.4
165.8
164.1
154.6
152.9
152.4
149.9
149.5
149.5
146.2
140.0
130.9
128.3
126.7
126.1
126.0
125.1
120.3
117.5
117.3
112.5
112.4
111.8
56.7
56.5
40.45
210 200 190 180 170 160 150 140 130 120 110 100 90 80 70 60 50 40 30 20 10 0

^{1}H NMR (600MHz, DMSO-d6): δ (ppm): 8.55 (1H, t, $J_{H2',H4',6'}$ = 1.8 Hz, $H_{2'}$), 8.35 (1H, dt, $J_{H5',H6'}$ = 7.8 Hz, $J_{H4',H6'}$ = 1.8 Hz, $H_{6'}$), 8.12 (1H, ddd, $J_{H4',H5'}$ = 8.4 Hz, $H_{4'}$), 8.03 (2H, dd, $J_{H8',H9'}$ = 9 Hz, $J_{H8',F}$ = 5.4 Hz, $H_{8'}$), 7.95 (1H, AB spin system, d, $J_{H7,H8}$ = 15.6 Hz, H_7), 7.79 (1H, t, $H_{5'}$), 7.78 (1H, AB spin system, d, H_8), 7.58 (1H, d, $J_{H2,H6}$ = 1.8 Hz, H_2), 7.47 (2H, t, $J_{H9',F}$ = 9 Hz, $H_{9'}$), 7.44 (1H, dd, $J_{H5,H6}$ = 8.4 Hz, H_6), 7.04 (1H, d, H_5), 3.87 (3H, s, OCH_3), 3.83 (3H, s, OCH_3).
^{13}C NMR (150MHz, DMSO-d6): δ (ppm): 189.4, 165.0 (d, J = 249 Hz), 154.6, 152.9, 152.4, 149.9, 149.5 (d, J = 3 Hz), 146.2, 140.0, 130.9, 128.3, 126.7, 126.1 (d, J = 12 Hz), 125.1, 120.3, 117.4 (d, J = 22.5 Hz), 112.5, 112.4, 111.8, 56.7, 56.5.
HRMS (ESI): $C_{23}H_{19}FN_2O_3$+H, calculated m/z 391.14525; found m/z 391.14518.

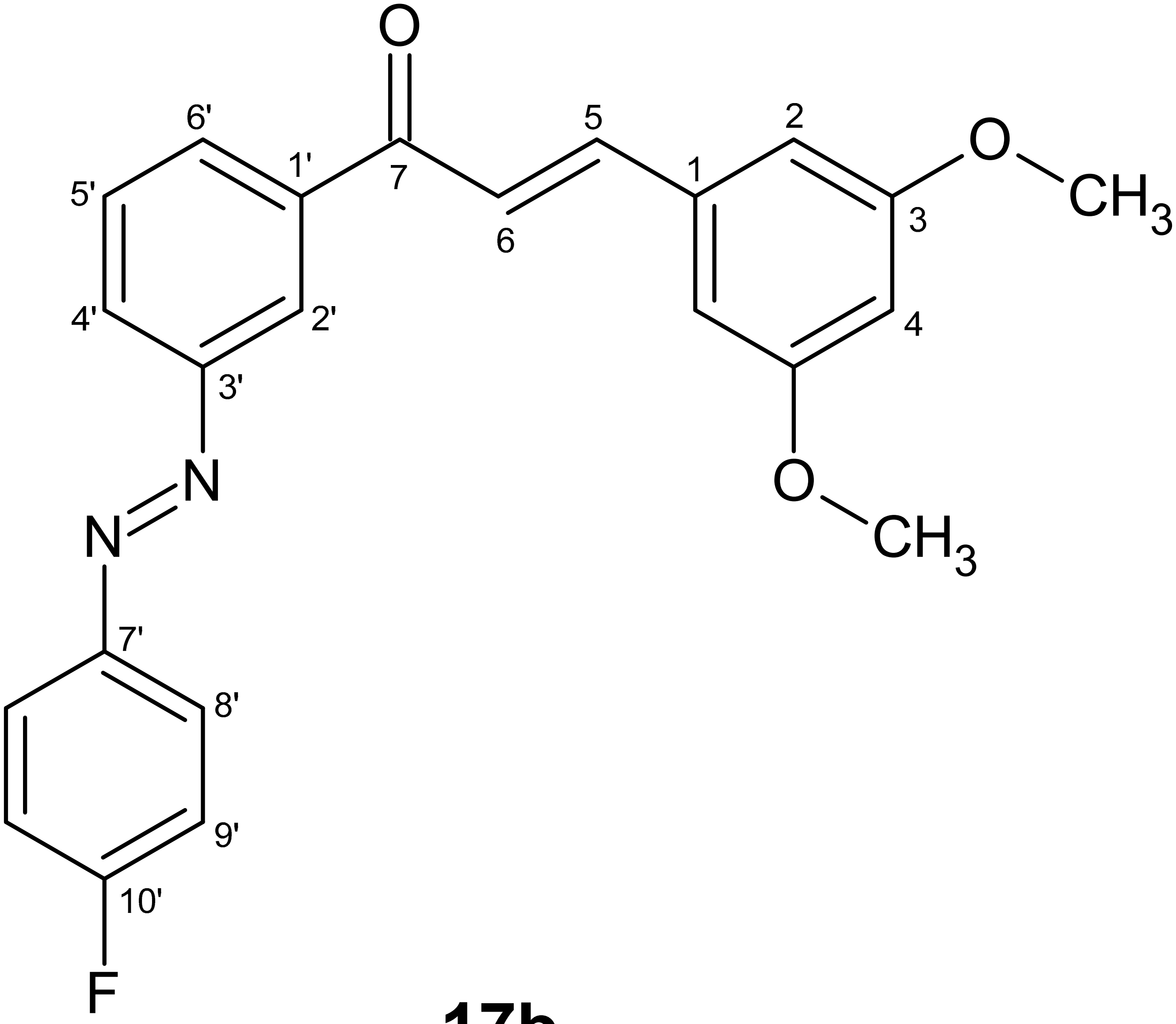

17b

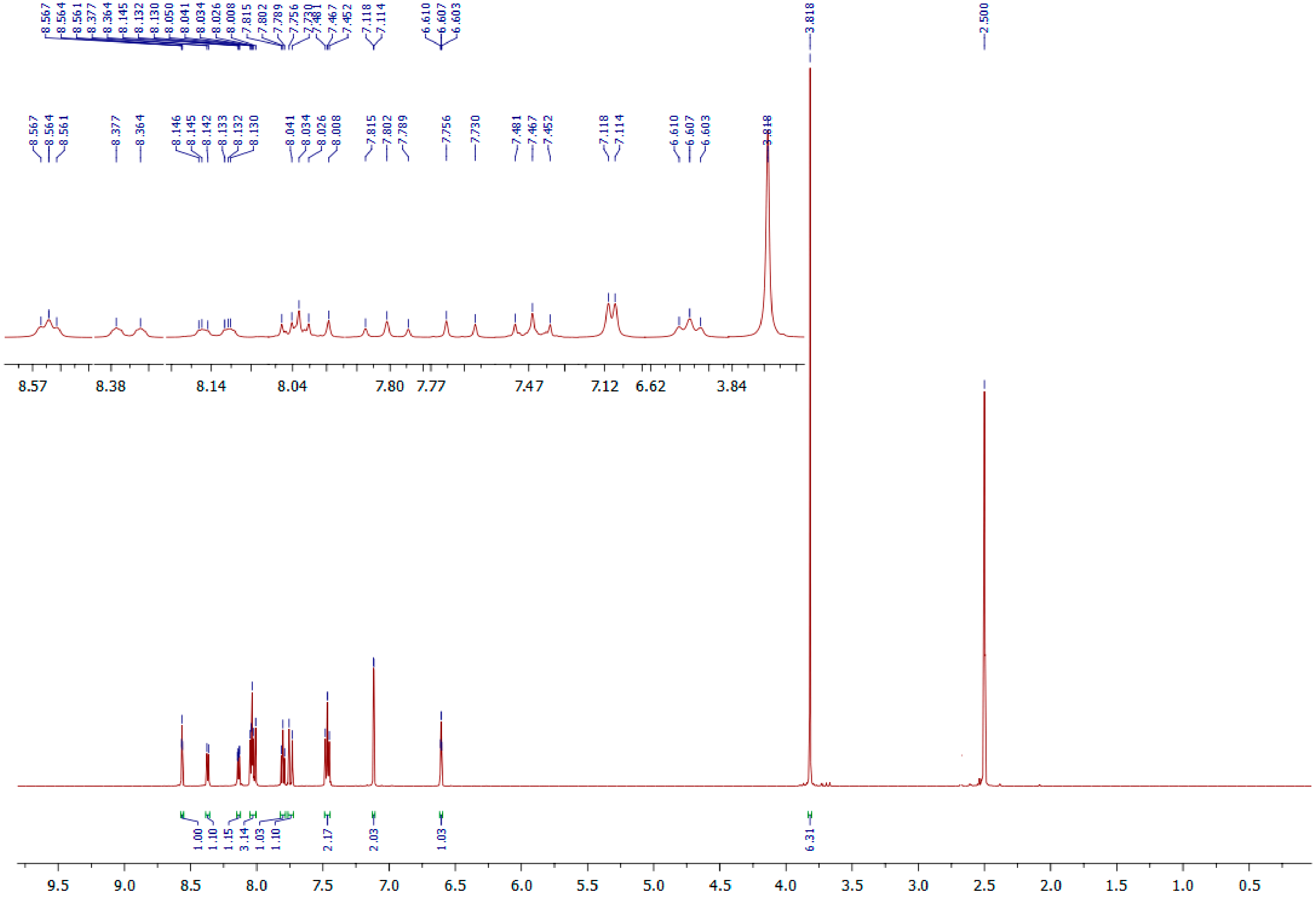
8.567 8.564 8.561 8.377 8.364 8.145 8.132 8.130 8.050 8.041 8.034 8.026 8.008 7.815 7.802 7.789 7.756 7.730 7.481 7.467 7.452 7.118 7.114 6.610 6.607 6.603
3.818
2.500
8.567 8.564 8.561 8.377 8.364 8.146 8.145 8.142 8.133 8.132 8.130 8.041 8.034 8.026 8.008 7.815 7.802 7.789 7.756 7.730 7.481 7.467 7.452 7.118 7.114 6.610 6.607 6.603 3.818
8.57 8.38 8.14 8.04 7.80 7.77 7.47 7.12 6.62 3.84
1.00 1.10 1.15 3.14 1.03 1.10 2.17 2.03 1.03 6.31
9.5 9.0 8.5 8.0 7.5 7.0 6.5 6.0 5.5 5.0 4.5 4.0 3.5 3.0 2.5 2.0 1.5 1.0 0.5

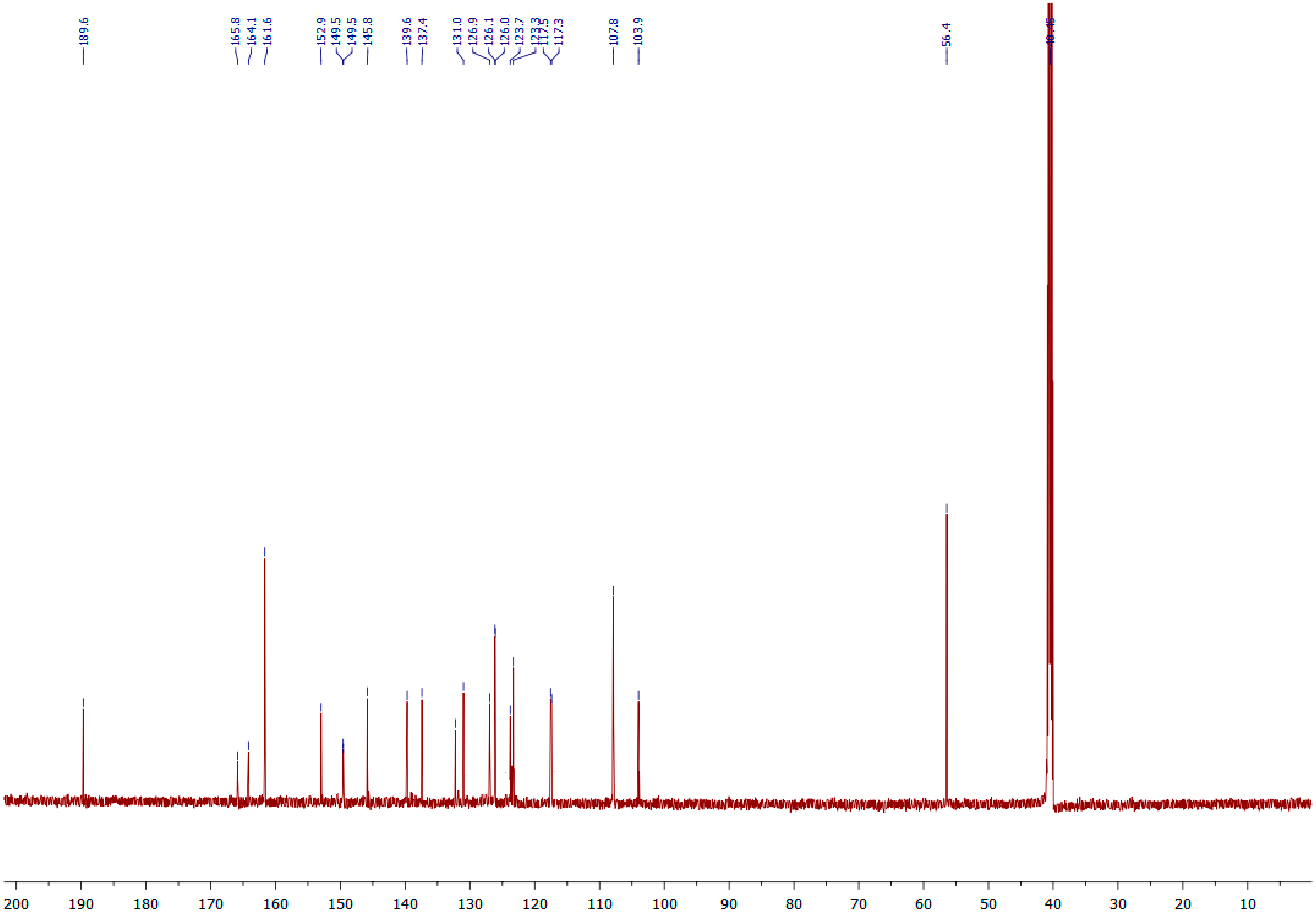
189.6
165.8
164.1
161.6
152.9
149.5
149.5
145.8
139.6
137.4
131.0
126.9
126.1
126.0
123.7
123.3
117.5
117.3
107.8
103.9
56.4
200
190
180
170
160
150
140
130
120
110
100
90
80
70
60
50
40
30
20
10

^{1}H NMR (600MHz, DMSO-d6): δ (ppm): 8.56 (1H, t, $J_{H2',H4',6'}$ = 1.8 Hz, $H_{2'}$), 8.36 (1H, dt, $J_{H5',H6'}$ = 7.8 Hz, $J_{H4',H6'}$ = 1.8 Hz, $H_{6'}$), 8.14 (1H, ddd, $J_{H4',H5'}$ = 7.8 Hz, $H_{4'}$), 8.04 (2H, dd, $J_{H8',H9'}$ = 9 Hz, $J_{H8',F}$ = 4.8 Hz, $H_{8'}$), 8.02 (1H, AB spin system, d, $J_{H5,H6}$ = 15.6 Hz, H_5), 7.80 (1H, t, $H_{5'}$), 7.74 (1H, AB spin system, d, H_6), 7.47 (1H, t, $J_{H9',F}$ = 9 Hz, $H_{9'}$), 7.12 (2H, d, $J_{H2,H4}$ = 2.4 Hz, H_2), 6.61 (1H, t, H_4), 3.82 (6H, s, OCH_3).
^{13}C NMR (150MHz, DMSO-d6): δ (ppm): 189.6, 165.0 (d, J = 250.5 Hz), 161.6, 152.9, 149.5 (d, J = 3 Hz), 145.8, 139.6, 137.4, 132.2, 131.00, 126.9, 126.1 (d, J = 9 Hz), 123.7, 123.3, 117.4 (d, J = 22.5 Hz), 107.8, 103.9, 56.4.
HRMS (ESI): $C_{23}H_{19}FN_2O_3$+H, calculated m/z 391.14525; found m/z 391.14505.

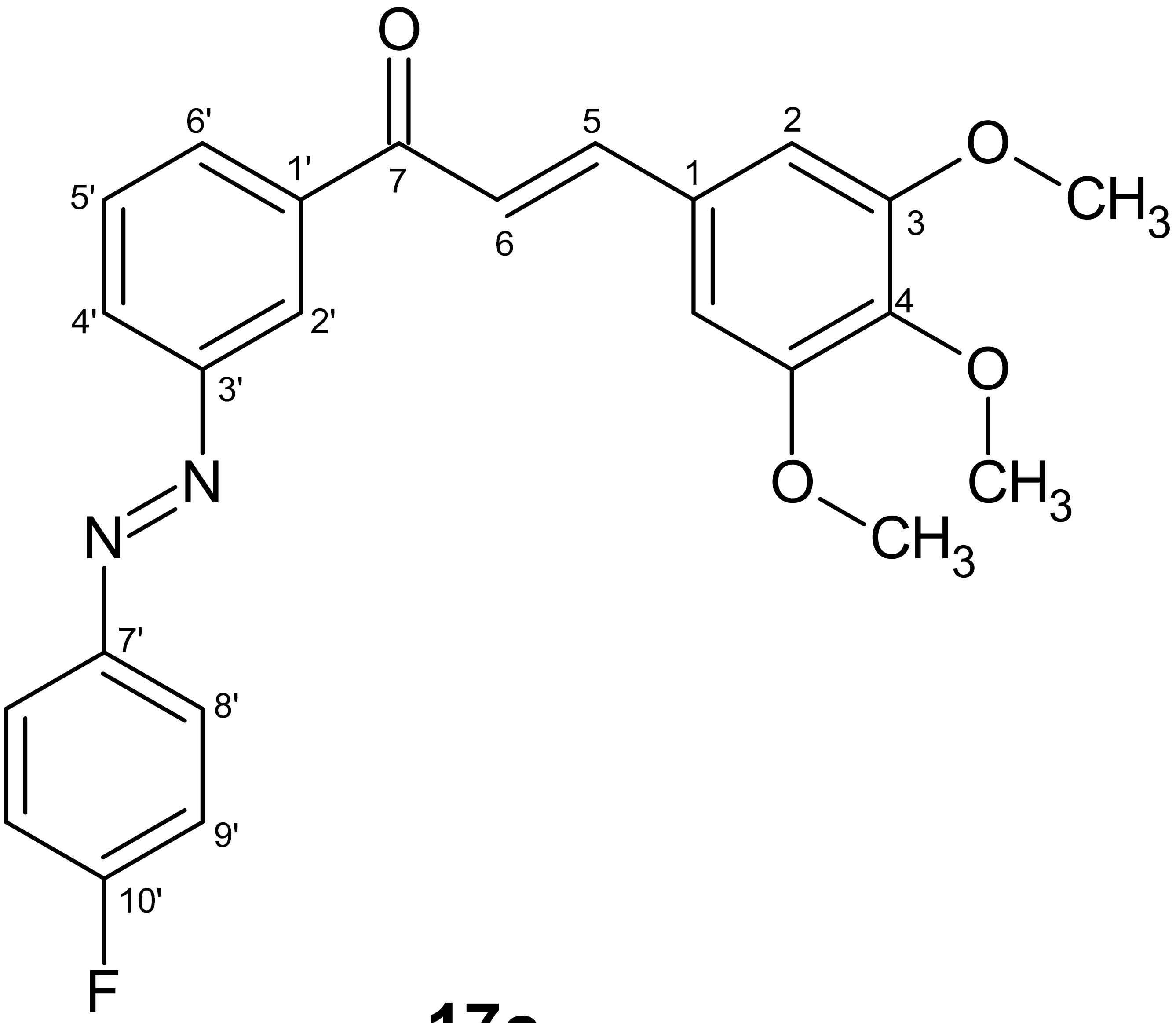

17c

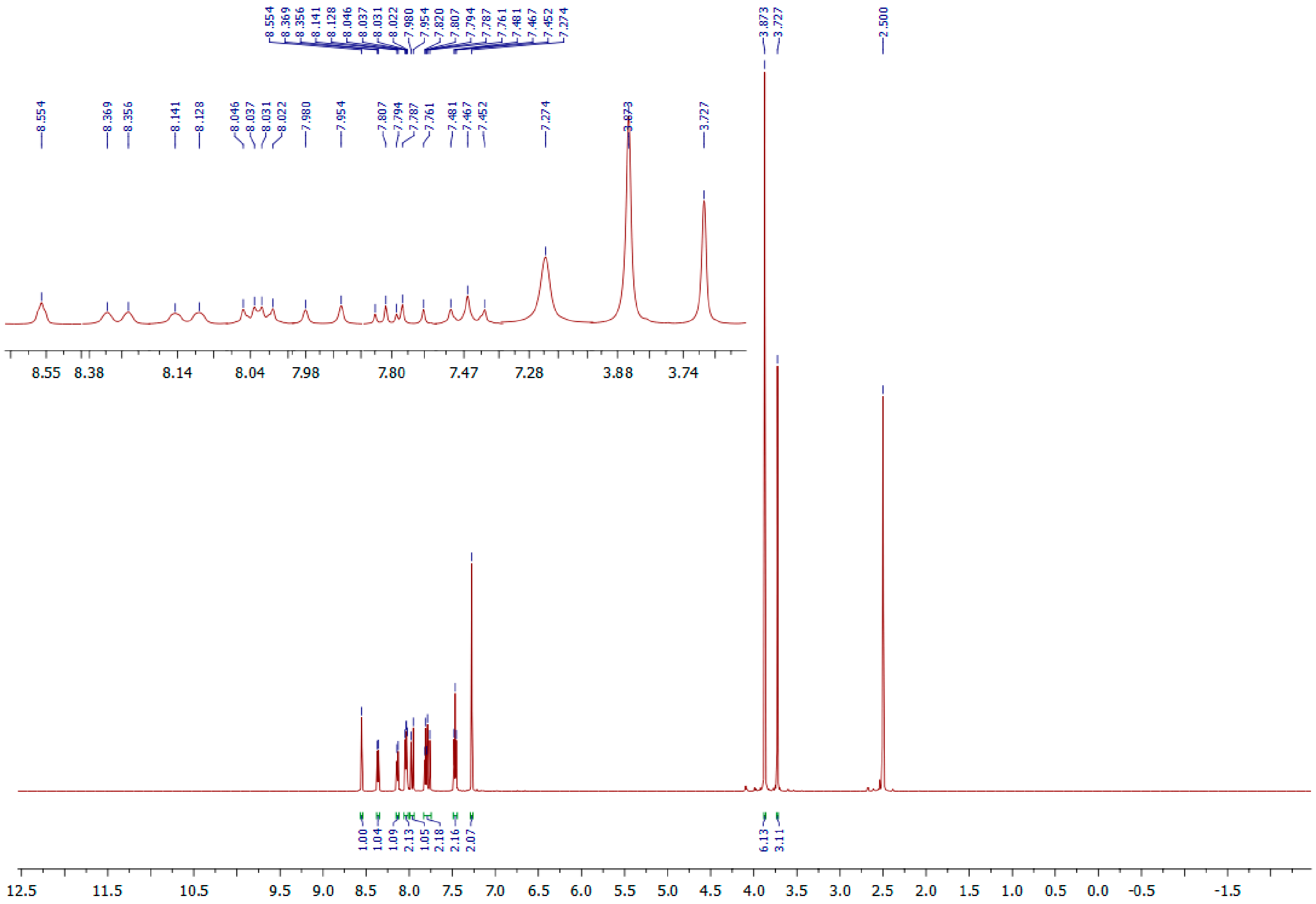
8.554
8.369
8.356
8.141
8.128
8.046
8.037
8.031
8.022
7.980
7.954
7.820
7.807
7.794
7.787
7.761
7.481
7.467
7.452
7.274
3.873
3.727
2.500
1.00
1.04
1.09
2.13
1.05
2.18
2.16
2.07
6.13
3.11

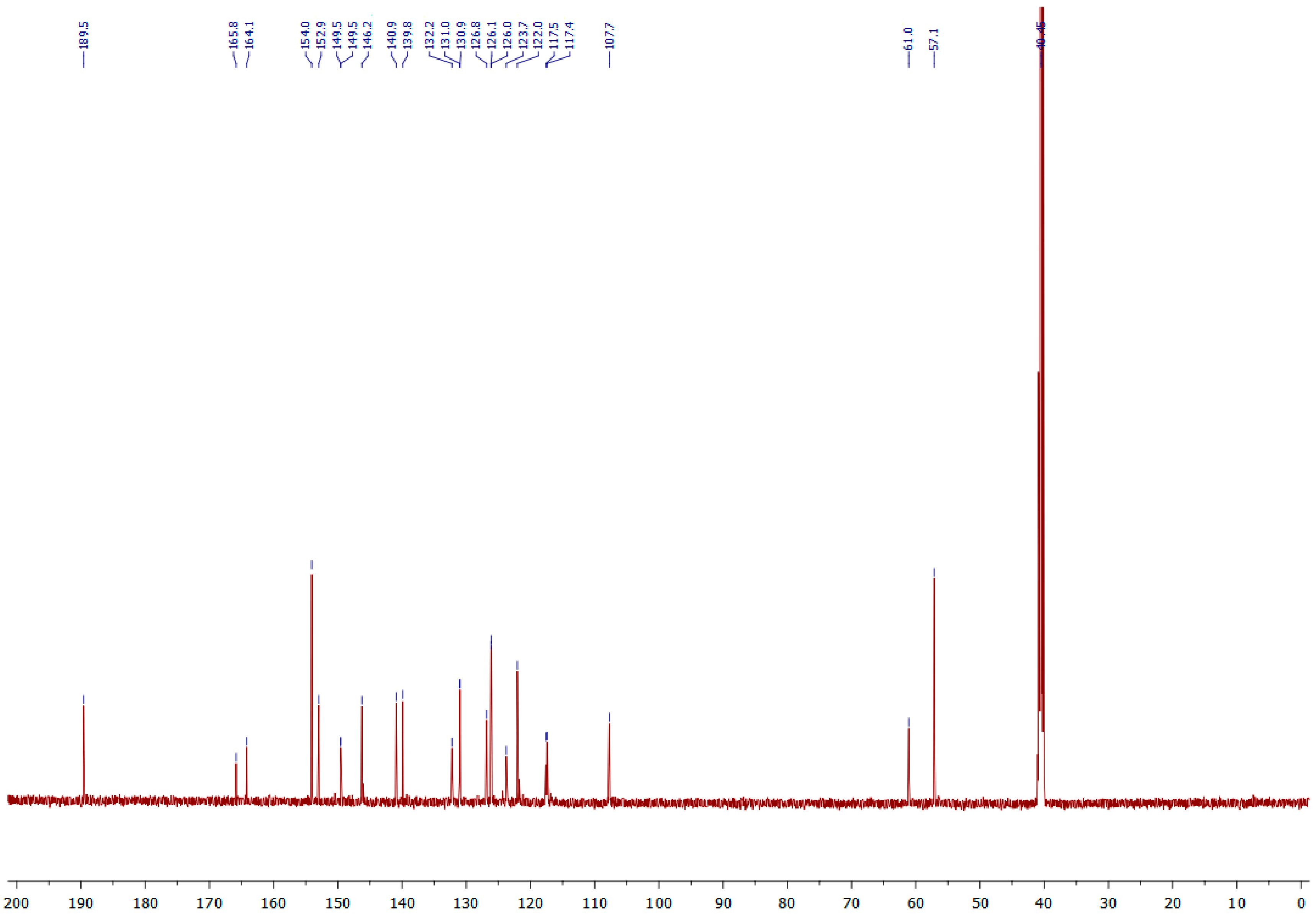
189.5
165.8
164.1
154.0
152.9
149.5
149.5
146.2
140.9
139.8
132.2
131.0
130.9
126.8
126.1
126.0
123.7
122.0
117.5
117.4
107.7
61.0
57.1
200 190 180 170 160 150 140 130 120 110 100 90 80 70 60 50 40 30 20 10 0

^{1}H NMR (600MHz, DMSO-d6): δ (ppm): 8.55 (1H, t, $J_{H2',H4',6'}$ = 1.8 Hz, $H_{2'}$), 8.36 (1H, dt, $J_{H5',H6'}$ = 7.8 Hz, $J_{H4',H6'}$ = 1.8 Hz, $H_{6'}$), 8.13 (1H, ddd, $J_{H4',H5'}$ = 9 Hz, $H_{4'}$), 8.03 (2H, dd, $J_{H8',H9'}$ = 9 Hz, $J_{H8',F}$ = 5.4 Hz, $H_{8'}$), 7.97 (1H, AB spin system, d, $J_{H7,H8}$ = 15.6 Hz, H_7), 7.81 (1H, t, $H_{5'}$), 7.77 (1H, AB spin system, d, H_8), 7.47 (2H, t, $J_{H9',F}$ = 9 Hz, $H_{9'}$), 7.27 (2H, s, H_2), 3.87 (6H, s, OCH_3), 3.73 (3H, s, OCH_3).
^{13}C NMR (150MHz, DMSO-d6): δ (ppm): 189.5, 165.0 (d, J = 250.5 Hz), 154.0, 152.9, 149.5 (d, J = 1.5 Hz), 146.2, 140.9, 139.8, 132.2, 131.0, 130.9, 126.8, 126.1 (d, J = 9 Hz), 123.7, 122.0, 117.5 (d, J = 22.5 Hz), 107.7, 61.0, 57.1.
HRMS (ESI): $C_{24}H_{21}FN_2O_4$+H, calculated m/z 421.15581; found m/z 421.15559.

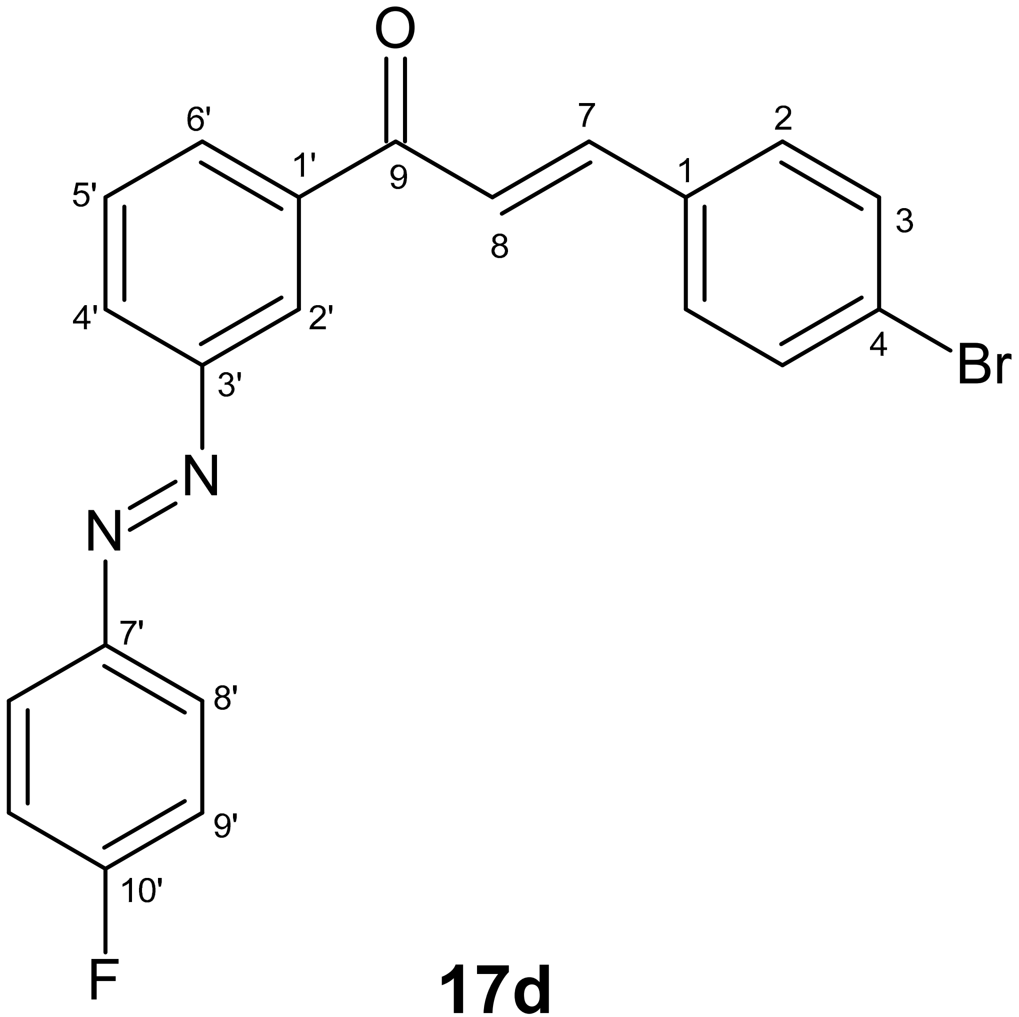

17d

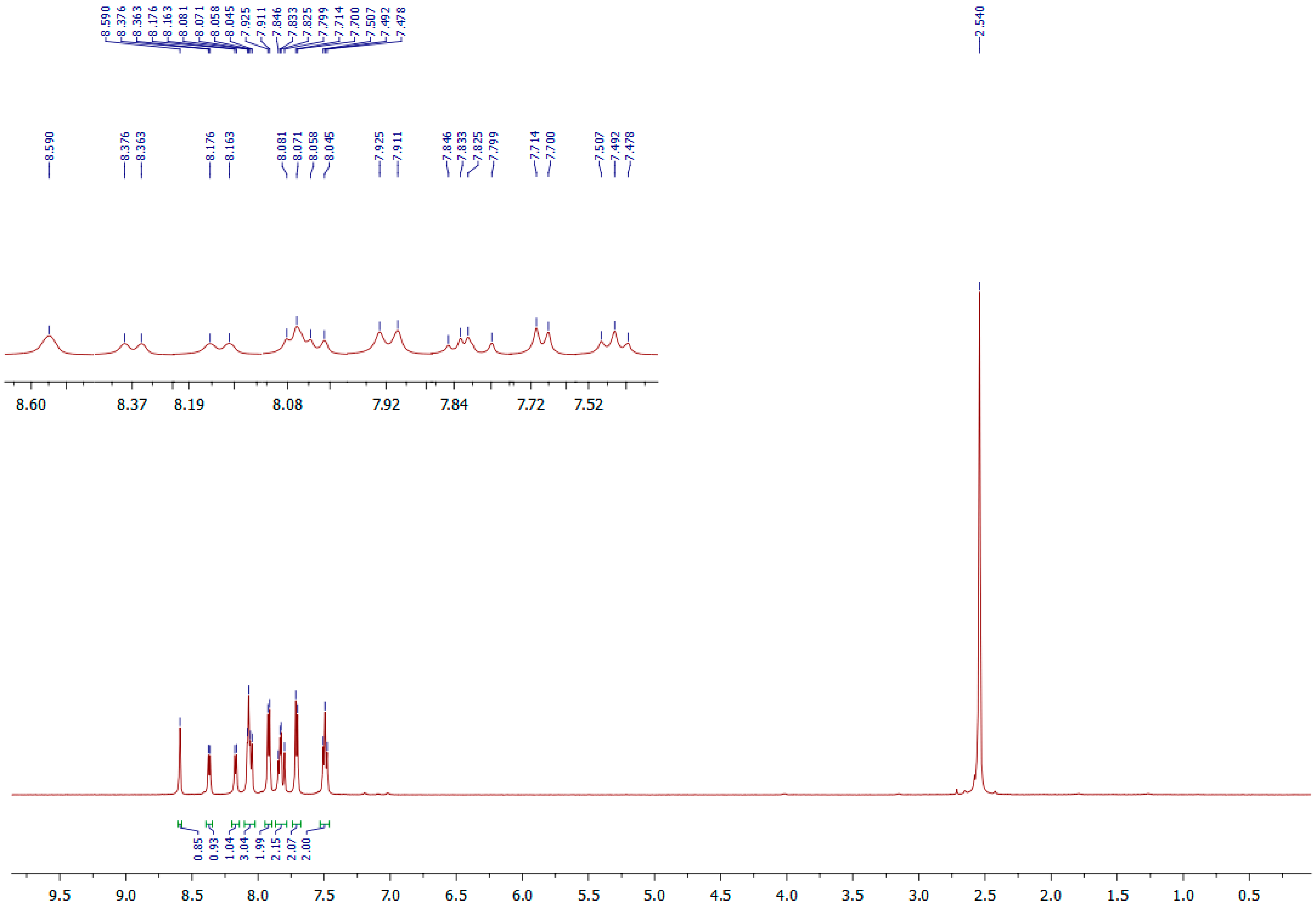
8.590
8.376
8.363
8.176
8.163
8.081
8.071
8.058
8.045
7.925
7.911
7.846
7.833
7.825
7.799
7.714
7.700
7.507
7.492
7.478
2.540
8.60
8.37
8.19
8.08
7.92
7.84
7.72
7.52
0.85
0.93
1.04
3.04
1.99
2.15
2.07
2.00
9.5
9.0
8.5
8.0
7.5
7.0
6.5
6.0
5.5
5.0
4.5
4.0
3.5
3.0
2.5
2.0
1.5
1.0
0.5

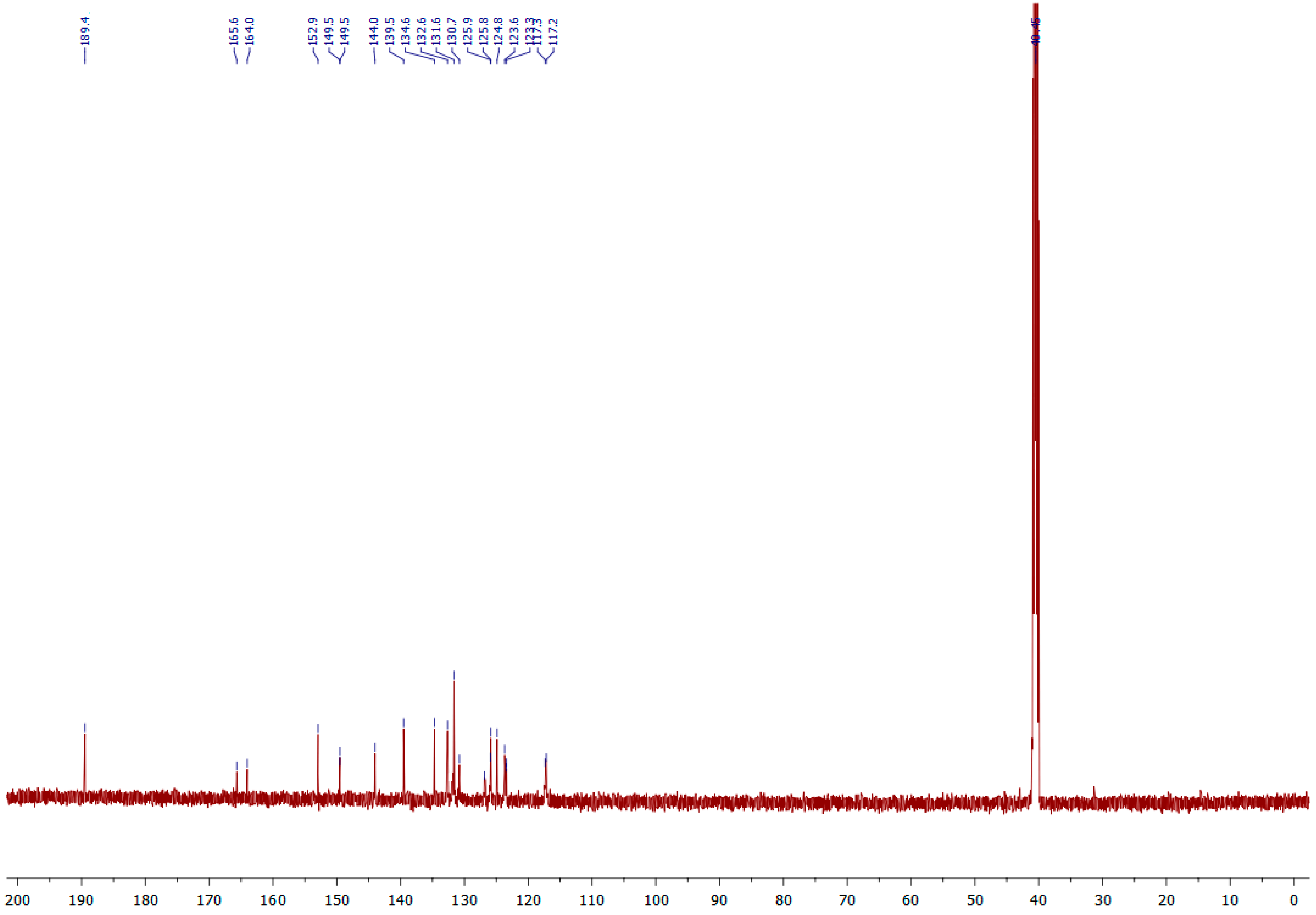
189.4
165.6
164.0
152.9
149.5
149.5
144.0
139.5
134.6
132.6
131.6
130.7
125.9
125.8
124.8
123.6
123.3
117.3
117.2
200 190 180 170 160 150 140 130 120 110 100 90 80 70 60 50 40 30 20 10 0

^{1}H NMR (600MHz, DMSO-d6): δ (ppm): 8.59 (1H, t, $J_{H2',H4',6'}$ = 1.8 Hz, $H_{2'}$), 8.37 (1H, dt, $J_{H5',H6'}$ = 7.8 Hz, $J_{H4',H6'}$ = 1.8 Hz, $H_{6'}$), 8.17 (1H, ddd, $J_{H4',H5'}$ = 7.8 Hz, $H_{4'}$), 8.07 (2H, dd, $J_{H8',H9'}$ = 9.0 Hz, $J_{H8',F}$ = 5.4 Hz, $H_{8'}$), 8.06 (1H, AB spin system, d, $J_{H7,H8}$ = 15.6 Hz, H_7), 7.92 (2H, d, $J_{H2,H3}$ = 8.4 Hz, H_3), 7.83 (1H, t, $H_{5'}$), 7.81 (1H, AB spin system, d, H_8), 7.71 (2H, d, H_2), 7.49 (2H, t, $J_{H9',F}$ = 9 Hz, $H_{9'}$).
^{13}C NMR (150MHz, DMSO-d6): δ (ppm): 189.4, 164.8 (d, J = 249 Hz), 152.9, 149.5 (d, J = 1.5 Hz), 144.0, 139.5, 134.6, 132.6, 131.6, 130.78, 126.8, 125.9 (d, J = 10.5 Hz), 124.8, 123.6, 123.4, 123.3, 117.3 (d, J = 22.5 Hz).
HRMS (ESI): $C_{21}H_{14}BrFN_2O$+H, calculated m/z 409.03463; found m/z 409.03459.

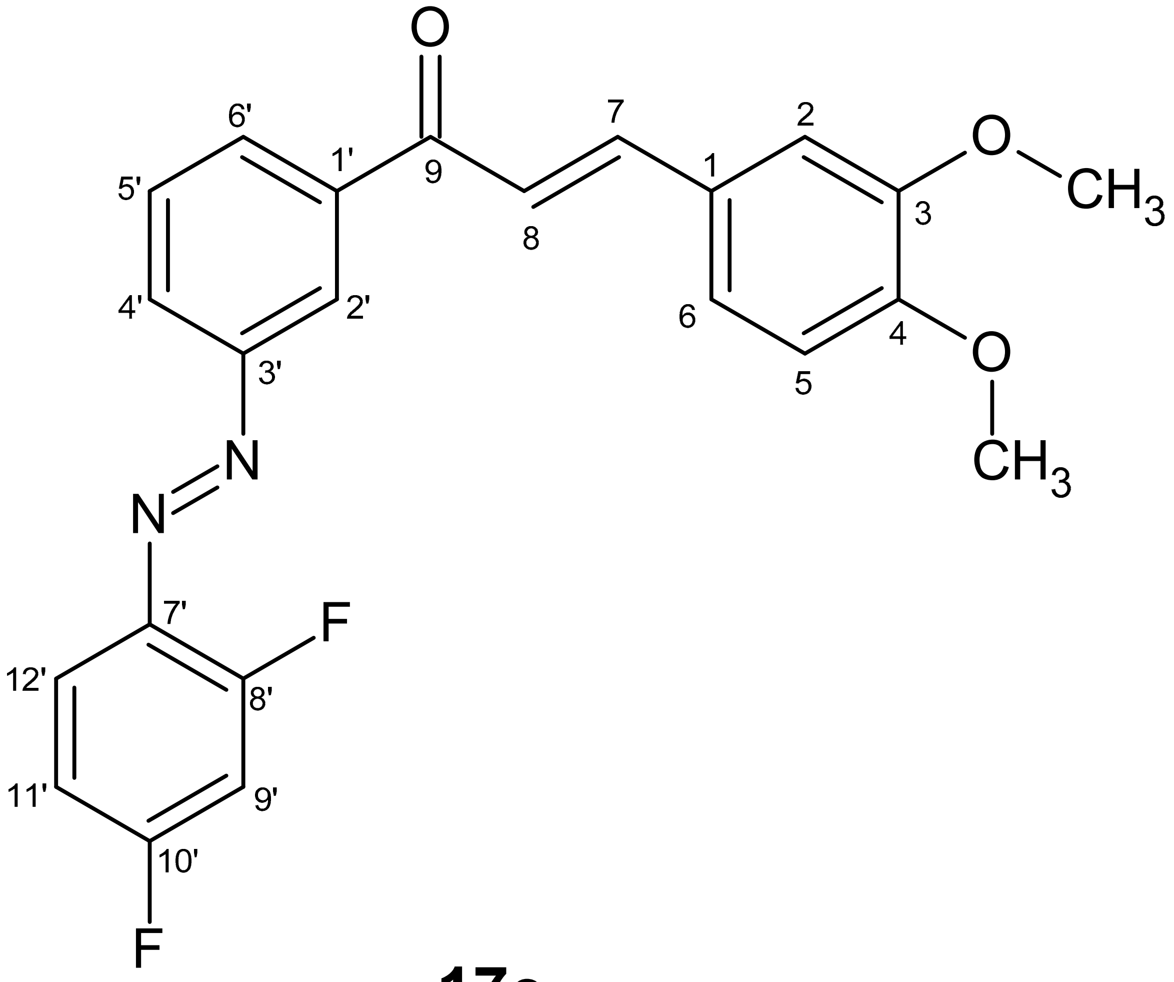

17e

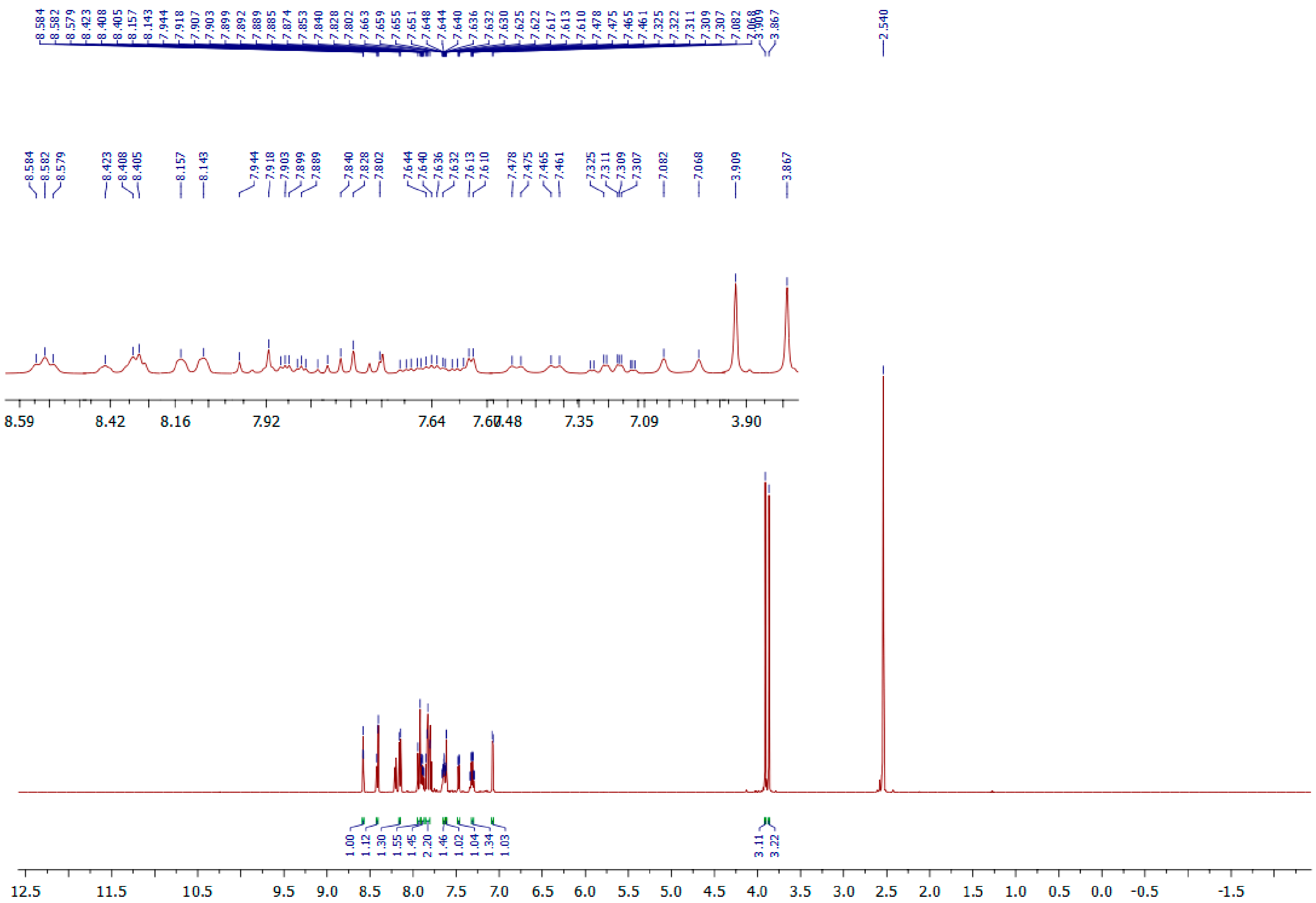

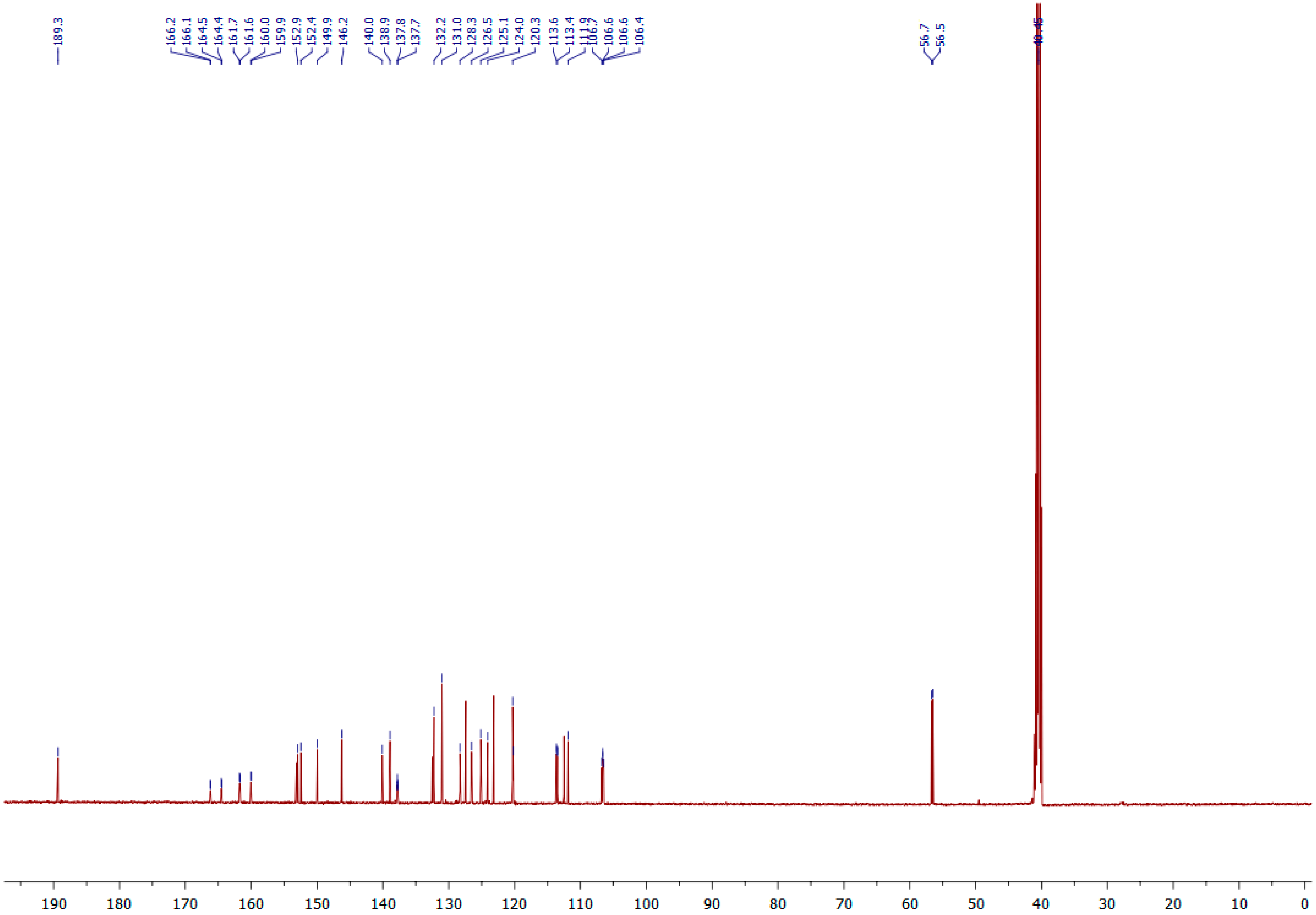
189.3
166.2
166.1
164.5
164.4
161.7
161.6
160.0
159.9
152.9
152.4
149.9
146.2
140.0
138.9
137.8
137.7
132.2
131.0
128.3
126.5
125.1
124.0
120.3
113.6
113.4
111.9
106.7
106.6
106.6
106.4
56.7
56.5
190 180 170 160 150 140 130 120 110 100 90 80 70 60 50 40 30 20 10 0

^{1}H NMR (600MHz, DMSO-d6): δ (ppm): 8.58 (1H, t, $J_{H2',H4',6'}$ = 1.8 Hz, $H_{2'}$), 8.41 (1H, dt, $J_{H5',H6'}$ = 7.8 Hz, $J_{H4',H6'}$ = 1.8 Hz, $H_{6'}$), 8.16 (1H, ddd, $J_{H4',H5'}$ = 7.8 Hz, $H_{4'}$), 7.93 (1H, AB spin system, d, $J_{H7,H8}$ = 15.6 Hz, H_7), 7.90 (1H, dt, $J_{H11',H12'}$ = 9 Hz, $J_{H12',F}$ = 6.6 Hz, $H_{12'}$), 7.84 (1H, t, $H_{5'}$), 7.82 (1H, AB spin system, d, H_8), 7.64 (1H, ddd, $J_{H11',F}$ = 9 Hz, $J_{H11',H9'}$ = 2.6 Hz), 7.61 (1H, d, $J_{H2,H6}$ = 1.8 Hz, H_2), 7.47 (1H, dd, $J_{H5,H6}$ = 7.8 Hz, H_6), 7.32 (1H, td, $J_{H9',F}$ = 8.4 Hz, $H_{9'}$), 7.08 (1H, d, H_5), 3.91 (3H, s, OCH_3), 3.87 (3H, s, OCH_3).
^{13}C NMR (150MHz, DMSO-d6): δ (ppm): 189.3, 165.3 (dd, J = 252 Hz, J = 12 Hz), 160.8 (dd, J = 256.5 Hz, J = 12 Hz), 152.9, 152.4, 149.96, 146.2, 140.0, 138.9, 137.8 (dd, J = 15 Hz, J = 7.5 Hz), 132.2, 131.0, 128.3, 126.5, 125.1, 124.0, 120.2 (d, J = 13.5 Hz), 113.5 (dd, J = 27 Hz, J = 3 Hz), 111.9, 106.6 (dd, J = 27 Hz, J = 24 Hz), 56.7, 56.5.
HRMS (ESI): $C_{23}H_{18}F_2N_2O_3$+H, calculated m/z 409.13583; found m/z 409.13569.

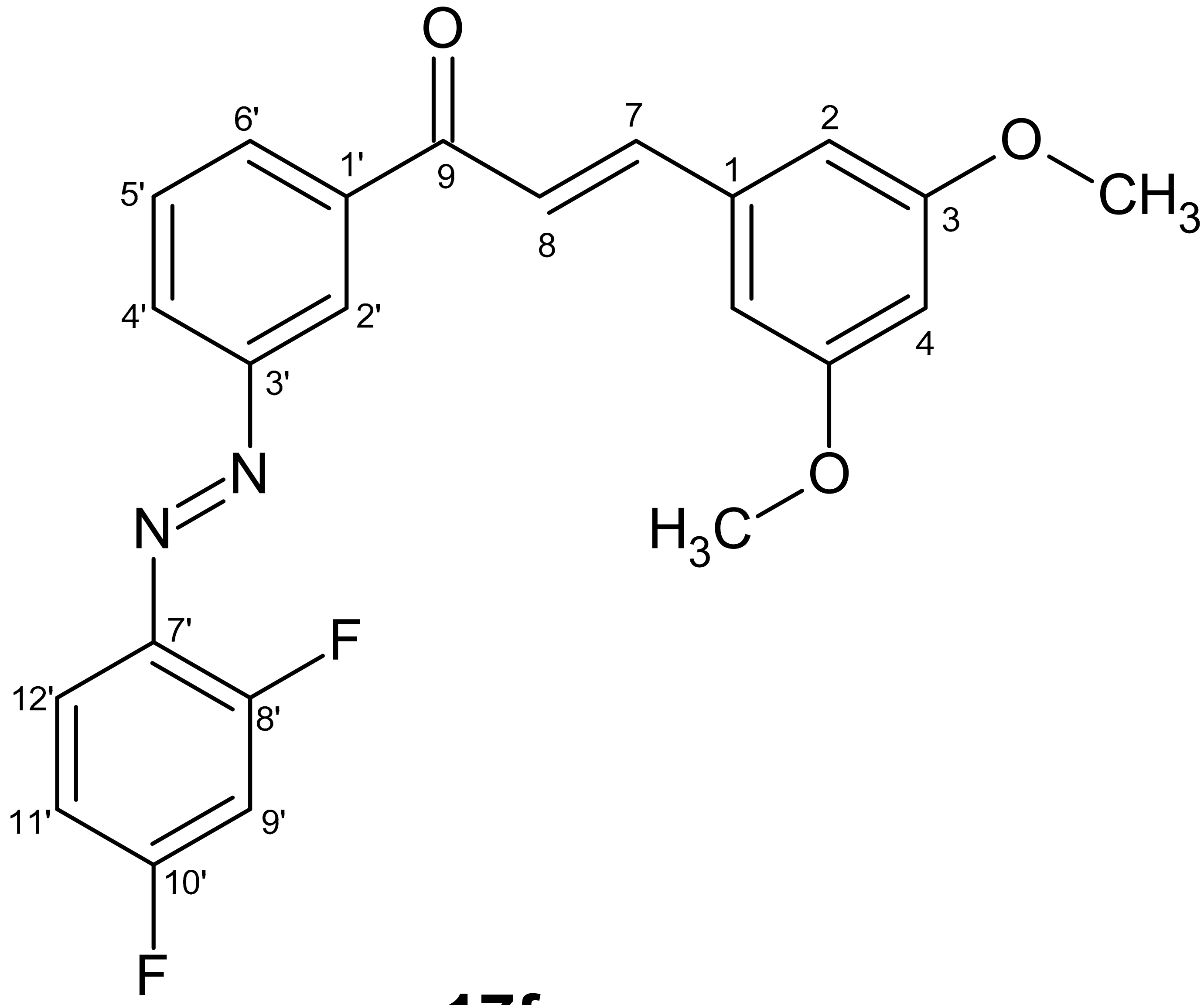

17f

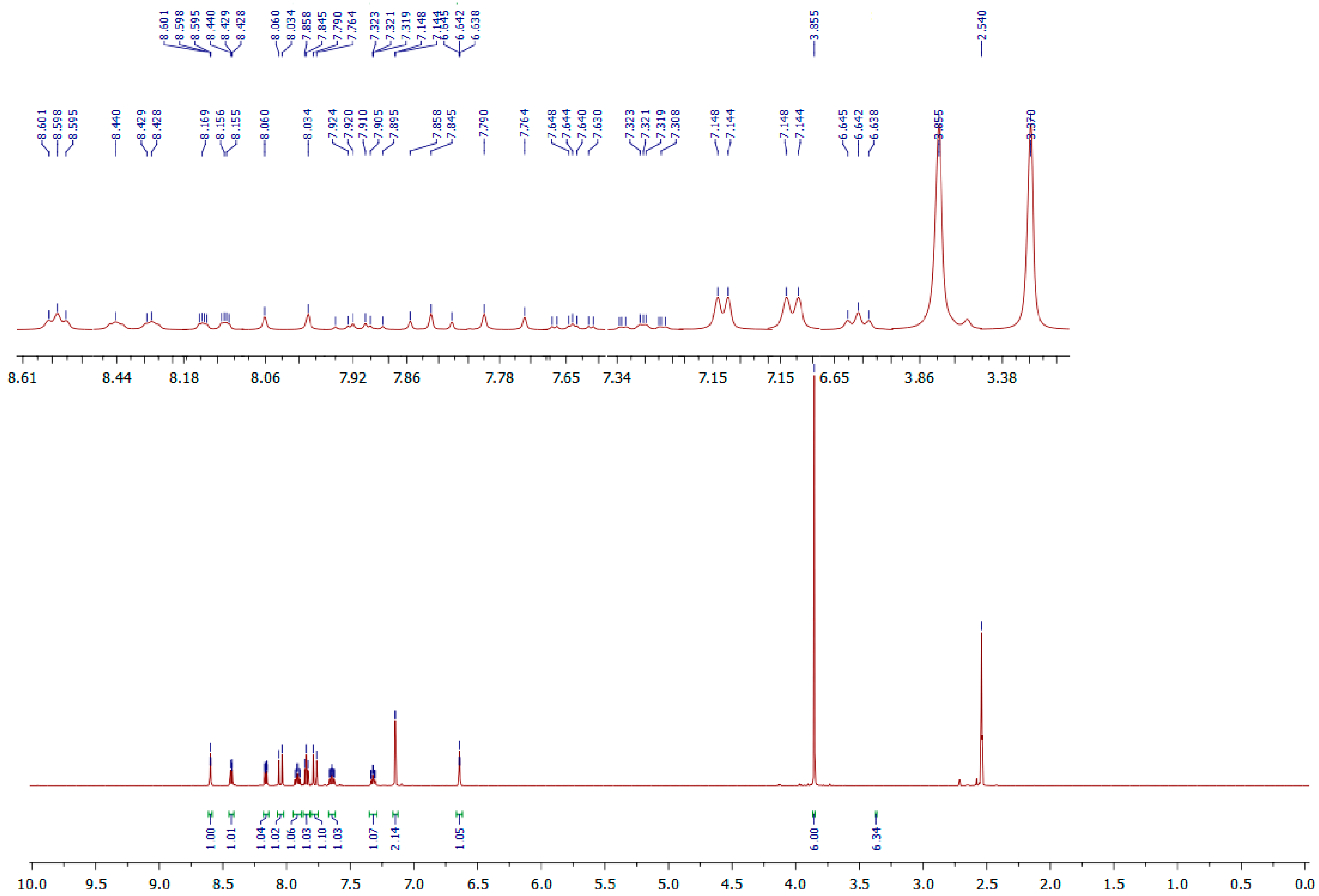

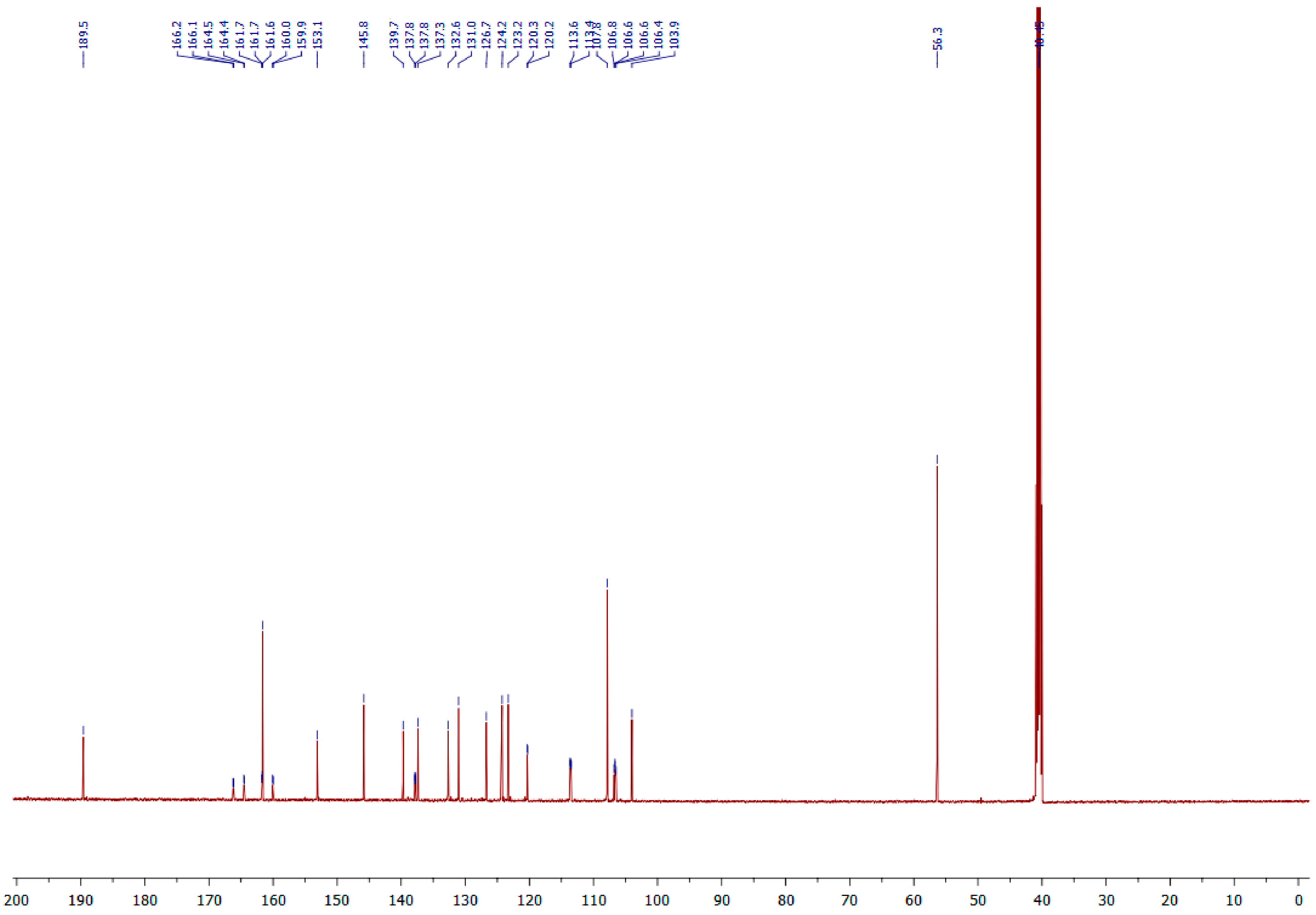
189.5
166.2
166.1
164.5
164.4
161.7
161.7
161.6
160.0
159.9
153.1
145.8
139.7
137.8
137.8
137.3
132.6
131.0
126.7
124.2
123.2
120.3
120.2
113.6
106.8
106.6
106.6
106.4
103.9
56.3
200 190 180 170 160 150 140 130 120 110 100 90 80 70 60 50 40 30 20 10 0

^{1}H NMR (600MHz, DMSO-d6): δ (ppm): 8.60 (1H, t, $J_{H2',H4',6'}$ = 1.8 Hz, $H_{2'}$), 8.43 (1H, dt, $J_{H5',H6'}$ = 7.2 Hz, $J_{H4',H6'}$ = 1.8 Hz, $H_{6'}$), 8.16 (1H, ddd, $J_{H4',H5'}$ = 7.8 Hz, $H_{4'}$), 8.05 (1H, AB spin system, d, $J_{H7,H8}$ = 15.6 Hz, H_7), 7.92 (1H, dt, $J_{H11',H12'}$ = 9 Hz, $J_{H12',F}$ = 6.6 Hz, $H_{12'}$), 7.85 (1H, t, $H_{5'}$), 7.78 (1H, AB spin system, d, H_8), 7.64 (1H, ddd, $J_{H11',F}$ = 9 Hz, $J_{H11',H9'}$ = 2.6 Hz), 7.32 (1H, td, $J_{H9',F}$ = 8.4 Hz, $H_{9'}$), 7.15 (2H, d, $J_{H2,H4}$ = 2.4 Hz, H_2), 6.64 (1H, t, H_4), 3.86 (6H, s, OCH_3).
^{13}C NMR (150MHz, DMSO-d6): δ (ppm): 189.5, 165.3 (dd, J = 250.5 Hz, J = 12 Hz), 161.6, 160.8 (dd, J = 258 Hz, J = 13.5 Hz), 153.1, 145.8, 139.7, 137.8, (dd, J = 7.5 Hz, J = 4.5 Hz), 137.3, 132.6, 131.0, 126.7, 124.2, 123.2, 120.2 (d, J = 10.5 Hz), 113.5 (dd, J = 24 Hz, J = 4.5 Hz), 107.8, 106.6 (dd, J = 27 Hz, J = 24 Hz), 103.9, 56.3.
HRMS (ESI): $C_{23}H_{18}F_2N_2O_3$+H, calculated m/z 409.13583; found m/z 409.13568.

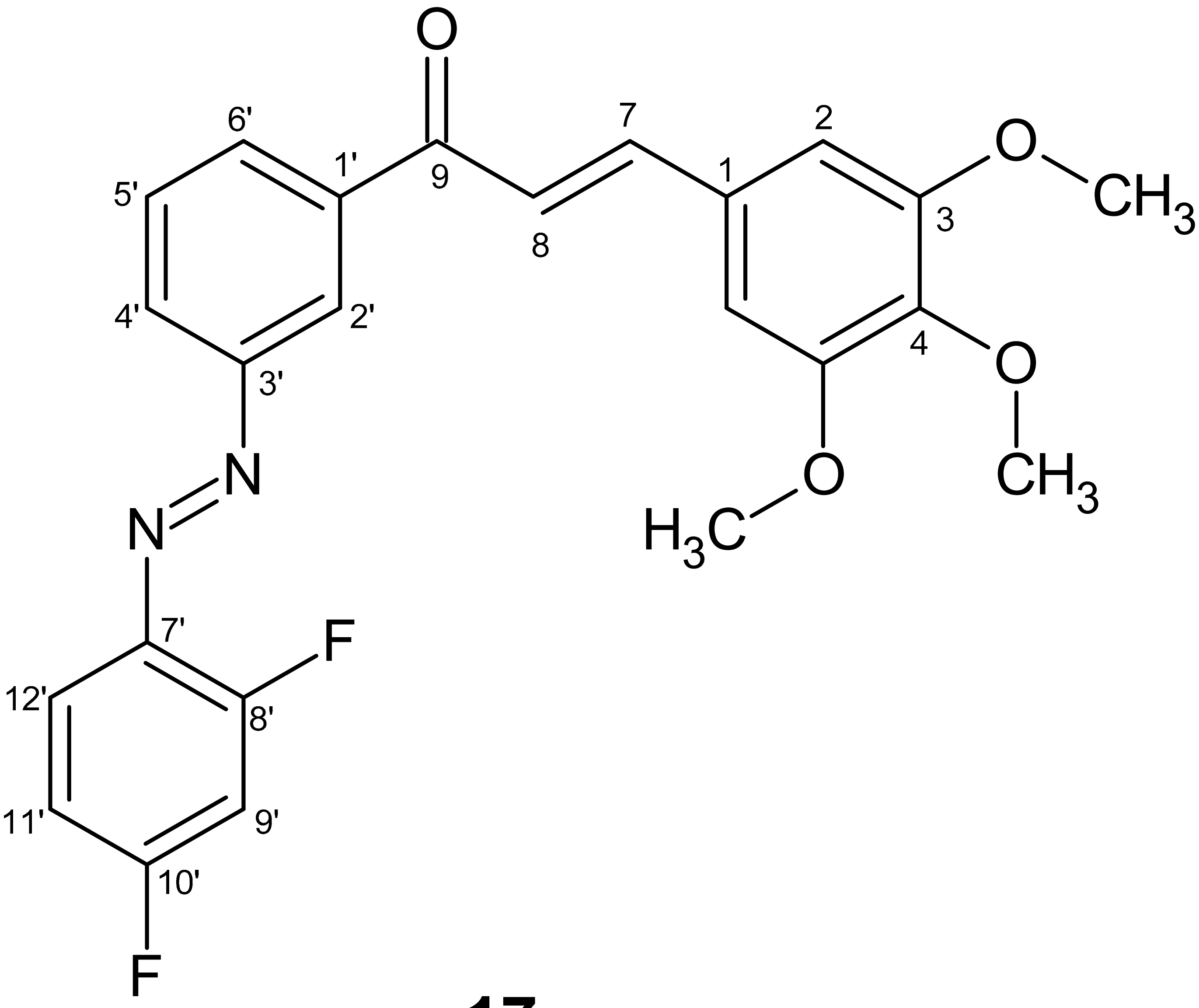

17g

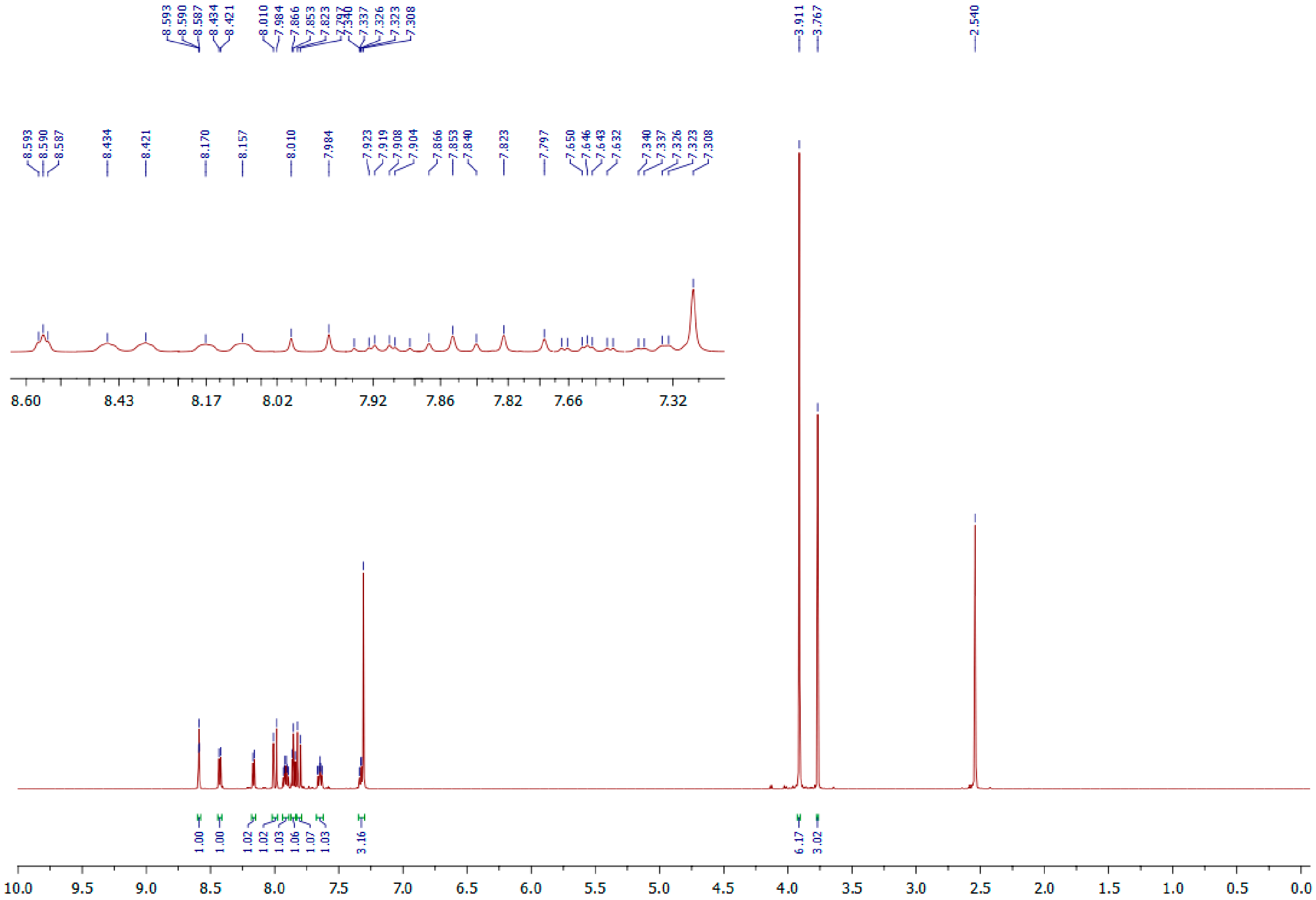

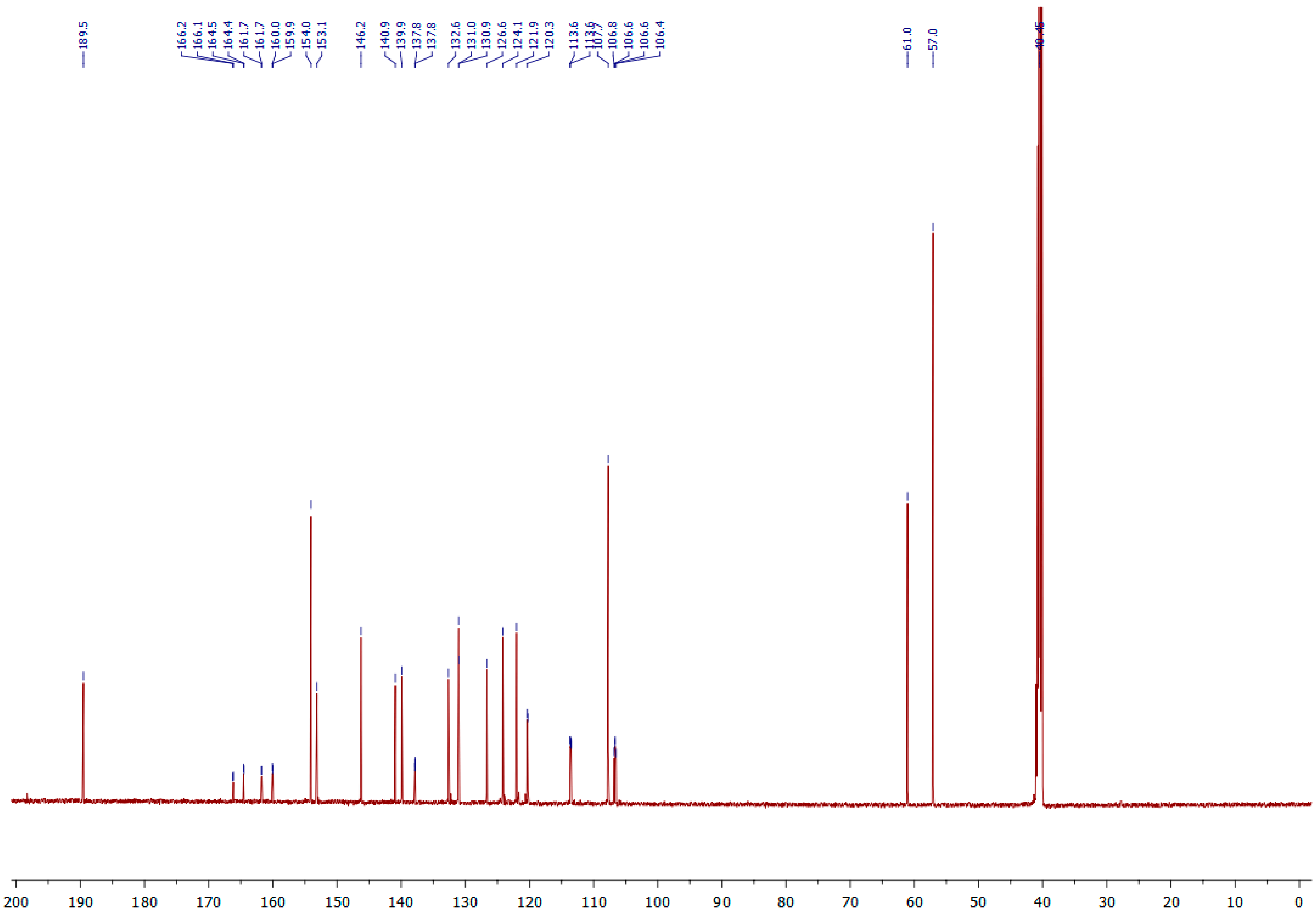
189.5
166.2
166.1
164.5
164.4
161.7
161.7
160.0
159.9
154.0
153.1
146.2
140.9
139.9
137.8
137.8
132.6
131.0
130.9
126.6
124.1
121.9
120.3
113.6
113.6
107.7
106.8
106.6
106.6
106.4
61.0
57.0
200
190
180
170
160
150
140
130
120
110
100
90
80
70
60
50
40
30
20
10
0

^{1}H NMR (600MHz, DMSO-d6): δ (ppm): 8.59 (1H, t, $J_{H2',H4',6'}$ = 1.8 Hz, $H_{2'}$), 8.43 (1H, dt, $J_{H5',H6'}$ = 7.8 Hz, $J_{H4',H6'}$ = 1.8 Hz, $H_{6'}$), 8.16 (1H, ddd, $J_{H4',H5'}$ = 7.8 Hz, $H_{4'}$), 8.00 (1H, AB spin system, d, $J_{H7,H8}$ = 15.6 Hz, H_7), 7.91 (1H, dt, $J_{H11',H12'}$ = 9 Hz, $J_{H12',F}$ = 6.6 Hz, $H_{12'}$), 7.85 (1H, t, $H_{5'}$), 7.81 (1H, AB spin system, d, H_8), 7.64 (1H, ddd, $J_{H11',F}$ = 9 Hz, $J_{H11',H9'}$ = 2.6 Hz), 7.32 (1H, td, $J_{H9',F}$ = 8.4 Hz, $H_{9'}$), 7.31 (2H, s, H_2), 3.91 (6H, s, OCH_3), 3.77 (3H, s, OCH_3).
^{13}C NMR (150MHz, DMSO-d6): δ (ppm): 189.5, 165.3 (dd, J = 250.5 Hz, J = 10.5 Hz), 160.8 (dd, J = 258 Hz, J = 13.5 Hz), 154.0, 153.1, 146.2, 140.9, 139.9, 137.8 (dd, J = 6 Hz, J = 3 Hz), 132.6, 131.0, 130.9, 126.6, 124.1, 121.9, 120.2 (d, J = 10.5 Hz), 113.5 (dd, J = 24 Hz, J = 4.5 Hz), 107.7, 106.6 (dd, J = 25.5 Hz, J = 24 Hz), 61.0, 57.0.
HRMS (ESI): $C_{24}H_{20}F_2N_2O_4$+H, calculated m/z 439.14639; found m/z 439.14627.

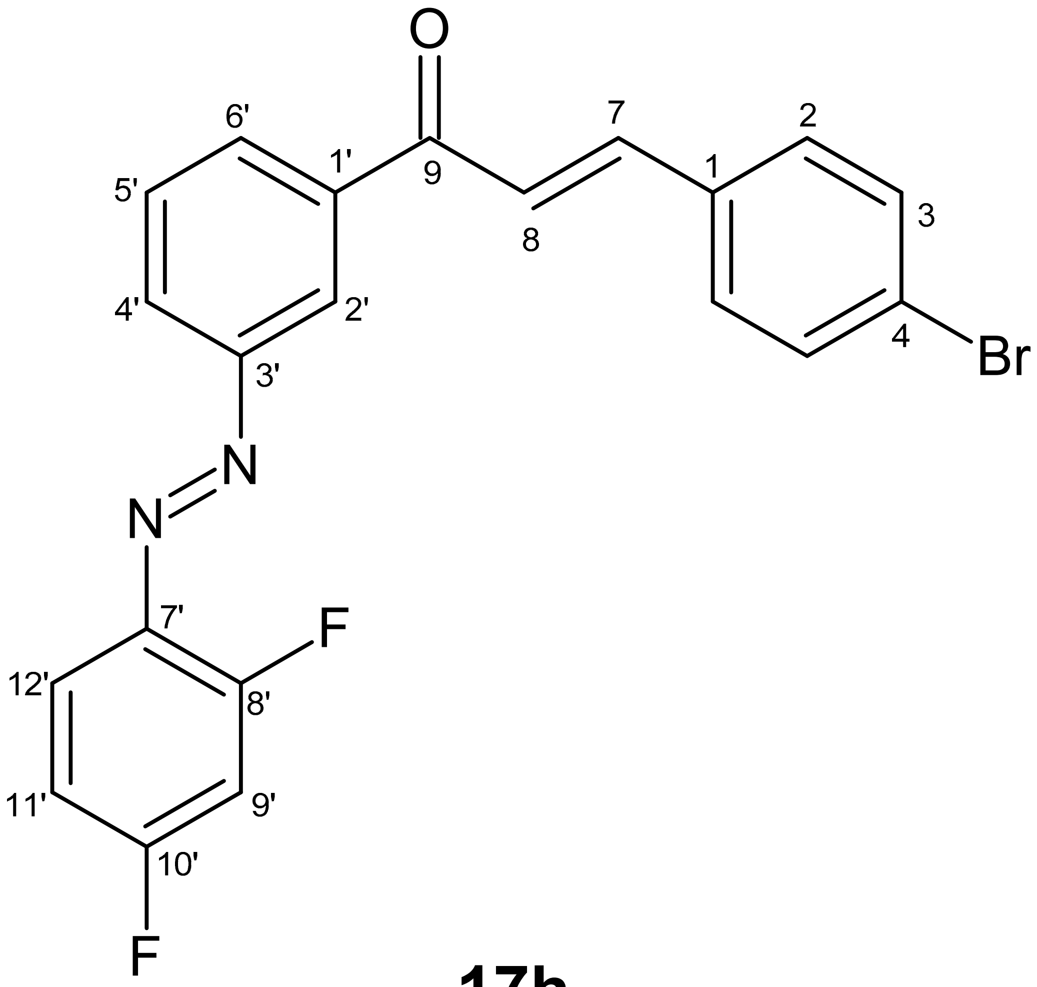

17h

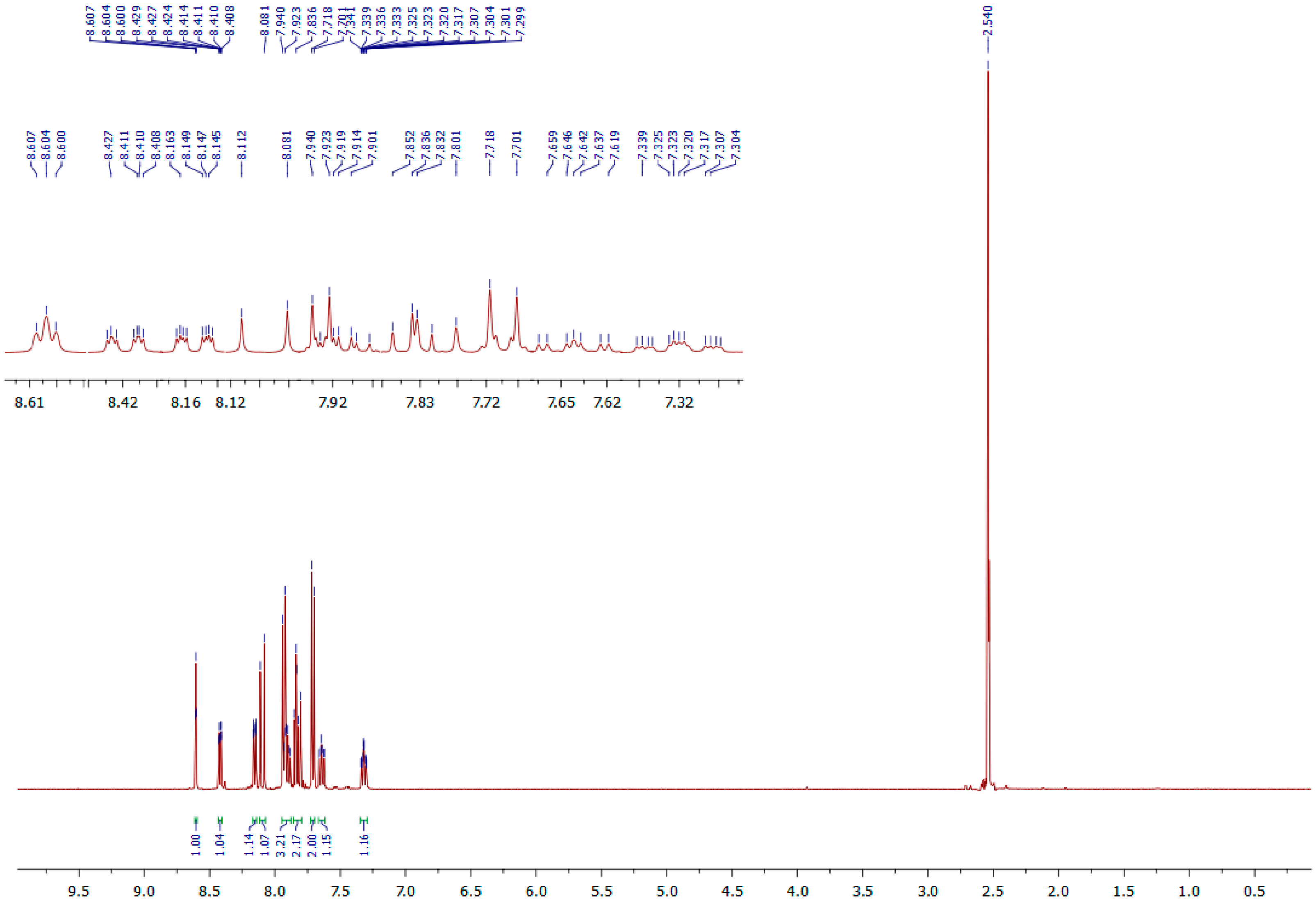

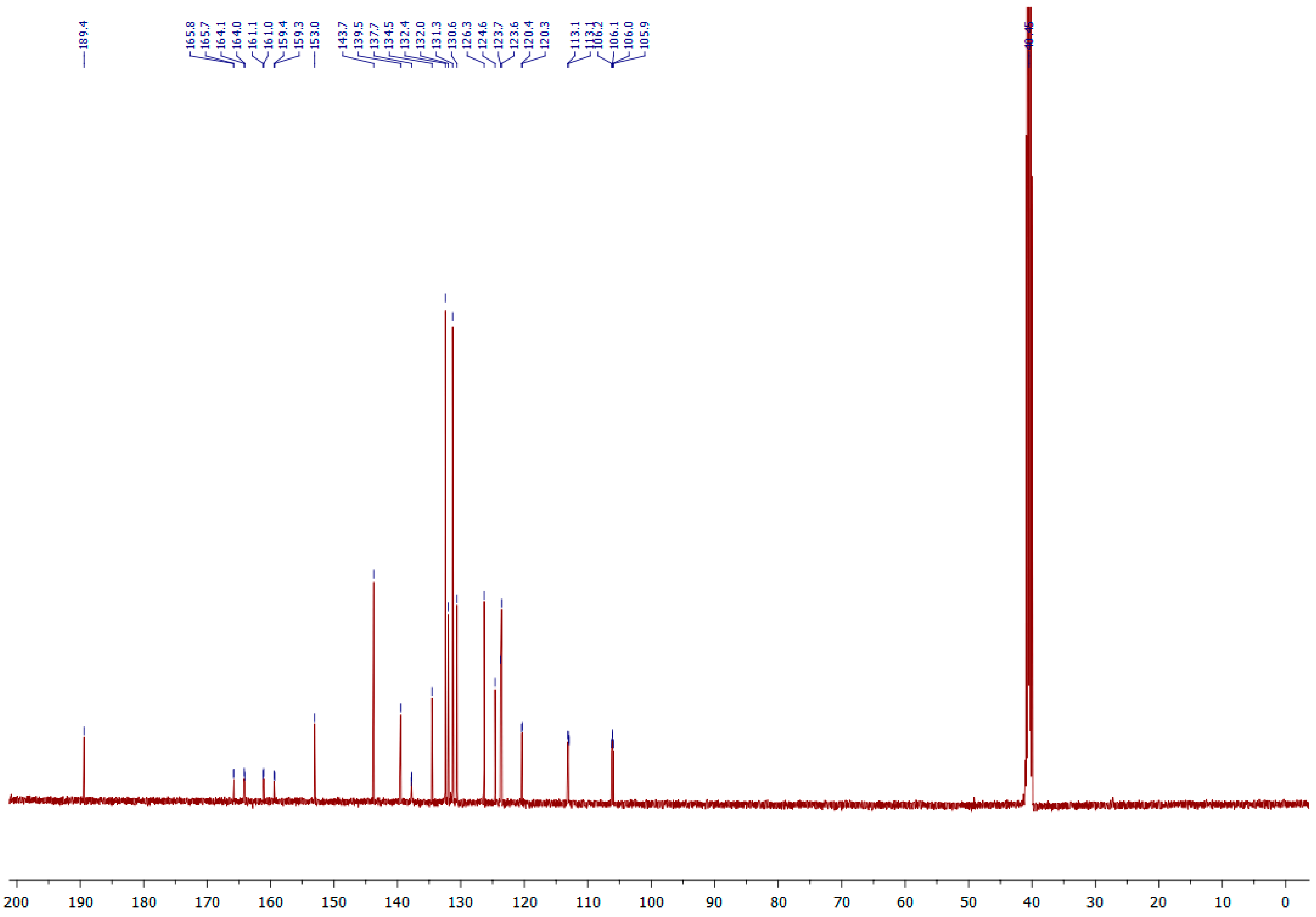

^{1}H NMR (500MHz, DMSO-d6): δ (ppm): 8.60 (1H, t, $J_{H2',H4',6'}$ = 2 Hz, $H_{2'}$), 8.42 (1H, dt, $J_{H5',H6'}$ = 8 Hz, $J_{H4',H6'}$ = 1.5 Hz, $H_{6'}$), 8.15 (1H, ddd, $J_{H4',H5'}$ = 8 Hz, $H_{4'}$), 8.10 (1H, AB spin system, d, $J_{H7,H8}$ = 15.5 Hz, H_7), 7.93 (2H, d, $J_{H2,H3}$ = 8.5 Hz, H_3), 7.91 (1H, dt, $J_{H11',H12'}$ = 8.5 Hz, $J_{H12',F}$ = 6 Hz, $H_{12'}$), 7.84 (1H, t, $H_{5'}$), 7.82 (1H, AB spin system, d, H_8), 7.71 (2H, d, H_2), 7.64 (1H, ddd, $J_{H11',F}$ = 9.5 Hz, $J_{H11',H9'}$ = 3 Hz), 7.32 (1H, tdd, $J_{H9',F}$ = 8 Hz, $H_{9'}$).
^{13}C NMR (150MHz, DMSO-d6): δ (ppm): 189.4, 164.9 (dd, J = 250.5 Hz, J = 12 Hz), 160.2 (dd, J = 258 Hz, J = 12 Hz), 153.0, 143.7, 139.51, 137.7 (dd, J = 7.5 Hz, J = 4.5 Hz), 134.5, 132.4, 132.0, 131.3, 130.6, 126.3, 124.6, 123.7, 123.6, 120.3 (d, J = 10.5 Hz), 113.0 (dd, J = 22.5 Hz, J = 3 Hz), 106.1 (dd, J = 27 Hz, J = 24 Hz).
HRMS (ESI): $C_{21}H_{13}BrF_2N_2O$+H, measured m/z 407.02521; found m/z 407.02526.

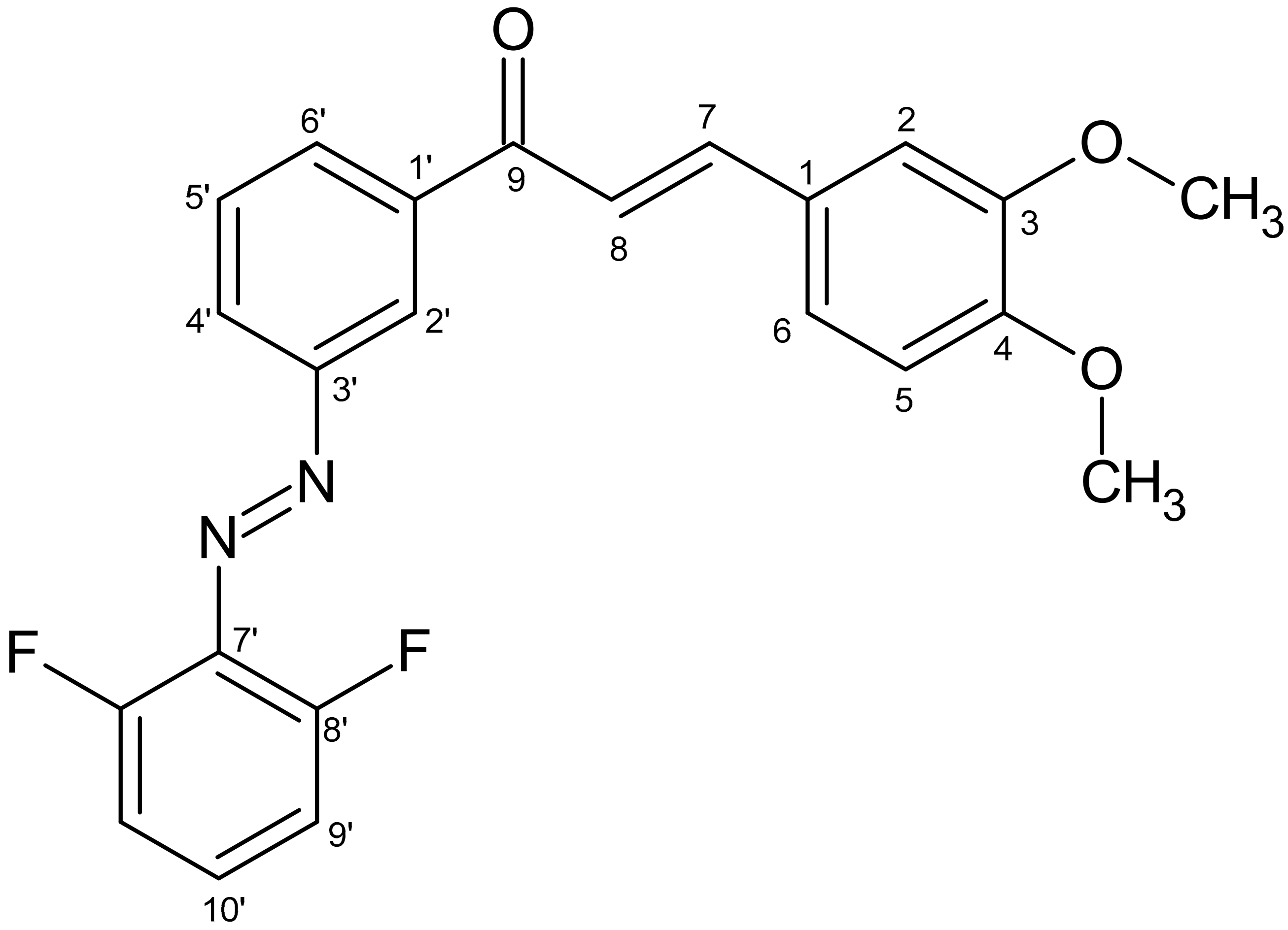

17i

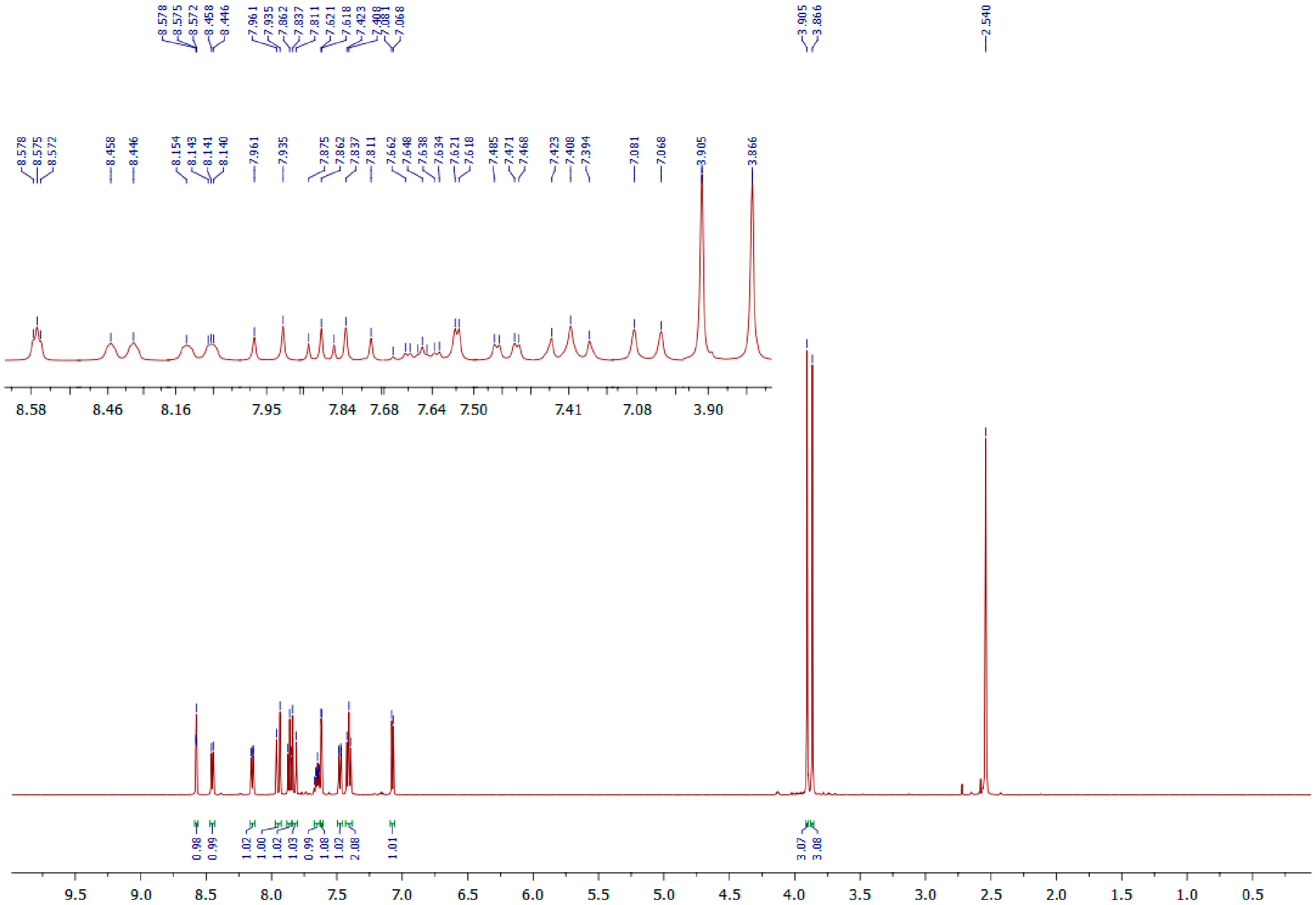
8.578
8.575
8.572
8.458
8.446
8.154
8.143
8.141
8.140
7.961
7.935
7.875
7.862
7.837
7.811
7.662
7.648
7.638
7.634
7.621
7.618
7.485
7.471
7.468
7.423
7.408
7.394
7.081
7.068
3.905
3.866
2.540
0.98
0.99
1.02
1.00
1.02
1.03
0.99
1.08
1.02
2.08
1.01
3.07
3.08
9.5
9.0
8.5
8.0
7.5
7.0
6.5
6.0
5.5
5.0
4.5
4.0
3.5
3.0
2.5
2.0
1.5
1.0
0.5

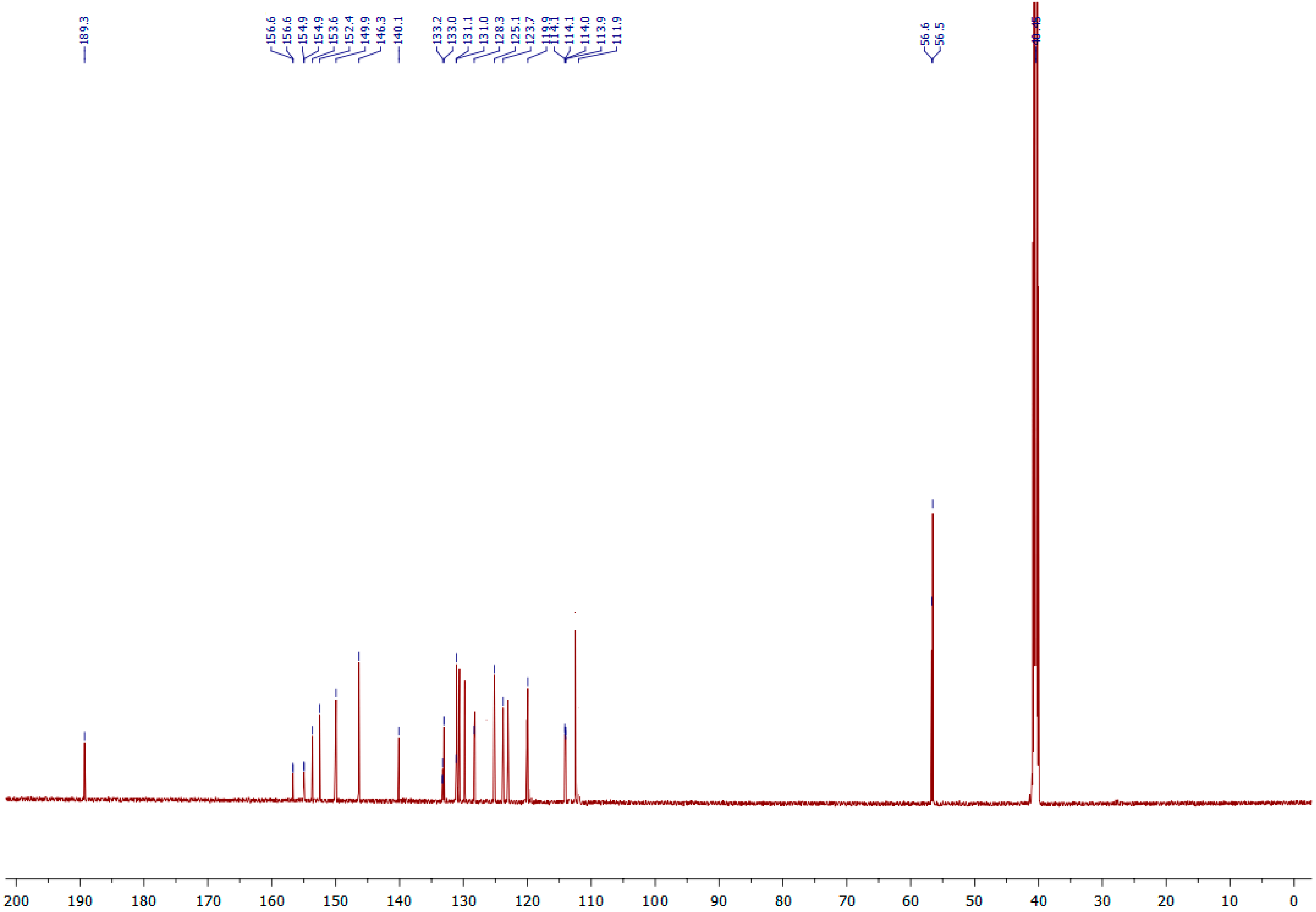
189.3
156.6
156.6
154.9
154.9
153.6
152.4
149.9
146.3
140.1
133.2
133.0
131.1
131.0
128.3
125.1
123.7
119.9
114.1
114.1
114.0
113.9
111.9
56.6
56.5
200
190
180
170
160
150
140
130
120
110
100
90
80
70
60
50
40
30
20
10
0

^{1}H NMR (600MHz, DMSO-d6): δ (ppm): 8.58 (1H, t, $J_{H2',H4',6'}$ = 1.8 Hz, $H_{2'}$), 8.45 (1H, dt, $J_{H5',H6'}$ = 7.2 Hz, $J_{H4',H6'}$ = 1.8 Hz, $H_{6'}$), 8.15 (1H, ddd, $J_{H4',H5'}$ = 7.8 Hz, $H_{4'}$), 7.95 (1H, AB spin system, d, $J_{H7,H8}$ = 15.6 Hz, H_7), 7.86 (1H, t, $H_{5'}$), 7.82 (1H, AB spin system, d, H_8), 7.65 (1H, tt, $J_{H9',H10'}$ = 9.0 Hz, $J_{,H10',F}$ = 6.0 Hz, $H_{10'}$), 7.62 (1H, d, $J_{H2,H6}$ = 1.8 Hz, H_2), 7.48 (1H, dd, $J_{H5,H6}$ = 8.4 Hz, H_6), 7.41 (2H, t, $J_{H9',F}$= 9 Hz, $H_{9'}$), 7.07 (1H, d, H_5), 3.91 (3H, s, OCH_3), 3.87 (3H, s, OCH_3).
^{13}C NMR (150MHz, DMSO-d6): δ (ppm): 189.3, 155.8 (dd, J = 256.5 Hz, J = 4.5 Hz), 153.6, 152.4, 149.9, 146.3, 140.1, 133.2 (t, J = 10.5 Hz), 133.0, 131.1 (t, J = 10 Hz), 131.0, 128.3, 125.1, 123.7, 119.9, 114.0, (dd, J = 19.5 Hz, J = 3 Hz), 111.9, 56.6, 56.5.
HRMS (ESI): $C_{23}H_{18}F_2N_2O_3$+H, calculated m/z 409.13583; found m/z 409.13565.

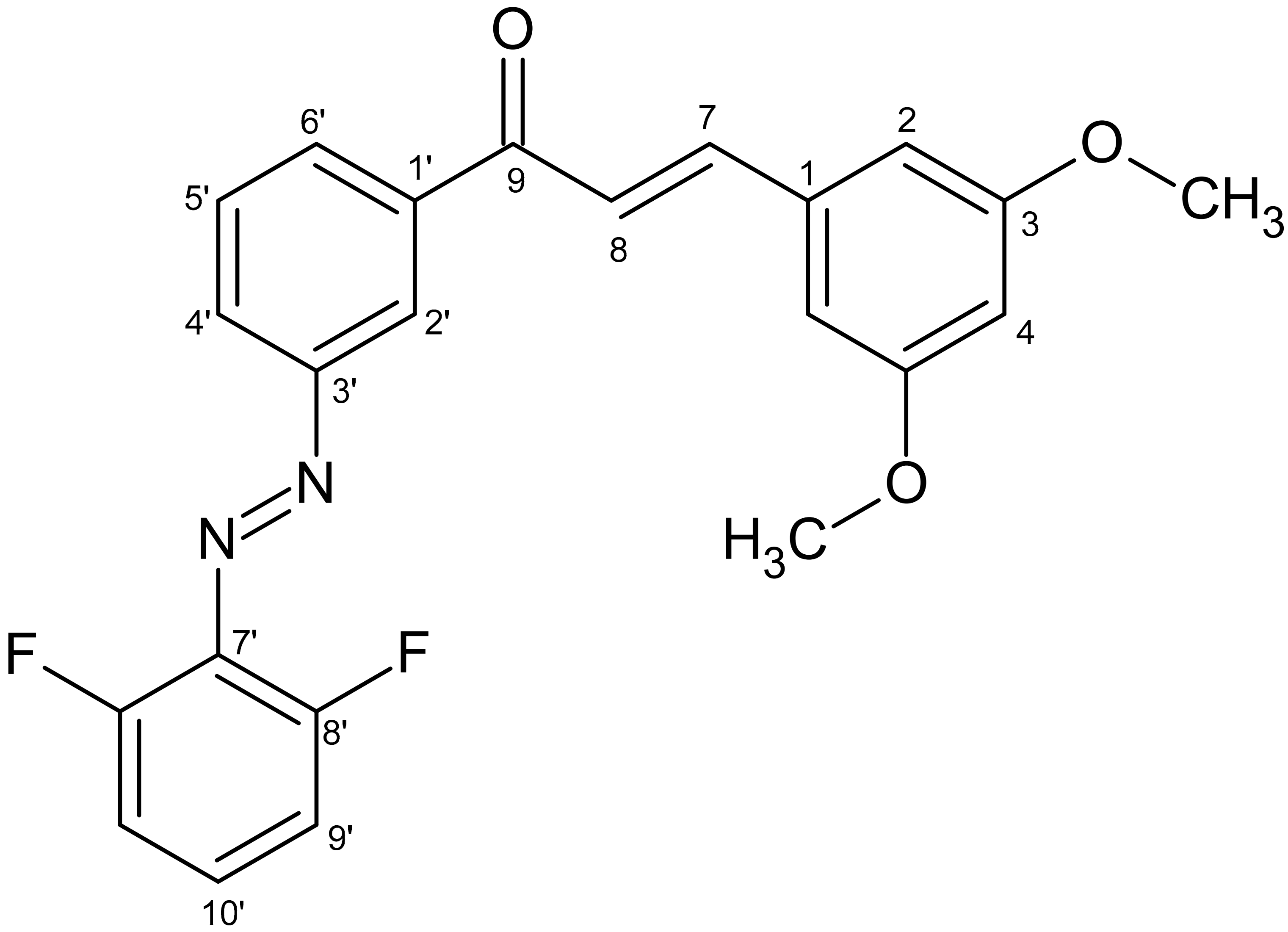

17j

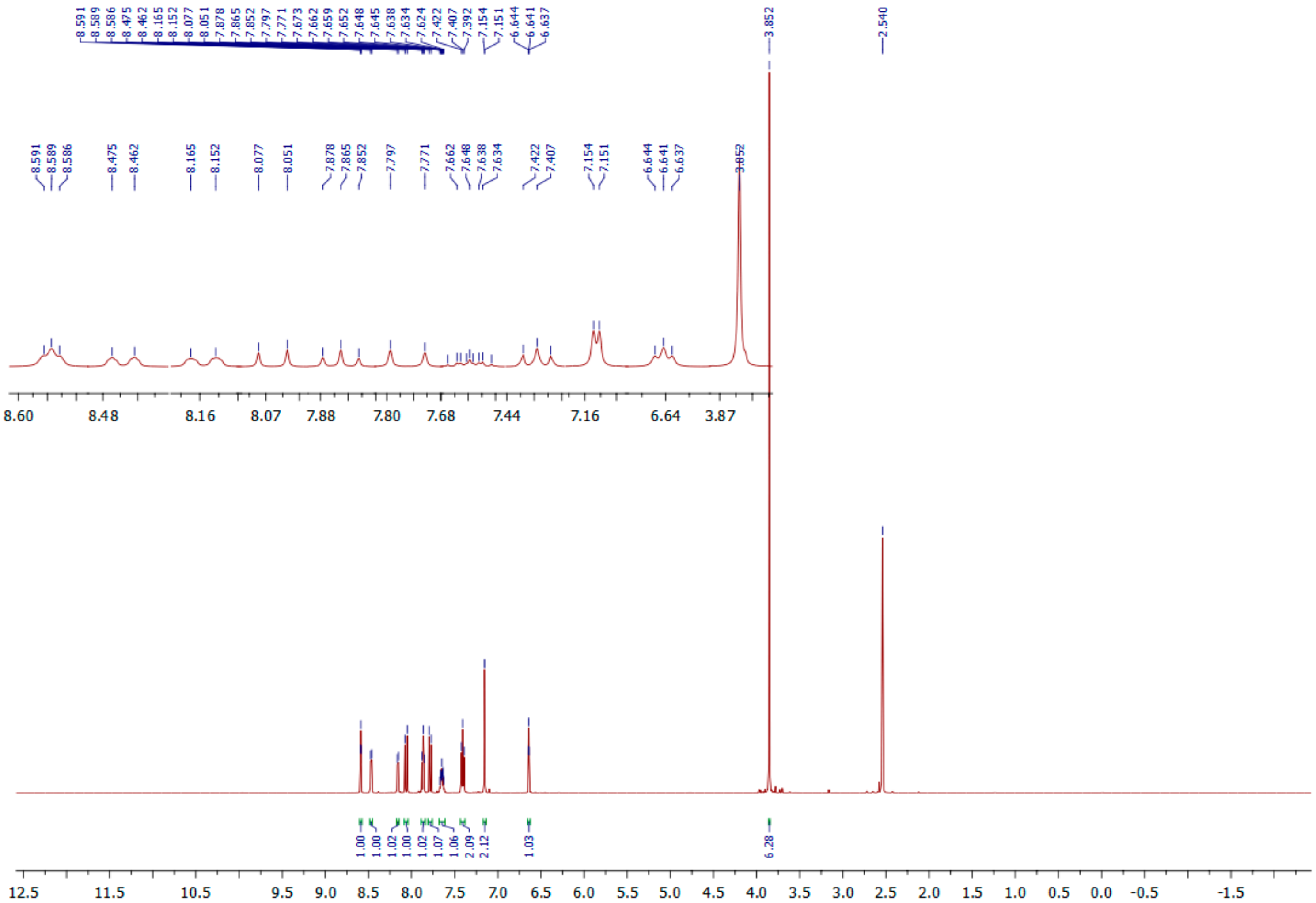

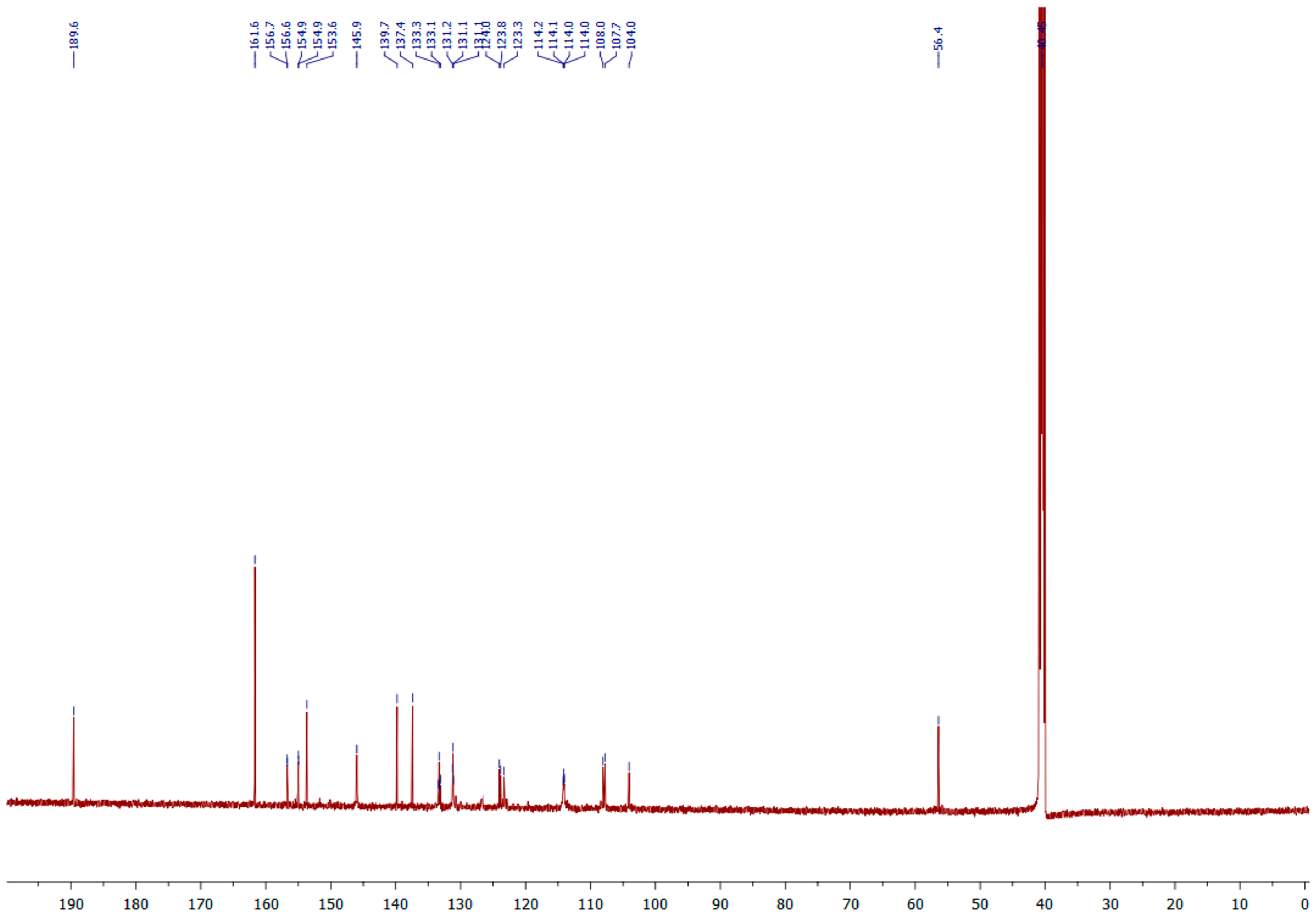
189.6
161.6
156.7
156.6
154.9
154.9
153.6
145.9
139.7
137.4
133.3
133.1
131.2
131.1
131.1
124.0
123.8
123.3
114.2
114.1
114.0
114.0
108.0
107.7
104.0
56.4
190
180
170
160
150
140
130
120
110
100
90
80
70
60
50
40
30
20
10
0

^{1}H NMR (600MHz, DMSO-d6): δ (ppm): 8.59 (1H, t, $J_{H2',H4',6'}$ = 1.8 Hz, $H_{2'}$), 8.47 (1H, dt, $J_{H5',H6'}$ = 7.8 Hz, $J_{H4',H6'}$ = 1.8 Hz, $H_{6'}$), 8.16 (1H, ddd, $J_{H4',H5'}$ = 7.8 Hz, $H_{4'}$), 8.06 (1H, AB spin system, d, $J_{H7,H8}$ = 15.6 Hz, H_7), 7.87 (1H, t, $H_{5'}$), 7.78 (1H, AB spin system, d, H_8), 7.65 (1H, tt, $J_{H9',H10'}$ = 9.0 Hz, $J_{H10',F}$ = 6.0 Hz, $H_{10'}$), 7.41 (2H, t, $J_{H9',F}$= 9 Hz, $H_{9'}$), 7.15 (2H, d, $J_{H2,H4}$ = 1.8 Hz, H_2), 6.64 (1H, t, H_4), 3.85 (6H, s, OCH_3).
^{13}C NMR (150MHz, DMSO-d6): δ (ppm): 189.6, 161.6, 155.2 (dd, J_1 = 256.5 Hz, J_2 = 4.5 Hz), 153.6, 145.9, 139.7, 137.4, 133.3 (t, J = 10.5 Hz), 133.1, 131.1 (t, J = 10.5 Hz), 124.0, 123.8, 123.3, 114.1 (dd, J = 12 Hz), 108.0, 107.7, 104.0, 56.4.
HRMS (ESI): $C_{23}H_{18}F_2N_2O_3$+H, calculated m/z 409.13583; found m/z 409.13577.

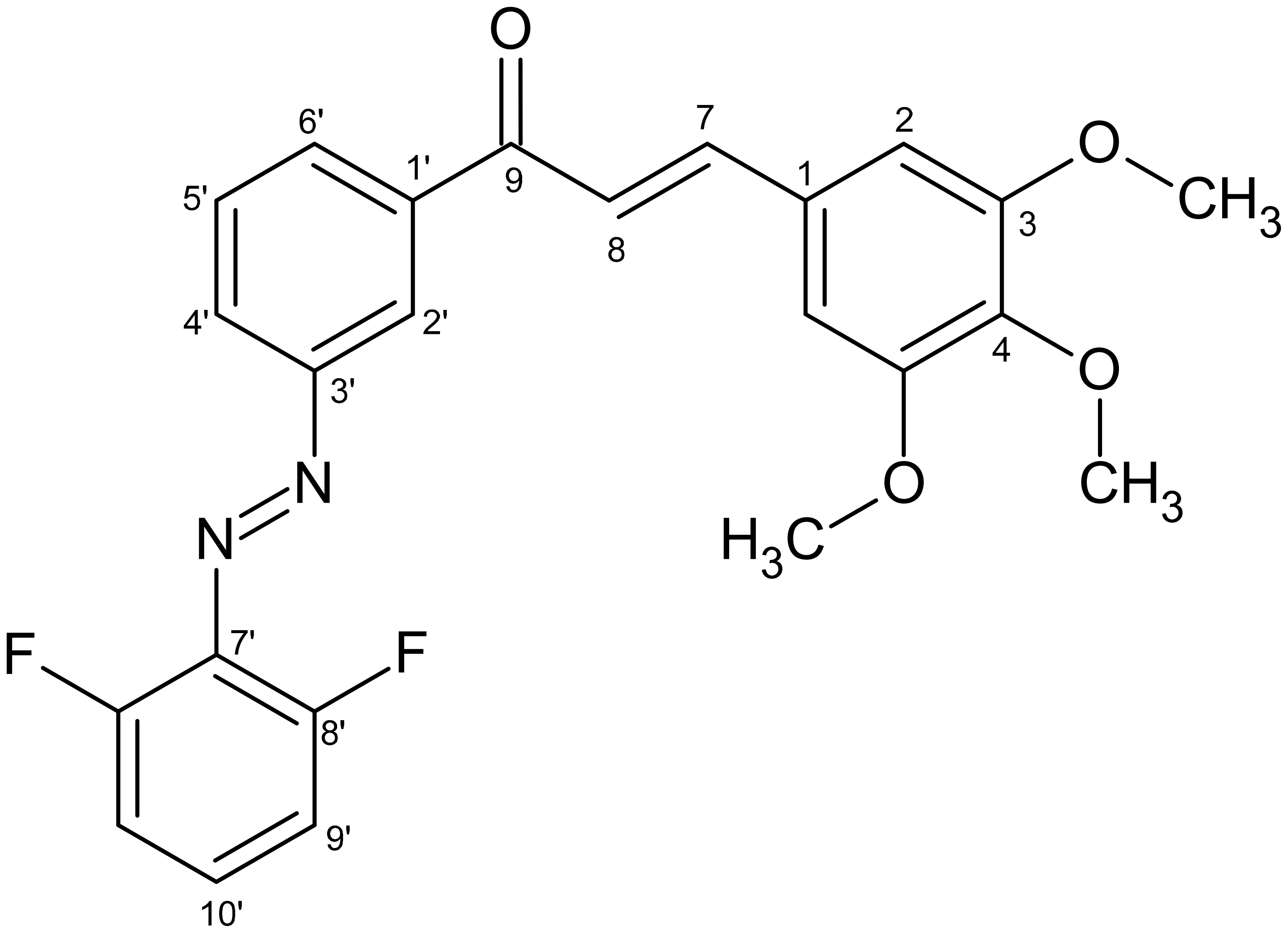

17k

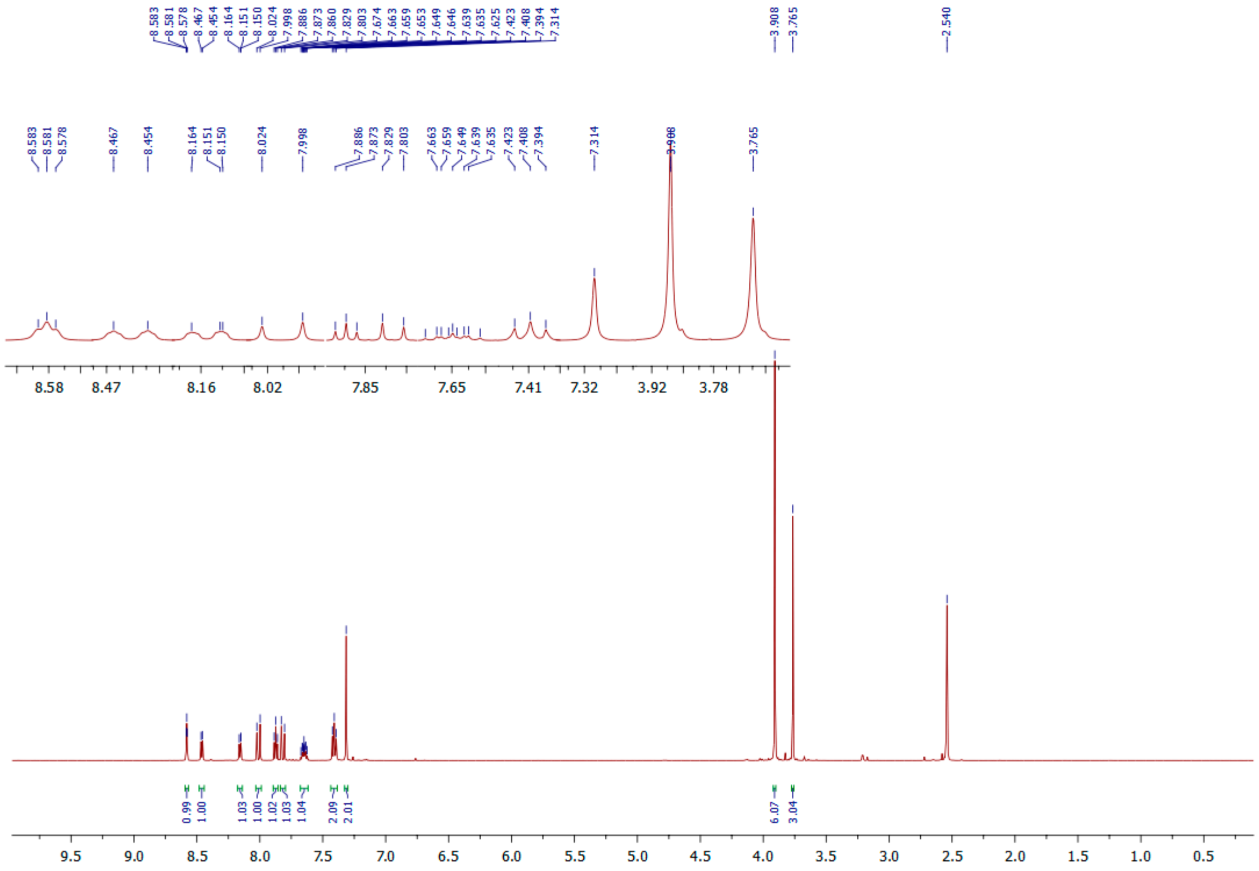

^{1}H NMR (600MHz, DMSO-d6): δ (ppm): 8.58 (1H, t, $J_{H2',H4',6'}$ = 1.8 Hz, $H_{2'}$), 8.46 (1H, dt, $J_{H5',H6'}$ = 7.8 Hz, $J_{H4',H6'}$ = 1.8 Hz, $H_{6'}$), 8.16 (1H, ddd, $J_{H4',H5'}$ = 7.8 Hz, $H_{4'}$), 8.01 (1H, AB spin system, d, $J_{H7,H8}$ = 15.6 Hz, H_{7}), 7.87 (1H, t, $H_{5'}$), 7.82 (1H, AB spin system, d, H_{8}), 7.65 (1H, tt, $J_{H9',H10'}$ = 9.0 Hz, $J_{H10',F}$ = 6.0 Hz, $H_{10'}$), 7.41 (2H, t, $J_{H9',F}$ = 9 Hz, $H_{9'}$), 7.31 (2H, s, H_{2}), 3.91 (6H, s, OCH_3), 3.77 (3H, s, OCH_3).
^{13}C NMR (150MHz, DMSO-d6): δ (ppm): 189.3, 155.8 (dd, J = 256.5 Hz, J = 4.5 Hz), 153.6, 152.4, 149.9, 146.3, 140.1, 133.2 (t, J = 10.5 Hz), 133.0, 131.1 (t, J = 10 Hz), 131.0, 128.3, 125.1, 123.7, 119.9, 114.0, (dd, J = 19.5 Hz, J = 3 Hz), 111.9, 56.6, 56.5.

HRMS (ESI): $C_{24}H_{20}F_2N_2O_4$+H, calculated m/z 439.14639; found m/z 439.14637.

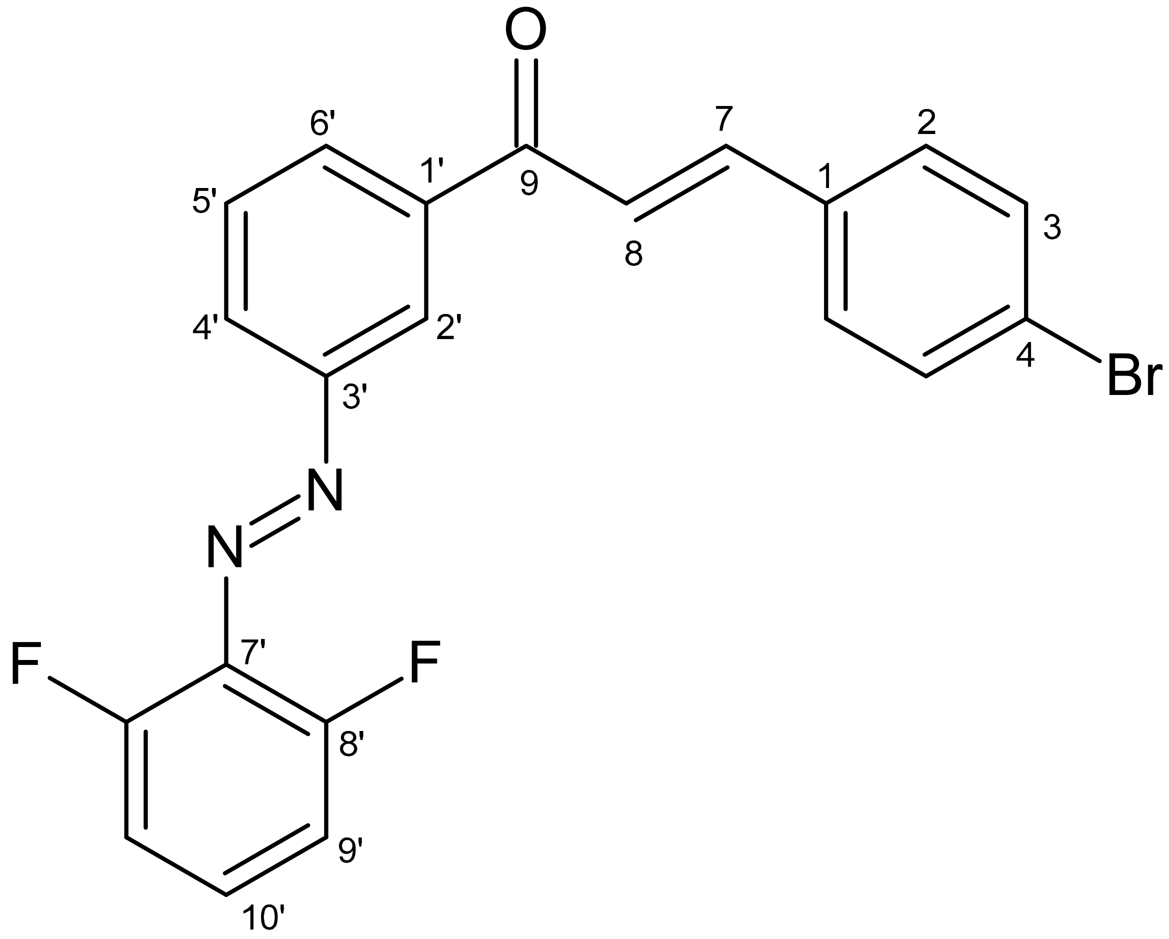

17l

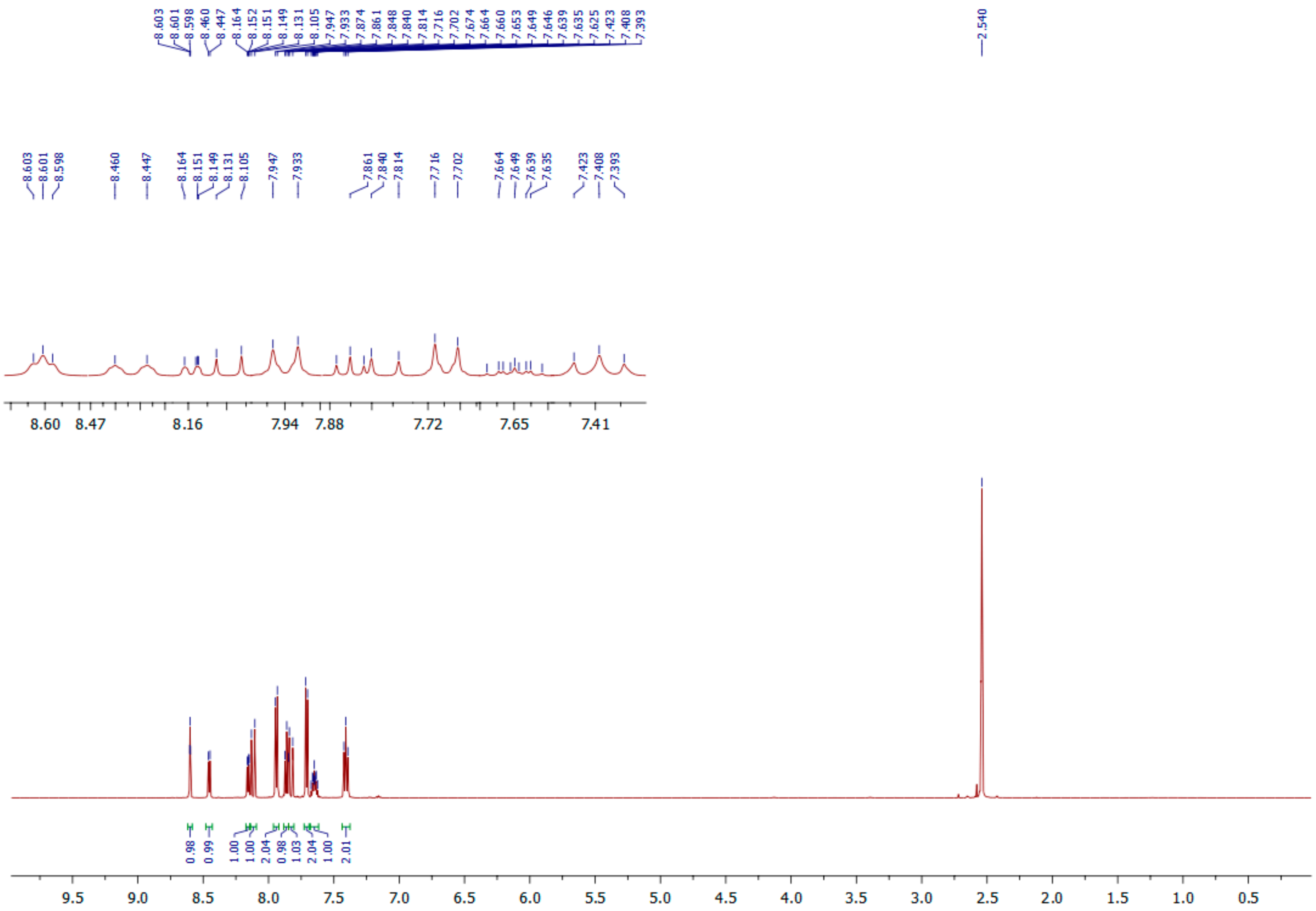
8.603
8.601
8.598
8.460
8.447
8.164
8.152
8.151
8.149
8.131
8.105
7.947
7.933
7.874
7.861
7.848
7.840
7.814
7.716
7.702
7.674
7.664
7.660
7.653
7.649
7.646
7.639
7.635
7.625
7.423
7.408
7.393
2.540
8.60
8.47
8.16
7.94
7.88
7.72
7.65
7.41
0.98
0.99
1.00
1.00
2.04
0.98
1.03
2.04
1.00
2.01
9.5
9.0
8.5
8.0
7.5
7.0
6.5
6.0
5.5
5.0
4.5
4.0
3.5
3.0
2.5
2.0
1.5
1.0
0.5

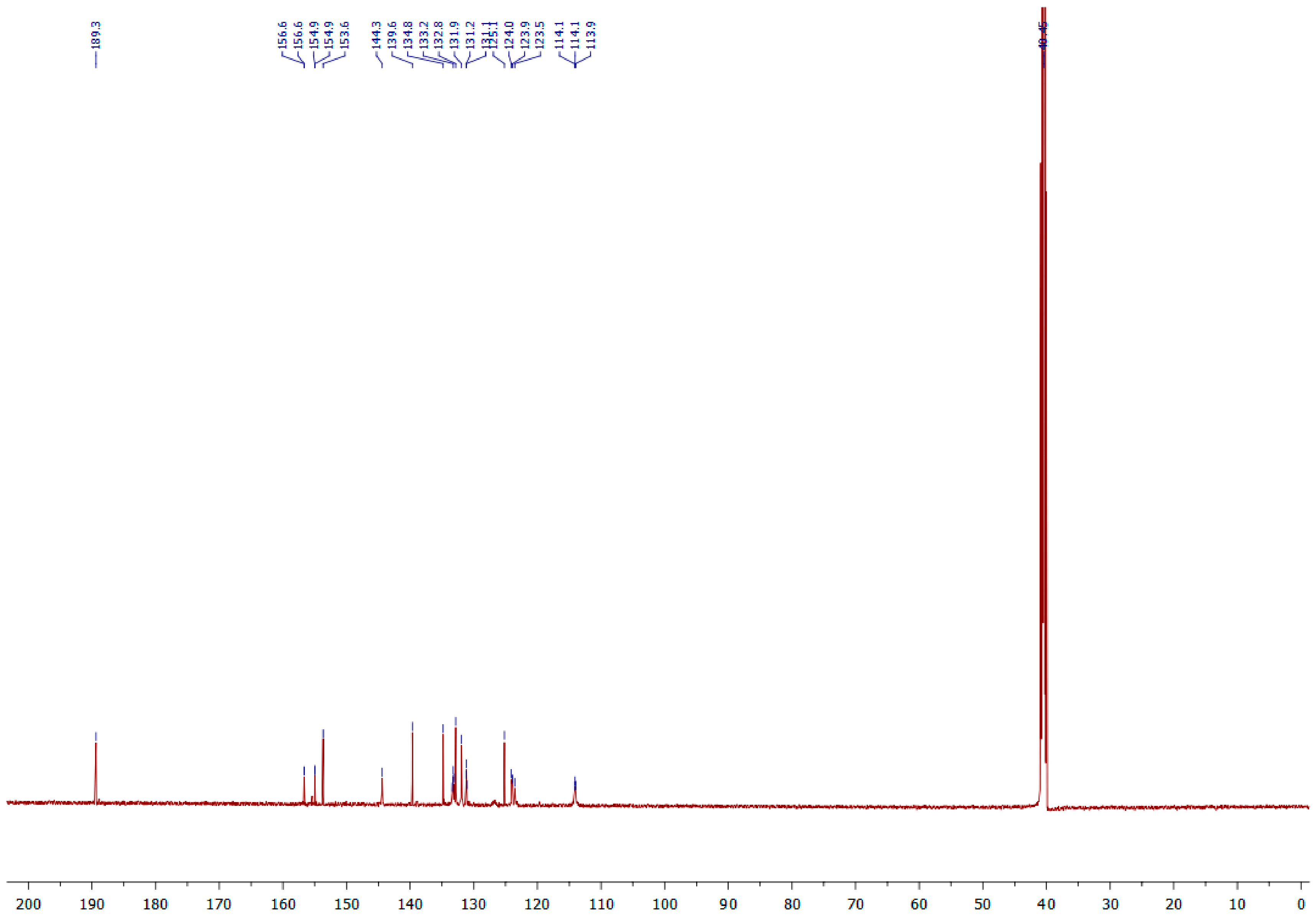
189.3
156.6
156.6
154.9
154.9
153.6
144.3
139.6
134.8
133.2
132.8
131.9
131.2
131.1
125.1
124.0
123.9
123.5
114.1
114.1
113.9
200
190
180
170
160
150
140
130
120
110
100
90
80
70
60
50
40
30
20
10
0

^{1}H NMR (600MHz, DMSO-d6): δ (ppm): 8.60 (1H, t, $J_{H2',H4',6'}$ = 1.8 Hz, $H_{2'}$), 8.45 (1H, dt, $J_{H5',H6'}$ = 7.8 Hz, $J_{H4',H6'}$ = 1.8 Hz, $H_{6'}$), 8.16 (1H, ddd, $J_{H4',H5'}$ = 7.8 Hz, $H_{4'}$), 8.12 (1H, AB spin system, d, $J_{H7,H8}$ = 15.6 Hz, H_7), 7.94 (2H, d, $J_{H2,H3}$ = 8.4 Hz, H_3), 7.86 (1H, t, $H_{5'}$), 7.83 (1H, AB spin system, d, H_8), 7.71 (2H, d, H_2), 7.65 (1H, tt, $J_{H9',H10'}$ = 9.0 Hz, $J_{H10',F}$ = 6.0 Hz, $H_{10'}$), 7.41 (2H, t, $J_{H9',F}$ = 9 Hz, $H_{9'}$).
^{13}C NMR (150MHz, DMSO-d6): δ (ppm): 189.3, 155.8 (dd, J_1 = 256.5 Hz, J_2 = 4.5 Hz), 153.6, 144.3, 139.6, 134.8, 133.3 (t, J = 10.5 Hz), 133.2, 133.0, 132.8, 131.9, 131.1 (t, J = 10.5 Hz), 125.1, 124.0, 123.9, 123.5, 114.1 (dd, J = 12 Hz).
HRMS (ESI): $C_{21}H_{13}BrF_2N_2O$+H, calculated m/z 407.02521; found m/z 407.02520.

4. Photochemical studies

4.1 Illumination setup for light-dependent assays

Based on described data [7-9], for illumination of samples, we decided to use self-built arrays of 24 low-power light-emitting diodes (LEDs) controlled by an Adurino board. Such an automated system allowed precise pulsed illumination during long-term assays. Most importantly, it has been proven to be compatible with cell-culturing conditions. The proposed system consisted of the Arduino microcomputer to drive an 8-relay module, which turns on and off sets of LED arrays so that they flash with identical timings. The following wavelengths of LEDs were used in this study (in nm): 390, 400, 430, 470, 505, 515, 525, 535, 590, and 610 nm. All LEDs were bought from Mouser Electronics, Inc., and the corresponding Mouser part numbers are 749-UV5TZ-390-30, 749-UV5TZ-400-15, 749-5BWC, 630-HLMP-CB3A-UV0DD, 630-HLMP-CE34-Y1CDD, 859-LTL2V3TCYK2, 630-HLMP-CM3A-Z10DD, 859-LTL2V3TGX3KS, and 630-HLMP-EL1A-Z1KDD, respectively. Only one wavelength of LED was used per array, and arrays at different wavelengths were kept in separate cardboard boxes. We consider this crucial since with standard, clear well plates, light scattering from one well across the whole plate was often observed to be significant enough to compromise results.

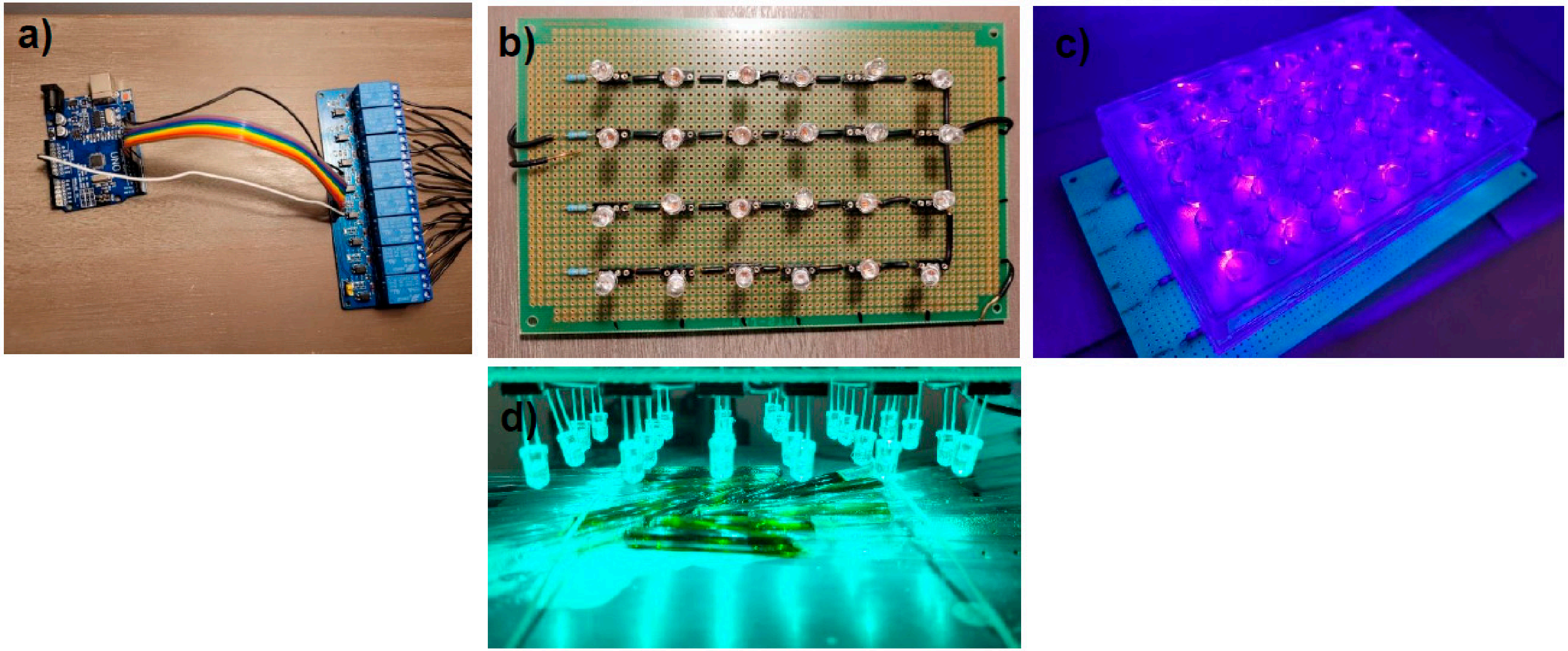
a)
b)
c)
d)

4.2 Photostationary state (PSS) analysis

Photostationary state (PSS) analysis compositions for chalcones (**17a-17c**, **17e-17g**, **17i-17l**) determined by ^{19}F NMR analysis (c ≈ 100 mM in DMSO-d_6):

**Wavelength [nm]**	**Percentage of isomer *Z* [%] Compound**									
	**17a**	**17b**	**17c**	**17e**	**17f**	**17g**	**17i**	**17j**	**17k**	**17l**
390 nm	24.94	15.28	20.75	16.18	16.05	17.75	24.02	16.39	21.68	15.46
400 nm	25.92	17.33	22.06	22.41	16.75	20.2	25.04	16.51	21.51	16.55
430 nm	20.28	12.11	14.3	11.94	15.7	21.74	20.47	19.78	20.88	12.27
470 nm	28.04	22.89	26.96	37.12	21.39	36.2	53.33	49.38	52.56	53.26
505 nm	32.44	32.38	30.59	46.84	47.13	44.82	71.32	68.87	71.35	71.88
525 nm	29.47	27.08	29.75	45.56	45.85	43.41	74.84	73.2	74.39	75.44
535 nm	27.72	25.4	28.75	45.63	41.18	44.75	73.53	72.24	73.94	74.61
590 nm	1.02	0.02	0.62	3.99	0.36	3.82	28.97	9.82	42.8	52.99
610 nm	0.69	0	0	0	0	0	9.88	10.49	23.81	16.98

17a:

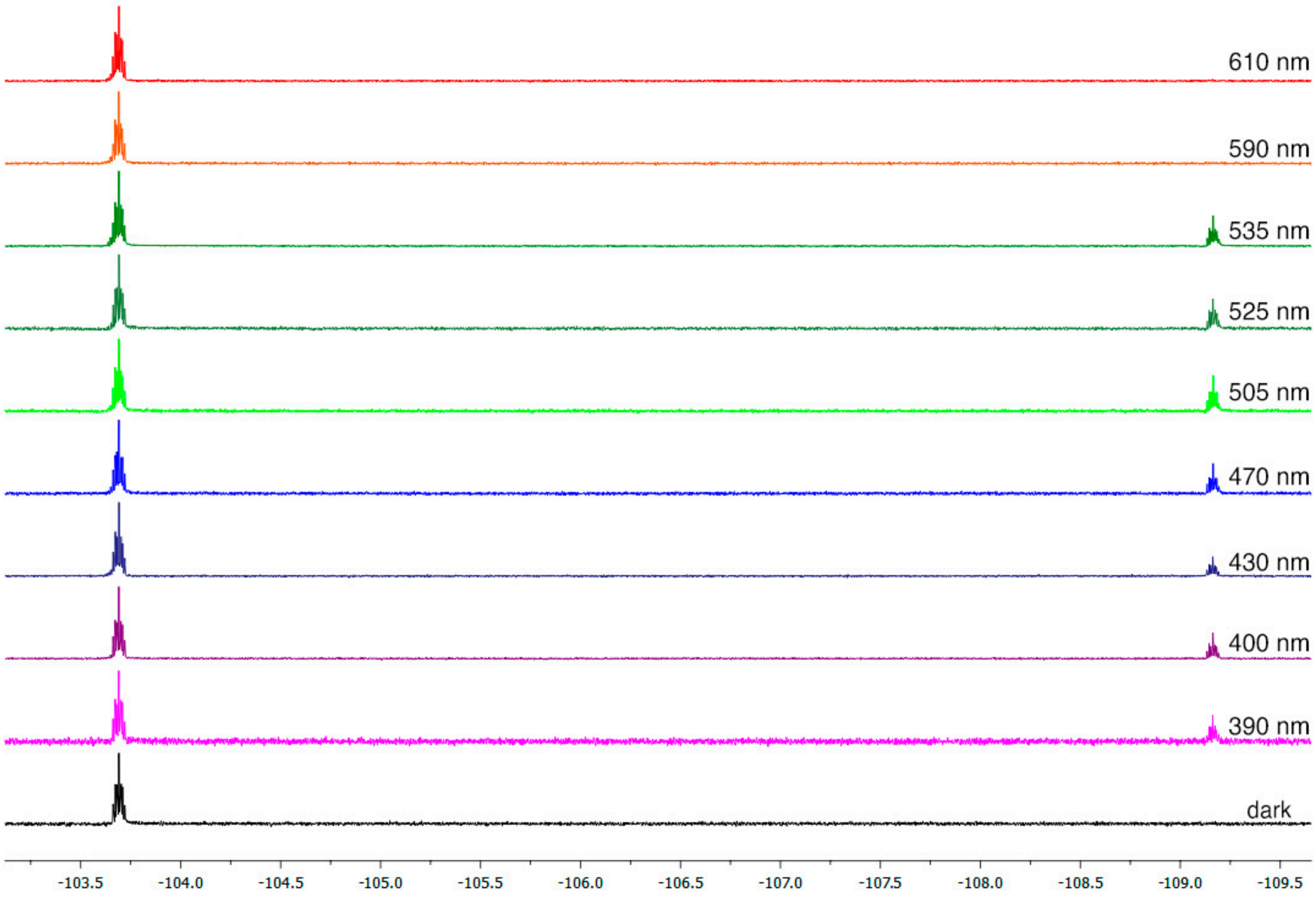

610 nm
590 nm
535 nm
525 nm
505 nm
470 nm
430 nm
400 nm
390 nm
dark
-103.5
-104.0
-104.5
-105.0
-105.5
-106.0
-106.5
-107.0
-107.5
-108.0
-108.5
-109.0
-109.5

17b:

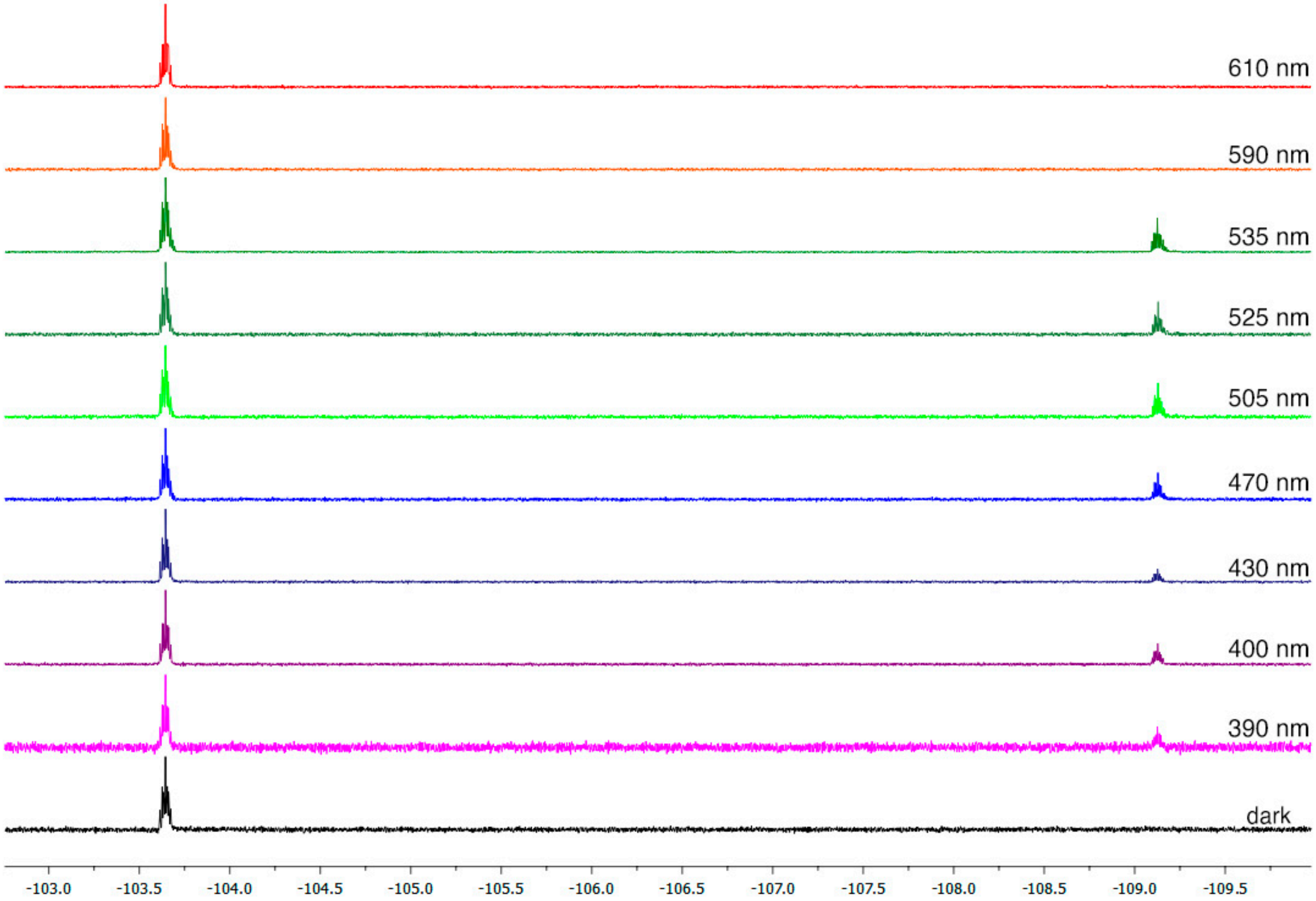
610 nm
590 nm
535 nm
525 nm
505 nm
470 nm
430 nm
400 nm
390 nm
dark
-103.0
-103.5
-104.0
-104.5
-105.0
-105.5
-106.0
-106.5
-107.0
-107.5
-108.0
-108.5
-109.0
-109.5

17c:

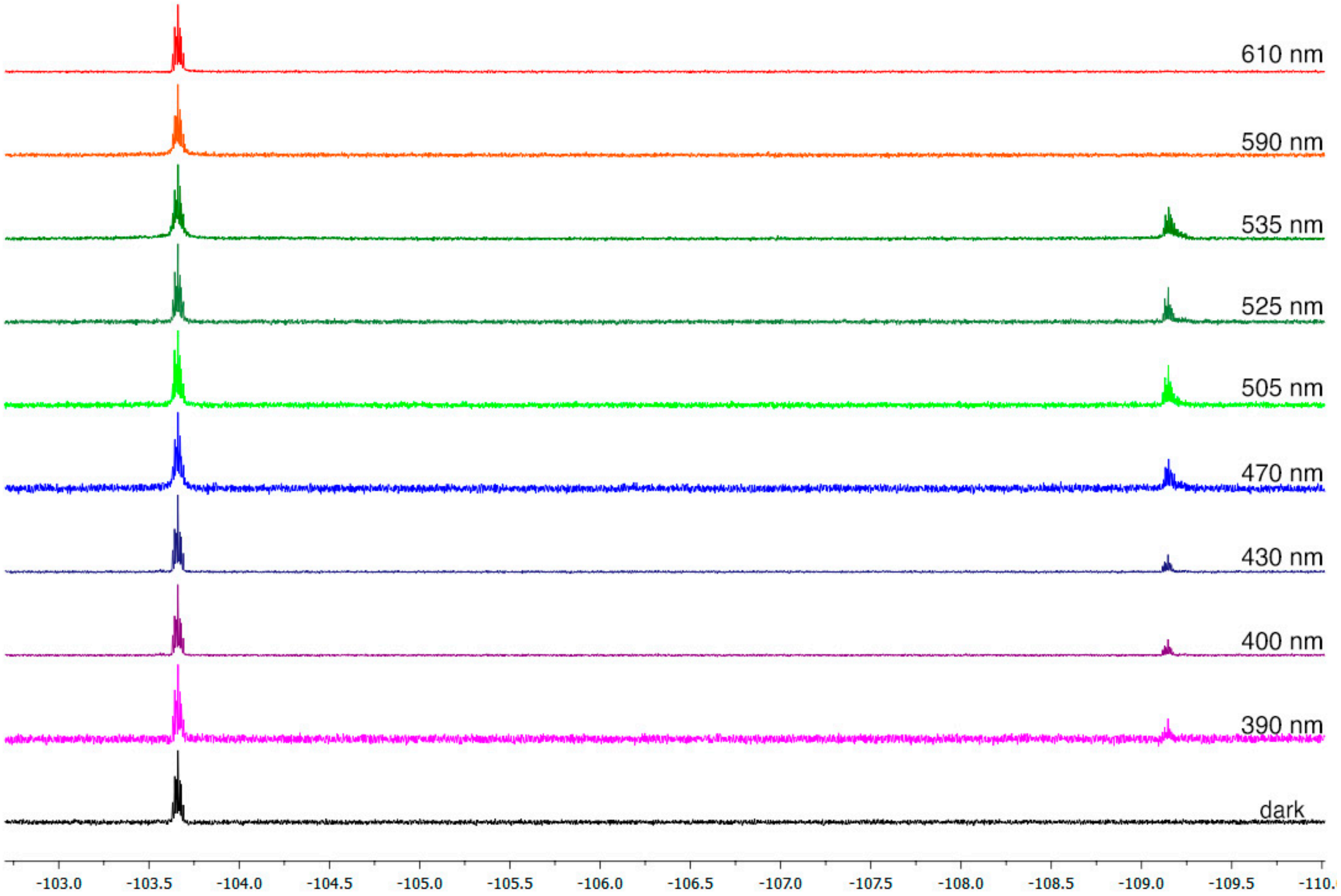

610 nm
590 nm
535 nm
525 nm
505 nm
470 nm
430 nm
400 nm
390 nm
dark
-103.0
-103.5
-104.0
-104.5
-105.0
-105.5
-106.0
-106.5
-107.0
-107.5
-108.0
-108.5
-109.0
-109.5
-110.

17e:

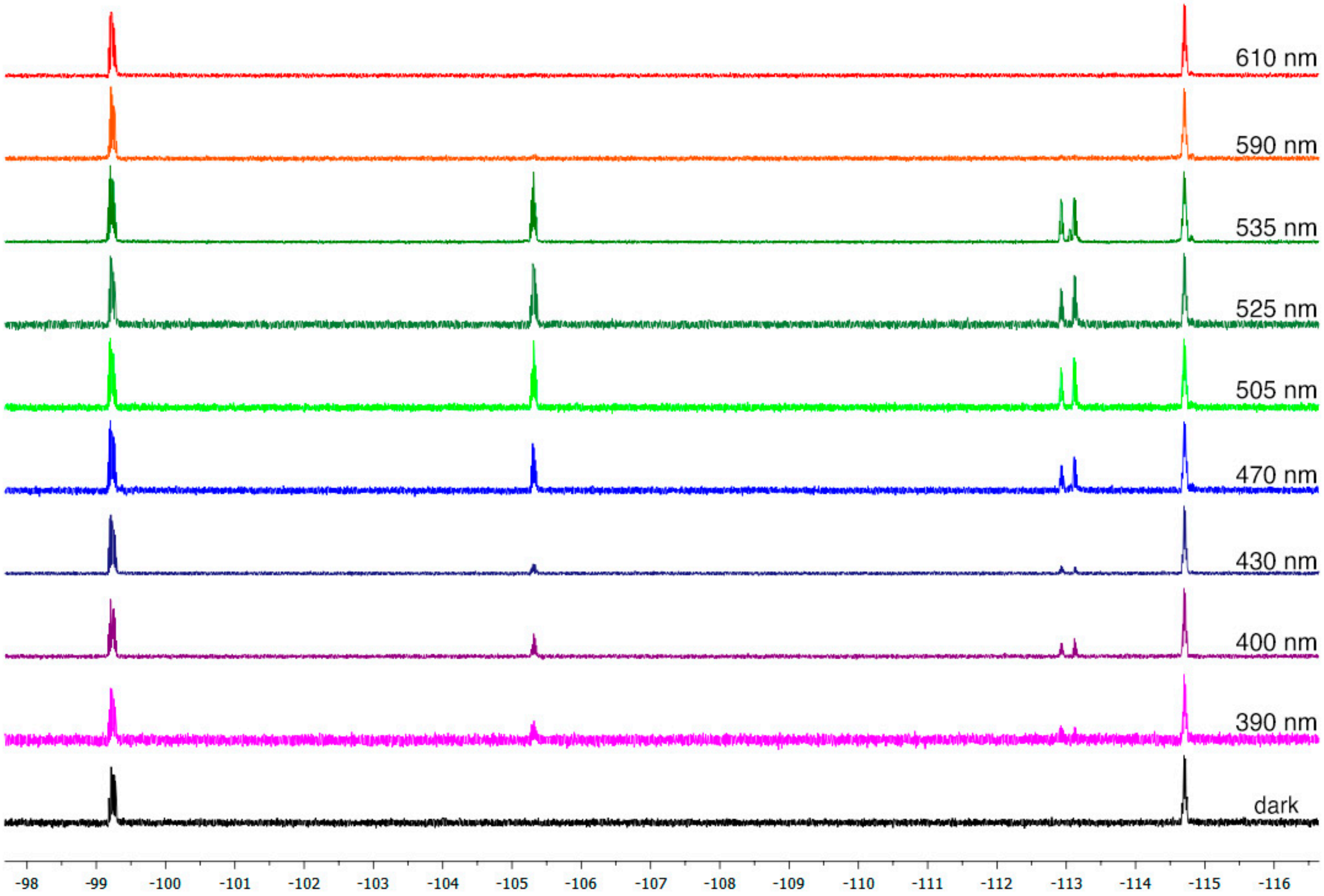

610 nm
590 nm
535 nm
525 nm
505 nm
470 nm
430 nm
400 nm
390 nm
dark
-98
-99
-100
-101
-102
-103
-104
-105
-106
-107
-108
-109
-110
-111
-112
-113
-114
-115
-116

17f:

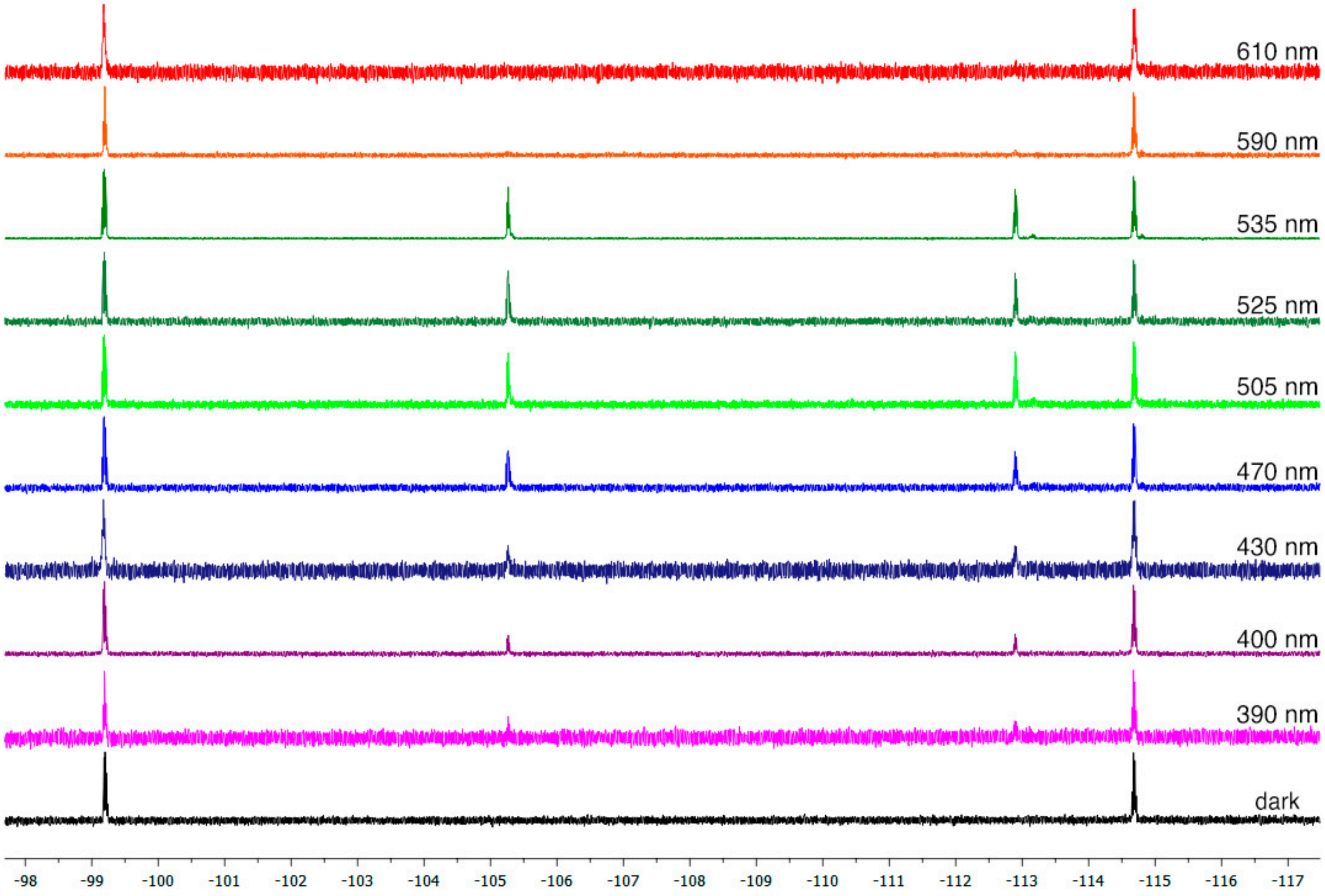
610 nm
590 nm
535 nm
525 nm
505 nm
470 nm
430 nm
400 nm
390 nm
dark
-98
-99
-100
-101
-102
-103
-104
-105
-106
-107
-108
-109
-110
-111
-112
-113
-114
-115
-116
-117

17g:

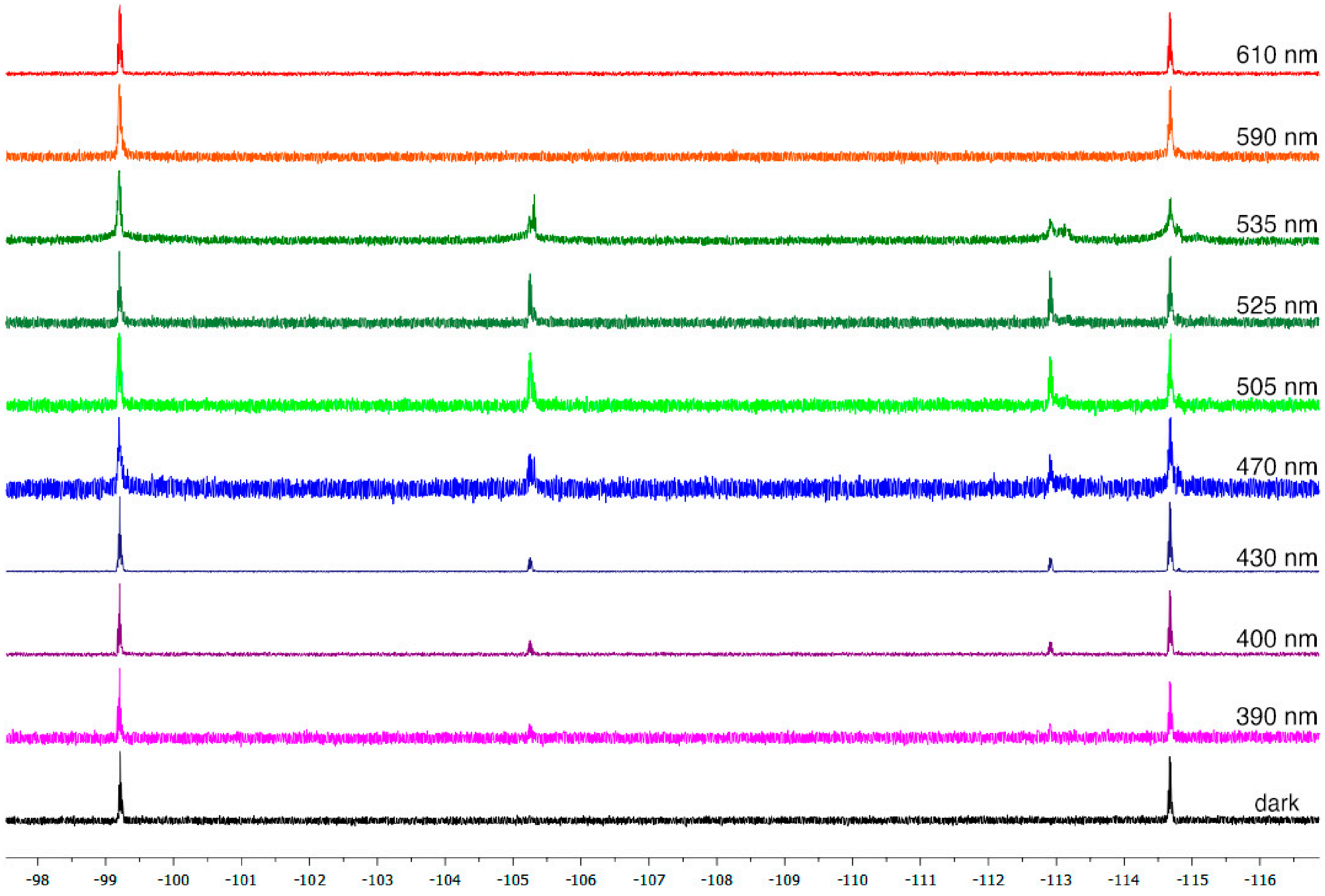
610 nm
590 nm
535 nm
525 nm
505 nm
470 nm
430 nm
400 nm
390 nm
dark
-98
-99
-100
-101
-102
-103
-104
-105
-106
-107
-108
-109
-110
-111
-112
-113
-114
-115
-116

17i:

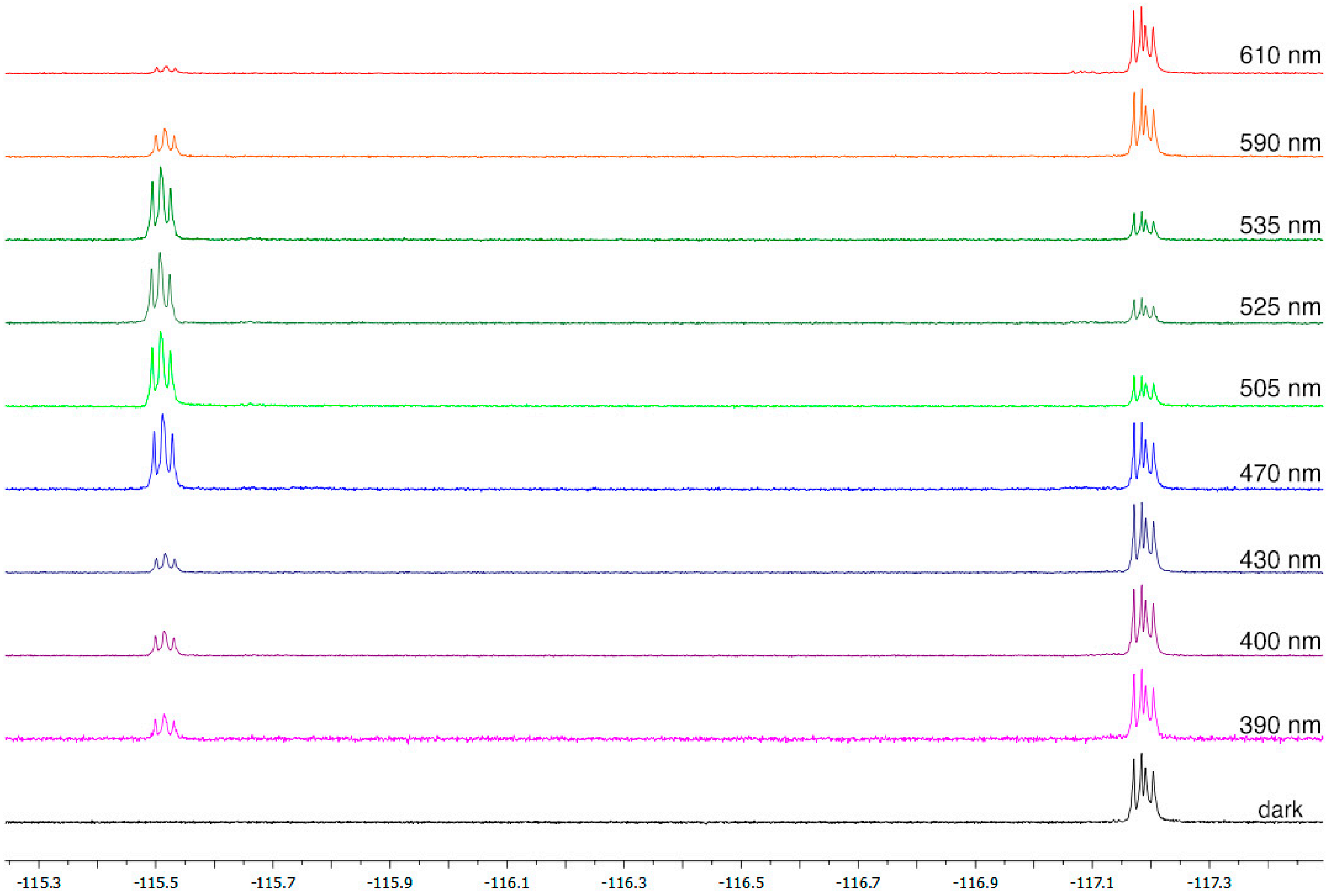
610 nm
590 nm
535 nm
525 nm
505 nm
470 nm
430 nm
400 nm
390 nm
dark
-115.3
-115.5
-115.7
-115.9
-116.1
-116.3
-116.5
-116.7
-116.9
-117.1
-117.3

17j:

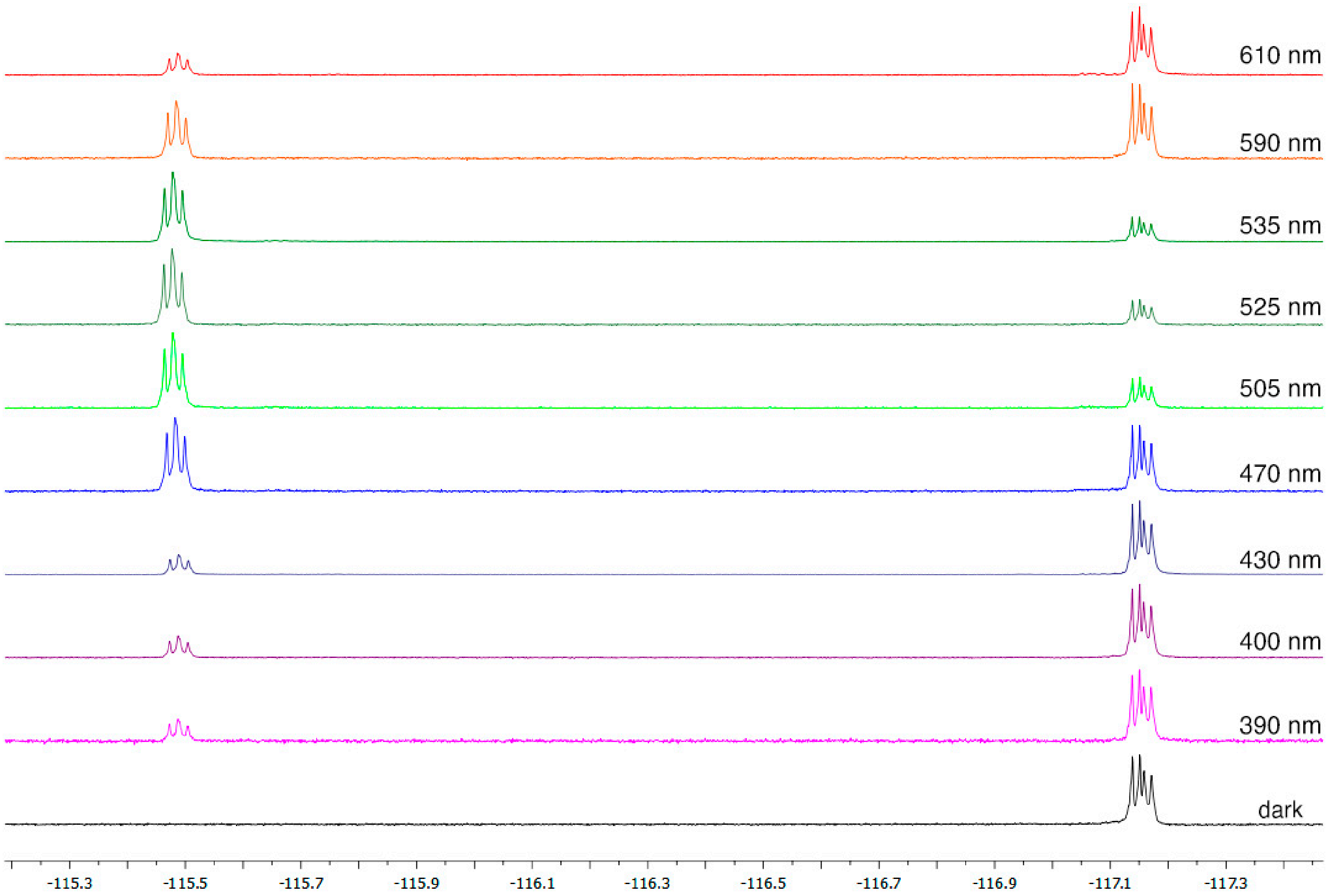
610 nm
590 nm
535 nm
525 nm
505 nm
470 nm
430 nm
400 nm
390 nm
dark
-115.3
-115.5
-115.7
-115.9
-116.1
-116.3
-116.5
-116.7
-116.9
-117.1
-117.3

17k:

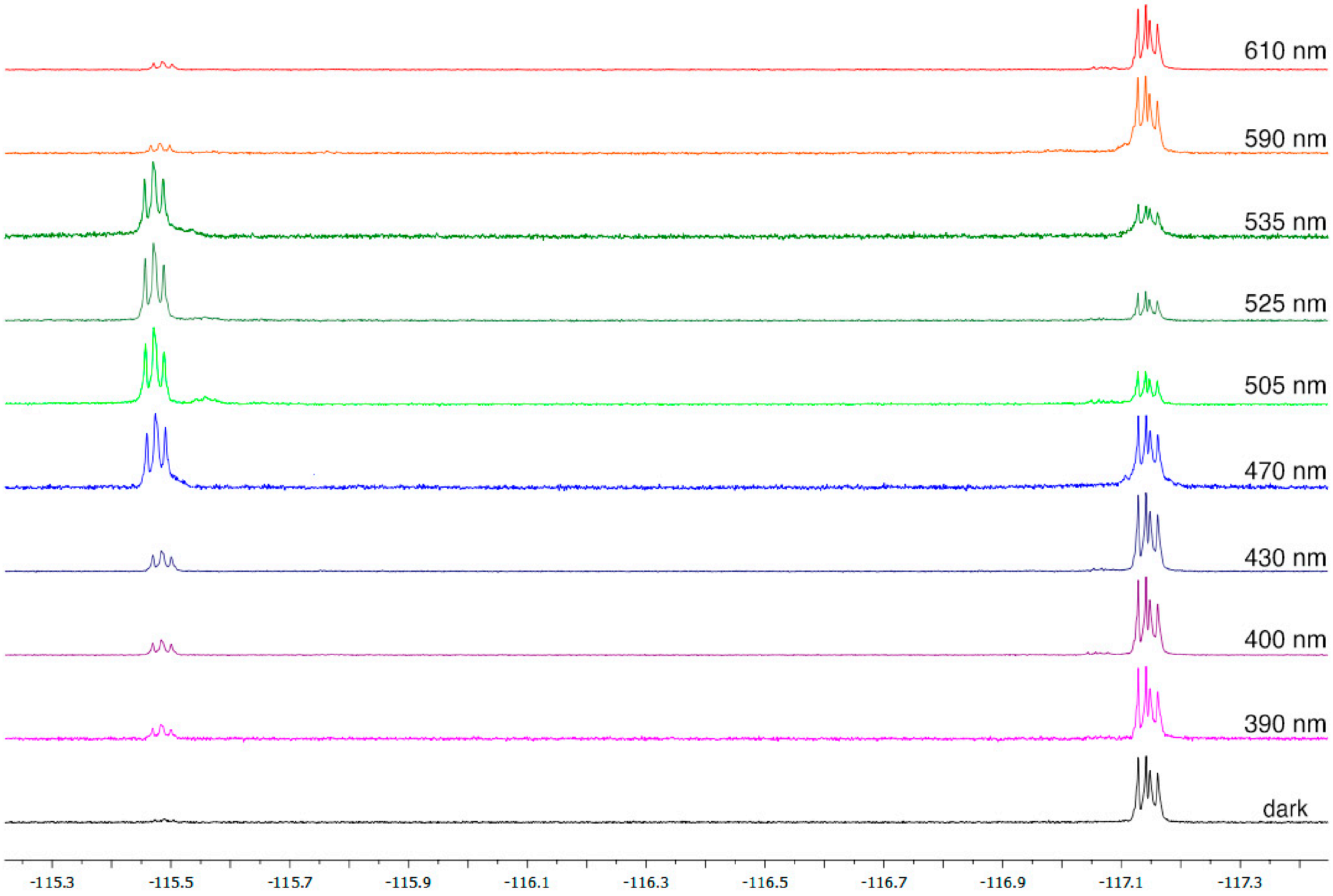
610 nm
590 nm
535 nm
525 nm
505 nm
470 nm
430 nm
400 nm
390 nm
dark
-115.3
-115.5
-115.7
-115.9
-116.1
-116.3
-116.5
-116.7
-116.9
-117.1
-117.3

17l:

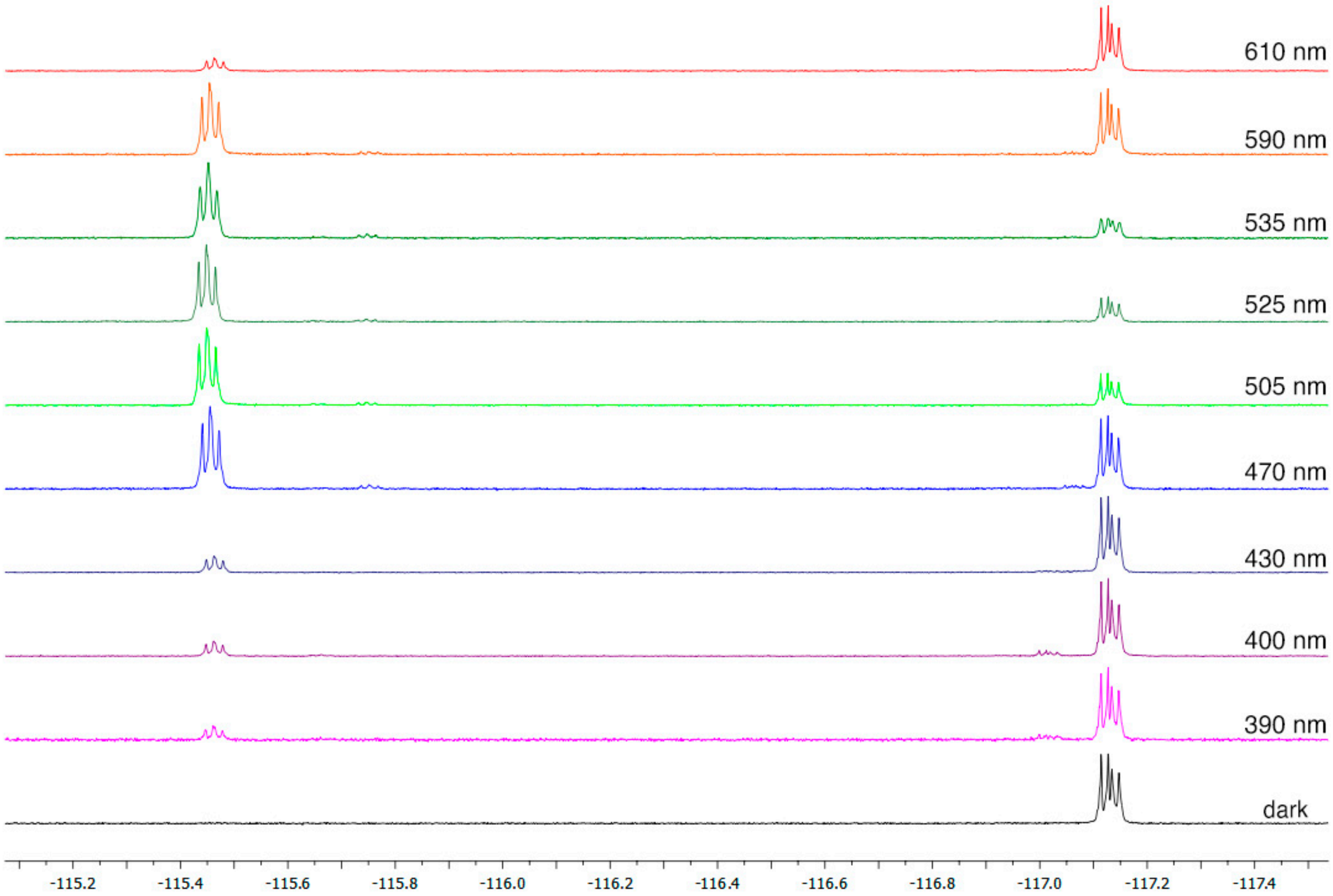
610 nm
590 nm
535 nm
525 nm
505 nm
470 nm
430 nm
400 nm
390 nm
dark
-115.2
-115.4
-115.6
-115.8
-116.0
-116.2
-116.4
-116.6
-116.8
-117.0
-117.2
-117.4

4.3 Photoisomerization kinetics

Changes over time (photoisomerization kinetics) transitions from *Z* to *E* upon irradiation for (**17a-l**) irradiated at 535 nm (for sample concentration of 100 mM in DMSO) determined by ^{19}F NMR analysis.

17a:

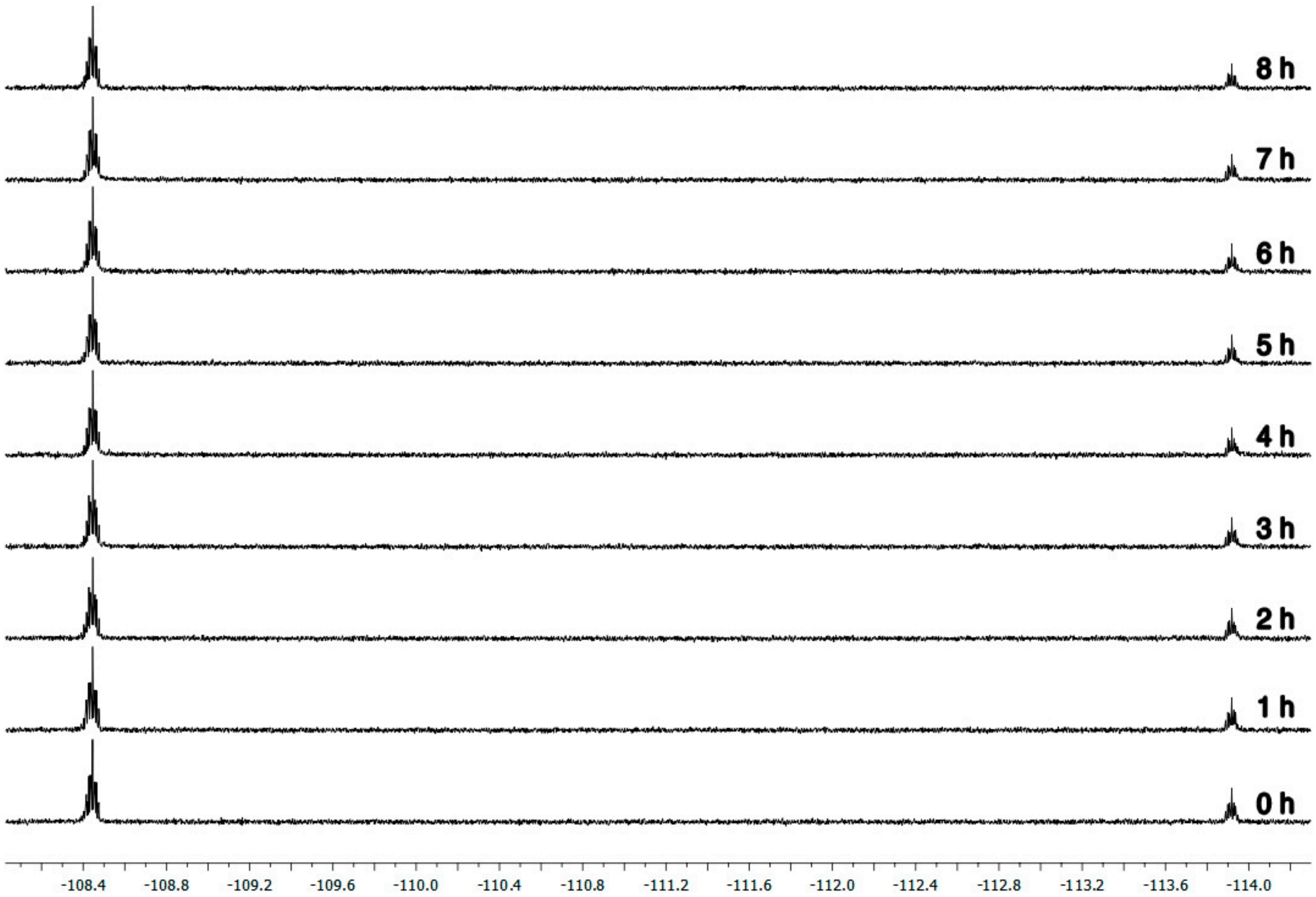

17b:

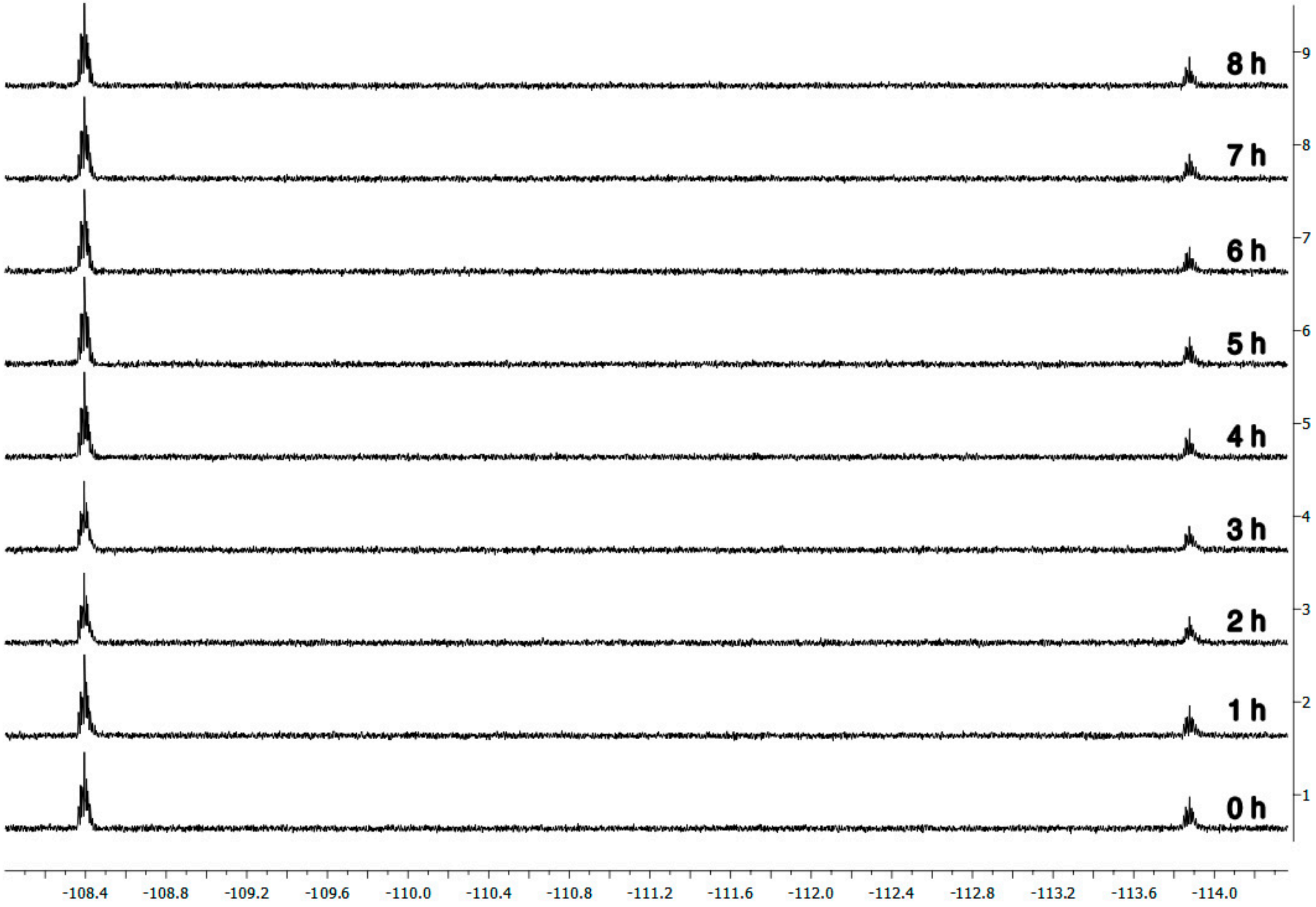
8 h
7 h
6 h
5 h
4 h
3 h
2 h
1 h
0 h
9
8
7
6
5
4
3
2
1
-108.4
-108.8
-109.2
-109.6
-110.0
-110.4
-110.8
-111.2
-111.6
-112.0
-112.4
-112.8
-113.2
-113.6
-114.0

17c:

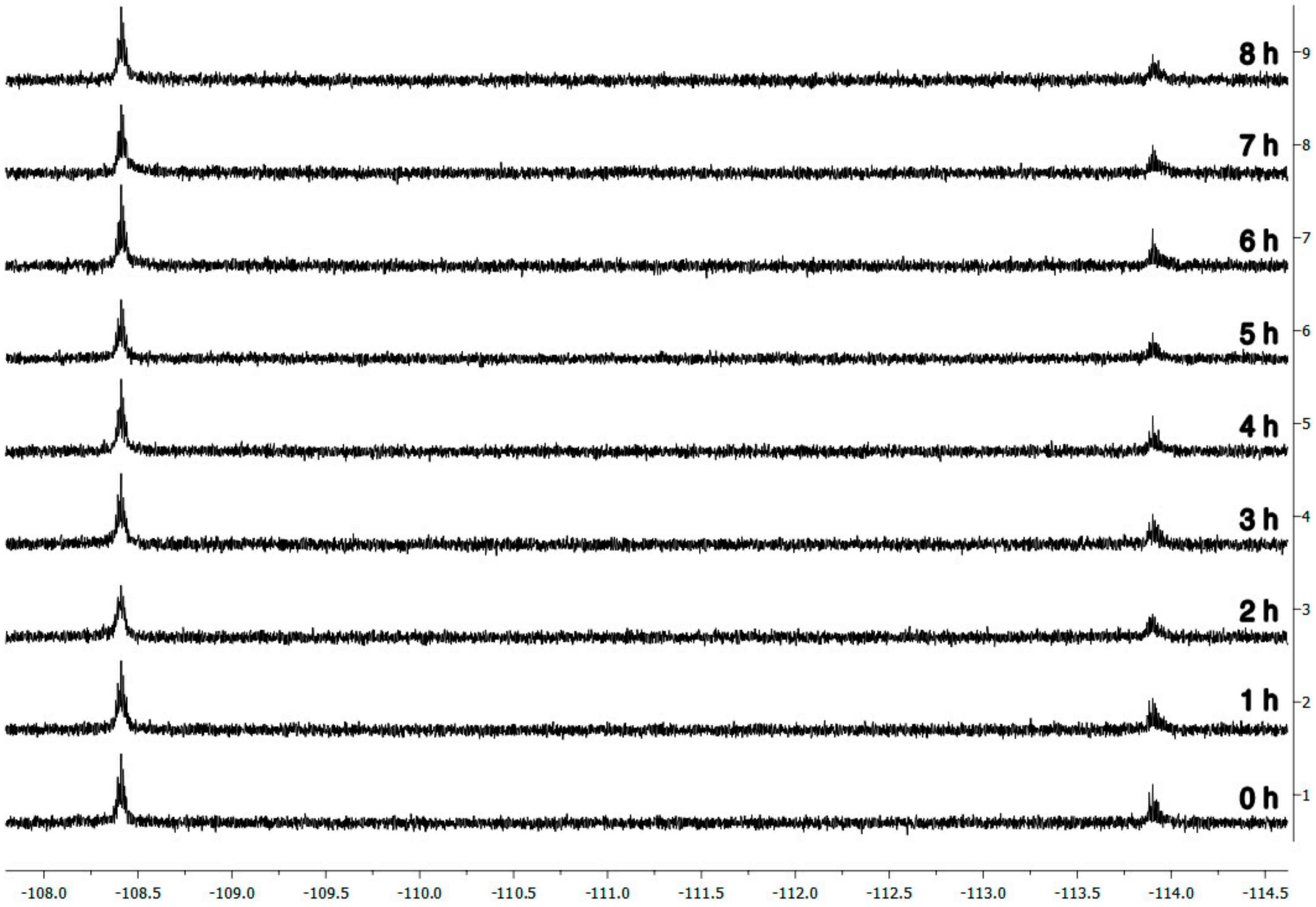
8 h
7 h
6 h
5 h
4 h
3 h
2 h
1 h
0 h
9
8
7
6
5
4
3
2
1
-108.0
-108.5
-109.0
-109.5
-110.0
-110.5
-111.0
-111.5
-112.0
-112.5
-113.0
-113.5
-114.0
-114.5

17e:

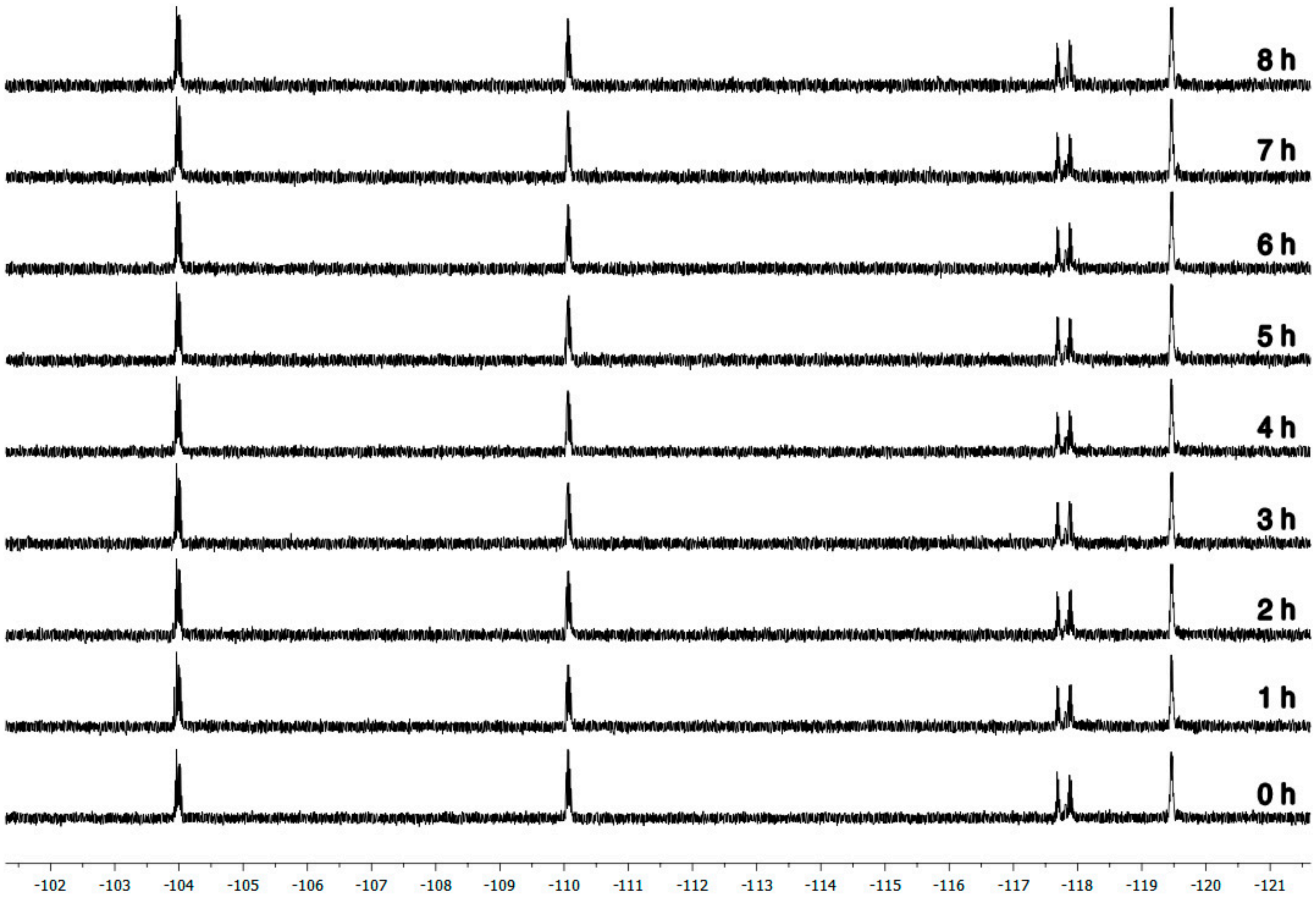
8 h
7 h
6 h
5 h
4 h
3 h
2 h
1 h
0 h
-102 -103 -104 -105 -106 -107 -108 -109 -110 -111 -112 -113 -114 -115 -116 -117 -118 -119 -120 -121

17f:

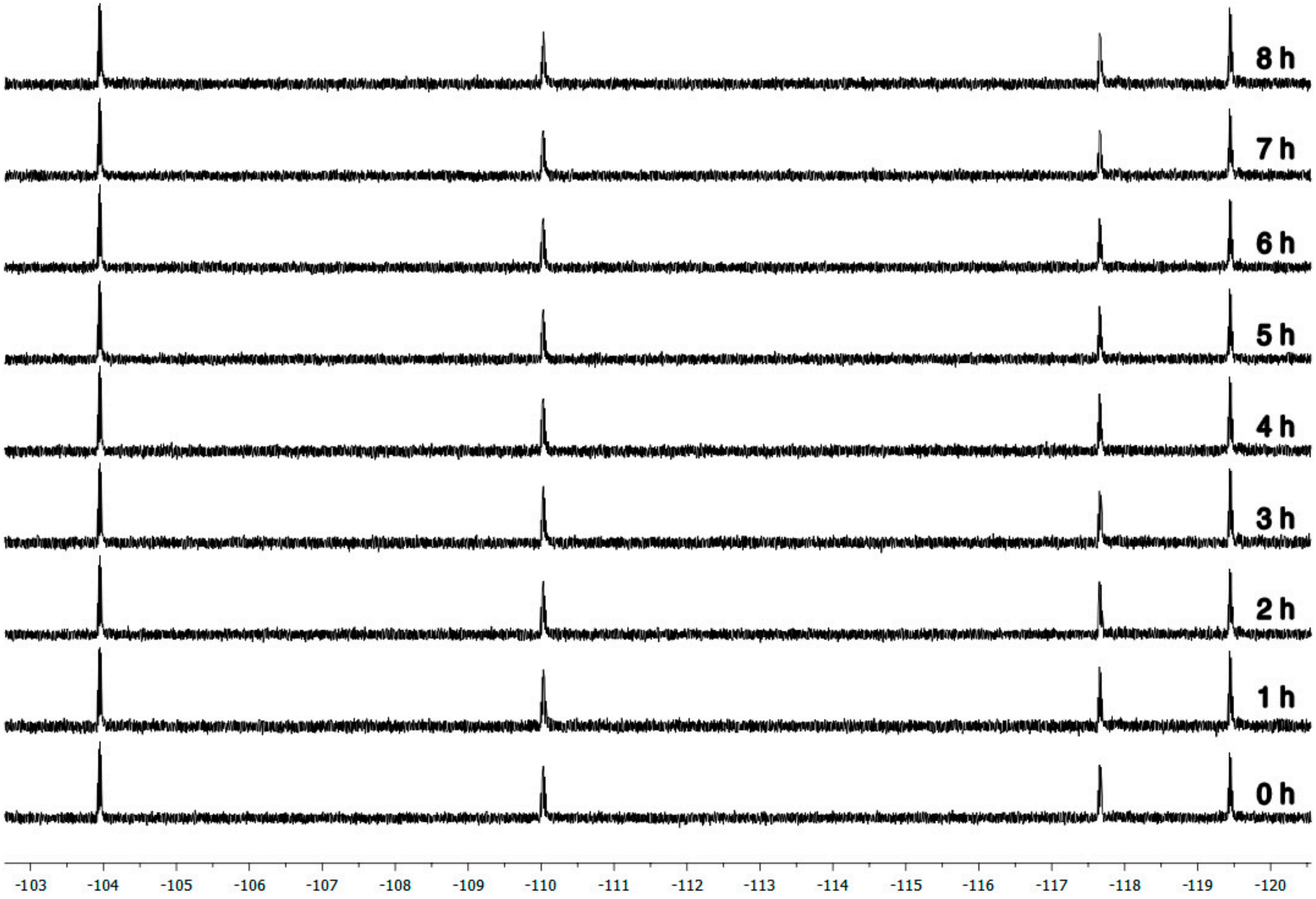
8 h
7 h
6 h
5 h
4 h
3 h
2 h
1 h
0 h
-103
-104
-105
-106
-107
-108
-109
-110
-111
-112
-113
-114
-115
-116
-117
-118
-119
-120

17g:

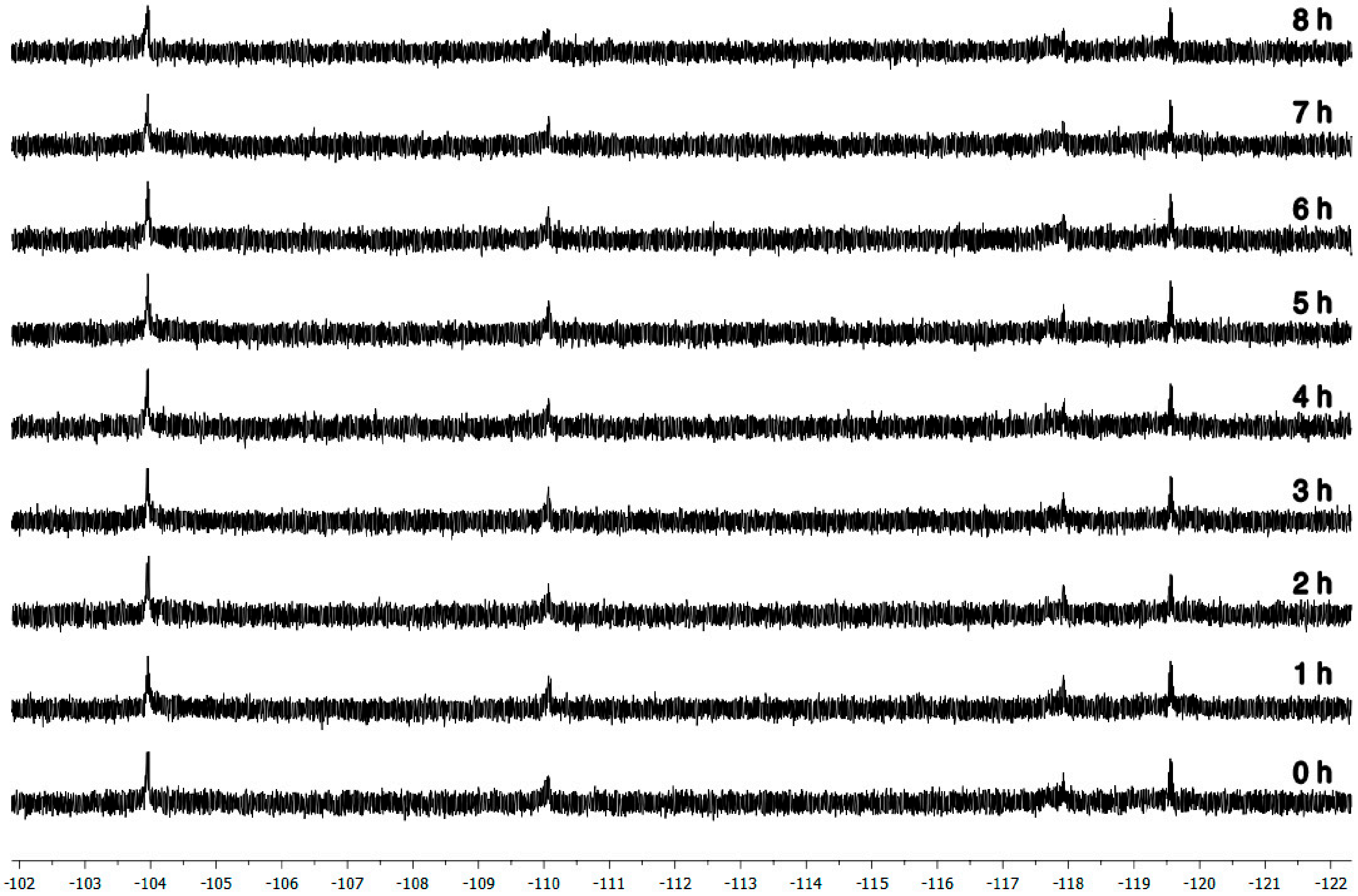
8 h
7 h
6 h
5 h
4 h
3 h
2 h
1 h
0 h
-102
-103
-104
-105
-106
-107
-108
-109
-110
-111
-112
-113
-114
-115
-116
-117
-118
-119
-120
-121
-122

17i:

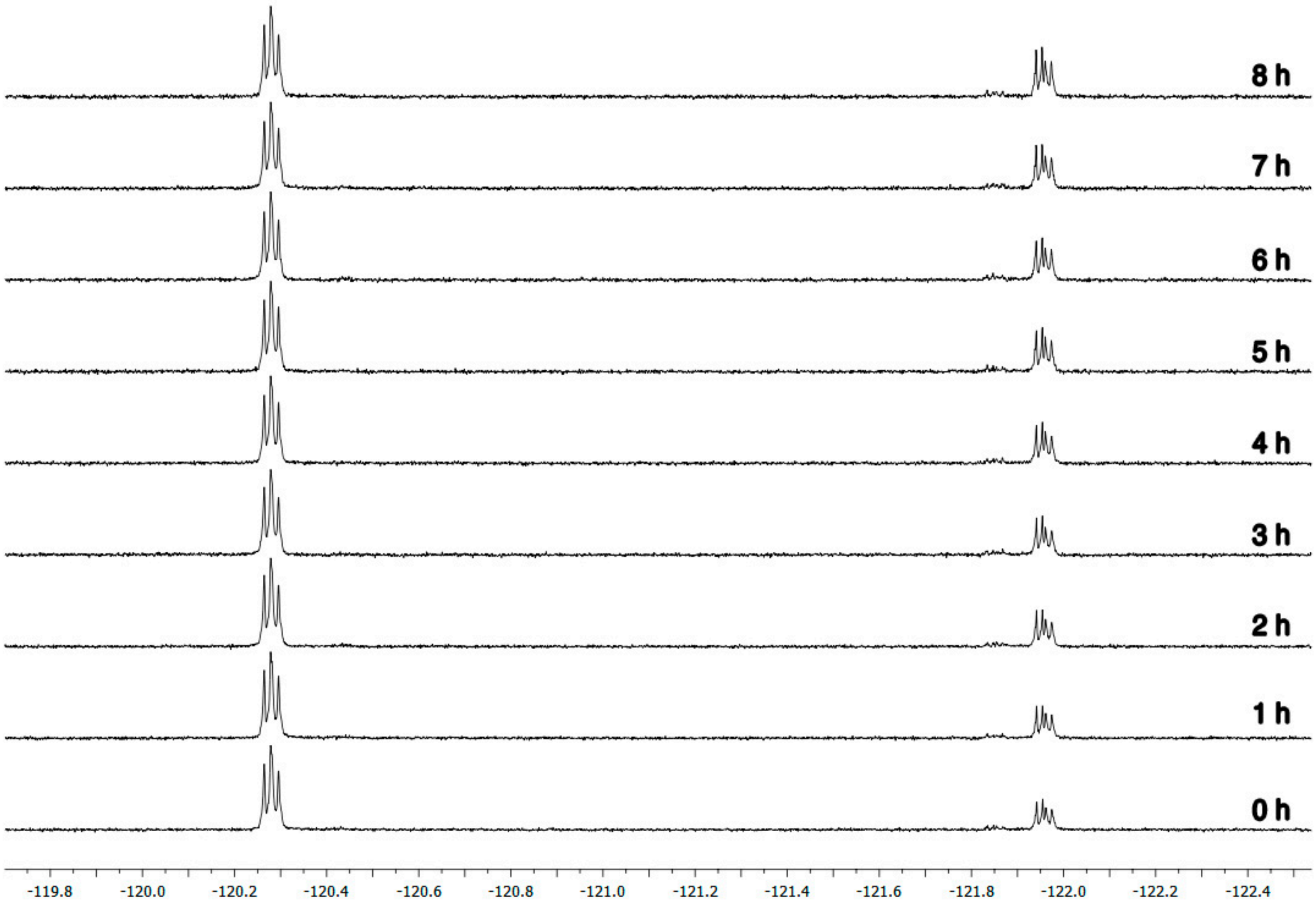
8 h
7 h
6 h
5 h
4 h
3 h
2 h
1 h
0 h
-119.8
-120.0
-120.2
-120.4
-120.6
-120.8
-121.0
-121.2
-121.4
-121.6
-121.8
-122.0
-122.2
-122.4

17j:

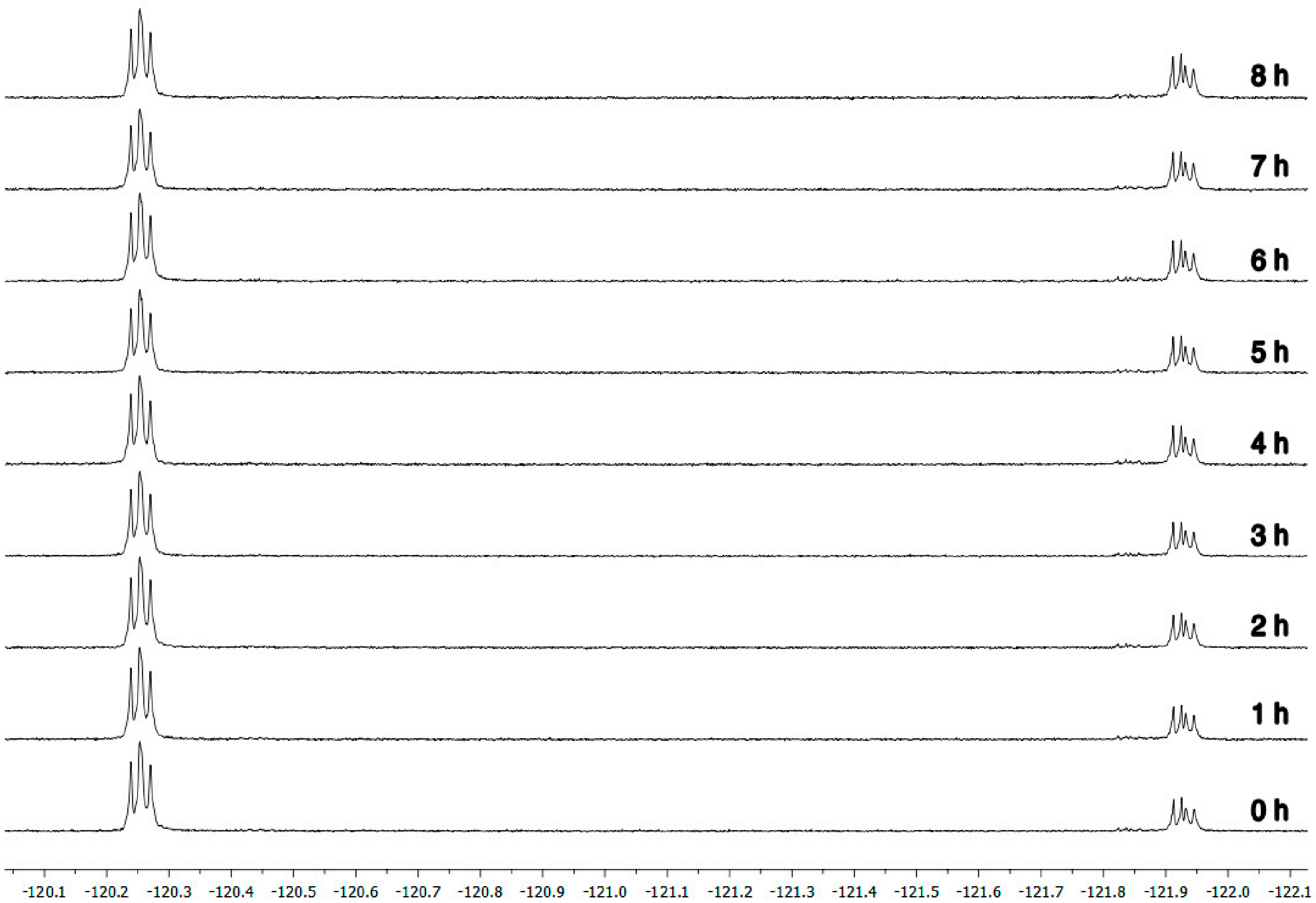
8 h
7 h
6 h
5 h
4 h
3 h
2 h
1 h
0 h
-120.1 -120.2 -120.3 -120.4 -120.5 -120.6 -120.7 -120.8 -120.9 -121.0 -121.1 -121.2 -121.3 -121.4 -121.5 -121.6 -121.7 -121.8 -121.9 -122.0 -122.1

17k:

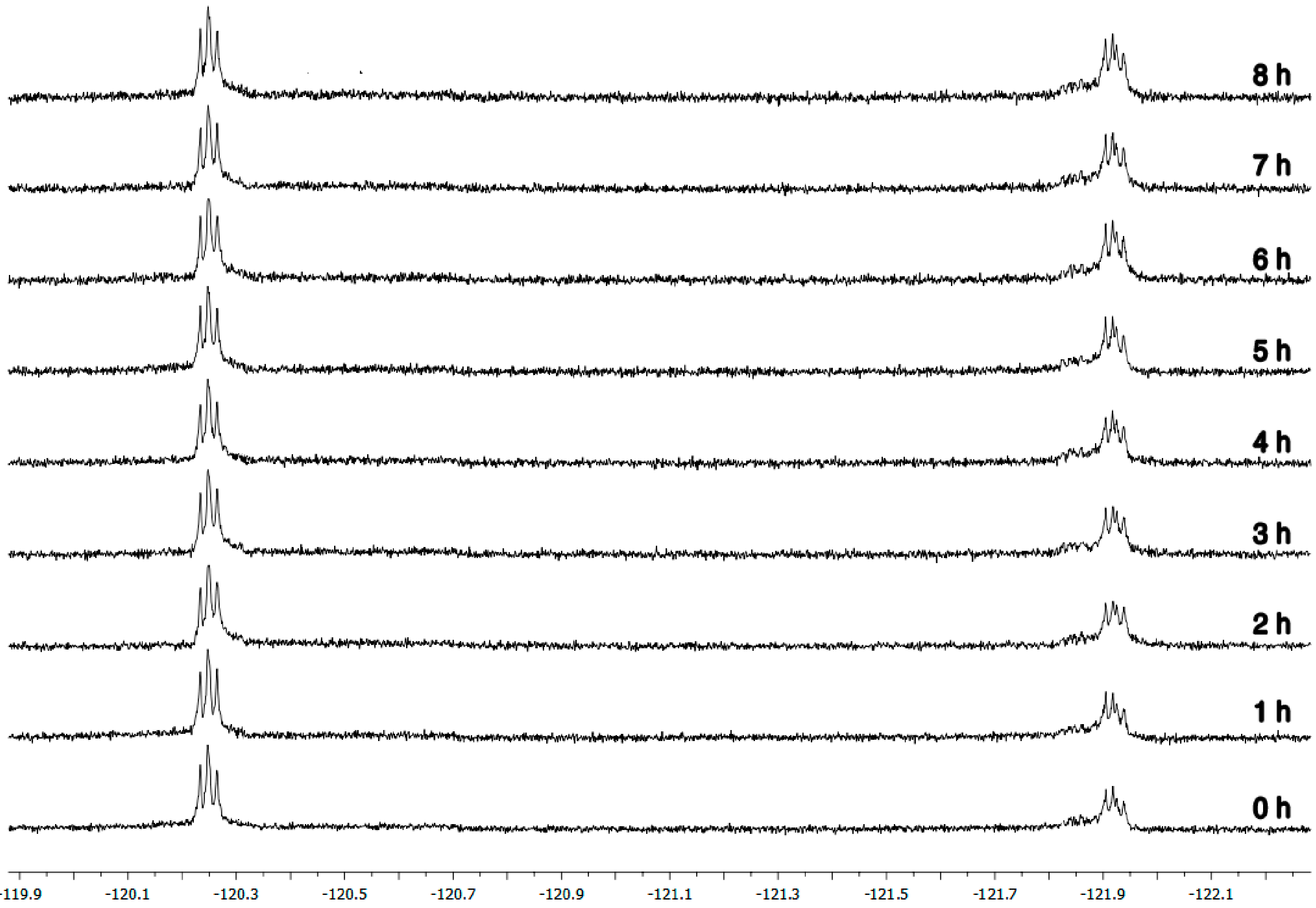

8 h
7 h
6 h
5 h
4 h
3 h
2 h
1 h
0 h
-119.9
-120.1
-120.3
-120.5
-120.7
-120.9
-121.1
-121.3
-121.5
-121.7
-121.9
-122.1

17l:

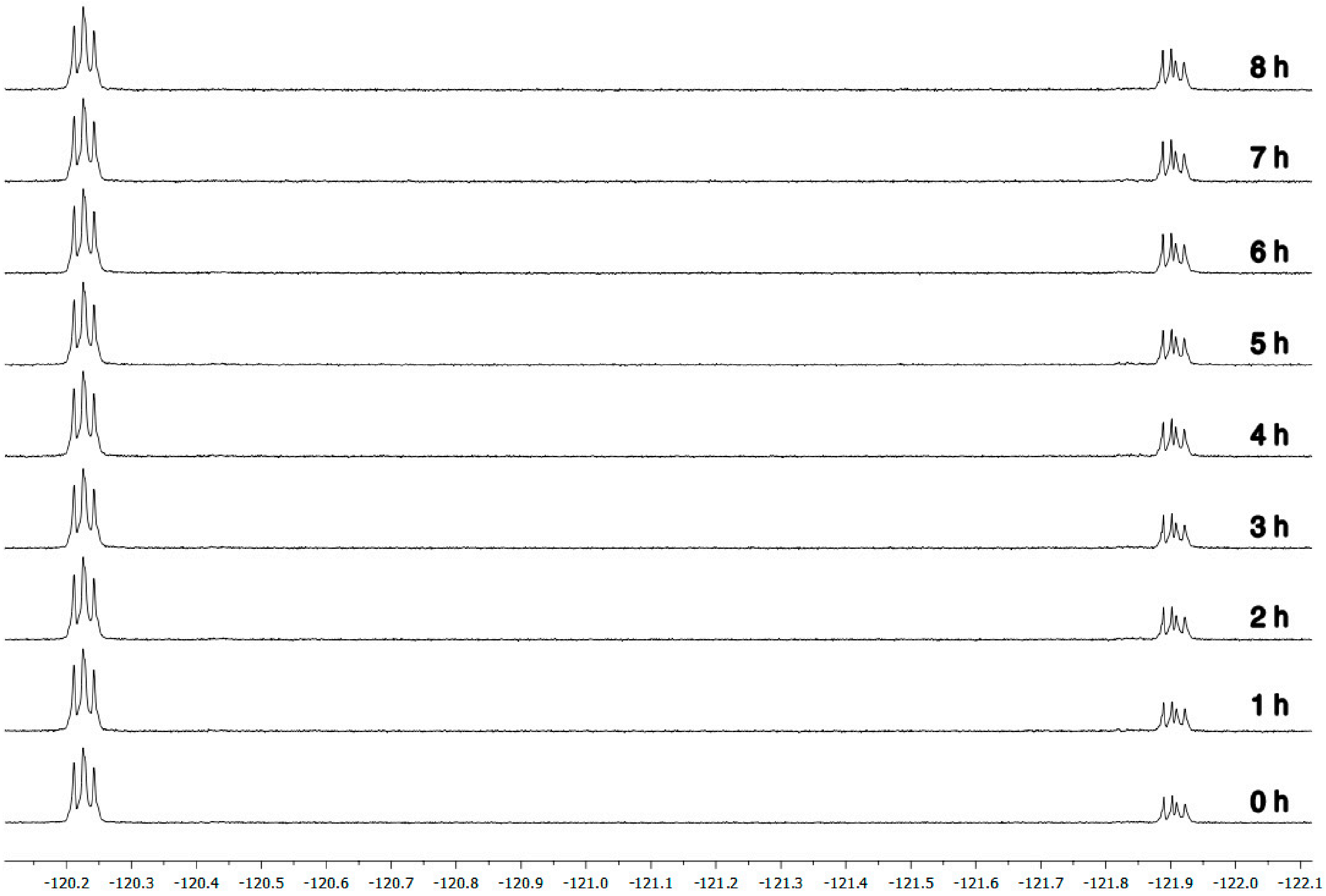

8 h
7 h
6 h
5 h
4 h
3 h
2 h
1 h
0 h
-120.2 -120.3 -120.4 -120.5 -120.6 -120.7 -120.8 -120.9 -121.0 -121.1 -121.2 -121.3 -121.4 -121.5 -121.6 -121.7 -121.8 -121.9 -122.0 -122.1

4.4 UV-ViS spectra

UV-ViS spectra of compounds **(17a-l)**:

17a:

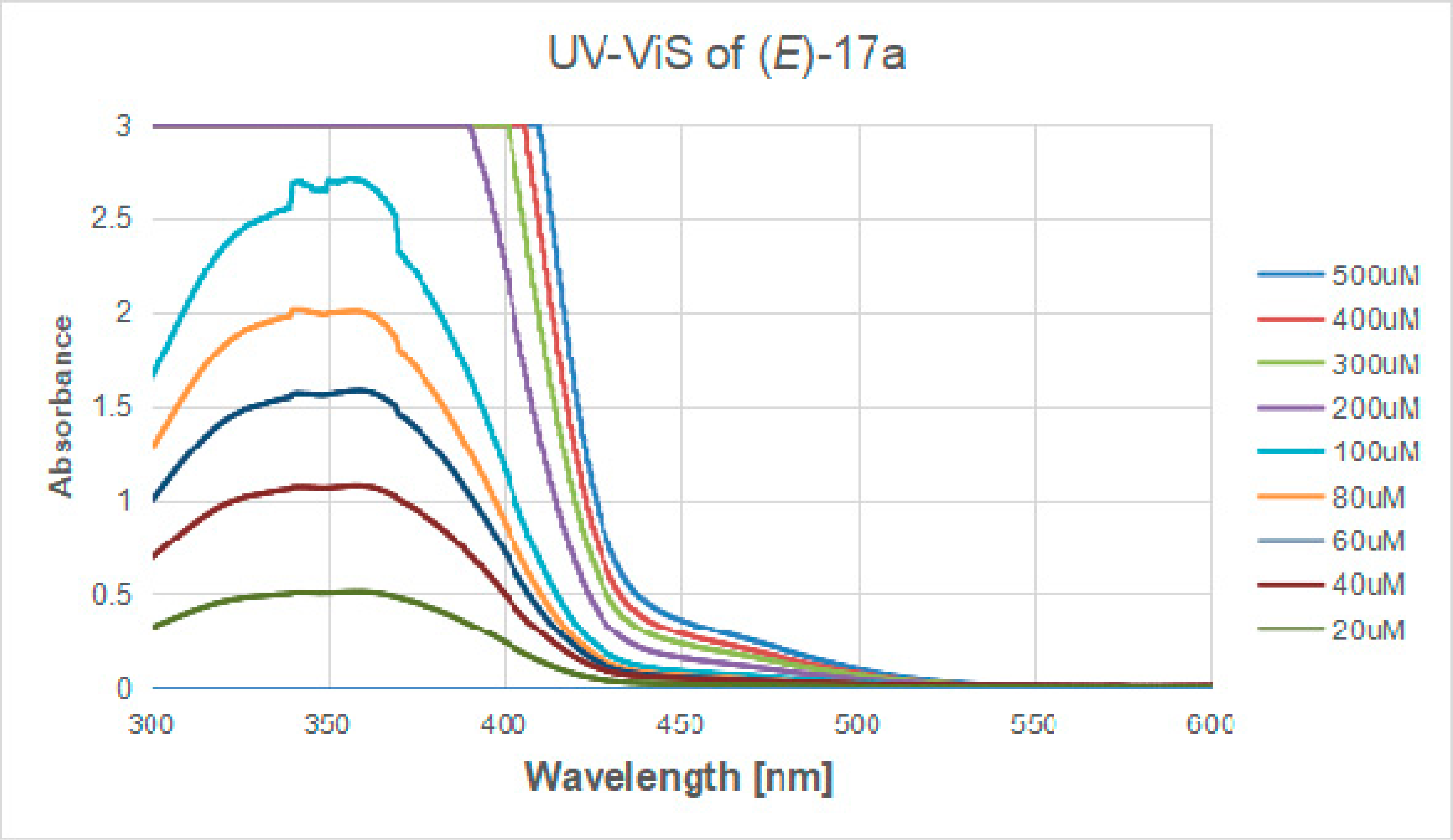

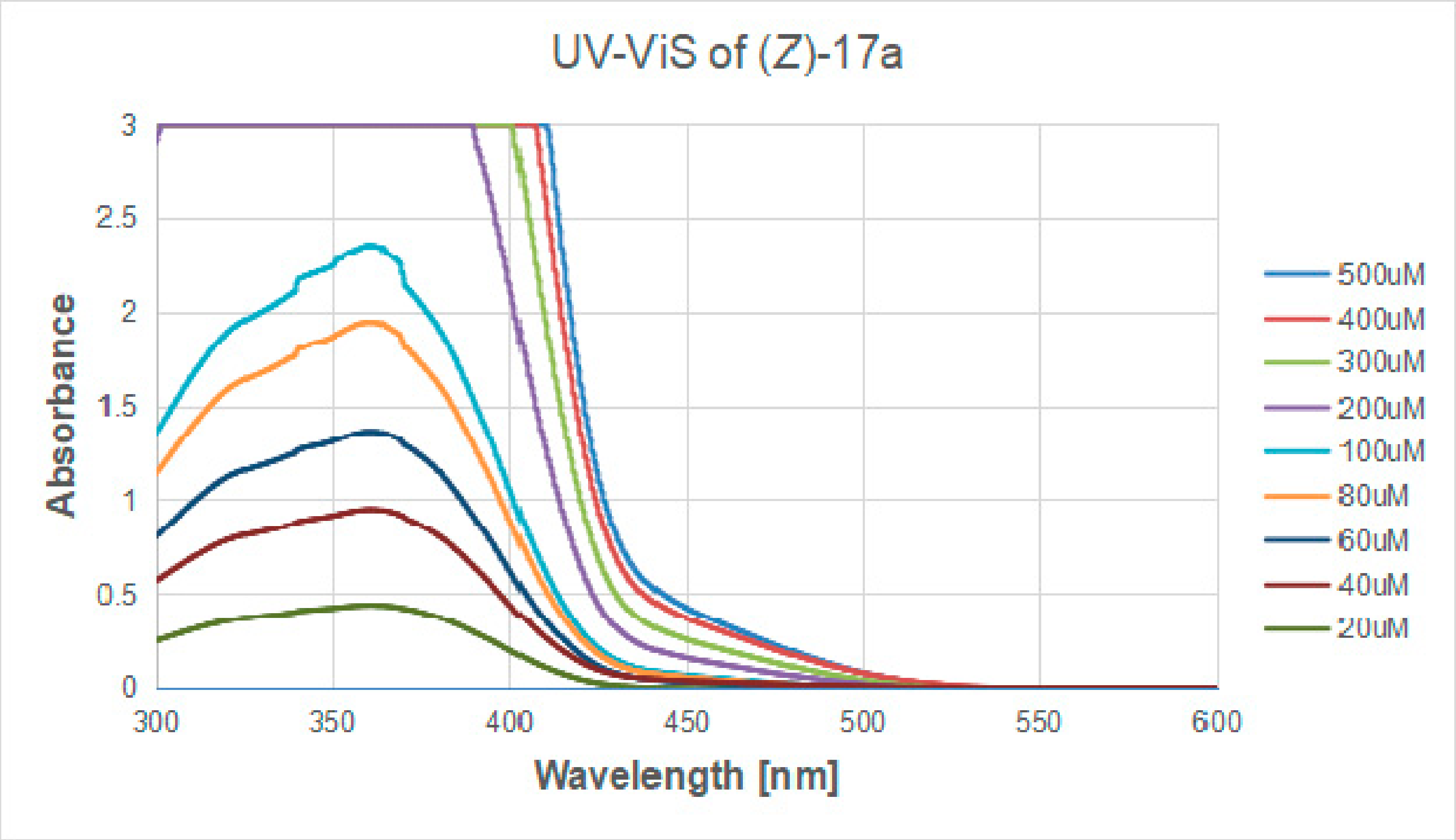
UV-ViS of (Z)-17a
Absorbance
Wavelength [nm]
3
2.5
2
1.5
1
0.5
0
300
350
400
450
500
550
600
500uM
400uM
300uM
200uM
100uM
80uM
60uM
40uM
20uM

17b:

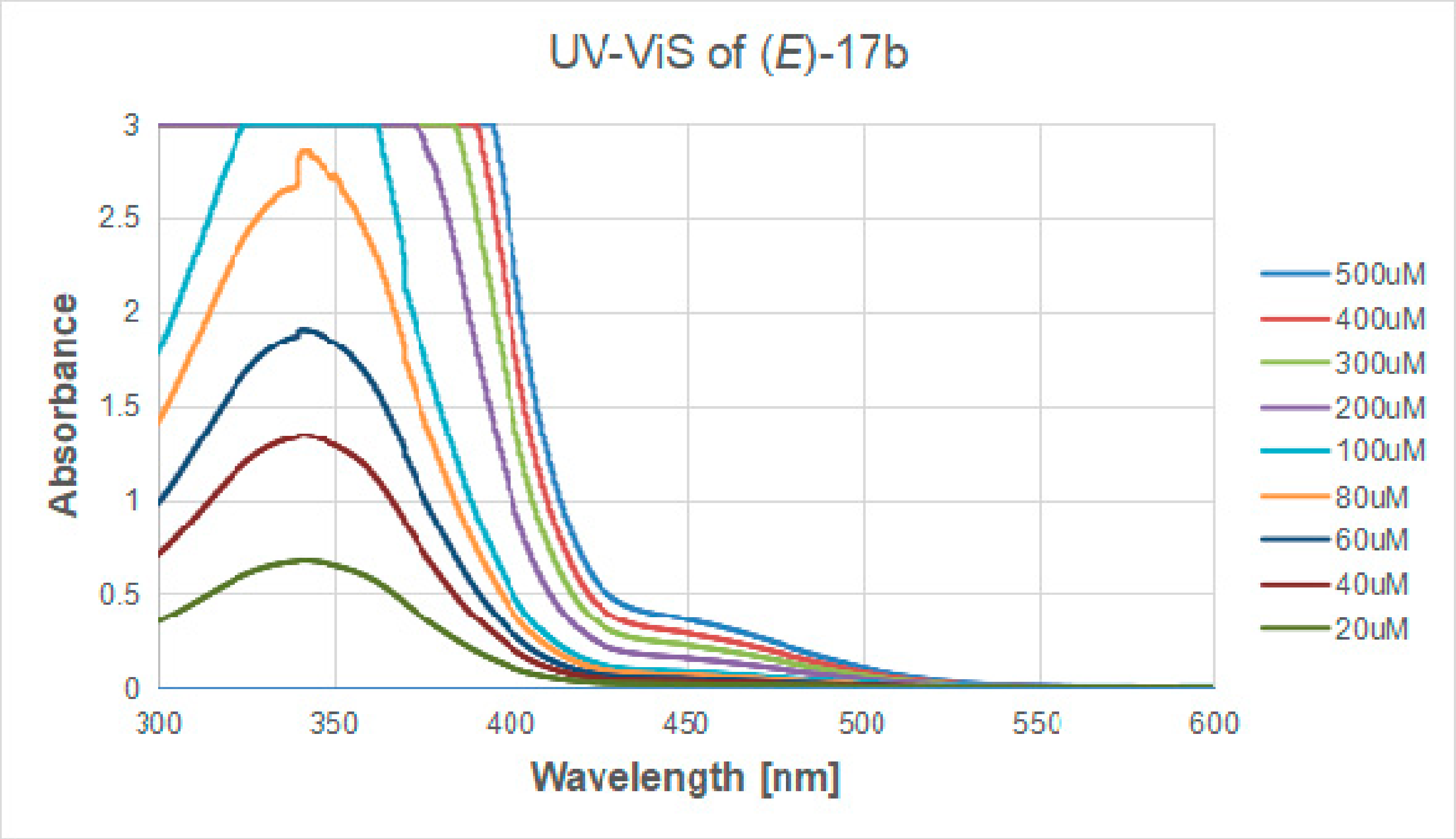

UV-ViS of (*E*)-17b
Absorbance
Wavelength [nm]
3
2.5
2
1.5
1
0.5
0
300
350
400
450
500
550
600
500uM
400uM
300uM
200uM
100uM
80uM
60uM
40uM
20uM

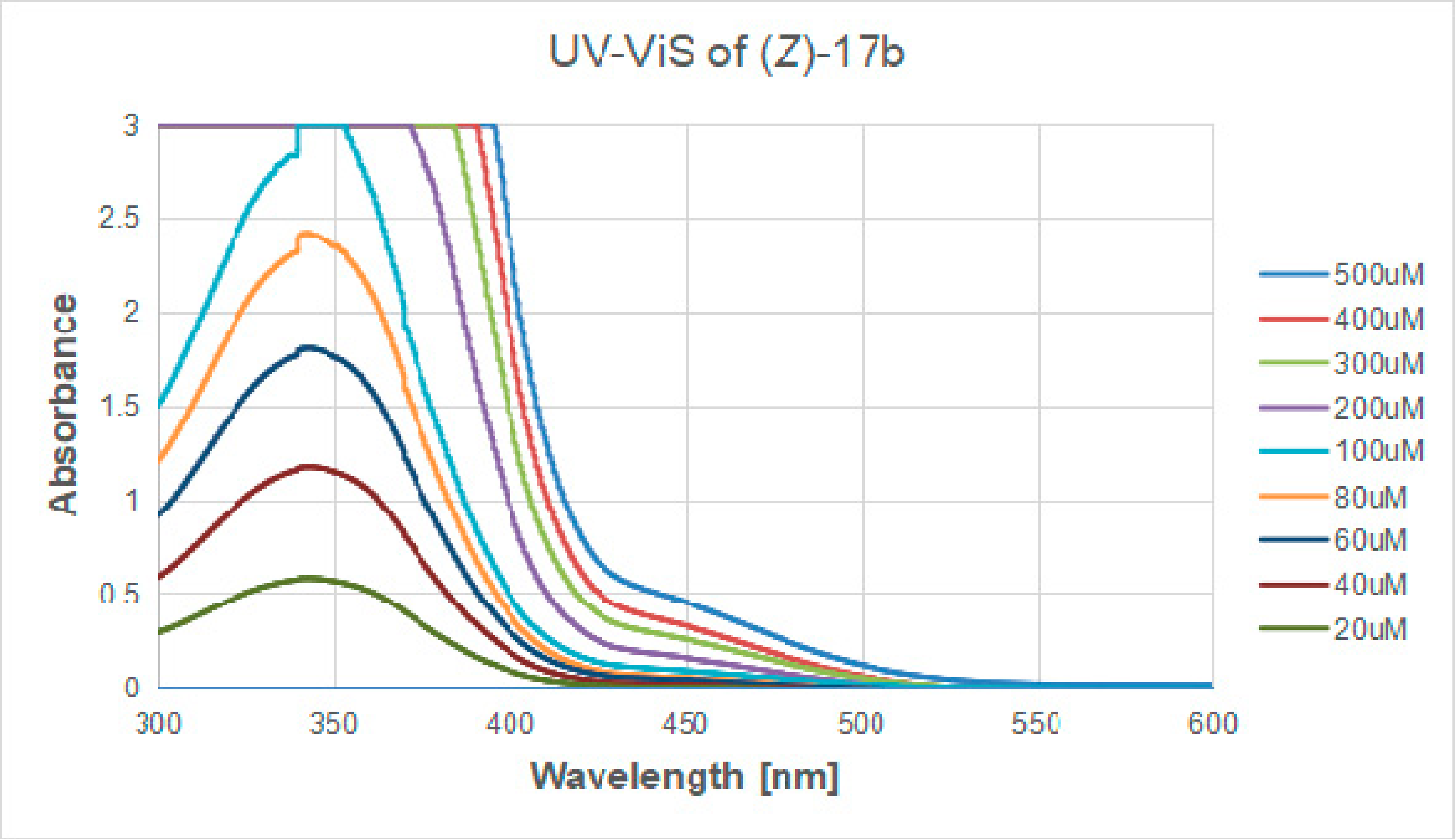

UV-ViS of (Z)-17b
Absorbance
3
2.5
2
1.5
1
0.5
0
300
350
400
450
500
550
600
Wavelength [nm]
500uM
400uM
300uM
200uM
100uM
80uM
60uM
40uM
20uM

17c:

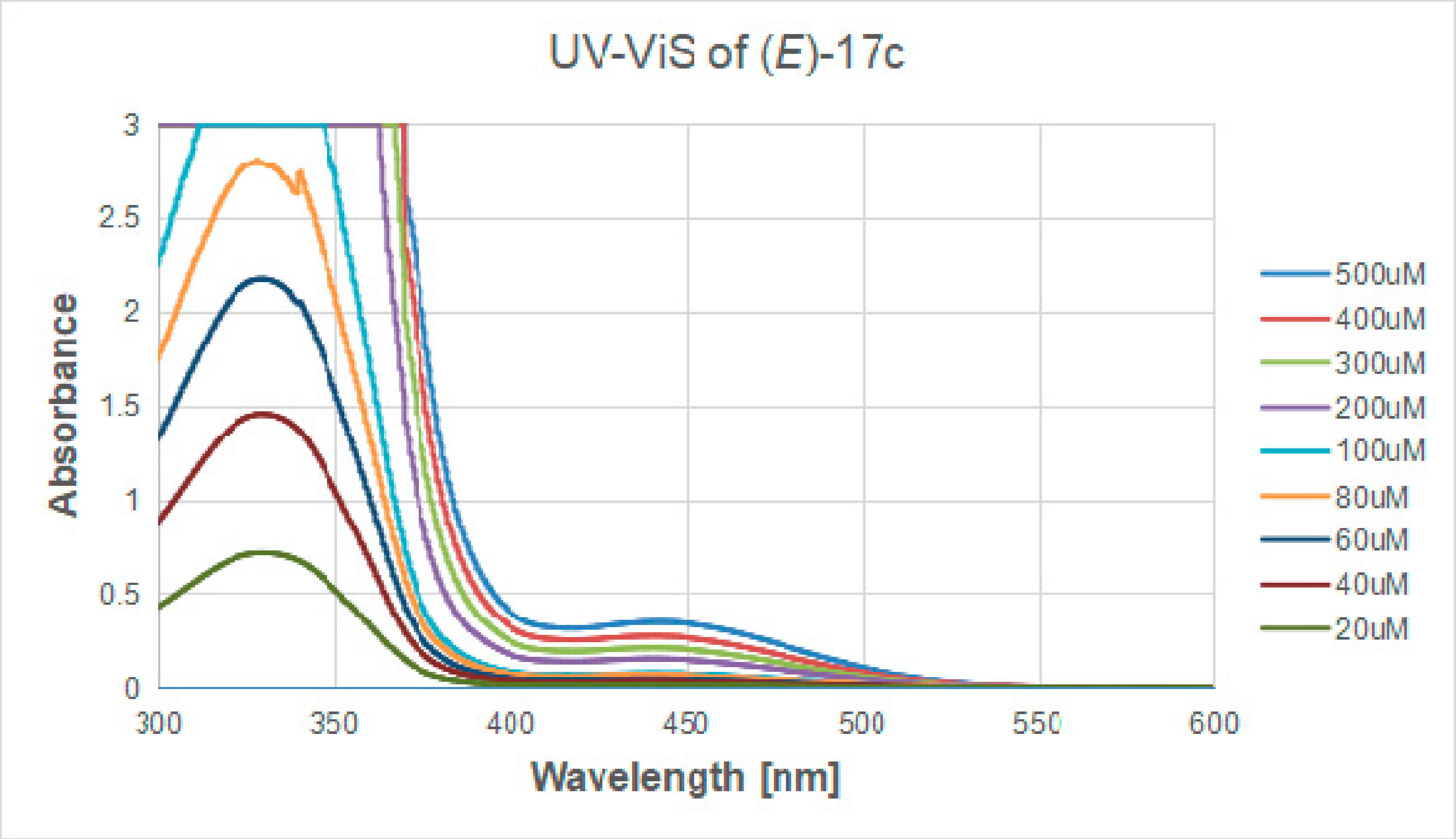

UV-ViS of (*E*)-17c
3
2.5
2
1.5
1
0.5
0
Absorbance
300
350
400
450
500
550
600
Wavelength [nm]
500uM
400uM
300uM
200uM
100uM
80uM
60uM
40uM
20uM

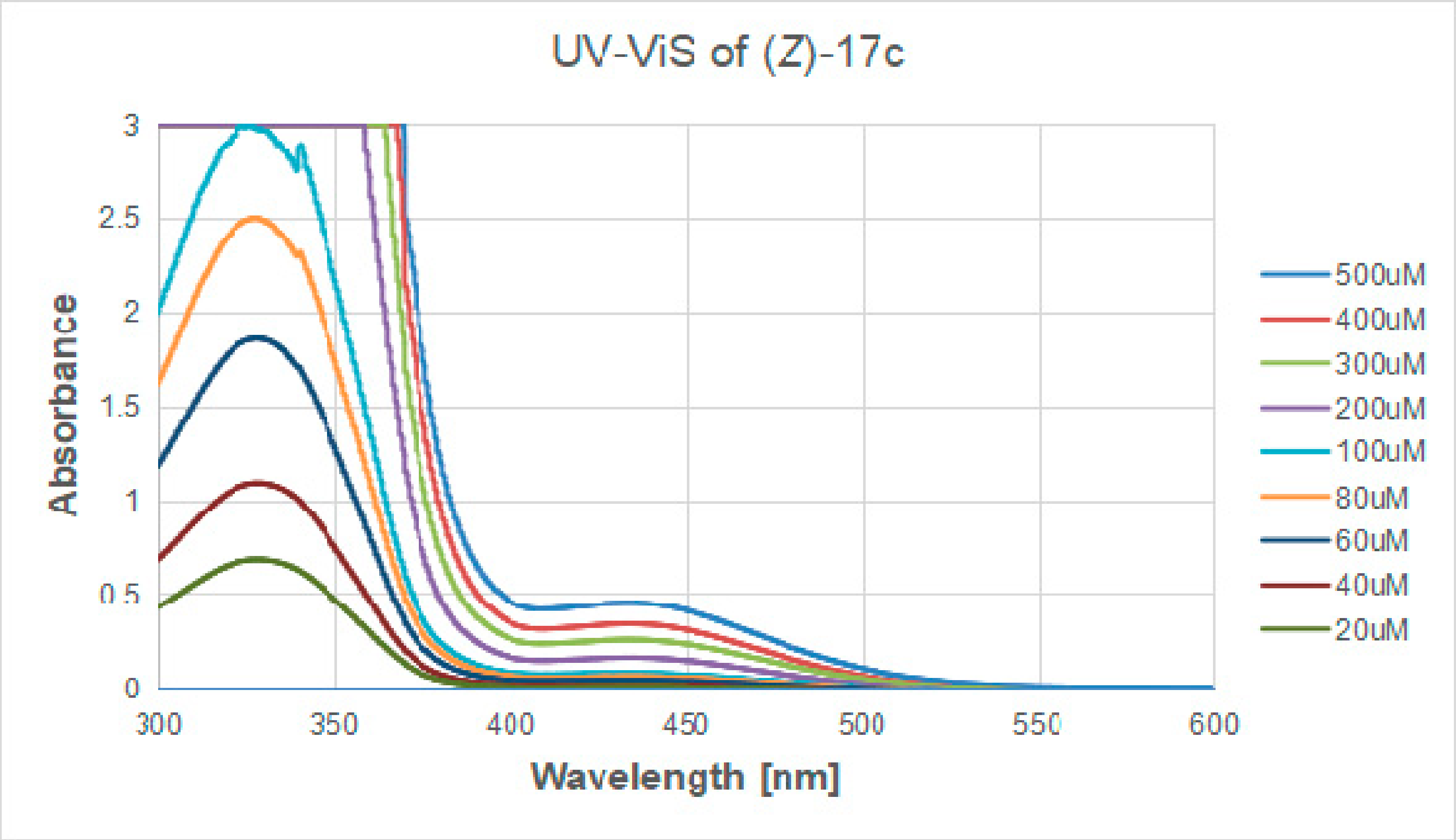

UV-ViS of (Z)-17c
Absorbance
3
2.5
2
1.5
1
0.5
0
300
350
400
450
500
550
600
Wavelength [nm]
500uM
400uM
300uM
200uM
100uM
80uM
60uM
40uM
20uM

17d:

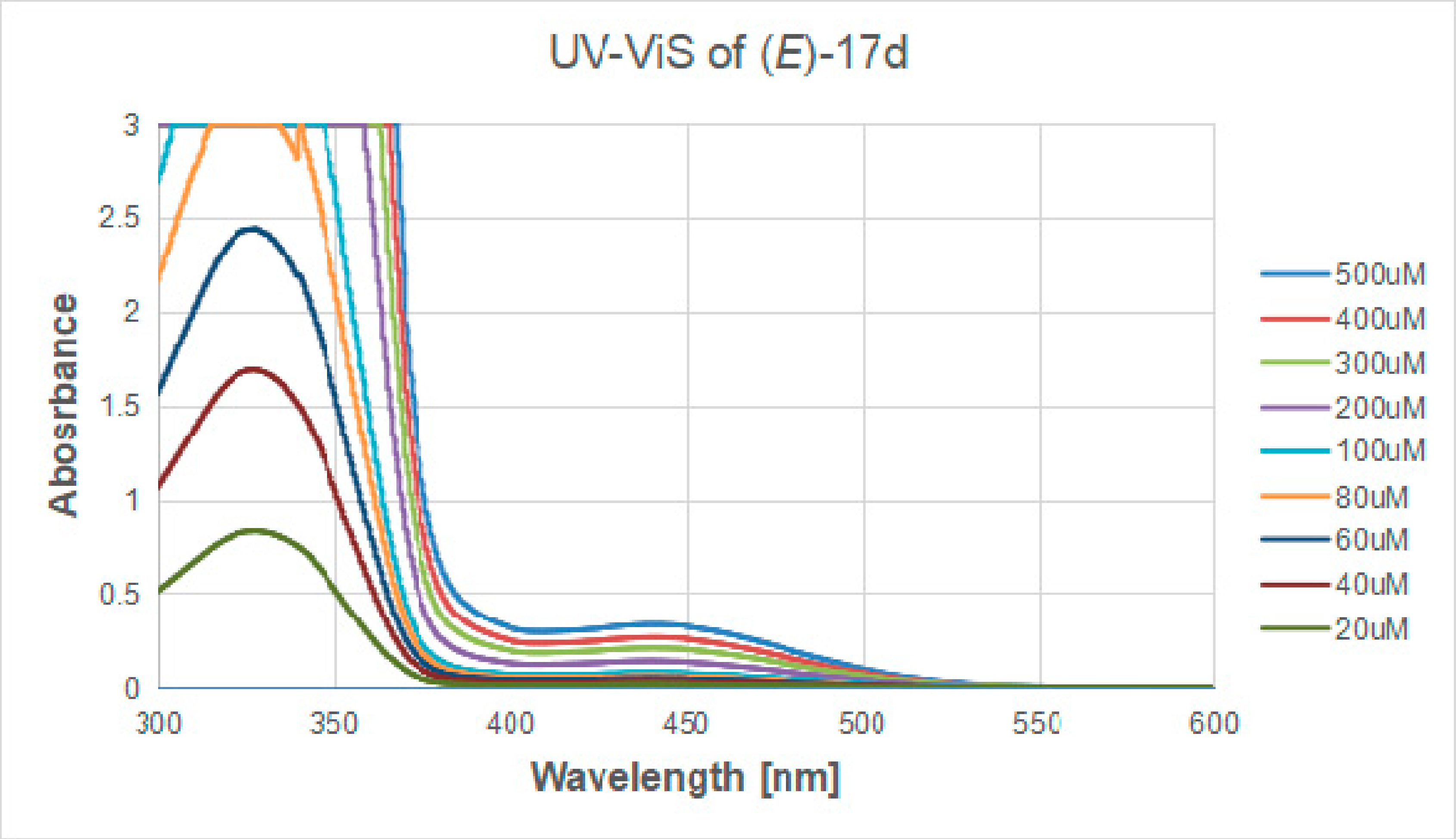
UV-ViS of (*E*)-17d
Abosrbance
Wavelength [nm]
3
2.5
2
1.5
1
0.5
0
300
350
400
450
500
550
600
500uM
400uM
300uM
200uM
100uM
80uM
60uM
40uM
20uM

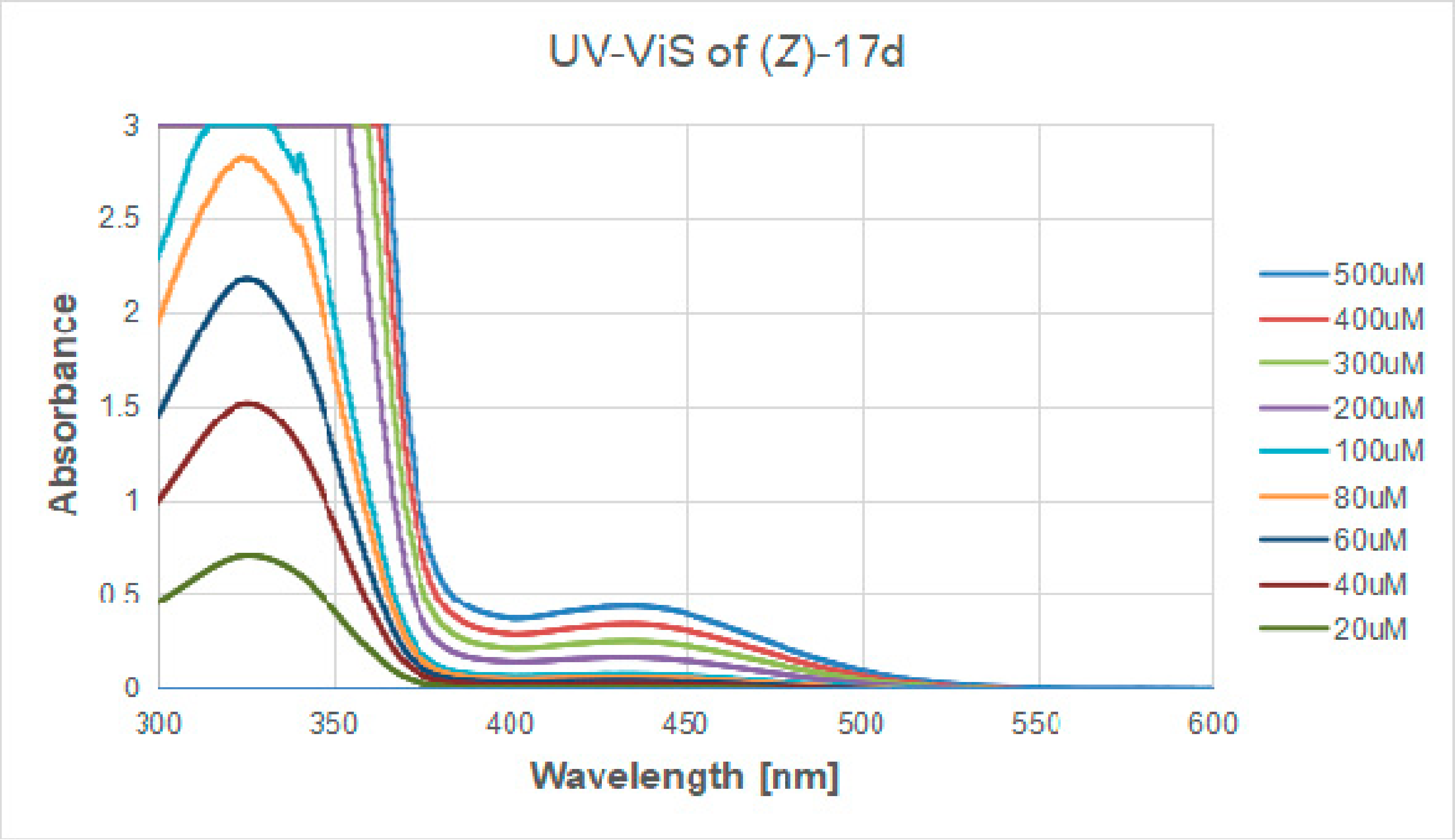

UV-ViS of (Z)-17d
Absorbance
Wavelength [nm]
0
0.5
1
1.5
2
2.5
3
300
350
400
450
500
550
600
500uM
400uM
300uM
200uM
100uM
80uM
60uM
40uM
20uM

17e:

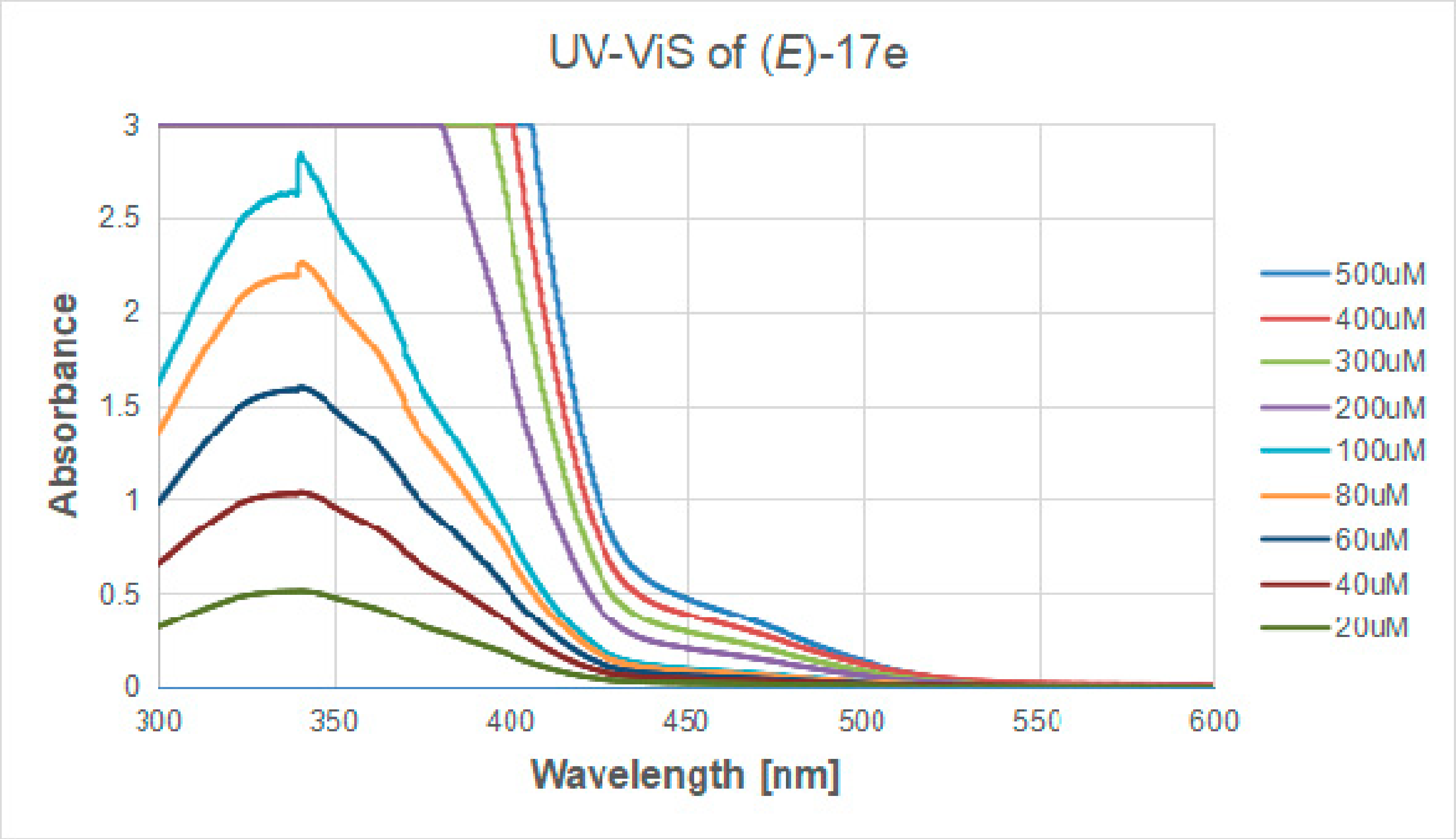

UV-ViS of (*E*)-17e
Absorbance
3
2.5
2
1.5
1
0.5
0
300
350
400
450
500
550
600
Wavelength [nm]
500uM
400uM
300uM
200uM
100uM
80uM
60uM
40uM
20uM

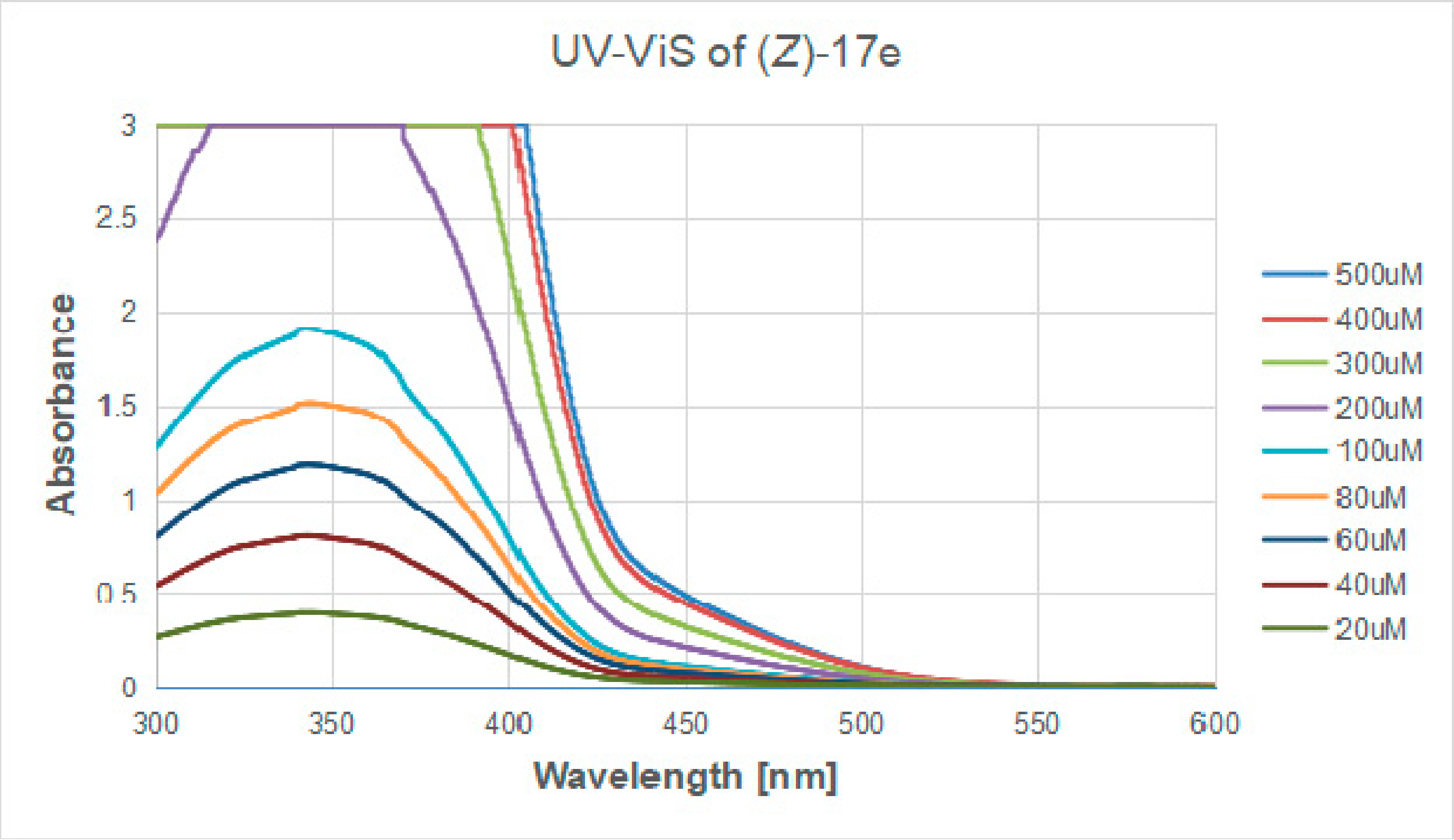
UV-ViS of (Z)-17e
Absorbance
Wavelength [nm]
3
2.5
2
1.5
1
0.5
0
300
350
400
450
500
550
600
500uM
400uM
300uM
200uM
100uM
80uM
60uM
40uM
20uM

17f:

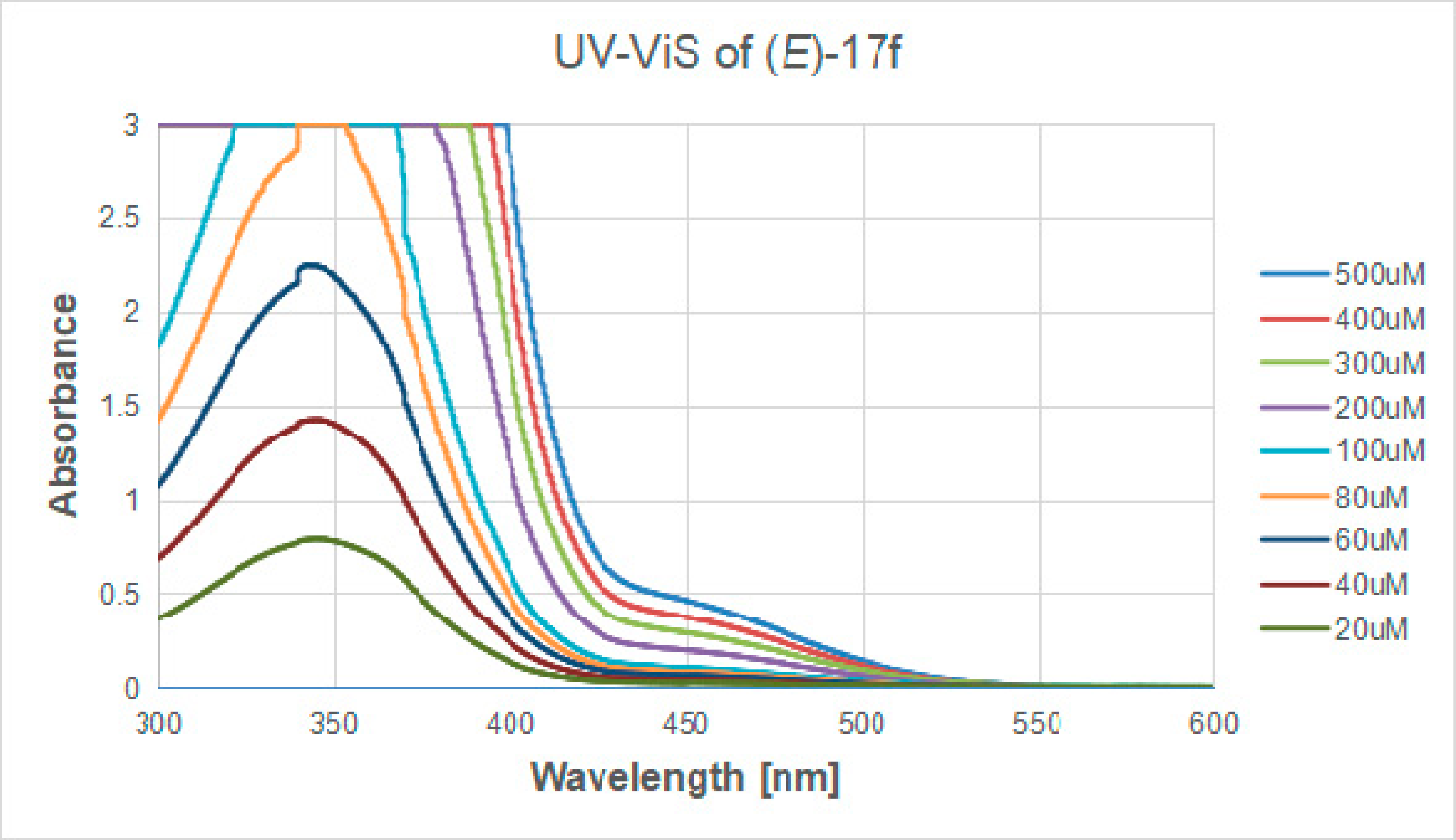
UV-ViS of (*E*)-17f
Absorbance
3
2.5
2
1.5
1
0.5
0
300
350
400
450
500
550
600
Wavelength [nm]
500uM
400uM
300uM
200uM
100uM
80uM
60uM
40uM
20uM

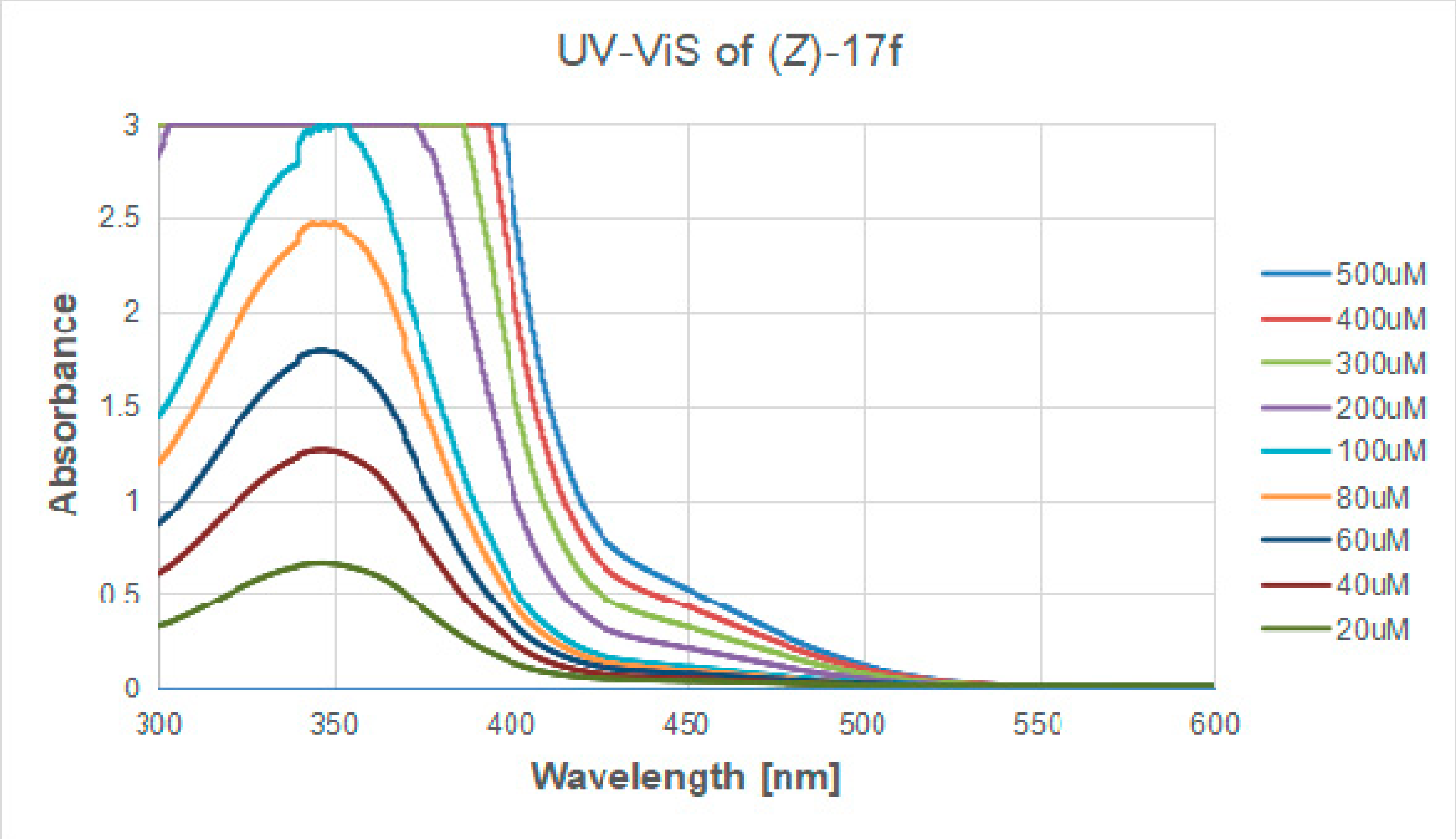
UV-ViS of (Z)-17f
Absorbance
Wavelength [nm]
3
2.5
2
1.5
1
0.5
0
300
350
400
450
500
550
600
500uM
400uM
300uM
200uM
100uM
80uM
60uM
40uM
20uM

17g:

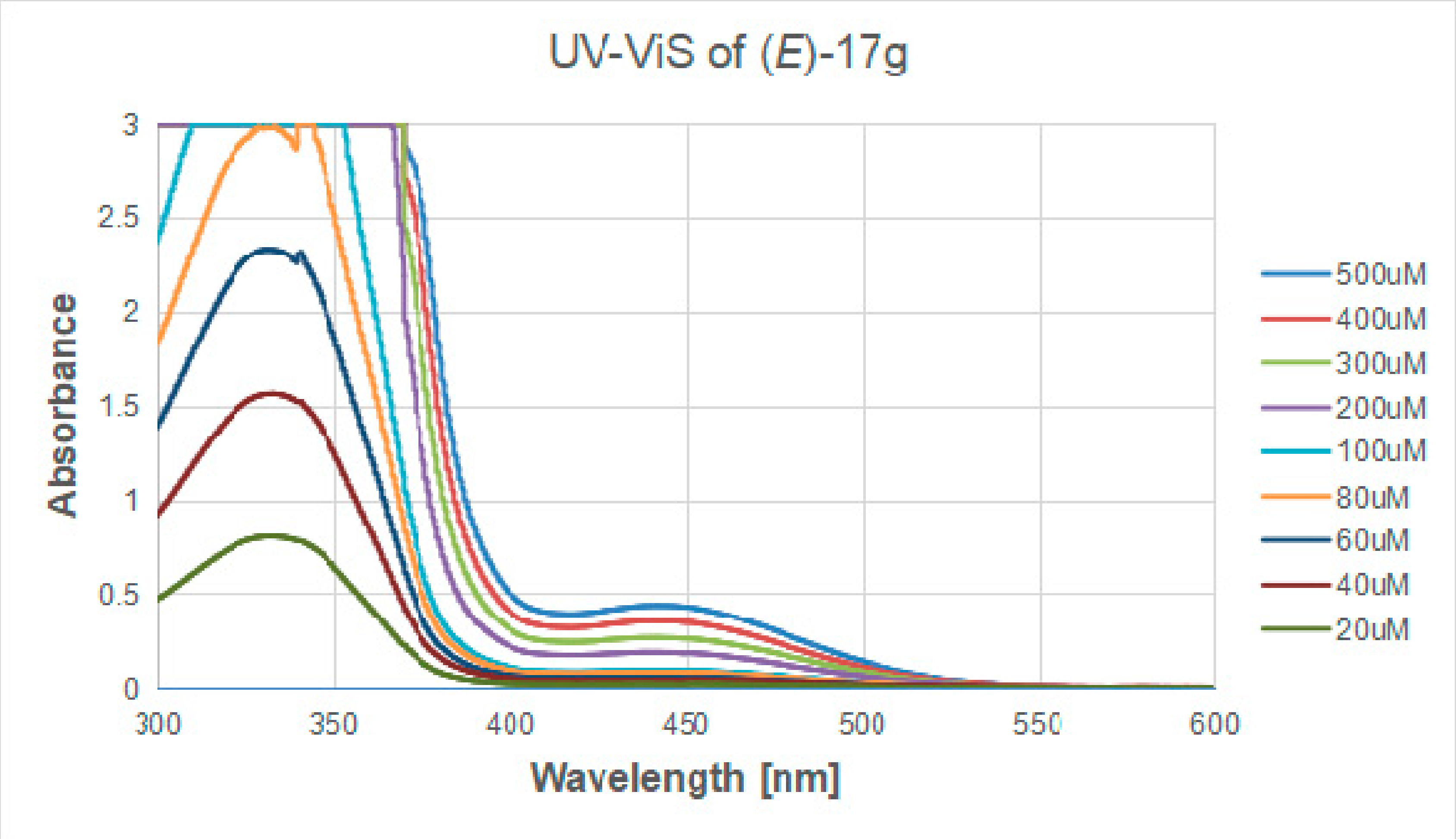

UV-ViS of (*E*)-17g
Absorbance
3
2.5
2
1.5
1
0.5
0
300
350
400
450
500
550
600
Wavelength [nm]
500uM
400uM
300uM
200uM
100uM
80uM
60uM
40uM
20uM

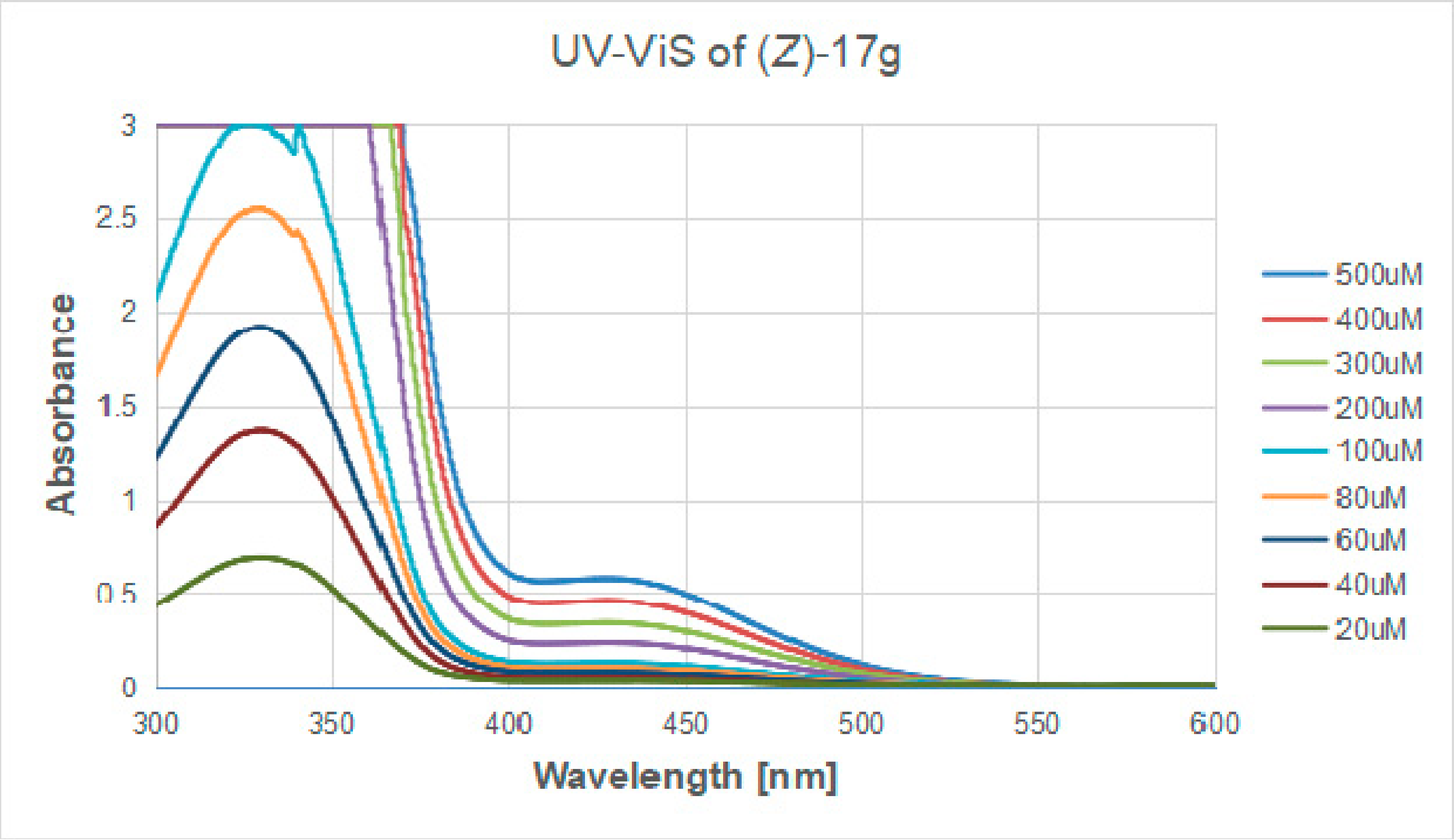
UV-ViS of (Z)-17g
Absorbance
3
2.5
2
1.5
1
0.5
0
300
350
400
450
500
550
600
Wavelength [nm]
500uM
400uM
300uM
200uM
100uM
80uM
60uM
40uM
20uM

17h:

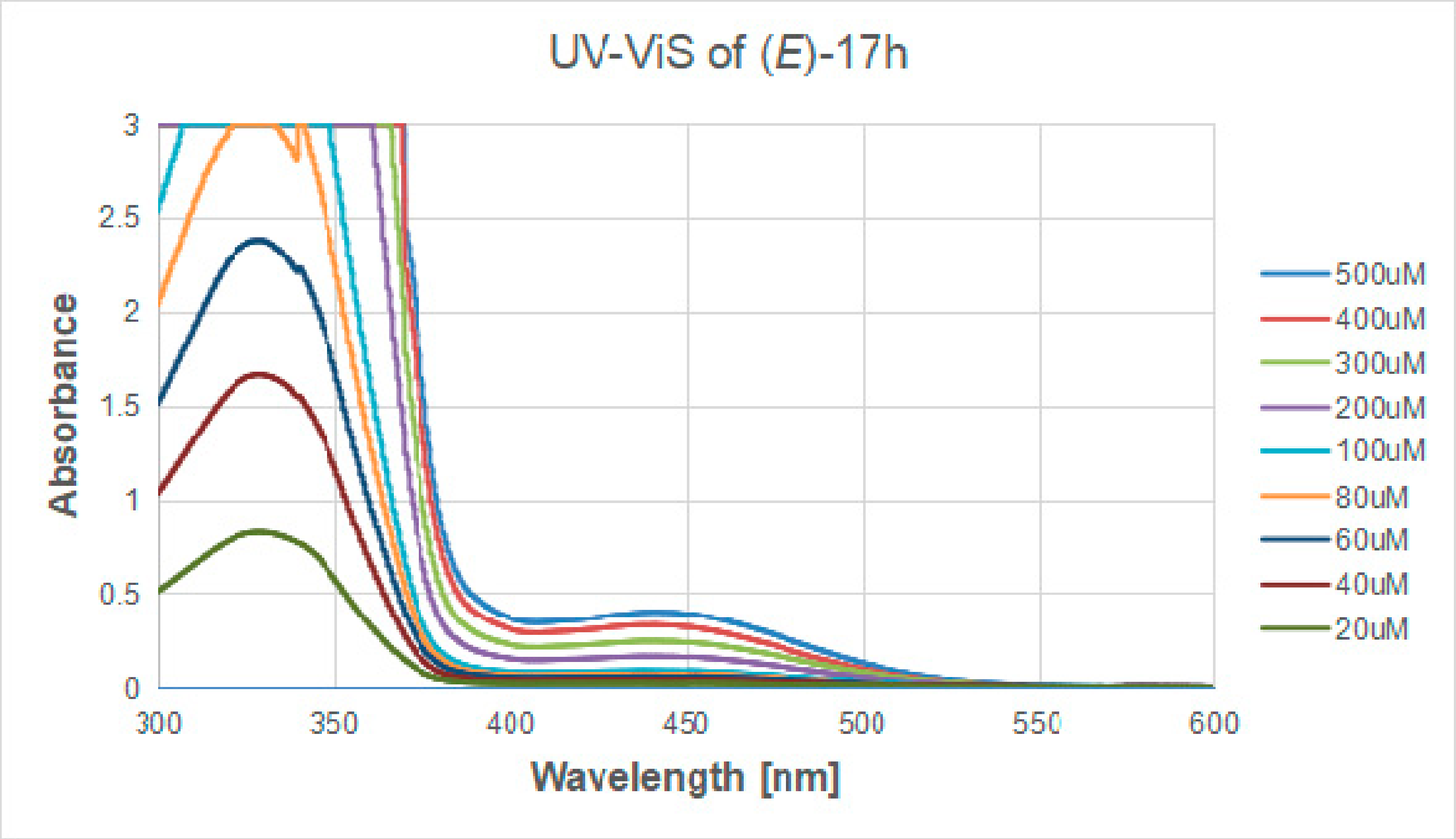

UV-ViS of (*E*)-17h
Absorbance
3
2.5
2
1.5
1
0.5
0
300
350
400
450
500
550
600
Wavelength [nm]
500uM
400uM
300uM
200uM
100uM
80uM
60uM
40uM
20uM

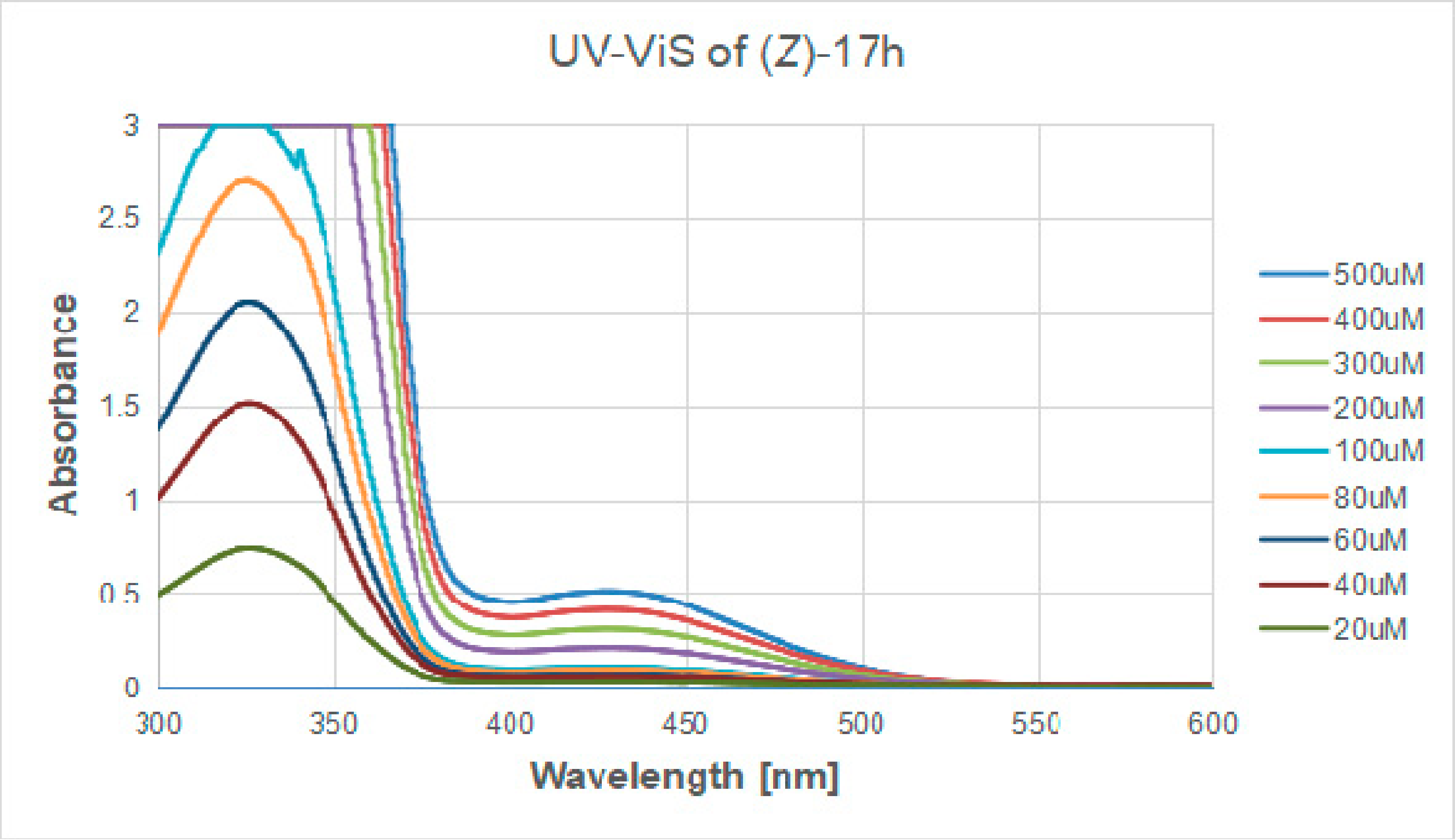

UV-ViS of (Z)-17h
Absorbance
Wavelength [nm]
3
2.5
2
1.5
1
0.5
0
300
350
400
450
500
550
600
500uM
400uM
300uM
200uM
100uM
80uM
60uM
40uM
20uM

17i:

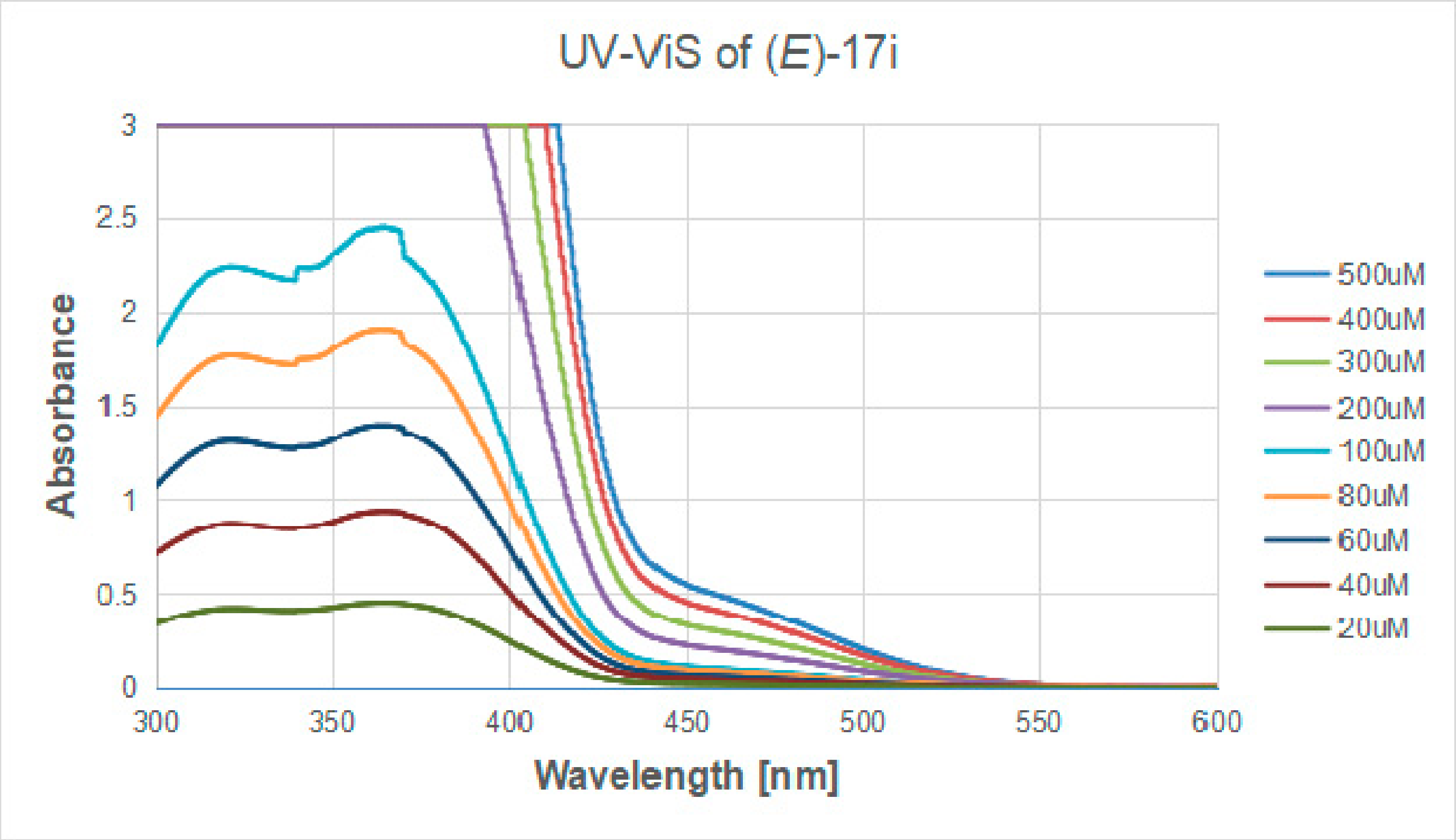

UV-ViS of (*E*)-17i
Absorbance
Wavelength [nm]
3
2.5
2
1.5
1
0.5
0
300
350
400
450
500
550
600
500uM
400uM
300uM
200uM
100uM
80uM
60uM
40uM
20uM

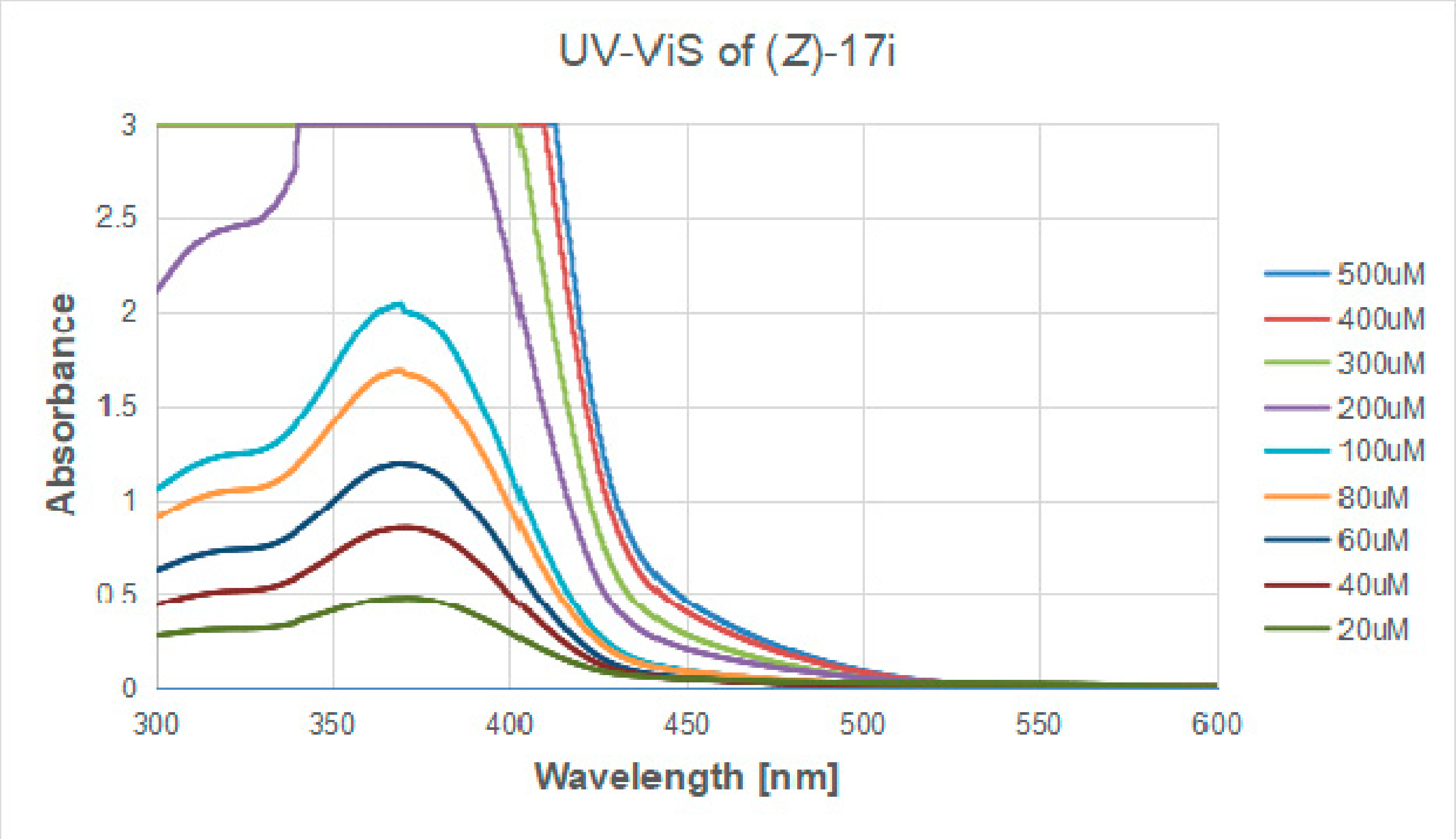

UV-ViS of (Z)-17i
Absorbance
Wavelength [nm]
0
0.5
1
1.5
2
2.5
3
300
350
400
450
500
550
600
500uM
400uM
300uM
200uM
100uM
80uM
60uM
40uM
20uM

17j:

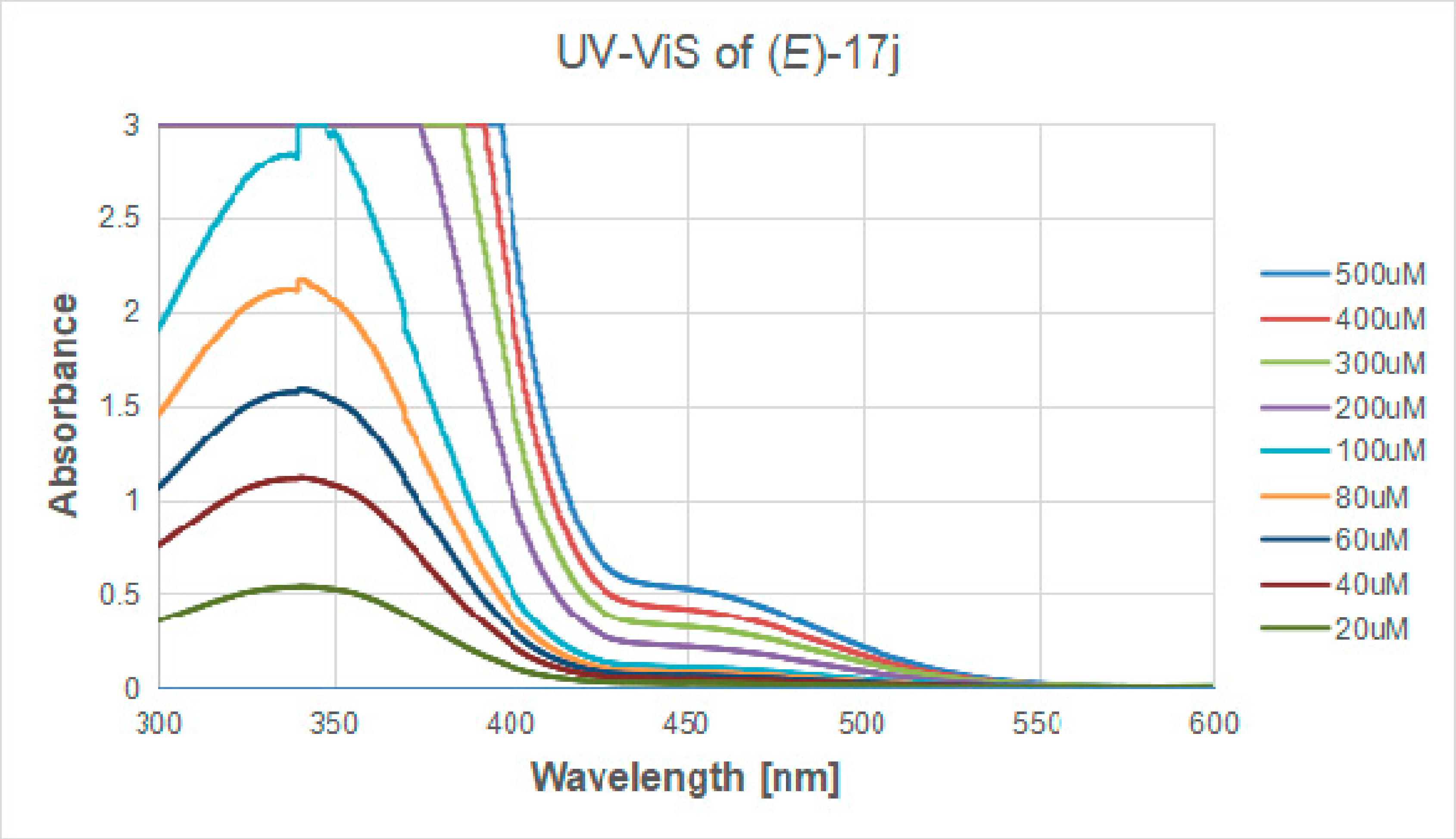
UV-ViS of (E)-17j
Absorbance
Wavelength [nm]
3
2.5
2
1.5
1
0.5
0
300
350
400
450
500
550
600
500uM
400uM
300uM
200uM
100uM
80uM
60uM
40uM
20uM

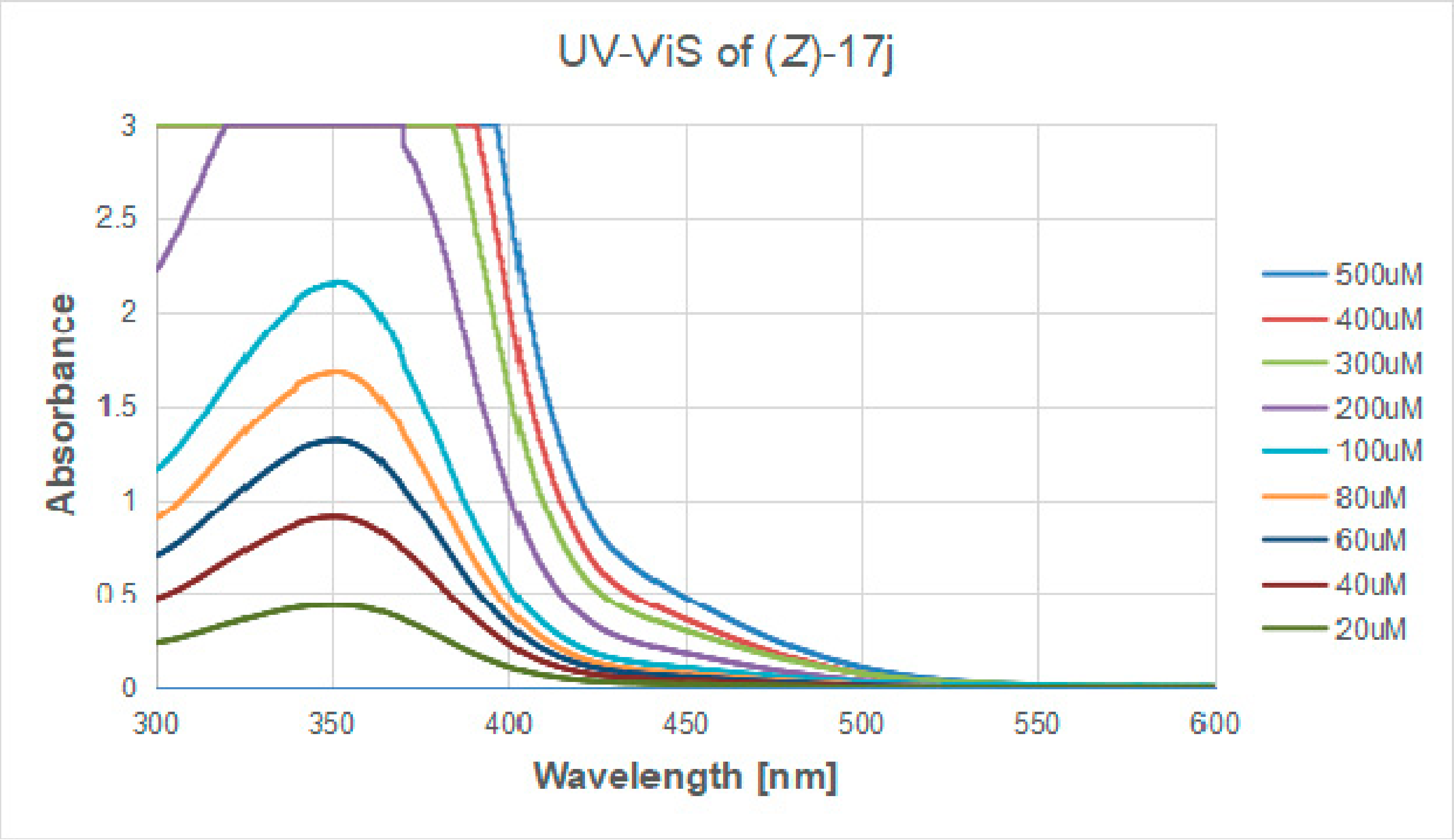
UV-ViS of (Z)-17j
Absorbance
Wavelength [nm]
3
2.5
2
1.5
1
0.5
0
300
350
400
450
500
550
600
500uM
400uM
300uM
200uM
100uM
80uM
60uM
40uM
20uM

17k:

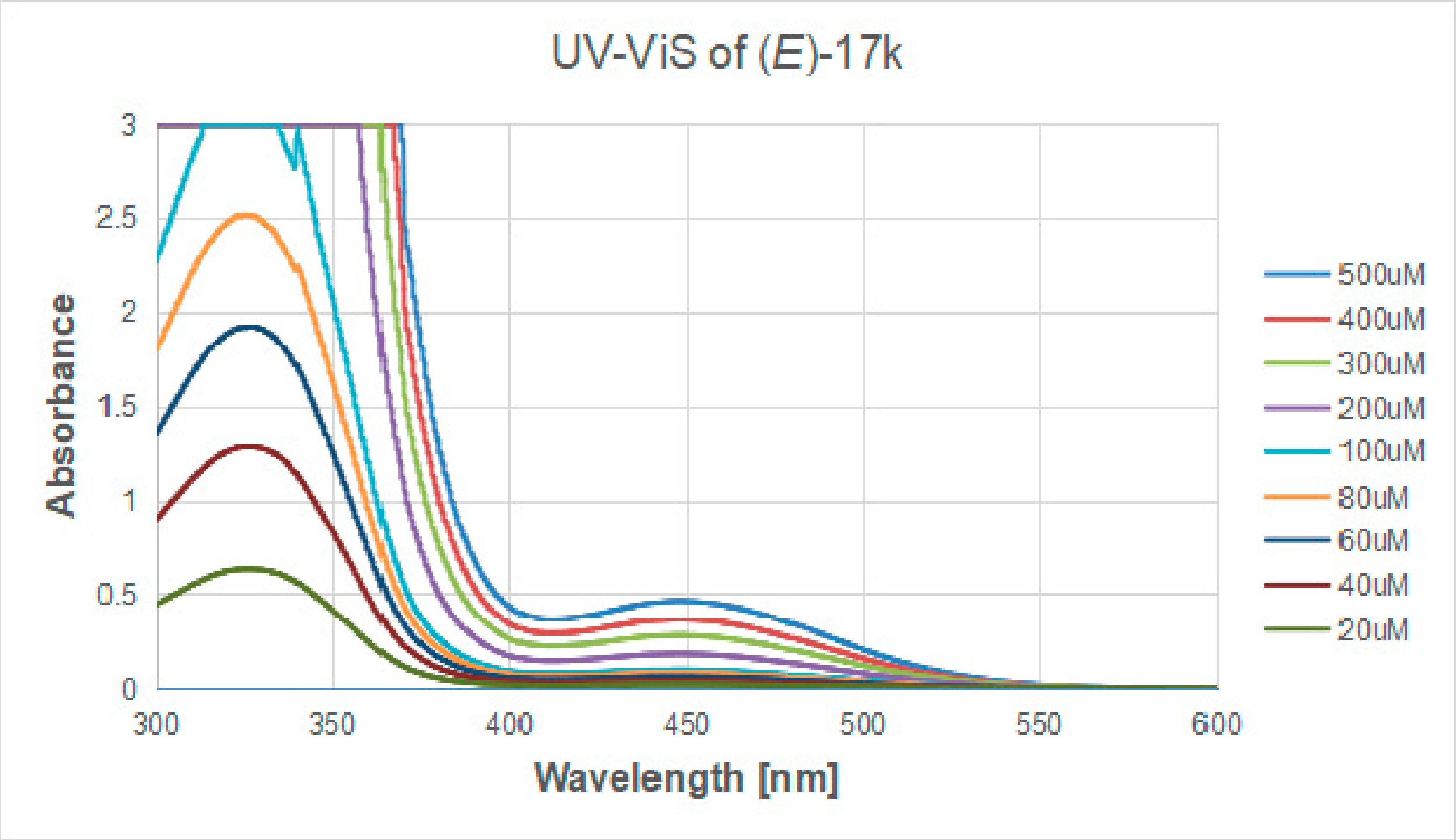

UV-ViS of (*E*)-17k
Absorbance
3
2.5
2
1.5
1
0.5
0
300
350
400
450
500
550
600
Wavelength [nm]
500uM
400uM
300uM
200uM
100uM
80uM
60uM
40uM
20uM

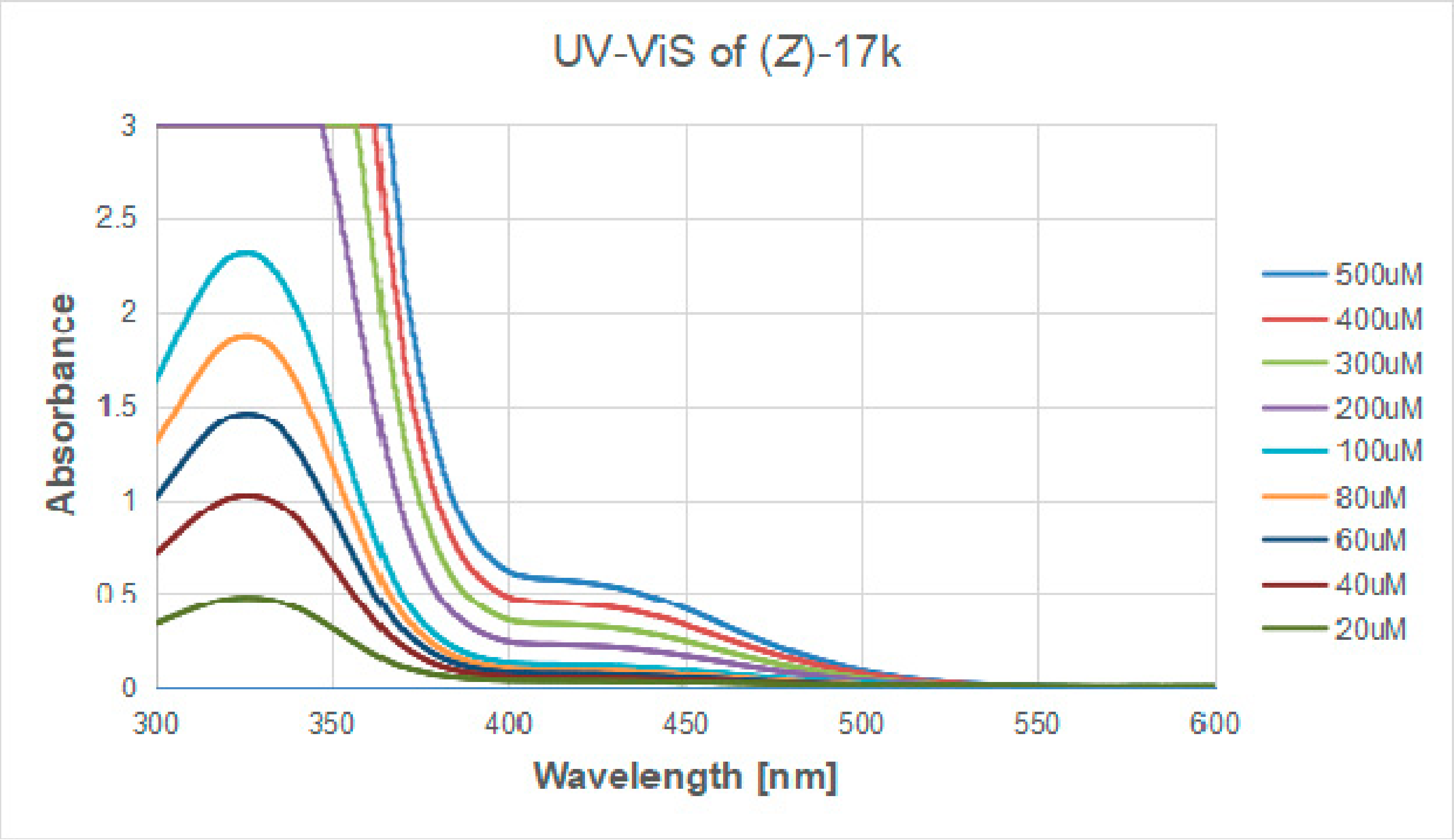
UV-ViS of (Z)-17k
Absorbance
Wavelength [nm]
500uM
400uM
300uM
200uM
100uM
80uM
60uM
40uM
20uM
0
0.5
1
1.5
2
2.5
3
300
350
400
450
500
550
600

17l:

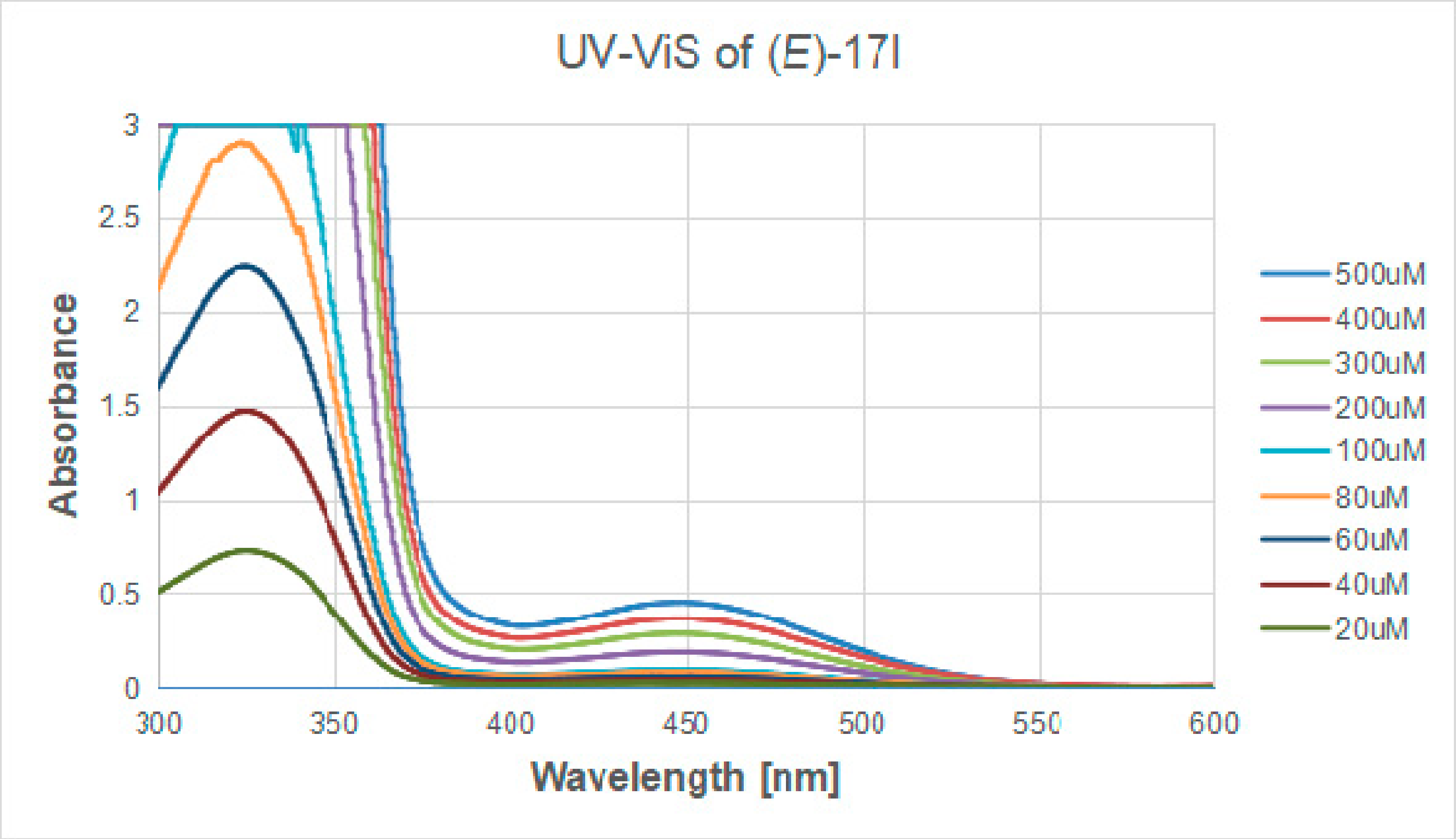

UV-ViS of (*E*)-17l
Absorbance
Wavelength [nm]
3
2.5
2
1.5
1
0.5
0
300
350
400
450
500
550
600
500uM
400uM
300uM
200uM
100uM
80uM
60uM
40uM
20uM

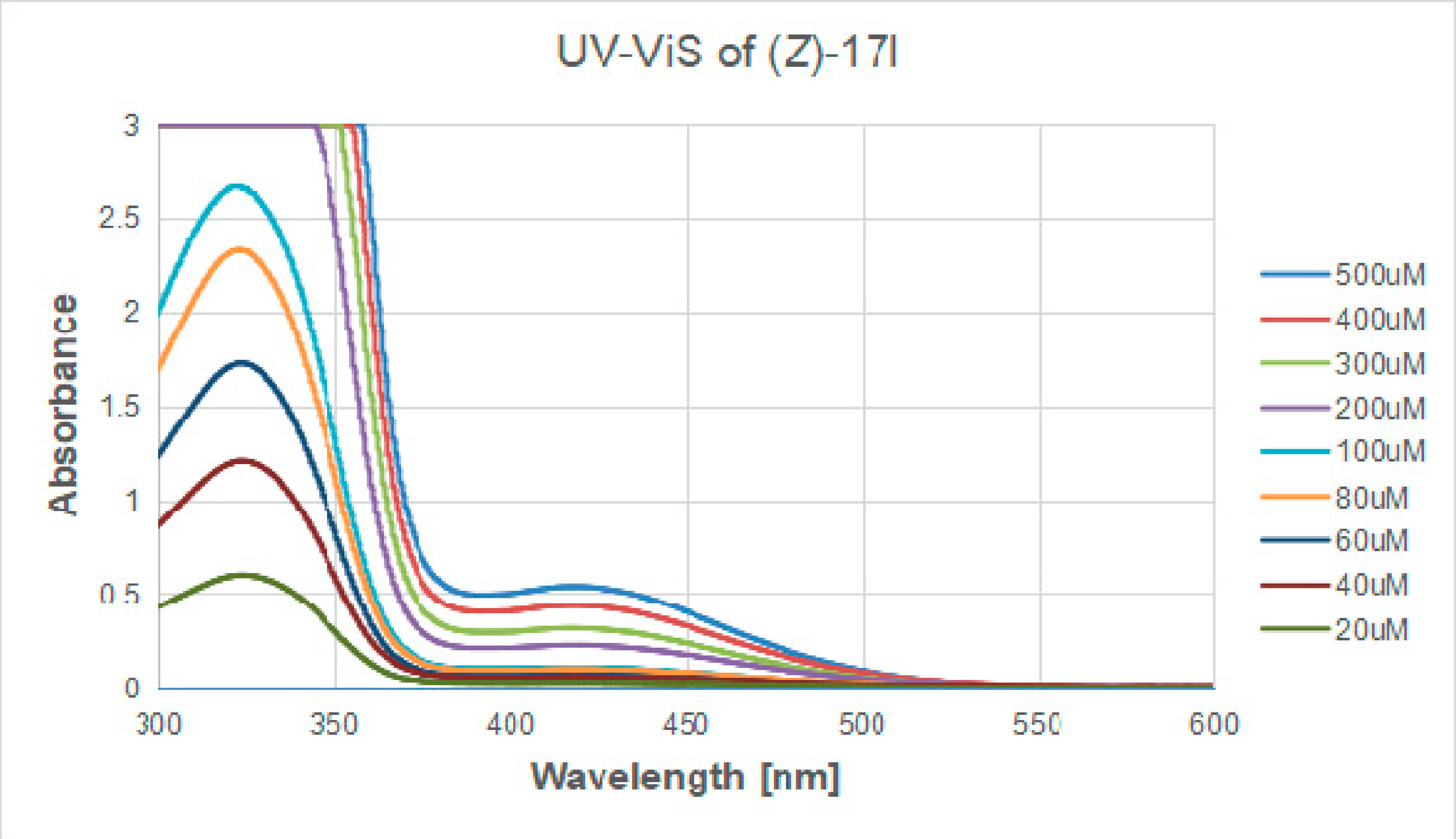
UV-ViS of (Z)-17l
Absorbance
Wavelength [nm]
3
2.5
2
1.5
1
0.5
0
300
350
400
450
500
550
600
500uM
400uM
300uM
200uM
100uM
80uM
60uM
40uM
20uM

5. References

----

[1] Frisch, M.J.; Trucks, G.W.; Schlegel, H.B.; Scuseria, G.E.; Robb, M.A.; Cheeseman, J.R.; Montgomery, J.A., Jr.; Vreven, T.; Kudin, K.N.; Burant, J.C.; et al. Gaussian 03, Revision C.02; Gaussian, Inc.: Wallingford, UK, 2004.

[2] J. Tomasi, B. Mennucci, R. Cammi, Quantum mechanical continuum solvation models, *Chem. Rev*. 105 (2005) 2999-3094. DOI: 10.1021/cr9904009.

[3] RCSB Protein Data Bank—RCSB PDB. Available online: http://www.rcsb.org/pdb/home/home.do (accessed 23 February 2024).

[4] O. Trott, A.J. Olson, AutoDock Vina: Improving the speed and accuracy of docking with a new scoring function, efficient optimization and multithreading, *J. Comput. Chem.* 31 (2010) 455–461. DOI: 10.1002/jcc.21334.

[5] E.F. Pettersen, T.D. Goddard, C.C. Huang, G.S. Couch, D.M. Greenblatt, E.C. Meng, T.E. Ferrin, UCSF Chimera-a visualization system for exploratory research and analysis, *J. Comput. Chem*. 25 (2004) 1605–1612. DOI: 10.1002/jcc.20084.

[6] M. Borowiak, W. Nahaboo, M. Reynders, K. Nekolla, P. Jalinot, J. Hasserodt, M. Rehberg, M. Delattre, S. Zahler, A. Vollmar, D. Trauner, O. Thorn-Seshold, Photoswitchable Inhibitors of Microtubule Dynamics Optically Control Mitosis and Cell Death, Cell 162 (2015) 403–411. DOI: 10.1016/j.cell.2015.06.049.

[7] A. Sailer, F. Ermer, Y. Kraus, F.H. Lutter, C.A. Donau, M. Bremerich, J. Ahlfeld, O. Thorn-Seshold, Hemithioindigos for Cellular Photopharmacology: Desymmetrised Molecular Switch Scaffolds Enabling Design Control over the Isomer-dependency of Potent Antimitotic Bioactivity, ChemBioChem 20 (2019) 1305-1314. DOI:10.1002/cbic.201800752.

[8] A. Sailer, J.C.M. Meiring, C. Heise, L.N. Pettersson, A. Akhmanova, J. Thorn-Seshold, O. Thorn-Seshold, Pyrrole Hemithioindigo Antimitotics with Near-quantitative Bidirectional Photoswitching Photocontrol Cellular Microtubule Dynamics with Single-cell Precision, Angew. Chem. Int. Ed. 60 (2021) 23695-23704. DOI:10.1002/anie.202104794.

[9] L. Gao, J.C.M. Meiring, Y. Kraus, M. Wranik, T. Weinert, S.D. Pritzl, R. Bingham, E. Ntouliou, K.I. Jansen, N. Olieric, A Robust, Gfp-orthogonal Photoswitchable Inhibitor Scaffold Extends Optical Control over the Microtubule Cytoskeleton. Cell Chem. Biol. 28 (2021) 228-241. DOI:10.1016/j.chembiol.2020.11.007.
